# Global Incidence of Diarrheal Diseases—An Update Using an Interpretable Predictive Model Based on XGBoost and SHAP: A Systematic Analysis

**DOI:** 10.3390/nu16183217

**Published:** 2024-09-23

**Authors:** Dan Liang, Li Wang, Shuang Liu, Shanglin Li, Xing Zhou, Yun Xiao, Panpan Zhong, Yanxi Chen, Changyi Wang, Shan Xu, Juan Su, Zhen Luo, Changwen Ke, Yingsi Lai

**Affiliations:** 1 Department of Immunology and Microbiology, College of Life Science and Technology, Jinan University, Guangzhou 510632, China; liangdancat@stu2022.jnu.edu.cn (D.L.); liushuang@stu.jnu.edu.cn (S.L.); zhouxing@stu2022.jnu.edu.cn (X.Z.); zpp2819195@stu.jnu.edu.cn (P.Z.); chenyanxi@stu2022.jnu.edu.cn (Y.C.); 2 Department of Medical Statistics, School of Public Health, Sun Yat-sen University, Guangzhou 510275, China; wangli237@mail2.sysu.edu.cn; 3 Department of Microbiology and Immunology, Basic Medicine College, Jinan University, Guangzhou 510632, China; lishanglinlsl@stu2021.jnu.edu.cn; 4 School of Public Health, Southern Medical University, Guangzhou 510515, China; 17369321541@163.com; 5 Department of Cardiovascular and Cerebrovascular and Diabetes Prevention and Treatment, Shenzhen Nanshan Center for Chronic Disease Control, Shenzhen 518000, China; wangchangyi2002@163.com (C.W.); m13823723231@163.com (S.X.); 6 Guangdong Provincial Key Laboratory for Emergency Detection and Research on Pathogen of Emerging Infectious Disease, Guangdong Provincial Center for Disease Control and Prevention, Guangdong Workstation for Emerging Infectious Disease Control and Prevention, Guangzhou 511430, China; susu05@126.com

**Keywords:** diarrheal disease, global burden of disease, epidemiology, incidence, forecasting, SHAP, XGBoost

## Abstract

Background: Diarrheal disease remains a significant public health issue, particularly affecting young children and older adults. Despite efforts to control and prevent these diseases, their incidence continues to be a global concern. Understanding the trends in diarrhea incidence and the factors influencing these trends is crucial for developing effective public health strategies. Objective: This study aimed to explore the temporal trends in diarrhea incidence and associated factors from 1990 to 2019 and to project the incidence for the period 2020–2040 at global, regional, and national levels. We aimed to identify key factors influencing these trends to inform future prevention and control strategies. Methods: The eXtreme Gradient Boosting (XGBoost) model was used to predict the incidence from 2020 to 2040 based on demographic, meteorological, water sanitation, and sanitation and hygiene indicators. SHapley Additive exPlanations (SHAP) value was performed to explain the impact of variables in the model on the incidence. Estimated annual percentage change (EAPC) was calculated to assess the temporal trends of age-standardized incidence rates (ASIRs) from 1990 to 2019 and from 2020 to 2040. Results: Globally, both incident cases and ASIRs of diarrhea increased between 2010 and 2019. The incident cases are expected to rise from 2020 to 2040, while the ASIRs and incidence rates are predicted to slightly decrease. During the observed (1990–2019) and predicted (2020–2040) periods, adults aged 60 years and above exhibited an upward trend in incidence rate as age increased, while children aged < 5 years consistently had the highest incident cases. The SHAP framework was applied to explain the model predictions. We identified several risk factors associated with an increased incidence of diarrhea, including age over 60 years, yearly precipitation exceeding 3000 mm, temperature above 20 °C for both maximum and minimum values, and vapor pressure deficit over 1500 Pa. A decreased incidence rate was associated with relative humidity over 60%, wind speed over 4 m/s, and populations with above 80% using safely managed drinking water services and over 40% using safely managed sanitation services. Conclusions: Diarrheal diseases are still serious public health concerns, with predicted increases in the incident cases despite decreasing ASIRs globally. Children aged < 5 years remain highly susceptible to diarrheal diseases, yet the incidence rate in the older adults aged 60 plus years still warrants additional attention. Additionally, more targeted efforts to improve access to safe drinking water and sanitation services are crucial for reducing the incidence of diarrheal diseases globally.

## 1. Introduction

Diarrhea is defined as passing three or more loose or liquid feces per day for three or more days and less than 14 days, with or without accompanying extra signs and symptoms, such as vomiting, nausea, fever, or abdominal pain [1,2]. Diarrhea is commonly classified into acute, chronic, and traveler’s diarrhea, each with distinct etiologies and health consequences. Acute diarrhea, which typically lasts less than 14 days, is most commonly caused by infections from viruses, bacteria, or parasites [3]. Chronic diarrhea, which lasts for over 4 weeks, may arise from non-infectious causes such as chronic inflammatory diseases, irritable bowel syndrome, or malabsorption disorders [4]. Traveler’s diarrhea is a form of acute diarrhea often triggered by consuming contaminated food or water during travel to regions with inadequate sanitation and is commonly associated with bacterial pathogens such as Escherichia coli [5]. Infectious agents, including viruses, bacteria, and parasites, are the most common causes of diarrhea, which can be transmitted through contaminated food or water or from person to person due to poor hygiene practices. However, climatic factors, such as increased temperatures and humidity, can exacerbate the spread of diarrheal diseases by promoting microbial growth. Inadequate access to clean water and sanitation, along with insufficient health policies, particularly in low- and middle-income countries, are also critical contributors to the global burden of diarrheal diseases [6,7]. These risk factors highlight the need for targeted interventions, especially in regions with poor infrastructure and high vulnerability to climate change.

Globally, the diarrheal disease was the second leading cause of mortality among children under five years, contributing to 370,000 deaths in 2019 [8]. Annually, the diarrheal disease occurs in nearly 1.7 billion children and kills approximately 525,000 children below the age of 5 [9]. GBD Diarrheal Disease collaborators displayed the global burden covering cases, deaths, and etiologies of diarrhea from 1990 to 2015, 2016, and 2017 in 195 countries classified through the socio-demographic index (SDI) [10,11,12]. Although the previous studies reported the status of diarrheal disease, gaps remain in providing updated data over time and predictive models that can capture complex temporal and non-linear trends effectively. Traditional models, such as the Age-Period-Cohort (APC) model and AutoRegressive Integrated Moving Average (ARIMA), commonly used in social sciences or epidemiological studies, have limitations in predicting complex data patterns. APC models, for instance, struggle to attribute observed changes to specific factors and tend to oversimplify complex interactions, while ARIMA models, often effective for linear trends, may fail in capturing the non-linear relationships typical of disease incidence [12,13]. In contrast to these models, XGBoost is widely recognized for its superior model-fitting capabilities in handling complex, non-linear relationships and has outperformed traditional models in various fields. For example, studies comparing XGBoost with ARIMA in predicting diseases like human brucellosis and COVID-19 demonstrated XGBoost’s superior ability to handle seasonality and non-linear trends, achieving lower predictive errors [14,15]. To fill these gaps and leverage the strengths of XGBoost, we employed it as our primary predictive model. XGBoost is a decision-tree-based ensemble machine learning algorithm utilizing a gradient boosting framework. Its ability to build models sequentially, with each model correcting the errors of the previous one, enhances its prediction accuracy over time. Furthermore, XGBoost’s flexibility in handling non-linear relationships between factors such as demographic, meteorological, and sanitation variables makes it particularly suitable for our analysis of diarrhea incidence, where traditional models like APC and ARIMA struggle to account for these complexities [16,17,18]. Additionally, XGBoost’s ability to incorporate regularization techniques reduces overfitting and increases generalizability, making it highly effective in real-world scenarios where data may be messy or violate statistical assumptions, as is often the case in environmental and health data [19]. 

Despite XGBoost’s predictive power, one limitation of machine learning models is their “black-box” nature, which makes it challenging to interpret how individual variables contribute to predictions. To address this, we employed SHAP, a cooperative game theory-based method, to interpret the contribution of individual features to the model’s predictions. SHAP assigns importance scores by measuring the change in model output when a feature is added to a baseline prediction, allowing us to understand the influence of variables such as meteorological conditions, water quality, and sanitation indicators on diarrhea incidence at both global and national levels [20]. This combination of XGBoost’s predictive power and SHAP’s interpretability provided a comprehensive framework, offering actionable insights into the role of different factors, which is essential for designing targeted interventions.

In this study, we comprehensively analyzed GBD (2019) data associated with all-cause diarrheal diseases from 1990 to 2019 at global, regional, and national levels, stratified by gender, age group, and SDI. We used the XGBoost model to predict future diarrhea incidence rates and applied SHAP to quantify the importance of key factors influencing these rates. The combination of XGBoost and SHAP offers a more advanced approach than traditional models, providing valuable guidance for public health practitioners and policymakers in addressing diarrheal diseases.

## 2. Methods

### 2.1. Data Sources, Definitions

The GBD dataset in 2019 analyzed 369 diseases and injuries and 87 risk factors in 204 countries or territories, 21 regions, and seven super-regions from 1990 to 2019 around the world [21]. Annual relevant data for diarrheal diseases in this study were sourced from the GBD dataset in 2019 by the Global Health Data Exchange query tool [22]. The SDI is a composite metric that measures per capita income, educational years, and the fertility rate among females under 25 years of age [21] and contains low SDI (the reference quintiles from 0 to 0.454743), low-middle SDI (from 0.454743 to 0.607679), middle SDI (from 0.607679 to 0.689504), middle-high SDI (from 0.689604 to 0.805129), and high SDI (from 0.805129 to 1) based on SDI quintiles [23,24,25]. The 204 countries and territories were also divided into 21 regions [21]. We extracted the incident cases and incidence rates of diarrheal diseases between 1990 and 2019 for location, age (from <5 to 80+ years in 5-year intervals), and gender with 95% uncertainty intervals (UIs).

### 2.2. Demographic, Meteorological, and Health Condition Data

Population data for the years 1990–2019 were retroactively derived from the number of cases/incidence rate, while projections for the years 2020–2040 were obtained from the Institute for Health Metrics and Evaluation (IHME) [26]. Meteorological variables, water sanitation, and sanitation and hygiene indicators were sourced from publicly available websites and were shown in Appendix S1. Meteorological factors were organized by nation or region into annual data. We predicted meteorological factor data for each nation or region up to the year 2040 based on historical meteorological data (Appendix S2). For water sanitation and hygiene variables, we collected data from 2000 to 2022 for various countries or regions. We constructed a log-linear model to estimate indicators for 1990–1999. For 2023–2040, we used two strategies: the first strategy involved using the 2022 data directly, and the second strategy was based on Sustainable Development Goals (SDGs) 6, aiming for universal and sustainable water and sanitation by 2030 [27]. Under this strategy, each nation’s or region’s proportions increased annually from 2023 to 2030 at a constant annual growth rate, gradually reaching 100% by 2030, and remained at 100% up to 2040. As the second strategy, while ideal, may be challenging to achieve in practice, our study focused on forecasting and analysis based on the first strategy while also predicting disease burden under the second strategy (Appendix S3). 

### 2.3. Statistical Analysis

EAPCs were generally used for analyzing trends in ASIRs and comparing their relative changes [28]. They were estimated based on a regression (Appendix S4). When the lower limit of the 95% UI of the EAPC is greater than zero, the ASIRs will display an increasing trend. Conversely, when the upper limit of the 95% UI of the EAPC is less than zero, the ASIRs will exhibit a decreasing trend. Otherwise, the ASIRs will remain a stable trend [29].

We utilized data from 1990 to 2019, including demographic information (gender, age, natural logarithm of population, and year), meteorological factors (including minimum, maximum, mean temperatures, wind speed, relative humidity, vapor pressure deficit, and precipitation), water sanitation, and sanitation and hygiene indicators (e.g., proportion of population using safely managed drinking water and sanitation services) as inputs to construct an XGBoost model for predicting the incidence rate of diarrheal disease. The model formula, describing the features used, is outlined below:

$$\begin{array}{l} {\left(Incidence\;rate\right)}_{y,c,s,a}\;\sim \;s+a+y+{\left(log\left(pnum\right)\right)}_{y,c,s,a}+{\left(mean\;temperature\right)}_{y,\;c}\\ +{\left(minimum\;temperature\right)}_{y,\;c}+{\left(maximum\;temperature\right)}_{y,\;c}\\ +{\left(vapor\;pressure\;deficit\right)}_{y,\;c}+{\left(relative\;humidity\right)}_{y,\;c}\\ +{\left(precipitation\right)}_{y,\;c}+{\left(wind\;speed\right)}_{y,\;c}\\ +{\left(proportion\;of\;population\;using\;safely\;managed\;drinking\;water\;services\;\left(\%\right)\right)}_{y,c}\\ +{\left(proportion\;of\;population\;using\;safely\;managed\;sanitation\;services\;\left(\%\right)\right)}_{y,c} \end{array}$$

where 

y represented the calendar years.

c denoted a specific nation or region.

s represented sex (0 for female and 1 for male).

a represented the midpoint age of an age group (e.g., a = 2 for ages under 5, a = 7 for ages 5–9, …, and a = 82 for ages 80 and above).

${\left(Incidence\;rate\right)}_{y,c,s,a}$ denoted the incidence rate in year y, for nation or region c, sex s, and age a.

${\left(log\left(pnum\right)\right)}_{y,c,s,a}$ represented the natural logarithm of population in year y, for nation *c*, sex s, and age a. 

XGBoost is a tree-based model that minimizes a composite objective function comprising a loss function l and a regularization term Ω. It constructs the model incrementally by adding new functions in each iteration to minimize the objective [14]. The formula is as follows:

$$L\left(\phi \right)=\sum_{i=1}^{n}l\left({\hat{y}}_{i},\;y_{i}\right)+\sum_{k=1}^{K}\Omega \left(f_{k}\right)$$

$$\Omega \left(f\right)=\gamma T+\frac{1}{2}\lambda \sum_{j=1}^{T}w_{j}^{2}$$

L(ϕ) is the objective function of the XGBoost model, composed of two terms: the sum of the loss function L(ϕ) over all samples and the regularization term $\Omega \left(f_{k}\right)$ over all leaf weights. 

$l\left({\hat{y}}_{i},\;y_{i}\right)$ is the loss function, which measures the discrepancy between the predicted ${\hat{y}}_{i}$ and observed value $y_{i}$.

$\Omega \left(f_{k}\right)$ is the regularization term, aiming to control the complexity of the model and prevent overfitting. 

Regularization parameters controlling the number of leaf nodes (γ) and the weight magnitude of leaf nodes (λ). 

T is the number of leaf nodes in the tree. 

$w_{j}$ is the weight of a leaf node.

We employed grid search to optimize the model’s hyperparameters. Each combination of hyperparameters was evaluated using 5-fold cross-validation, with the root mean square error (*RMSE*) used to assess the model’s predictive performance. To obtain prediction uncertainty intervals, we trained the model using 500 bootstrap samples. In addition, to mitigate overfitting during the training process with bootstrap samples, we utilized the out-of-bag (OOB) data from each bootstrap sample for monitoring. The *RMSE* of the OOB data was calculated, and an early stopping strategy was implemented to prevent model overfitting. The model was validated through 5-fold cross-validation, and metrics such as Pearson correlation coefficient, *RMSE*, and mean absolute percentage error (*MAPE*) were employed to assess its performance (Appendix S5). Based on the established model, we forecasted the incidence rates stratified by country or region, gender, and age group for each year from 2020 to 2040. Subsequently, the predicted results were utilized to calculate the incident cases and ASIRs. 

Additionally, SHAP was conducted to evaluate the effects of variables in the model on the incidence rate of diarrheal diseases. 

SHAP values are calculated based on the contribution of each feature to the model’s prediction, taking into account all possible combinations of feature subsets. The formula for SHAP value for feature j in a model with features *S* is [20]:

$$\phi_{j}=\underset{S\subseteq F\backslash \left\{j\right\}}{\sum }\frac{\left|S\right|!\left(\left|F\right|-\left|S\right|-1\right)!}{\left|F\right|!}\left[f\left(S\cup \left\{j\right\}\right)-f\left(S\right)\right]$$

$\phi_{j}$ is the SHAP value for feature j. It represents the contribution of feature j to the model’s prediction by averaging its marginal contributions across all possible subsets of the feature set F. 

S⊆F\{j} represents all possible subsets S of set F (which is the set of all features), excluding feature *j*. 

$\frac{\left|S\right|!\left(\left|F\right|-\left|S\right|-1\right)!}{\left|F\right|!}$ is a weight factor based on the size of the subset S.

|S|! is the factorial of the size of the subset *S*, representing the number of ways to order the subset S.

(|F|−|S|−1)! is the factorial of the remaining features not in *S*, minus 1 because we are excluding the feature j we are considering.

|F|! is the factorial of the total number of features in the full set F. This weighting factor ensures that the contribution of the feature is fairly averaged over all possible feature subsets of different sizes. 

f(S∪{j}) is the model’s prediction when feature j is included in the subset S. It represents the prediction made using the features in S combined with feature j.

f(S) is the model’s prediction when only the features in the subset S are used, without feature j.

All data cleaning, analysis, and visualization were conducted in R (version 4.3.2). The ‘xgboost’ package (version 1.7.7.1) was utilized for implementing XGBoost [14,30]. SHAP analysis for the variables was carried out using the ‘SHAPforxgboost’ package (version 0.1.3) [31]. 

### 2.4. Ethical Considerations

Our research constituted a secondary analysis of pre-existing data sources based on the 2019 GBD study, precluding the need for any original data collection or direct engagement with human subjects. Furthermore, the GBD data used in this study has been de-identified and aggregated, ensuring the privacy and confidentiality of the original participants; our analysis was not subject to the requirement for an additional IRB approval or the need to request an exemption [32].

## 3. Results

### 3.1. Sensitivity Analysis, Modeling Fitting, and Validation

To evaluate the accuracy and robustness of model predictions under different hyperparameter combinations, we employed a grid search method, comparing the predictive performance of models across various hyperparameter combinations (evaluated using 5-fold cross-validation and computing the *RMSE* metric). The results indicated that the XGBoost model was sensitive to parameters such as maximum depth of the trees (*max depth*) and learning rate (*eta*); adjusting these parameters appropriately could enhance both predictive accuracy and model robustness. Based on the sensitivity analysis results, we selected the optimal hyperparameter combination (*eta* = 0.05, *max_depth* = 10, and *nrounds* = 6000) (Figure S1). Based on data from 1990 to 2019, we applied 5-fold cross validation to evaluate the XGBoost model’s performance in predicting incidence rates of diarrheal disease, resulting in an *RMSE* of 4050.81 per 100,000 persons and a *MAPE* of 0.50%, along with a Pearson correlation coefficient of 1.00 between observation and prediction of incidence rate. The results demonstrated the XGBoost model exhibited superior predictive accuracy (Figure S2). 

### 3.2. Diarrheal Disease Profiles

**Figure 1:**
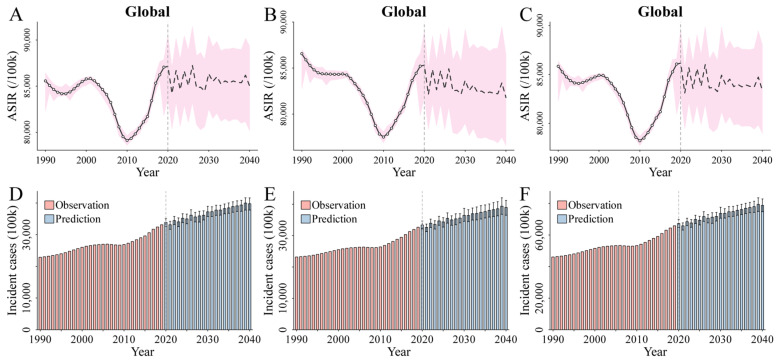
(**A**–**C**) The trends and projections of ASIRs per 100,000 in diarrheal diseases from 1990 to 2040 at the global level by genders ((**A**), males, (**B**) females, and (**C**) both genders combined). The open spots correspond to the observations between 1990 and 2019, and the pink shadow indicates the 95% UIs of the predictions. The mean of the predictions is displayed as a black line (solid line for 1990–2019 and dashed line for 2020 to 2040), and a vertical dashed line denotes the start year of the prediction. (**D**–**F**) The trends in the incident cases of diarrheal diseases from 1990 to 2040 at the global level for males (**D**), females (**E**), and both genders combined (**F**). The black error bar refers to the 95% UIs of the predictions. Abbreviations: ASIRs, age-standardized incidence rates; UIs, 95% uncertainty intervals.

Globally, the ASIRs associated with diarrheal diseases for males (Figure 1A), females (Figure 1B), and both genders combined (Figure 1C) changed from 85,567.97, 86,562.28, and 85,833.63 per 100,000 population in 1990 to 87,059.34, 85,207.31, and 86,061.73 per 100,000 population in 2019, respectively, and EAPCs were −0.12 (95% UI [−0.23, −0.01]) for males, −0.22 (95% UI [−0.33, −0.10]) for females, and −0.16 (95% UI [−0.28, −0.05]) for both genders combined between 1990 and 2019 (Table 1 and Tables S1 and S2). The ASIRs for males (Figure 1A), females (Figure 1B), and both genders combined (Figure 1C) were predicted to decrease from 87,156.29, 85,316.42, and 86,161.16 per 100,000 population in 2020 to 85,024.07, 81,792.99, and 83,349.25 per 100,000 population in 2040, and EAPCs were predicted to −0.22 (95% UI [−0.10, −0.05]) for males, −0.11 (95% UI [−0.19, −0.03]) for females, and −0.07 (95% UI [−0.14, −0.01]) for both genders combined from 2020 to 2040 (Table 1 and Tables S1 and S2).

Table 1 Incident cases and ASIRs per 100,000 persons of diarrheal diseases in 1990, 2019, 2020, and 2040 for males, females, and both genders combined by GBD regions, and EAPCs (95% UIs) of ASIRs for 1990–2040. Abbreviations: ASIRs, age-standardized incidence rates; GBD, global burden of disease; EAPCs, estimated annual percentage changes; UIs, 95% uncertainty intervals.

The global incident cases of the diarrheal diseases for males (Figure 1D), females (Figure 1E), and both genders combined (Figure 1F) were 22,871.76, 23,177.75, and 46,049.51 hundred thousand in 1990 and 33,150.58, 32,666.25, and 65,816.83 hundred thousand in 2019, respectively (Table 1 and Table S3). Over the same time, the global incidence rates of the diarrheal diseases for males, females, and both genders combined were 84,906.78, 87,262.41, and 86,076.30 per 100,000 in 1990 and 85,418.19, 84,704.62, and 85,062.53 per 100,000, respectively, in 2019 (Table S4). The number of diarrhea cases for males (Figure 1D), females (Figure 1E), and both genders combined (Figure 1F) were predicted to increase to 33,883.55, 33,379.24, and 67,262.79 hundred thousand in 2020 and 39,771.12, 38,959.60, and 78,730.72 hundred thousand in 2040, respectively (Table 1 and Table S3). Over the same period, the global incidence rates of the diarrheal diseases for males, females, and both genders combined were predicted to increase to 85,619.44, 84,951.02, and 85,286.43 per 100,000 in 2020 and 86,582.30, 84,960.06, and 85,771.87 per 100,000 in 2040, respectively (Table S4).

At the regional level, the highest increase in ASIRs of diarrheal diseases per 100,000 were found in North Africa and the Middle East (EAPC, 1.25 (95% UI [1.17, 1.34])), Central Sub-Saharan Africa (EAPC, 1.08 (95% UI [1.00, 1.16])), and Andean Latin America (EAPC, 1.08 (95% UI [0.99, 1.16])) between 1990 and 2019. Over the same time, the highest decrease in ARISs of diarrheal diseases per 100,000 were found in Central Latin America (EAPC, −1.41 (95% UI [−1.50, −1.32])), South Asia (EAPC, −1.39 (95% UI [−1.65, −1.13])), and Central Europe (EAPC, −0.61 (95% UI [−0.67, −0.56])) (Table 1, Tables S1 and S2). The fastest increase in ASIRs of diarrheal diseases per 100,000 was projected to occur in High-income Asia Pacific (EAPC, 0.74 (95% UI [0.18, 1.31])), Eastern Sub-Saharan Africa (EAPC, 0.52 (95% UI [0.49, 0.56])), and East Asia (EAPC, 0.37 (95% UI [0.34, 0.40])) between 2020 and 2040. At the same time, the highest decrease in ARISs of diarrheal diseases per 100,000 was projected to occur in High-middle SDI (EAPC, −0.45 (95% UI [−0.56, −0.34])), Oceania (EAPC, −0.31 (95% UI [−0.35, −0.28])), and Andean Latin America (EAPC, −0.31 (95% UI [−0.41, −0.20])) (Table 1, Tables S1 and S2).

**Figure 2:**
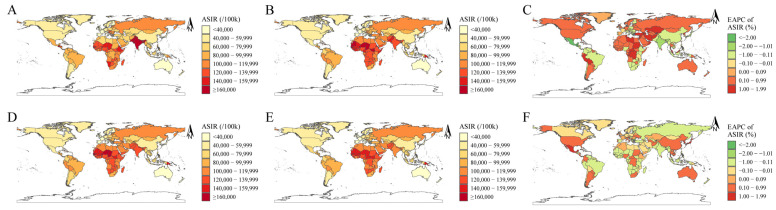
(**A**,**B**,**D**,**E**) The distribution in ASIRs per 100,000 persons for diarrheal diseases at the national level in 1990 (**A**), 2019 (**B**), 2020 (**D**), and 2040 (**E**). (**C**,**F**) The EAPCs in ASIRs for diarrheal diseases at the national level from 1990 to 2019 (**C**) and from 2020 to 2040 (**F**). Abbreviations: ASIRs, age-standardized incidence rates; EAPCs, estimated annual percentage changes.

Nationally, the highest increase in ASIRs of diarrheal diseases per 100,000 were found in Turkey (EAPC, 1.66 (95% UI [1.56, 1.76])), followed by Afghanistan (EAPC, 1.57 (95% UI [1.45, 1.68])) and Libya (EAPC, 1.55 (95% UI [1.43, 1.66])) between 1990 and 2019. Over the same period, the highest decrease in ARISs of diarrheal diseases per 100,000 were found in Guatemala (EAPC, −2.33 (95% UI [−2.40, −2.26])), Mexico (EAPC, −2.12 (95% UI [−2.29, −1.96])), and India (EAPC, −1.62 (95% UI [−1.94, −1.30])) (Figure 2A–C, Table 2, Table 3, Tables S1 and S2). The fastest increase in ASIRs of diarrheal diseases per 100,000 were projected to occur in Mexico (EAPC, 1.93 (95% UI [1.59, 2.28])), Japan (EAPC, 1.79 (95% UI [1.21, 2.38])), and the Republic of Korea (EAPC, 1.78 (95% UI [1.28, 2.29])) between 2020 and 2040. Over the same time, the highest decrease in ASIRs of diarrheal diseases per 100,000 were projected to occur in Bulgaria (EAPC, −4.02 (95% UI [−5.03, −3.00])), United Arab Emirates (EAPC, −1.02 (95% UI [−1.95, −0.07])), and Hungary (EAPC, −0.81 (95% UI [−1.09, −0.53])) (Figure 2D–F and Table 2, Table 3, Tables S1 and S2).

**Figure 3:**
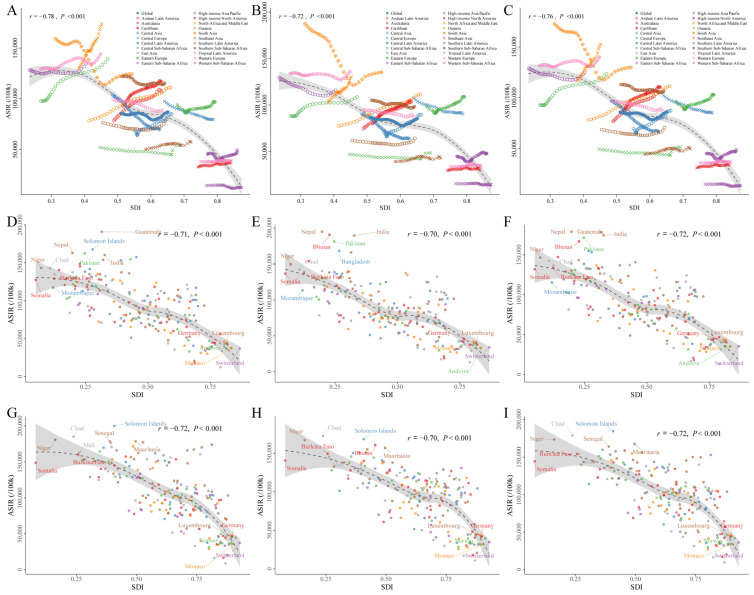
(**A**–**C**) ASIRs per 100,000 persons of diarrheal diseases at the global and regional levels by SDIs from 1990 to 2019 for males (**A**), females (**B**), and both genders combined (**C**). The predictions based on SDI and ASIRs in all 21 GBD regions are displayed as the solid black line, and the grey shadow indicates the 95% UIs of the predictions. (**D**–**I**) ASIRs per 100,000 in diarrheal diseases at the national level by SDI in 1990 for male (**D**), female (**E**), and both genders combined (**F**) and in 2019 for male (**G**), female (**H**), and both genders combined (**I**). The predictions based on SDI and ASIRs in all 204 countries or territories are demonstrated as the solid black line, and the grey shadow indicates the 95% UIs of the predictions. Abbreviations: ASIRs, age-standardized incidence rates; SDI, socio-demographic index; GBD, global burden of disease.

Figure 3A–C demonstrated the ASIRs by SDI and GBD regions between 1990 and 2019 for males, females, and both genders combined, for all causes of diarrheal diseases. In general, there was a negative correlation between ASIRs and SDIs for diarrheal diseases between 1990 and 2019 (for males: *r* = −0.78, *p* < 0.001; for females: *r* = −0.72, *p* < 0.001; for both genders combined: *r* = −0.76, *p* < 0.001). SDI-based modes were similar in terms of gender: the top location of ASIRs between 1990 and 2019 in the lowest SDI quintile was South Asia for both males and females, whereas in the highest SDI quintile it was High-income North America. Additionally, some regions showed an increase trend consistently in ASIRs spanning three decades, such as Oceania, Central Sub-Saharan Africa, the Caribbean, and Andean Latin America, whereas regions with a higher baseline SDI had generally experienced minimal changes in ASIRs over a period of three decades despite advances in SDI, such as Western Europe, Australasia, and High-income Asia Pacific. Furthermore, some regions initially experienced decreasing ASIRs as SDI increased, followed by increasing ASIRs as SDI continued to increase over time, as seen in South Asia, Tropical Latin America, Central Asia, Eastern Europe, and High-income North America. We also demonstrated the ASIRs by SDI and the national level for males, females, and both genders combined in 1990 (Figure 3D–F) and 2019 (Figure 3G–I).

**Figure 4:**
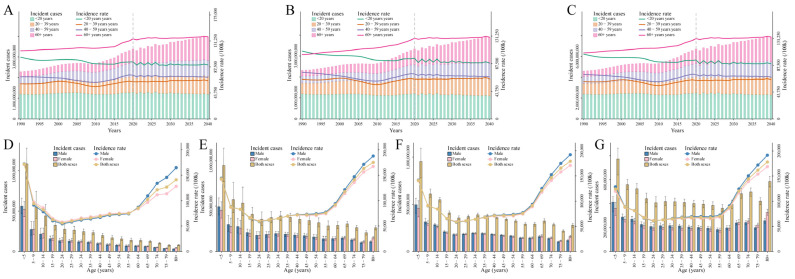
(**A**–**C**) The variation trends of incident cases and incidence rates for diarrheal diseases from 1990 to 2040 in all age groups in 20-year intervals at the global level for males (**A**), females (**B**), and both genders combined (**C**). A vertical dashed line denotes the start year of the prediction. (**D**–**G**) The variation trends of incident cases and incidence rates for diarrheal diseases in all age groups in 5-year intervals at the global level for both genders combined in 1990 (**D**), 2019 (**E**), 2020 (**F**), and 2040 (**G**).

To explore the temporal trends in age over time during the observation and prediction periods, the individuals were divided into four age groups from under 20 to 60 plus years in 20-year intervals for males (Figure 4A), females (Figure 4B), and both genders combined (Figure 4C) (Table S5) [32]. For all age groups, the incidence rates of diarrheal diseases were projected to remain relatively stable from 1990 to 2040. For the group under 20 years, the incidence rate slightly decreased, with projections showing a reduction from 105,560.57 per 1,000,000 in 1990 to 89,877.16 per 100,000 in 2040 (Figure 4C and Table S5). In contrast, the incidence rate for the 60+ years group was projected to increase slightly, rising from 107,596.20 per 100,000 in 1990 to 133,587.36 per 100,000 in 2040 (Figure 4C and Table S5).

In addition, the population was arranged into 17 age groups from under five years to 80 plus years in 5-year intervals to describe the age distribution of the population affected by diarrheal diseases. Globally, the highest number of incident cases was observed in children under 5 years old from 1990 to 2019, with the number of cases being 1084.65 million in 1990 (Figure 4D) and 947.67 million in 2019 (Figure 4E). The corresponding incidence rates were 171,590.87 per 100,000 and 142,970.56 per 100,000, respectively (Table S6). This trend was predicted to persist between 2020 and 2040, with the incidence rate for children under 5 years old expected to decline from 141,202.37 per 100,000 in 2020 (Figure 4F) to 124,369.35 per 100,000 in 2040 (Figure 4G). The corresponding number of incident cases in the under 5 age group is projected to decrease from 954.22 million in 2020 to 773.68 million in 2040 (Table S6). However, it was worth noting that the persons over 60 years old experienced an increasing incidence rate as age increased between 1990 and 2040 (Figure 4D–G and Table S6). The incidence rates for the 60–64, 65–69, 70–74, 75–79, and 80+ age groups in 1990 were 85,343.32, 102,463.60, 121,465.09, 126,672.77, and 141,099.09 per 100,000, respectively, with corresponding case numbers of 137.10 million, 126.53 million, 102.65 million, 77.66 million, and 78.63 (Figure 4D and Table S6). In 2040, the incidence rates for these age groups were projected to be 88,020.70, 120,336.81, 144,381.38, 162,032.30, and 178,134.97 per 100,000, respectively, with the corresponding case numbers expected to be 394.71 million, 482.92 million, 498.18 million, 421.35 million, and 584.99 million (Figure 4G and Table S6).

### 3.3. Feature Analysis

**Figure 5:**
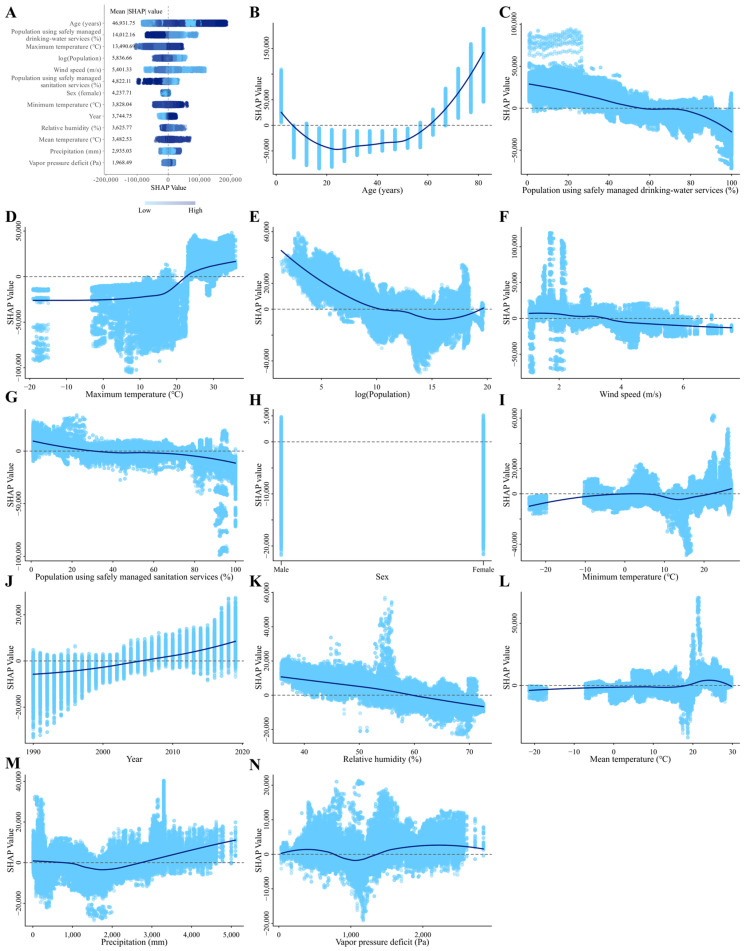
SHAP summary plot of feature contributions ranked by mean |SHAP| values and SHAP dependence plots for each feature in the XGBoost model predicting diarrheal incidence rate. (**A**) summary plot. Different colored dots stated the scaled feature values of all instances, with navy blue dots demonstrating high feature values and light sky blue dots expressing low feature values. (**B**–**N**) the dependence plot of the contribution to the model for age (**B**), gender (**C**), year (**D**), proportion of population using safely managed drinking water services (**E**), proportion of population using safely managed sanitation services (**F**), population (**G**), relative humidity (**H**), precipitation (**I**), mean temperature (**J**), minimum temperature (**K**), maximum temperature (**L**), vapor pressure deficit (**M**), and wind speed (**N**). Abbreviations: SHAP, SHapley Additive exPlanations.

SHAP values quantify the impact of each feature on individual samples. As features exert both positive and negative influences on samples, determining the overall significance of specific features involves computing the absolute average of their SHAP values across all samples. A SHAP summary plot was created to illustrate 13 features along with their mean |SHAP| value, which ranked the features based on their importance in predicting responsiveness to diarrheal diseases (Figure 5A). Each data point on the figure represents the SHAP value of a specific feature for an individual instance. The vertical axis denotes the feature value, while the horizontal axis indicates the corresponding SHAP value. Different colored dots stated the scaled feature values of all instances, with navy blue dots demonstrating high feature values and light sky blue dots expressing low feature values. The SHAP value associated with the light sky blue dot is negative, signifying that a high feature value decreases diarrheal diseases compared to the average of all samples. Conversely, the SHAP value linked to the navy blue dot is positive, indicating that a high feature value enhances diarrheal diseases. Consequently, this particular feature exerts a positive effect on diarrheal diseases.

SHAP values indicated that year, precipitation (mm), and maximum temperature (°C) make positive contributions to the model, while percentage of the population using safely managed drinking water and sanitation services, log (Population), relative humidity, and wind speed (m/s) make negative contributions to the model. In addition, the dependence plot showed that over age 60 years, post 2005, precipitation greater than 3000 mm, above 20 °C of maximum and minimum temperatures, and over 1500 Pa of vapor pressure deficit were associated with a comparatively higher risk of diarrheal diseases (Figure 5B–N). At the national level, we explored the influencing factors based on SHAP values for the five countries (Chad, Mauritania, Niger, Senegal, and Solomon Islands) with the highest ASIR in 2019. The results showed that vapor pressure deficit, precipitation, and maximum temperature had a positive effect on diarrhea incidence in these five countries, while the proportions of the population using safely managed drinking water services had a negative effect on the incidence (Figures S3–S7).

## 4. Discussion

### 4.1. Global Trends in Diarrheal Disease Incidence: 1990–2040

In this study, we used GBD data to systematically report temporal trends in the incident case and rate for the diarrheal diseases at the levels of the globe, 21 GBD regions, and 204 countries or territories by gender, age, and SDI between 1990 and 2019 and to foresightedly predict the future trends at the global, regional, and national levels over the next 21 years period. Globally, there were over 6.58 billion incident cases in 2019, with an ASIR of 86.06 thousand per 100,000. It is projected that by 2040, incidence cases will grow to more than 7.5 billion, while ASIRs will decline to 83.35 thousand per 100,000 population, following the same trend for males and females.

### 4.2. The Role of SHAP Analysis in Identifying Key Drivers of Diarrhea Incidence 

Our results indicated that in 2019, the five countries with the highest ASIR of diarrhea were Chad, Mauritania, Niger, Senegal, and the Solomon Islands. The high incidence in these countries can be attributed to several key factors, as follows: (1) lack of safe drinking water: water scarcity or contamination is a primary cause of diarrhea, especially in arid regions [33]; and (2) inadequate sanitation facilities and weak public health systems: many rural and remote areas in these countries lack sufficient sanitation facilities, leading to the spread of pathogens through water or food. Additionally, weak infrastructure, insufficient healthcare coverage, and poverty further limit access to healthcare services [33]; (3) Climate and environmental factors: Extreme weather conditions such as droughts and floods affect water quality, increasing the risk of waterborne diseases [34,35]. Our SHAP analysis for these countries revealed similar patterns. For instance, low proportions of the population using safely managed drinking water services have a positive effect on diarrhea incidence in all five countries, while higher maximum temperatures contribute positively to diarrhea incidence in Chad (Figure S3), Mauritania (Figure S4), Niger (Figure S5), and Senegal (Figure S6). Furthermore, higher precipitation positively affects diarrhea incidence in the Solomon Islands (Figure S7). These findings, derived from our SHAP value analysis, highlight the significant role these factors play in driving diarrhea incidence in these countries and underscore the importance of SHAP analysis in identifying and quantifying the impact of key factors. Our SHAP analysis further demonstrated that precipitation exceeding 3000 mm, maximum and minimum temperatures above 20 °C, and a vapor pressure deficit greater than 1500 Pa were associated with a higher risk of diarrheal diseases, while wind speeds over 4 m/s and relative humidity above 60% were linked to a lower risk. Research has shown that an increase in wind speed can reduce diarrhea incidence, consistent with our findings [36]. However, other studies suggest that diarrhea can occur independently of wind speed or even increase with stronger winds [37,38,39], highlighting inconsistencies in the literature.

### 4.3. The Impact of Climate Factors and Access to Safely Managed Drinking Water and Sanitation Services on Diarrhea Incidence

Our analysis suggests that the incidence of diarrheal diseases is temperature-sensitive and likely related to climate change. Rising temperatures, in particular, exacerbate variability in meteorological factors such as humidity and precipitation, contributing to food spoilage and water contamination, thus increasing the incidence of water- and foodborne diseases, especially in vulnerable regions like Sub-Saharan Africa and South Asia [40,41,42,43]. However, it is essential to acknowledge that while there is a strong correlation between climatic factors (e.g., temperature, rainfall) and diarrhea incidence, these relationships are not purely causal and are often mediated by other factors, such as access to clean water and sanitation infrastructure. For instance, climate change may exacerbate the risk of diarrhea in regions with poor infrastructure, while areas with well-developed sanitation systems may not experience the same effects. 

Our results emphasize the critical role of infrastructure in mediating the relationship between climate and diarrhea risk. As demonstrated by our SHAP analysis, countries with poor access to safe drinking water and sanitation facilities, such as Chad and Niger, are disproportionately affected by climatic changes. For these countries, improving access to clean water and enhancing sanitation infrastructure could significantly reduce the sensitivity of diarrhea risk to climatic factors. Future research should explore how climatic variables interact with local infrastructure, healthcare access, and socioeconomic factors to better understand these complex relationships. In particular, region-specific empirical studies will be essential for providing more granular insights into the varying impacts of climate change across different contexts. This approach will allow for more tailored interventions and effective public health strategies, especially in regions most vulnerable to climate change.

### 4.4. Trends in Diarrheal Disease Incidence among Children and the Elderly

Our results indicated that the global incident cases always peak in younger age groups (below five years) from 1990 to 2040. However, the incidence rate of diarrheal diseases in children aged <5 years decreases over the same time, which is consistent with previous studies. For instance, in India, while the incidence of childhood diarrhea has dramatically dropped, it still contributes significantly to DALYs related to the disease [44]. Similarly, children under five years of age in Sub-Saharan Africa experienced a considerable decrease in incidence from 2005 to 2015 [45]. The GBD 2016 study also showed that the incidence rate for this age group decreased in most locations, although it increased in some countries [10]. 

Notably, our study revealed a steady increase in the incidence rate of diarrheal diseases among individuals over 60 years of age at the global level. This increased risk in the elderly can be attributed to multiple factors, including immunosenescence, chronic diseases, and poor nutritional status [46]. Immunosenescence, the gradual deterioration of the immune system associated with aging, reduces the body’s ability to respond effectively to infections and vaccines, thereby increasing susceptibility to gastrointestinal pathogens [47]. Additionally, chronic diseases common among older adults, such as diabetes and cardiovascular conditions, further weaken the immune system, contributing to a heightened risk of infections [48]. Poor nutritional status, often resulting from reduced appetite, malabsorption, or socioeconomic challenges, further compromises the immune system and the body’s ability to combat infections. Deficiencies in key nutrients such as zinc, vitamin D, and protein have a particularly detrimental effect on immune function, increasing susceptibility to infections [49]. Moreover, age-related changes in gut microbiota composition, known as dysbiosis, compromise mucosal immunity and further heighten the risk of diarrheal diseases [50]. Chronic conditions more common in the elderly, such as microscopic colitis, also contribute significantly to the burden of diarrhea in this population [51]. To mitigate these risks, specific interventions should focus on improving the immune function of older adults. This could include promoting regular physical activity, which has been shown to enhance immune responses and reduce inflammation [52]. Additionally, optimizing nutritional intake through supplementation of critical micronutrients like zinc and vitamin D could strengthen immune defenses [53,54]. Managing chronic diseases, promoting gut health through probiotic interventions, and providing targeted vaccination programs are also crucial in reducing the incidence and severity of diarrheal diseases in the elderly population [55].

### 4.5. The Impact of Population Aging on Future Diarrheal Disease Burden

The global population is steadily increasing from 1990 to 2040, with the most significant growth occurring in individuals over 60 years old (Figure S8). This demographic shift has contributed to an overall rise in diarrheal disease incident cases. In contrast, the proportion of the population under 20 years old has slightly decreased, while the proportion of the elderly has gradually increased (Table S7). Despite the stability in the ASIR from 2020 to 2040, the absolute number of diarrhea cases is expected to rise due to the aging population. This underscores the need for preventive measures tailored to older age groups, such as improved sanitation, expanded vaccination programs, and enhanced healthcare access. 

### 4.6. Accounting for Variations in Disease Registration and the Role of Sanitation and Health Interventions in Predicting Future Diarrheal Disease Trends

When interpreting the rise in disease incidence, it is important to consider variations in disease registration systems across different countries. In less developed regions, these systems may be less accurate, resulting in coding errors or incomplete cause-of-death records. Changes in registration procedures over time may also affect the reported disease occurrences [56,57,58]. These variations must be acknowledged when comparing epidemiological data across regions. Moreover, the lack of data on health intervention policies across various countries limits the accuracy of our model’s forecasts regarding diarrheal disease incidence. Health policies, such as the introduction of rotavirus vaccines and improved access to medications like oral rehydration solutions (ORS) and antibiotics, can significantly influence diarrheal disease outcomes. For instance, countries that have implemented widespread rotavirus vaccination programs have experienced a notable reduction in the incidence of rotavirus-associated diarrhea [59]. Likewise, improved access to treatments like ORS and antibiotics has contributed to lower mortality rates among children under five [60]. Considering that health policies can improve water sanitation and hygiene conditions, to compensate for the model’s limitations, we based our approach on SDG 6, aiming for universal and sustainable water and sanitation by 2030 [26]. Under this strategy, we projected that the proportions for each country or region would increase at a constant annual growth rate from 2023 to 2030, reaching 100% by 2030 and remaining constant thereafter. Based on this condition, we repredicted the global numbers of diarrheal cases and ASIR up to 2040 using the XGBoost model. Compared to the predictions maintaining the levels of water sanitation and hygiene from 2023 to 2040 consistent with those of 2022, the model forecasts a reduction in the global number of diarrheal incident cases by 41.69% at 2040, reaching 4.59 billion, and a reduction in ASIR by 43.33%, reaching 47,232.75/100,000 (Table S8). These results suggest that if countries and regions, particularly those with poor sanitation conditions, actively improve water sanitation and hygiene, the number of diarrheal cases could decrease significantly. Additionally, incorporating more specific health interventions, such as vaccination programs and access to essential medications, could further enhance these reductions. Future models could be improved by integrating detailed data on these diverse health policies to provide more accurate predictions of diarrheal disease trends.

### 4.7. The Influence of SDI on Diarrheal Disease Burden and the Challenges of Data Accuracy

The SDI is a strong predictor of diarrheal mortality and has varied among countries between 1990 and 2019 [8,24]. Our analysis showed that the ASIRs of diarrheal diseases were negatively correlated with SDI during this period, consistent with previous findings from a global systematic analysis [45]. This correlation indicates that increases in SDI are associated with decreases in all-age years of healthy life lost due to disability (YLD) and age-standardized YLD rates, due to the reduction in diarrheal diseases, pneumonia, and other infectious diseases. This pattern reflects the epidemiological transition seen in earlier studies, where, as SDI increases, the disease burden shifts from infectious, maternal, neonatal, and nutritional diseases to noncommunicable causes [19]. While this transition underscores the positive effects of socioeconomic development, it also highlights the need for more refined measures that can capture both broad and nuanced impacts of socioeconomic changes on public health. Although SDI is closely associated with health status, it may not fully reflect rapid changes in living conditions, such as the onset or cessation of internal conflicts, sudden shifts in healthcare systems, or fluctuations in vaccination access [11,61]. These rapid changes can have a substantial impact on health outcomes but are often not captured by the SDI. Additionally, our analysis may be affected by potential systematic underreporting of disease cases, particularly in less developed regions. Such underreporting could lead to an underestimation of the actual disease burden and may skew the observed correlations between SDI and health outcomes [62,63,64]. We recommend interpreting our findings with caution, especially considering global variations in disease registration practices, where data quality and reporting consistency vary widely.

### 4.8. Forecasting Diarrheal Disease: Data Challenges and Modeling Strategies

Forecasting meteorological data poses several challenges, particularly where historical data are incomplete or inconsistent. The use of lagged data (1–3 years) in our linear regression model provided a method to incorporate recent trends into the forecast, while the moving average approach smoothed out year-to-year variability. By combining these methods through weighted predictions, we were able to address potential gaps in the data. However, while this approach minimizes error, there remains some uncertainty in the projections, particularly for countries with sparse historical records or extreme climatic variability. This could result in slight deviations in the predicted diarrheal incidence rates, particularly in regions where climate factors heavily influence disease transmission.

Sanitation and drinking water services data, while crucial for understanding disease burden, are subject to variability in measurement and reporting across countries and regions. The use of a linear regression model to extrapolate historical data from 1990 to 1999 addressed gaps in the data, but such projections inherently carry uncertainty, particularly for countries with limited monitoring systems. Furthermore, while our second strategy, based on SDG 6, projects an ideal scenario of 100% access to safely managed water and sanitation by 2030, it may be overly optimistic given the current infrastructure in many low- and middle-income countries. Although the first strategy, which uses 2022 data as the basis for projections, offers a more realistic outlook, the disparity between the two approaches reflects the broader challenges of achieving universal access to sanitation.

To address incomplete data, we employed imputation and modeling techniques, as detailed in the Methods section and Appendices S2 and S3. While these approaches are statistically robust, they rely on the assumption that past trends will continue, which may not always hold true. Future research could benefit from integrating more dynamic models that account for sudden environmental or socioeconomic shifts, as well as incorporating real-time and localized datasets, particularly in regions with sparse historical records. Additionally, including more detailed population and health system factors could enhance prediction precision at the country level. Despite these limitations, our modeling approach provides valuable insights into the future burden of diarrheal diseases and underscores the critical importance of improving access to safely managed water and sanitation services.

### 4.9. Limitations

There are a few limitations to this study. First, as described in the previous study [32], the data derived from the GBD dataset are not a result of direct surveillance but are evaluated using mathematical models for surveillance data. Nonetheless, the global-scale data are available from the GBD study, providing unprecedented access to explore the global burden of disease. Second, although we projected the incident cases on a global scale between 2020 and 2040, the quality of registry data in each country may partly impact previous and future trends of incidence rate for diarrheal diseases due to the detection and reporting rates. Additionally, further investigation regarding the etiology of diarrheal diseases in our future research is required to better illustrate the variation trend in the disease and consequently establish more appropriate prevention and control strategies. While our model validation results indicate good predictive performance and the SHAP values of variables partly reveal potential relationships between influencing factors and disease incidence rates, this merely represents correlations between variables and changes in disease incidence. When interpreting these trends, it is essential to recognize that correlation does not imply causation, and ecological data may lead to ecological fallacy.

Although the XGBoost model was employed to predict global diarrhea morbidity trends due to its high predictive accuracy and ability to handle large, complex datasets, it is essential to recognize the limitations and uncertainties inherent in this approach. Predictive models are sensitive to the quality and completeness of the input data, and in our case, registration errors, incomplete health records, and regional variability in data reporting could influence the results. Additionally, regional differences in health policies, access to healthcare, and sanitation infrastructure may lead to significant variability in outcomes that the model may not fully capture. For example, discrepancies in vaccine coverage, varying access to clean water, and differences in healthcare delivery systems across regions could cause the actual incidence of diarrhea to differ from the model’s predictions. Furthermore, while XGBoost is highly effective at identifying patterns and trends from historical data, its reliance on past data means that sudden changes in health policy, environmental conditions, or healthcare delivery are difficult to predict. These uncertainties underscore the need for caution when interpreting the forecasts and highlight the importance of continuing to refine predictive models with updated and region-specific data. Therefore, in discussing our results, we should prudently consider these limitations and potential biases.

## 5. Conclusions

In summary, the global ASIRs of diarrheal diseases exhibited a W-shaped pattern from 1990 to 2019. After it reached the lowest point in 2010, there was an increase between 2010 and 2019. However, it is predicted to slightly decrease over the next 21 years, accompanied by an increase in the number of reported cases. The geographical variations in the incident case and incidence rate were observed. From 1990 to 2019, the three regions with the highest increases in the EAPCs for ASIRs were North Africa and the Middle East, Central Sub-Saharan Africa, and Andean Latin America. However, it is expected to be the High-income Asia Pacific, Eastern Sub-Saharan Africa, and East Asia between 2020 and 2040. At the national level, Turkey, Afghanistan, and Libya experienced the highest increases in ASIRs (in EAPC) for diarrhea from 1990 to 2019. Meanwhile, Mexico, Japan, and the Republic of Korea were expected to lead in the EAPC of ASIR between 2020 and 2040. There is the highest incidence rate of diarrheal diseases in the young-aged (<5 years) and high-aged (≥60 years) people for both genders combined in all observation and projection years, with children under five years of age being the most affected by diarrheal diseases. No notable difference was found in the incidence rate for diarrheal diseases between women and men. In addition, meteorological features, such as wind speed, temperature, humidity, precipitation, and vapor pressure deficit, were influencing factors for diarrhea. Moreover, increasing awareness of the importance of diarrheal diseases, dietary and hygiene habits, and prevention, along with increasing the proportion of using safely managed sanitation water and services, are essential to reducing the disease incidence.

## Figures and Tables

**Table 1:** Incident cases and ASIRs per 100,000 persons of diarrheal diseases in 1990, 2019, 2020, and 2040 for males, females, and both genders combined by GBD regions, and EAPCs (95% UIs) of ASIRs for 1990–2040. Abbreviations: ASIRs, age-standardized incidence rates; GBD, global burden of disease; EAPCs, estimated annual percentage changes; UIs, 95% uncertainty intervals.

Region	Number × 100,000 (ASIRs per 100,000)		EAPC (95% UI)	Number × 100,000 (ASIRs per 100,000)		EAPC (95% UI)
	1990	2019	1990–2019	2020	2040	2020–2040
Global	46,049.51 (85,833.63)	65,816.83 (86,061.73)	−0.16 (−0.28, −0.05)	67,262.79 (86,161.16)	78,730.72 (83,349.25)	−0.07 (−0.14, 0.01)
Andean Latin America	311.34 (76,457.94)	650.59 (105,214.12)	1.08 (0.99, 1.16)	651.98 (102,243.70)	787.79 (93,164.18)	−0.31 (−0.41, −0.20)
Australasia	61.82 (30,908.51)	100.88 (33,454.63)	0.27 (0.18, 0.35)	102.43 (33,366.34)	129.63 (33,082.95)	−0.05 (−0.07, −0.04)
Caribbean	290.44 (86,224.07)	520.04 (110,389.48)	0.77 (0.70, 0.85)	498.33 (105,357.46)	561.57 (10,2690.05)	−0.06 (−0.12, −0.01)
Central Asia	518.76 (69,752.54)	823.34 (87,143.83)	1.05 (0.92, 1.17)	824.36 (86,636.41)	965.33 (86,903.44)	0.01 (−0.01, 0.03)
Central Europe	1223.92 (102,329.91)	886.37 (84,878.28)	−0.61 (−0.67, −0.56)	917.62 (88,310.32)	806.41 (86,796.12)	−0.09 (−0.13, −0.05)
Central Latin America	1525.59 (96,676.23)	1592.78 (66,667.20)	−1.41 (−1.50, −1.32)	1824.32 (70,943.54)	2380.60 (74,147.92)	0.15 (0.10, 0.20)
Central Sub-Saharan Africa	499.40 (92,683.24)	1433.85 (123,486.14)	1.08 (1.00, 1.16)	1421.56 (120,794.10)	2216.29 (118,582.53)	−0.07 (−0.11, −0.03)
East Asia	5594.63 (49,294.30)	7549.93 (51,250.62)	−0.10 (−0.30, 0.11)	7771.27 (51,690.12)	9615.70 (54,882.91)	0.37 (0.34, 0.40)
Eastern Europe	2125.40 (95,911.86)	2100.59 (102,804.21)	0.45 (0.34, 0.55)	2093.80 (103,064.15)	1925.23 (100,582.75)	−0.12 (−0.18, −0.05)
Eastern Sub-Saharan Africa	2349.59 (128,815.87)	4569.49 (125,169.19)	−0.25 (−0.33, −0.18)	4687.10 (123,574.19)	8600.17 (135,790.04)	0.52 (0.49, 0.56)
High-income Asia Pacific	284.77 (14,699.08)	339.33 (13,288.22)	−0.60 (−0.74, −0.45)	325.22 (12,515.23)	425.83 (14,726.71)	0.74 (0.18, 1.31)
High-income North America	1229.12 (45,072.30)	1735.83 (48,527.18)	0.32 (0.08, 0.57)	1751.29 (48,163.31)	1982.84 (48,792.68)	0.04 (−0.03, 0.12)
North Africa and the Middle East	2972.22 (77,896.63)	6316.09 (111,112.47)	1.25 (1.17, 1.34)	6610.99 (110,852.18)	8937.29 (109,711.19)	−0.04 (−0.06, −0.03)
Oceania	66.19 (117,094.96)	180.68 (152,728.21)	0.74 (0.66, 0.82)	179.89 (150,115.21)	251.29 (140,770.67)	−0.31 (−0.35, −0.28)
South Asia	18,166.46 (172,743.41)	22,321.03 (129,771.79)	−1.39 (−1.65, −1.13)	22,325.77 (126,803.24)	25,889.20 (121,319.17)	−0.15 (−0.18, −0.11)
Southeast Asia	2904.47 (68,651.48)	4320.58 (70,616.16)	−0.08 (−0.20, 0.05)	4223.32 (67,756.42)	6027.77 (70,846.70)	0.15 (0.10, 0.20)
Southern Latin America	219.62 (44,253.74)	325.77 (49,554.97)	0.36 (0.25, 0.48)	346.48 (52,163.96)	394.17 (52,268.30)	0.01 (−0.01, 0.03)
Southern Sub-Saharan Africa	532.36 (113,000.81)	771.71 (10,6561.41)	−0.24 (−0.28, −0.20)	781.38 (104,052.10)	1077.08 (105,448.05)	0.09 (0.04, 0.14)
Tropical Latin America	1390.42 (96,865.99)	1849.39 (86,128.97)	−0.39 (−0.43, −0.35)	1986.36 (91,553.99)	2213.17 (874,90.00)	−0.23 (−0.28, −0.17)
Western Europe	1328.04 (35,912.50)	1740.81 (36,345.48)	0.16 (0.11, 0.20)	1733.50 (36,073.38)	2037.70 (36,776.92)	0.12 (0.07, 0.16)
Western Sub-Saharan Africa	2454.94 (131,604.05)	5687.75 (139,732.85)	0.11 (0.04, 0.17)	5852.38 (137,852.18)	10,066.69 (136,396.13)	0.00 (−0.05, 0.05)
High SDI	3134.81 (39,253.13)	4449.84 (43,109.82)	0.38 (0.27, 0.48)	4263.94 (40,892.55)	4884.31 (40,619.42)	−0.03 (−0.05, −0.02)
High-middle SDI	7481.21 (66,216.14)	9525.91 (67,807.72)	0.01 (−0.09, 0.11)	9589.73 (67,525.18)	10,170.77 (62,828.53)	−0.45 (−0.56, −0.34)
Middle SDI	12,251.76 (75,085.66)	17,859.52 (77,220.15)	−0.09 (−0.21, 0.03)	17,843.60 (76,257.39)	21,367.44 (75,532.80)	0.11 (0.04, 0.18)
Low-middle SDI	15,573.24 (142,032.31)	19,743.17 (117,600.90)	−0.95 (−1.13, −0.77)	20,124.59 (117,767.81)	25,244.54 (112,988.00)	0.02 (−0.05, 0.10)
Low SDI	7586.34 (147,493.70)	14,194.88 (137,592.10)	−0.41 (−0.51, −0.32)	14,486.46 (136,297.78)	24,922.63 (140,288.48)	0.15 (0.13, 0.16)

**Table 2:** EAPCs (95% UIs) of ASIRs of diarrhea diseases (top five countries in ascending and descending orders, respectively) for males, females, and both genders combined at the national level in 1990, 2019, 2020, and 2040. Abbreviations: EAPCs, estimated annual percentage changes; UIs, 95% uncertainty intervals; ASIRs, age-standardized incidence rates.

Gender	EAPCs (95% UIs) of ASIRs per 100,000 for Diarrheal Diseases			
	1990–2019		2020–2040	
	Ascend	Descend	Ascend	Descend
Male	Turkey 1.64 (1.53, 1.75)	Mexico −2.36 (−2.50, −2.22)	Japan 2.02 (1.35, 2.69)	Bulgaria −4.13 (−5.19, −3.06)
	Democratic Republic of the Congo 1.52 (1.41, 1.62)	Guatemala −2.30 (−2.37, −2.23)	Mexico 1.93 (1.60, 2.26)	United Arab Emirates −0.93 (−1.82, −0.04)
	Azerbaijan 1.49 (1.34, 1.63)	Japan −1.90 (−2.24, −1.56)	Republic of Korea 1.64 (1.00, 2.27)	Gabon −0.69 (−0.73, −0.65)
	Afghanistan 1.48 (1.36, 1.60)	India −1.46 (−1.78, −1.14)	Malta 1.20 (0.83, 1.58)	Hungary −0.60 (−0.83, −0.36)
	Northern Mariana Islands 1.47 (1.39, 1.55)	El Salvador −1.38 (−1.51, −1.25)	Austria 1.12 (0.72, 1.52)	Latvia −0.59 (−1.09, −0.09)
Female	Turkey 1.68 (1.59, 1.78)	Guatemala −2.36 (−2.43, −2.29)	Mexico 1.94 (1.58, 2.32)	Bulgaria −3.93 (−4.89, −2.95)
	Afghanistan 1.68 (1.56, 1.79)	Mexico −1.88 (−2.09, −1.68)	Republic of Korea 1.90 (1.42, 2.39)	United Arab Emirates −1.18 (−2.23, −0.11)
	Libya 1.66 (1.55, 1.78)	India −1.78 (−2.12, −1.45)	Japan 1.57 (1.07, 2.07)	Hungary −1.03 (−1.37, −0.68)
	Iran (Islamic Republic of) 1.58 (1.43, 1.73)	Honduras −1.18 (−1.27, −1.08)	Austria 1.10 (0.61, 1.60)	Bangladesh −0.66 (−0.76, −0.56)
	Oman 1.53 (1.38, 1.68)	Ethiopia −1.15 (−1.23, −1.08)	Malta 0.99 (0.70, 1.27)	Latvia −0.61 (−1.01, −0.20)
Both genders combined	Turkey 1.66 (1.56, 1.76)	Guatemala −2.33 (−2.40, −2.26)	Mexico 1.93 (1.59, 2.28)	Bulgaria −4.02 (−5.03, −3.00)
	Afghanistan 1.57 (1.45, 1.68)	Mexico −2.12 (−2.29, −1.96)	Japan 1.79 (1.21, 2.38)	United Arab Emirates −1.02 (−1.95, −0.07)
	Libya 1.55 (1.43, 1.66)	India −1.62 (−1.94, −1.30)	Republic of Korea 1.78 (1.28, 2.29)	Hungary −0.81 (−1.09, −0.53)
	Iran (Islamic Republic of) 1.51 (1.37, 1.65)	Japan −1.42 (−1.68, −1.17)	Austria 1.11 (0.67, 1.55)	Gabon −0.62 (−0.65, −0.58)
	Oman 1.50 (1.37, 1.63)	Honduras −1.21 (−1.31, −1.10)	Malta 1.09 (0.76, 1.42)	Latvia −0.60 (−1.05, −0.16)

**Table 3:** ASIRs per 100,000 persons of diarrhea diseases (top five countries in ascending and descending orders, respectively) for males, females, and both genders combined at the national level in 1990, 2019, 2020, and 2040. Abbreviations: ASIRs, age-standardized incidence rates.

Gender	ASIR per 100,000 of Diarrheal Disease			
	1990	2019	2020	2040
Male	Guatemala (191,029.37)	Solomon Islands (200,143.18)	Solomon Islands (192,186.30)	Solomon Islands (182,279.79)
	Solomon Islands (167,816.50)	Chad (184,939.18)	Chad (180,427.73)	Guam (168,949.17)
	Nepal (163,098.45)	Niger (180,635.73)	Niger (173,912.10)	Papua New Guinea (166,186.84)
	Pakistan (162,519.87)	Mauritania (178,521.18)	Senegal (171,802.96)	Central African Republic (165,239.04)
	India (160,386.47)	Senegal (178,435.84)	Papua New Guinea (171,241.37)	Northern Mariana Islands (163,803.43)
Female	Nepal (194,934.35)	Chad (172,063.21)	Chad (167,186.18)	Niger (153,971.12)
	Bhutan (190,632.57)	Solomon Islands (168,025.96)	Niger (160,744.76)	Chad (150,486.16)
	India (189,589.35)	Niger (166,598.35)	Solomon Islands (158,247.09)	Solomon Islands (149,062.77)
	Pakistan (181,280.34)	Bhutan (160,338.60)	Nepal (152,776.67)	Central African Republic (145,490.04)
	Bangladesh (168,351.79)	Mauritania (156,813.06)	Senegal (150,551.41)	Senegal (145,430.73)
Both genders combined	Nepal (178,920.30)	Solomon Islands (184,367.97)	Solomon Islands (175,488.75)	Solomon Islands (165,746.22)
	Guatemala (178,383.02)	Chad (178,640.63)	Chad (173,790.15)	Niger (158,136.46)
	India (174,335.39)	Niger (173,308.38)	Niger (167,044.40)	Chad (156,172.25)
	Pakistan (171,377.87)	Mauritania (167,343.87)	Senegal (160,743.59)	Central African Republic (154,726.34)
	Bhutan (166,862.97)	Senegal (164,117.71)	Mauritania (156,323.05)	Senegal (152,935.00)

## Data Availability

The datasets generated for this study can be found in the GBD at https://vizhub.healthdata.org/gbd-results/ (accessed on 1 December 2022).

## Supplementary Materials

The following supporting information can be downloaded at: https://www.mdpi.com/article/10.3390/nu16183217/s1, Appendix S1: Data sources of potential covariates; Appendix S2: Estimation of the meteorological variables up to 2040; Appendix S3: Estimation of the proportions of population using safely managed drinking water services and safely managed sanitation services for periods 1990–1999 and 2023–2040; Appendix S4: Calculation of EAPC; Appendix S5: Estimation of Model evaluation; Figure S1: The prediction performance of the XGBoost model varies with different combination of hyperparameters; Figure S2: Cross validation of XGBoost model for predicting incidence rate of diarrheal diseases; Figure S3: SHAP summary plot of feature contributions for Chad, ranked by mean |SHAP| values, and SHAP dependence plots for each feature in the XGBoost model predicting diarrheal incidence rate; Figure S4: SHAP summary plot of feature contributions for Mauritania, ranked by mean |SHAP| values, and SHAP dependence plots for each feature in the XGBoost model predicting diarrheal incidence rate; Figure S5: SHAP summary plot of feature contributions for Niger, ranked by mean |SHAP| values, and SHAP dependence plots for each feature in the XGBoost model predicting diarrheal incidence rate; Figure S6: SHAP summary plot of feature contributions for Senegal, ranked by mean |SHAP| values, and SHAP dependence plots for each feature in the XGBoost model predicting diarrheal incidence rate; Figure S7: SHAP summary plot of feature contributions for Solomon Islands, ranked by mean |SHAP| values, and SHAP dependence plots for each feature in the XGBoost model predicting diarrheal incidence rate; Figure S8: The global population by age group for different years; Table S1: ASIRs per 100,000 for diarrheal diseases in 1990, 2019, 2020, and 2040 for males, females, and both genders combined at the national, regional, and global levels; Table S2: EAPCs of ASIRs per 100,000 for diarrheal diseases for males, females, and both genders combined at the national, regional, and global levels from 1990 to 2019 and from 2020 to 2040; Table S3: Incident cases for diarrheal diseases in 1990, 2019, 2020, and 2040 for males, females, and both genders combined at the national, regional, and global levels; Table S4: Incidence rates per 100,000 for diarrheal diseases in 1990, 2019, 2020, and 2040 for males, females, and both genders combined at the national, regional, and global levels; Table S5: Temporal trends of incident cases and incidence rates for diarrheal diseases for males, females, and both genders combined in all age groups (four age groups) between 1990 and 2040 at the global level; Table S6: Temporal trends of incident cases and incidence rates for diarrheal diseases for males, females, and both genders combined in all age groups (17 age groups) between 1990 and 2040 at the global level; Table S7: The global population composition (%) by year from 1990-2040; Table S8: The global diarrheal incident cases and ASIR predicted by the XGBoost model, based on the adjustment of the proportions of population using safely managed drinking water services and safely managed sanitation services according to SDG 6.
