# Supplementary material for: Global Incidence of Diarrheal Diseases—An Update Using an Interpretable Predictive Model Based on XGBoost and SHAP: A Systematic Analysis
Source: Nutrients. 2024 Sep 23;16(18):3217. doi: 10.3390/nu16183217 (PMC11434730; doi:10.3390/nu16183217)
Supplement: Supplementary file 1 [file nutrients-16-03217-s001.zip › nutrients-3193426-supplementary.pdf]

APPENDIX

TABLE OF CONTENTS

Appendix S1: Data sources of potential covariates 3

Appendix S2: Estimation of the meteorological variables up to 2040 5

Appendix S3: Estimation of the proportions of population using safely managed drinking water services and safely managed sanitation services for periods 1990−1999 and 2023−2040 6

Appendix S4: Calculation of EAPC 7

Appendix S5: Estimation of Model evaluation 8

Figure S1. The prediction performance of the XGBoost model varies with different combination of hyperparameters. 9

Figure S2. Cross validation of XGBoost model for predicting incidence rate of diarrheal diseases 11

Table S1. ASIRs per 100,000 for diarrheal diseases in 1990, 2019, 2020, and 2040 for males, females, and both genders combined at the national, regional, and global levels 12

Table S2. EAPCs of ASIRs per 100,000 for diarrheal diseases for males, females, and both genders combined at the national, regional, and global levels from 1990 to 2019 and from 2020 to 2040 37

Table S3. Incident cases for diarrheal diseases in 1990, 2019, 2020, and 2040 for males, females, and both genders combined at the national, regional, and global levels 54

Table S4. Incidence rates per 100,000 for diarrheal diseases in 1990, 2019, 2020, and 2040 for males, females, and both genders combined at the national, regional, and global levels 72

Table S5. Temporal trends of incident cases and incidence rates for diarrheal diseases for males, females, and both genders combined in all age groups (four age groups) between 1990 and 2040 at the global level 101

Table S6. Temporal trends of incident cases and incidence rates for diarrheal diseases for males, females, and both genders combined in all age groups (17 age groups) between 1990 and 2040 at the global level 107

Figure S3. SHAP summary plot of feature contributions for Chad, ranked by mean |SHAP| values, and SHAP dependence plots for each feature in the XGBoost model predicting diarrheal incidence rate 145

Figure S4. SHAP summary plot of feature contributions for Mauritania, ranked by mean |SHAP| values, and SHAP dependence plots for each feature in the XGBoost model predicting diarrheal incidence rate 146

Figure S5. SHAP summary plot of feature contributions for Niger, ranked by mean |SHAP| values, and SHAP dependence plots for each feature in the XGBoost model predicting diarrheal incidence rate 147

Figure S6. SHAP summary plot of feature contributions for Senegal, ranked by mean |SHAP| values, and SHAP dependence plots for each feature in the XGBoost model predicting diarrheal incidence rate 148

Figure S7. SHAP summary plot of feature contributions for Solomon Islands, ranked by mean |SHAP| values, and SHAP dependence plots for each feature in the XGBoost model predicting diarrheal incidence rate 149

Figure S8. The global population by age group for different years 150

Table S7. The global population composition (%) by year from 1990–2040 151

Table S8. The global diarrheal incident cases and ASIR predicted by the XGBoost model, based on the adjustment of the proportions of population using safely managed drinking water services and safely managed sanitation services according to SDG 6 154

Appendix S1: Data sources of potential covariates

Data sources of potential covariates[a]

Source	Data type	Data period	Temporal resolution	Spatial resolution
CHELSA[b]	Minimum temperature (℃)[c]	1990−2019	Monthly	1 * 1 km^2
CHELSA[b]	Maximum temperature (℃)[d]	1990−2019	Monthly	1 * 1 km^2
CHELSA[b]	Mean temperature (℃)[e]	1990−2019	Monthly	1 * 1 km^2
CHELSA[b]	Wind speed (m/s)[f]	1990-2018	Monthly	1 * 1 km^2
CHELSA[b]	Relative humidity (%)[g]	1990−2018	Monthly	1 * 1 km^2
CHELSA[b]	Vapor pressure deficit (Pa)[h]	1990−2018	Monthly	1 * 1 km^2
WorldClim[i]	Precipitation (mm)[j]	1990−2021	Monthly	5 * 5 km^2
WHO[k]	Proportion of population using safely managed drinking water services (%)[l]	2000−2022	Yearly	National or regional level
WHO[k]	Proportion of population using safely managed sanitation services (%)[m]	2000−2022	Yearly	National or regional level

[a]Data accessed in April 2024.

[b]Climatologies at high resolution for the earth's land surface areas (CHELSA), available at: https://chelsa-climate.org/.

[c]Mean daily minimum air temperature for each month (°C), [d]mean daily maximum air temperature for each month (°C), [e]mean daily air temperature for each month (°C), [f]mean monthly near-surface wind speed (m/s), [g]mean monthly near-surface relative humidity (%), and [h]mean monthly vapor pressure deficit (Pa), available at https://envicloud.wsl.ch/#/?prefix=chelsa%2Fchelsa_V2%2FGLOBAL%2F.

[i]Available at: https://worldclim.org/.

[j]Total monthly precipitation (mm), available at https://worldclim.org/data/monthlywth.html.

[k]Available at: https://www.who.int/.

[l]Proportion of population using safely managed drinking water services (%), available at https://www.who.int/data/gho/data/indicators/indicator-details/GHO/population-using-safely-managed-drinking-water-services-(-).

[m]Proportion of population using safely managed sanitation services (%), available at: https://www.who.int/data/gho/data/indicators/indicator-details/GHO/population-using-safely-managed-sanitation-services-(-).

The yearly data for minimum temperature, maximum temperature, mean temperature, wind speed, relative humidity, vapor pressure deficit, and precipitation for each nation or region was derived by aggregating the corresponding monthly data.

Appendix S2: Estimation of the meteorological variables up to 2040.

To progressively forecast the meteorological data for each country or region up to 2040, we used a weighted combination of predictions from a linear regression model and a moving average approach. The specific methodology was as follows:

We used data lagged 1 to 3 years, as well as the calendar year as inputs, and the current year's data (lag 0) as output to construct the linear regression model:

$$y_{t,linear} = \beta_{0,linear} + \beta_1 * y_{t-1} + \beta_2 * y_{t-2} + \beta_3 * y_{t-3}$$

t was the calendar year. $y_{t,linear}$ was the prediction at year t in the linear model. $\beta_{0,linear}$ was the intercept in linear model. $\beta_i, i = 1, 2, 3$ were the coefficients of the lagged variables from years $t-1$ to $t-3$. y_t was the observed data of year t.

Simultaneously, we used a simple moving average to predict the annual meteorological data:

$$y_{t,ma} = \frac{y_{t-1} + y_{t-2} + y_{t-3}}{3}$$

$y_{t,ma}$ was the prediction at year t for moving average.

The final prediction $y_{t,pred}$ was given by:

$$y_{t,pred} = w_{linear} * y_{t,linear} + w_{ma} * y_{t,ma}$$

w_{linear}, w_{ma} were the weights of the linear model and moving average predictions, respectively.

The corresponding weights were obtained through 5-fold cross-validation. These weights were inversely proportional to the prediction errors of the respective models and satisfied the condition that: $w_{linear} + w_{ma} = 1$.

Appendix S3: Estimation of the proportions of population using safely managed drinking water services and safely managed sanitation services for periods 1990−1999 and 2023−2040

For a specific nation or region, we developed a linear regression model using historical data from 2000 to 2022 to analyze the proportions of the population using safely managed drinking water services (%) and safely managed sanitation services (%). The model was constructed for the natural logarithm of these proportions with respect to the years. Subsequently, we extrapolated the data for each nation or region for the periods 1990−1999. The model's formula is expressed as follows:

$$\log(y) = \beta * year + \mathcal{E}$$

Where y represents the proportions of population using safely managed drinking water services or safely managed sanitation services for a specific nation or region. β denotes the regression coefficient for the year, and $\mathcal{E}$ represents the model's error.

We employed two strategies to fill in the proportions of the population using safely managed drinking water services (%) and safely managed sanitation services (%) from 2023 to 2040. The first strategy was to directly use the data from 2022 for projection. The second strategy was based on SDG 6, aiming to ensure availability and sustainable management of water and sanitation for all globally by 2030 (https://www.who.int/data/gho/data/themes/topics/sdg-target-6-ensure-availability-and-sustainable-management-of-water-and-sanitation-for-all). Under the second approach, the proportions for each country or region were projected to increase at a constant annual growth rate from 2023 to 2030, reaching 100% by 2030 and remaining constant thereafter. For example, if the proportion in 2022 was a, and the annual growth rate was $b\%$, the projected proportions for 2023 to 2030 would be calculated as $a \times (1 + b\%)$ for 2023, $a \times (1 + b\%)^2$ for 2024, and so on, until $a \times (1 + b\%)^8$ in 2030, when it reaches 100%.

Considering that the second strategy, while ideal, may present some challenges, we primarily used the first strategy to estimate health conditions and predict global diarrheal incidence. However, while the article primarily focuses on predictions using the first strategy, we also present supplementary disease burden projections based on the second strategy to provide a more comprehensive view.

Appendix S4: Calculation of EAPC

EAPCs were estimated based on a regression:

$$y_1 = \beta_0 + \beta_1 * x_1 \qquad (1)$$

Where x_1 represents the calendar year, y_1 is ln (ASIR), β_0 is the intercept, and β_1 is the coefficient of the calendar year. Therefore, the EAPCs measured the trends of ASIRs during a time interval (past trends: 1990-2019; future trends: 2020−2030) and were calculated as:

$$EAPC = 100 * (\exp(\beta_1) - 1) \qquad (2)$$

Appendix S5: Estimation of Model evaluation

Root mean square error (*RMSE*) and Mean absolute percentage error (*MAPE*) are defined as follows:

$$RMSE = \sqrt{\frac{1}{n}\sum_{i=1}^{n}(\hat{y}_i - y_i)^2}$$

$$MAPE = \frac{1}{n}\sum_{1}^{n}\left|\frac{\hat{y}_i - y_i}{y_i}\right|$$

n is the number of incidence rates of diarrhea. y_i and $\hat{y}_i$ ($i = 1, 2, \ldots, n$) are the observed and predicted incidence rates, respectively.

Figure S1. The prediction performance of the XGBoost model varies with different combination of hyperparameters.

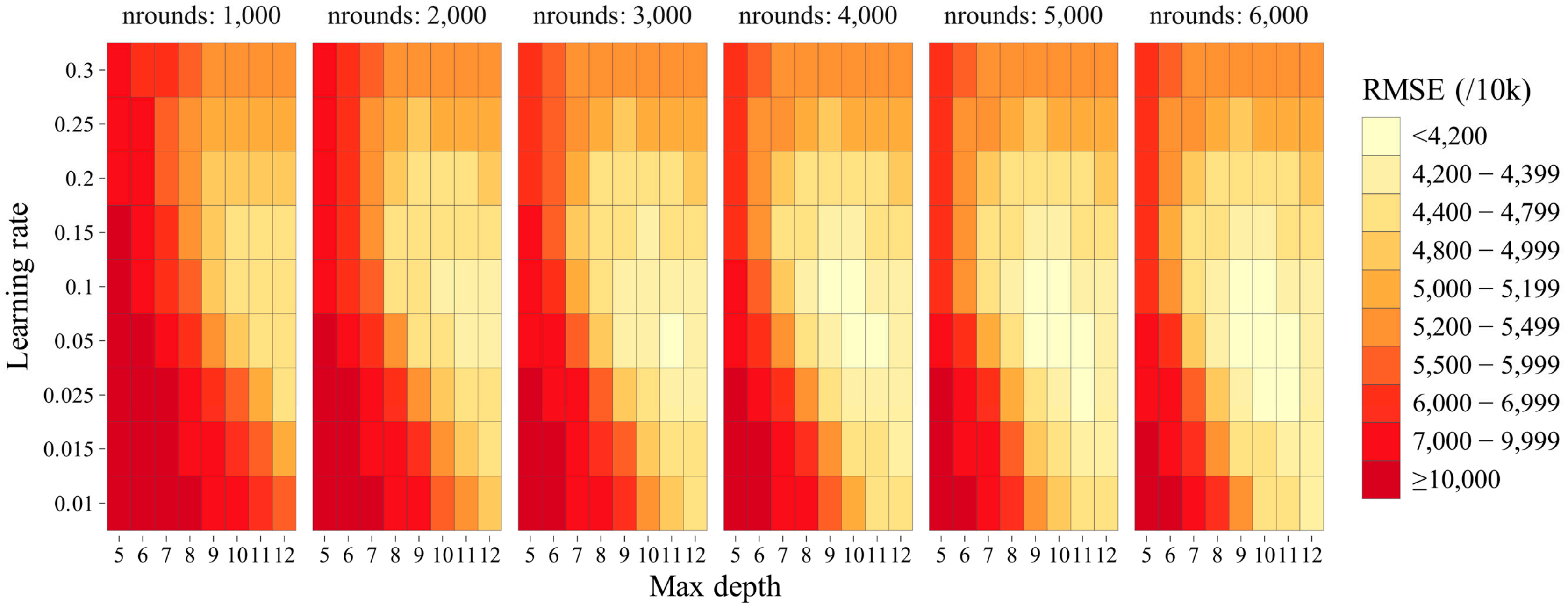

To achieve better model performance and more accurate predictions, we used grid search to tune and optimize hyperparameters such as *eta*, *max_depth*, and *nrounds*. We applied 5-fold cross-validation and calculated RMSE to evaluate each combination of hyperparameters.

max_depth: This parameter controls the maximum depth of the decision trees in the model. A higher *max_depth* allows the model to capture more complex patterns by increasing the number of splits in the trees. However, this also increases the risk of overfitting, where the model performs well on the training data but poorly on unseen data. On the other hand, a lower *max_depth* results in simpler trees that are less likely to overfit, potentially leading to better generalization on new data. However, this simplicity may come at the cost of missing subtle patterns or relationships within the data.

eta (learning rate): The *eta* parameter controls the size of the steps taken during each iteration as the model works to minimize the loss function. A smaller *eta* value leads to more conservative updates and usually requires more iterations to converge, but it can result in a more robust model by reducing the risk of

overshooting the optimal solution. On the other hand, a larger *eta* can speed up the learning process but may cause the model to converge prematurely or oscillate around suboptimal solutions.

nrounds refers to the number of iterations or number of trees to be built during the training process. Specifically, *nrounds* determines how many boosting iterations the model will go through, which corresponds to the number of decision trees being added to the model. In each iteration, XGBoost adds a new tree to improve the current model's predictions. The value of *nrounds* influences the model's complexity. If *nrounds* is too low, the model may underfit the data, while if *nrounds* is too high, the model may overfit.

In our study, *eta* was set to values of 0.01, 0.015, 0.025, 0.05, 0.1, 0.15, 0.2, 0.25, and 0.3, while *max_depth* was set to values ranging from 5 to 12. The number of boosting rounds (*nrounds*) was set from 1,000 to 6,000 in increments of 1,000.

Figure S2. Cross validation of XGBoost model for predicting incidence rate of diarrheal diseases

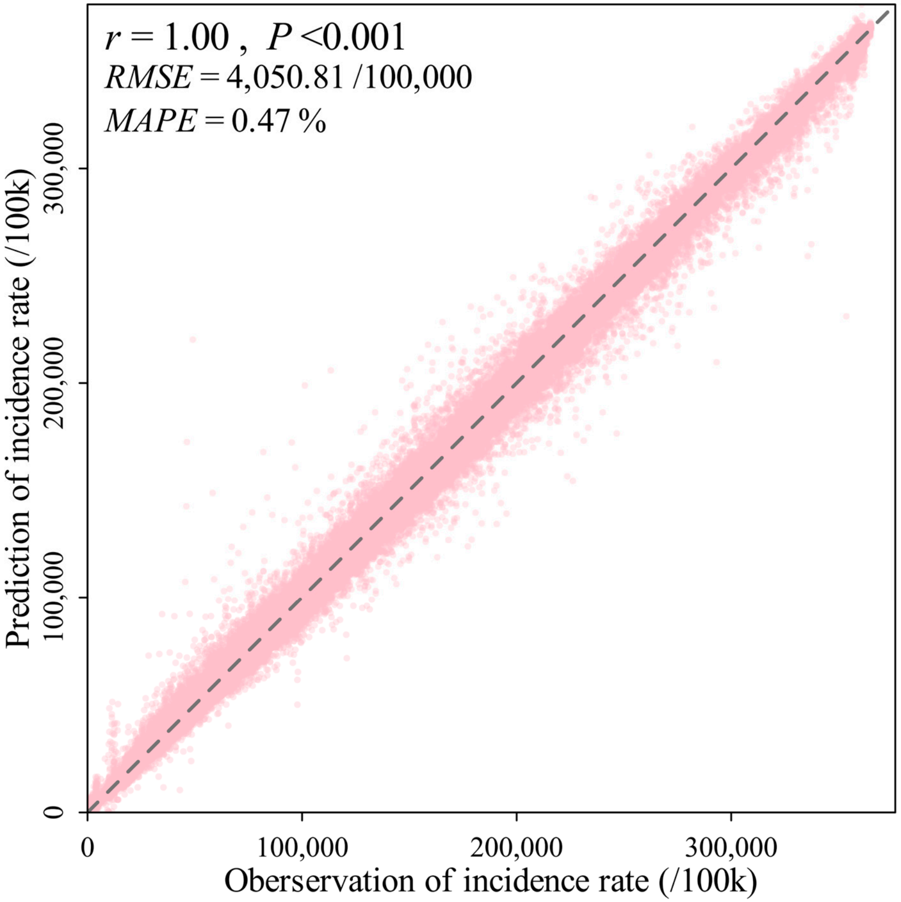

5-fold cross validation was used to assess the performance of the XGBoost model, with metrics including *RMSE* (root mean square error), *MAPE* (mean absolute percentage error), and correlation coefficient between observed and predicted values.

Table S1. ASIRs per 100,000 for diarrheal diseases in 1990, 2019, 2020, and 2040 for males, females, and both genders combined at the national, regional, and global levels

Location	ASIRs per 100,000											
	1990			2019			2020			2040		
	Male	Female	Both genders combined	Male	Female	Both genders combined	Male	Female	Both genders combined	Male	Female	Both genders combined
**Nation**												
Afghanistan	84192.23 (84112.96, 84271.59)	78559.40 (78483.05, 78635.84)	81381.65 (81326.82, 81436.53)	124829.53 (124763.80, 124895.30)	121308.38 (121245.62, 121371.19)	122989.90 (122944.63, 123035.19)	125212.15 (120519.69, 129910.20)	119062.87 (114581.76, 123582.16)	122155.22 (117936.52, 126345.20)	125189.54 (119863.71, 131273.26)	123518.95 (118457.47, 129514.05)	124366.72 (119284.96, 129955.44)
Albania	109172.52 (109013.48, 109331.91)	134414.12 (134233.23, 134595.24)	121361.53 (121242.03, 121481.16)	95787.14 (95608.23, 95966.32)	117269.65 (117070.10, 117469.50)	106475.57 (106341.80, 106609.47)	94427.42 (90752.71, 98061.09)	114841.67 (109375.41, 119147.30)	104524.91 (100289.57, 108185.31)	90611.52 (85948.35, 95409.10)	104326.64 (98536.72, 110269.69)	97274.70 (92189.12, 102182.57)
Algeria	74379.84 (74326.83, 74432.91)	76769.92 (76715.10, 76824.80)	75559.32 (75521.23, 75597.44)	100596.39 (100551.68, 100641.12)	102144.46 (102098.54, 102190.40)	101362.09 (101330.06, 101394.14)	101919.52 (100247.39, 104083.44)	103406.84 (101804.08, 105013.23)	102648.30 (101205.38, 104316.37)	103424.47 (101239.29, 106510.63)	104580.72 (102364.39, 107238.23)	103976.84 (101894.39, 106445.26)
American Samoa	101082.08 (99442.24, 102759.61)	82155.22 (80666.58, 83676.54)	92027.33 (90917.45, 93154.54)	148147.93 (146645.57, 149665.40)	113297.83 (111992.60, 114616.04)	130697.73 (129703.74, 131698.64)	138813.03 (132037.04, 145040.74)	107312.97 (102432.18, 111381.70)	123057.67 (117475.98, 127713.23)	139500.49 (133586.33, 145976.95)	106728.50 (102156.67, 110500.47)	123173.89 (118591.46, 127614.53)
Andorra	37779.98 (36948.62, 38627.63)	32623.19 (31824.17, 33439.84)	35296.46 (34718.36, 35883.00)	36975.87 (36225.72, 37740.95)	31607.55 (30895.88, 32334.86)	34301.10 (33783.10, 34826.73)	37069.67 (35843.00, 38667.93)	32476.64 (31333.50, 34244.78)	34755.56 (33713.38, 36417.33)	37648.80 (36221.42, 39205.68)	32988.16 (31650.69, 34830.65)	35336.53 (34093.75, 36852.78)
Angola	113060.13 (112927.09, 113193.44)	110960.60 (110835.75, 111085.66)	112066.70 (111975.81, 112157.72)	144259.39 (144170.60, 144348.28)	117290.30 (117218.26, 117362.39)	129944.95 (129888.70, 130001.24)	127157.88 (122381.46, 131622.14)	109380.03 (105427.41, 113055.35)	117747.45 (113756.64, 121522.75)	110128.11 (104599.49, 115606.97)	99052.35 (94272.86, 103824.50)	104251.64 (99133.01, 109360.21)

Antigua and Barbuda	80766.15 (79687.05, 81857.75)	68433.83 (67513.49, 69365.22)	74117.25 (73417.99, 74822.30)	106991.17 (105972.32, 108018.68)	97095.71 (96140.47, 98059.61)	101989.17 (101291.82, 102690.84)	104933.52 (101759.41, 107761.11)	94922.74 (92057.98, 96661.47)	99842.59 (96969.17, 101947.07)	99138.34 (89308.97, 108298.21)	88489.52 (79781.70, 95901.91)	93797.02 (84608.47, 101747.45)
Argentina	47817.53 (47783.60, 47851.48)	36973.21 (36944.18, 37002.26)	42203.98 (42181.83, 42226.14)	57336.57 (57304.32, 57368.83)	40776.51 (40749.84, 40803.18)	48860.00 (48839.18, 48880.82)	69612.39 (65537.36, 72834.38)	57810.49 (53355.64, 61196.37)	63583.74 (59491.50, 66793.71)	62082.62 (56410.83, 65996.44)	49523.11 (43330.71, 53863.93)	55728.52 (49933.14, 59575.35)
Armenia	79718.35 (79580.03, 79856.95)	84859.12 (84721.28, 84997.16)	82223.51 (82126.44, 82320.69)	104866.60 (104693.27, 105040.17)	112423.29 (112241.24, 112605.59)	108585.38 (108460.08, 108710.80)	104090.37 (101644.96, 106774.04)	112084.46 (109673.20, 114972.99)	108029.65 (105712.01, 110681.65)	105791.10 (103024.33, 109376.68)	112617.75 (109711.23, 116016.56)	108969.93 (106427.11, 112211.99)
Australia	30247.62 (30209.39, 30285.89)	26440.46 (26405.10, 26475.87)	28320.92 (28294.96, 28346.91)	34684.76 (34649.96, 34719.59)	27795.12 (27763.96, 27826.30)	31225.36 (31201.99, 31248.74)	35151.93 (33218.97, 37616.05)	28556.43 (26802.69, 31086.48)	31837.97 (30059.39, 34191.96)	41289.62 (36316.07, 46339.02)	35326.23 (29847.28, 40597.77)	38309.16 (33067.32, 43316.97)
Austria	38879.94 (38810.44, 38949.55)	34654.90 (34590.57, 34719.34)	36822.20 (36775.01, 36869.45)	38352.43 (38285.38, 38419.57)	34431.00 (34366.99, 34495.11)	36425.86 (36379.49, 36472.27)	36905.31 (35291.73, 38234.70)	34002.51 (32537.73, 35369.02)	35480.57 (34023.92, 36629.53)	50051.89 (44246.20, 56494.50)	47660.52 (41930.02, 54288.54)	48866.57 (43390.84, 55190.41)
Azerbaijan	71908.06 (71817.85, 71998.43)	76111.67 (76022.88, 76200.56)	74011.61 (73948.83, 74074.44)	99979.05 (99889.44, 100068.75)	105877.91 (105785.11, 105970.79)	102870.35 (102806.11, 102934.63)	99388.44 (95995.10, 102271.39)	109198.30 (105863.80, 112896.18)	104239.75 (101319.76, 106863.93)	96011.97 (92475.22, 99496.87)	101244.47 (96641.73, 105456.18)	98587.37 (95254.91, 102124.68)
Bahrain	71207.60 (70747.61, 71673.86)	70396.31 (69945.04, 70852.49)	70792.71 (70474.32, 71113.82)	106145.58 (105827.65, 106464.84)	105219.70 (104886.46, 105554.21)	105728.56 (105500.33, 105957.42)	101600.29 (98322.28, 104879.62)	101621.84 (98636.66, 104782.76)	101582.32 (98690.22, 104429.99)	102731.91 (99162.88, 105544.08)	102143.77 (97853.95, 104732.29)	102525.45 (98896.18, 105089.19)
Bangladesh	141957.16 (141920.67, 141993.66)	168351.79 (168309.45, 168394.15)	154638.37 (154610.65, 154666.10)	128764.97 (128739.33, 128790.61)	140700.42 (140673.45, 140727.39)	134744.33 (134725.74, 134762.92)	125404.88 (118029.73, 131319.22)	135553.55 (125269.23, 144133.98)	130500.96 (122031.43, 137162.20)	113284.37 (104815.38, 121158.65)	116586.30 (105847.40, 125975.99)	114991.85 (106101.05, 122829.81)
Barbados	81076.12 (80559.92, 81595.08)	68185.55 (67733.45, 68640.15)	74018.29 (73679.11, 74358.76)	114356.71 (113775.32, 114940.91)	106106.52 (105553.07, 106662.80)	110216.63 (109815.71, 110618.96)	109231.69 (103359.99, 113446.09)	99515.55 (93533.47, 104151.01)	104286.82 (98677.89, 108652.34)	109749.81 (105632.86, 113406.15)	101606.60 (96819.14, 105252.43)	105682.01 (101392.63, 109176.62)

Belarus	80918.64 (80837.82, 80999.54)	87967.25 (87885.70, 88048.87)	84601.54 (84544.35, 84658.76)	83324.42 (83233.34, 83415.60)	95769.72 (95673.31, 95866.21)	89730.39 (89664.27, 89796.55)	90895.47 (86189.35, 96406.76)	102160.26 (98190.93, 107427.80)	96676.13 (92416.17, 101676.73)	91921.42 (88330.85, 95932.18)	101457.79 (98068.36, 105074.35)	96678.54 (93429.76, 100105.36)
Belgium	47058.13 (46991.73, 47124.60)	42121.03 (42059.12, 42183.03)	44671.13 (44625.83, 44716.46)	49956.08 (49892.71, 50019.53)	44681.73 (44622.04, 44741.49)	47331.78 (47288.27, 47375.32)	50277.57 (48564.22, 52088.51)	45734.50 (43811.45, 47537.32)	48029.23 (46374.44, 49544.83)	47000.68 (45366.95, 48750.84)	43191.20 (41677.32, 44864.12)	45119.22 (43666.48, 46645.77)
Belize	83102.61 (82412.78, 83797.99)	69816.51 (69191.17, 70446.93)	76491.86 (76026.54, 76959.78)	103286.75 (102808.57, 103767.03)	90983.38 (90528.35, 91440.57)	97106.28 (96776.29, 97437.30)	110362.19 (105648.97, 114637.45)	94153.01 (90111.93, 98063.40)	102192.57 (97923.35, 106112.82)	108660.66 (104201.25, 113034.13)	92663.89 (88871.34, 96124.65)	100496.10 (96427.85, 104097.02)
Benin	119394.50 (119216.10, 119573.19)	115198.30 (115033.34, 115363.51)	117047.84 (116926.88, 117168.93)	140344.41 (140218.20, 140470.76)	127792.70 (127681.51, 127903.99)	133808.46 (133724.95, 133892.04)	136822.64 (133706.53, 139800.02)	124472.62 (121776.26, 126922.22)	130331.29 (127900.02, 132914.27)	130447.13 (125858.44, 135256.87)	117837.28 (112359.19, 122834.29)	123719.92 (118920.04, 128114.38)
Bermuda	80664.95 (79561.41, 81781.70)	63748.75 (62812.39, 64696.90)	71459.79 (70747.12, 72178.43)	108597.11 (107326.13, 109883.02)	95826.83 (94640.52, 97028.21)	102028.20 (101160.02, 102903.87)	105200.01 (99984.24, 108381.25)	93392.18 (87720.23, 96439.51)	99055.03 (93735.83, 101894.45)	106947.86 (99513.13, 112814.25)	94041.04 (87046.39, 99013.92)	100340.14 (93258.11, 105366.86)
Bhutan	145390.54 (144828.61, 145956.51)	190632.57 (190040.28, 191227.31)	166862.97 (166461.19, 167266.24)	157514.65 (157099.60, 157930.77)	160338.60 (159907.34, 160770.97)	158862.43 (158563.45, 159161.96)	140773.53 (135288.89, 145388.12)	148852.37 (143377.18, 154153.46)	144508.54 (139531.11, 149191.49)	135759.69 (130562.25, 140265.63)	143398.17 (137940.07, 148448.81)	139383.97 (134454.26, 143605.40)
Bosnia and Herzegovina	123705.65 (123558.15, 123853.33)	137310.18 (137155.52, 137464.99)	130580.36 (130473.66, 130687.14)	96958.83 (96783.43, 97134.53)	106987.10 (106805.24, 107169.24)	102035.32 (101908.99, 102161.80)	95109.77 (92000.50, 98089.79)	107059.84 (103293.58, 110303.36)	101067.08 (97928.78, 103875.00)	90880.69 (87098.07, 94497.47)	102100.06 (97809.60, 106767.26)	96435.11 (92714.90, 100215.06)
Botswana	154548.29 (154138.76, 154959.66)	121834.47 (121516.24, 122153.73)	137062.33 (136809.33, 137315.98)	149281.35 (149009.02, 149554.46)	124206.93 (123986.41, 124427.88)	136136.45 (135965.68, 136307.49)	145305.44 (141506.99, 148693.79)	119287.57 (116253.47, 122286.75)	131645.10 (128803.04, 134375.88)	131411.46 (127127.55, 135302.27)	114907.79 (111676.79, 117878.87)	123145.14 (119803.45, 126138.05)
Brazil	100097.33 (100071.52, 100123.14)	95108.75 (95084.92, 95132.59)	97459.47 (97441.98, 97476.95)	85033.62 (85015.33, 85051.92)	87167.55 (87149.44, 87185.66)	86054.96 (86042.13, 86067.80)	93432.54 (90089.92, 97543.41)	94994.92 (91282.06, 100172.99)	94186.52 (90846.18, 98698.07)	89482.74 (84258.50, 94706.57)	90118.67 (84266.28, 95394.62)	89804.17 (84571.84, 94995.49)

Bulgaria	104060.22 (103957.58, 104162.94)	112715.74 (112609.62, 112821.94)	108501.83 (108427.99, 108575.71)	85169.04 (85052.38, 85285.85)	95645.63 (95522.36, 95769.04)	90363.33 (90278.57, 90448.16)	92563.88 (87452.84, 99016.86)	103406.98 (97801.25, 109959.61)	97973.24 (92667.26, 104086.54)	45769.07 (39681.95, 56766.30)	51935.61 (45201.48, 61568.37)	48834.67 (42416.13, 58589.93)
Burkina Faso	129250.83 (129118.62, 129383.23)	137514.75 (137388.78, 137640.88)	133589.62 (133498.48, 133680.84)	160508.55 (160409.37, 160607.81)	149389.71 (149303.00, 149476.48)	154399.11 (154333.85, 154464.42)	158083.46 (154753.78, 160444.84)	145748.57 (142835.91, 147598.24)	151293.77 (148410.96, 153153.60)	152792.22 (149248.67, 156157.12)	142567.49 (139359.38, 145610.35)	147161.14 (144097.18, 150085.37)
Burundi	129679.89 (129505.60, 129854.50)	130350.18 (130190.09, 130510.51)	129985.37 (129867.67, 130103.20)	153539.64 (153410.57, 153668.88)	132602.22 (132481.16, 132723.41)	143393.53 (143305.00, 143482.15)	151568.58 (147677.09, 154638.42)	129800.32 (126387.23, 132295.18)	140927.71 (137770.17, 143253.59)	143842.36 (140058.75, 147516.43)	124547.06 (121158.52, 127747.93)	133972.66 (130763.68, 137160.42)
Cambodia	87201.76 (87088.59, 87315.13)	78523.17 (78431.01, 78615.46)	82283.63 (82212.27, 82355.08)	82192.89 (82118.69, 82267.17)	70088.88 (70028.37, 70149.43)	75598.71 (75552.01, 75645.44)	83560.48 (80307.64, 87278.28)	70832.21 (68015.78, 74460.09)	76610.52 (74015.08, 80149.62)	86864.41 (82249.66, 92382.70)	76030.78 (71833.57, 81517.65)	81262.78 (76991.88, 86380.66)
Cameroon	127704.22 (127579.98, 127828.61)	125347.69 (125229.38, 125466.15)	126471.98 (126386.31, 126557.72)	153804.64 (153721.83, 153887.52)	138958.60 (138883.68, 139033.56)	146290.31 (146234.69, 146345.95)	145121.41 (140244.43, 149227.14)	129771.35 (125415.88, 133488.53)	137317.77 (132839.46, 141015.96)	128220.33 (121710.95, 134978.54)	115467.32 (109153.81, 121941.09)	121598.48 (115492.96, 127847.48)
Canada	56237.56 (56195.95, 56279.20)	37302.03 (37268.77, 37335.31)	46844.93 (46818.25, 46871.63)	70060.75 (70018.07, 70103.45)	44119.33 (44085.97, 44152.71)	57130.44 (57103.28, 57157.62)	69229.90 (67694.01, 70442.86)	46093.49 (44842.71, 47659.72)	57712.99 (56641.52, 58709.38)	68512.64 (66752.09, 69960.39)	46370.43 (44957.81, 47985.76)	57567.23 (56289.97, 58778.33)
Central African Republic	104567.79 (104302.12, 104834.83)	100555.58 (100335.06, 100776.86)	102459.28 (102290.89, 102628.16)	152664.04 (152441.99, 152886.76)	127821.30 (127648.54, 127994.40)	139839.52 (139703.33, 139975.93)	150768.61 (147813.50, 155543.62)	128750.86 (125848.01, 132925.26)	139739.01 (137144.44, 143896.03)	165239.04 (160627.64, 169849.86)	145490.04 (140891.91, 149932.96)	154726.34 (150676.04, 159124.24)
Chad	143062.39 (142894.67, 143230.34)	142224.63 (142068.18, 142381.27)	142491.85 (142377.66, 142606.15)	184939.18 (184816.16, 185062.30)	172063.21 (171941.53, 172185.00)	178640.63 (178554.22, 178727.09)	180427.73 (178047.92, 182672.20)	167186.18 (164469.79, 169141.39)	173790.15 (171306.53, 175785.87)	162808.89 (158486.52, 167334.30)	150486.16 (146075.87, 154699.51)	156172.25 (152372.90, 160154.51)
Chile	57562.20 (57501.62, 57622.84)	45110.58 (45059.34, 45161.87)	51133.04 (51093.71, 51172.39)	57124.17 (57072.37, 57176.00)	47697.06 (47650.01, 47744.14)	52286.85 (52251.95, 52321.76)	56209.24 (54398.62, 57997.78)	48938.03 (46805.83, 50894.27)	52513.50 (50843.49, 54179.80)	54711.02 (52255.87, 57239.58)	47994.89 (45613.03, 50928.48)	51325.65 (49131.99, 54162.75)
China	51531.43 (51524.85, 51538.02)	46557.91 (46551.93, 46563.88)	48980.54 (48976.15, 48984.92)	53269.26 (53263.51, 53275.01)	47573.64 (47567.93, 47579.36)	50395.68 (50391.64, 50399.72)	53902.60 (52388.43, 55408.77)	48902.28 (47444.29, 50531.17)	51377.62 (50061.26, 52750.61)	57544.21 (55015.48, 60934.22)	53310.17 (50454.31, 56896.54)	55334.47 (52874.35, 58426.39)

Colombia	99737.20 (99683.65, 99790.78)	86963.89 (86913.45, 87014.36)	93259.61 (93222.86, 93296.37)	78986.18 (78949.21, 79023.16)	69658.68 (69624.39, 69692.99)	74429.02 (74403.81, 74454.23)	78938.00 (76684.48, 81236.38)	68391.14 (66041.72, 70805.20)	73778.08 (71694.95, 75958.10)	82591.72 (79521.80, 86027.25)	72379.11 (69009.28, 76787.71)	77612.14 (74558.58, 81033.17)
Comoros	131732.98 (131170.74, 132298.21)	132925.47 (132381.38, 133472.16)	132346.70 (131955.74, 132739.05)	149996.40 (149556.71, 150437.42)	129995.76 (129602.81, 130389.82)	139855.48 (139561.95, 140149.61)	138484.64 (132542.01, 144141.43)	120749.61 (115015.13, 125526.81)	129489.78 (124086.93, 134317.60)	138013.64 (132277.39, 143216.24)	118836.06 (113666.32, 123310.16)	128321.43 (123115.17, 132915.64)
Costa Rica	92626.72 (92458.95, 92794.79)	80957.09 (80798.30, 81116.16)	86832.53 (86717.00, 86948.20)	78506.20 (78387.41, 78625.14)	66912.01 (66804.37, 67019.80)	72677.28 (72597.24, 72757.39)	78078.73 (74656.15, 81997.15)	66881.74 (63911.53, 69868.32)	72506.92 (69652.53, 75488.58)	79543.50 (75359.14, 84925.45)	67872.07 (64298.61, 71817.24)	73827.90 (70424.92, 77891.18)
Croatia	101198.60 (101064.43, 101332.92)	100766.51 (100634.61, 100898.56)	101073.63 (100979.71, 101167.63)	80567.50 (80424.99, 80710.24)	84029.77 (83885.37, 84174.41)	82353.32 (82251.88, 82454.88)	81684.25 (79141.04, 84443.45)	86151.45 (82989.87, 89484.12)	83946.16 (81411.31, 86687.15)	80715.68 (76907.19, 84664.50)	84156.05 (79949.32, 88693.27)	82417.33 (78749.10, 86313.18)
Cuba	89164.14 (89081.64, 89246.69)	72860.73 (72785.73, 72935.79)	81020.18 (80964.44, 81075.96)	112716.63 (112624.90, 112808.42)	91932.96 (91850.81, 92015.18)	102488.55 (102426.87, 102550.27)	109412.78 (106079.65, 112202.06)	91268.47 (88552.99, 94473.63)	100484.58 (97745.19, 103181.12)	110773.15 (107832.19, 113669.35)	94647.31 (91470.15, 98004.03)	102839.87 (100165.60, 105650.27)
Cyprus	47992.55 (47767.38, 48218.71)	43378.14 (43163.41, 43593.79)	45766.09 (45610.35, 45922.30)	45306.32 (45124.41, 45488.84)	39403.29 (39231.09, 39576.14)	42331.58 (42206.37, 42457.11)	48545.57 (45038.53, 53968.15)	41191.48 (38010.81, 46795.93)	44813.40 (41675.26, 50140.86)	46453.16 (42410.03, 51787.96)	39844.10 (36064.40, 45359.18)	43082.00 (39520.73, 48233.41)
Congo	98898.40 (98646.36, 99151.49)	91231.20 (91016.91, 91446.20)	94838.87 (94675.78, 95002.37)	141423.40 (141245.30, 141601.83)	108835.26 (108684.92, 108985.86)	125098.72 (124982.78, 125214.79)	130001.30 (125591.72, 134719.78)	103419.82 (100002.27, 106940.55)	116734.18 (113081.23, 120623.78)	117609.91 (111933.75, 123152.68)	95653.46 (91162.06, 99572.30)	106620.26 (102012.82, 111039.62)
Denmark	46423.84 (46331.13, 46516.71)	41823.07 (41735.77, 41910.53)	44193.17 (44129.56, 44256.86)	47831.51 (47744.16, 47919.00)	44441.66 (44358.13, 44525.34)	46231.81 (46171.34, 46292.35)	46150.82 (44269.17, 48271.94)	42826.44 (40820.89, 45064.91)	44557.67 (42702.07, 46626.30)	44901.96 (42808.40, 46776.78)	41488.09 (39479.45, 43280.28)	43249.16 (41376.12, 44997.75)
Djibouti	119687.40 (118985.14, 120398.73)	112435.01 (111780.94, 113095.34)	116328.84 (115852.89, 116808.43)	130262.26 (129912.52, 130613.56)	107707.99 (107372.01, 108045.37)	119763.49 (119520.73, 120006.98)	133982.10 (129318.95, 139261.23)	112652.54 (108972.50, 117371.00)	124017.75 (120023.38, 128849.02)	134020.46 (127248.65, 140630.36)	114203.84 (108563.20, 119932.88)	124538.62 (118466.20, 130607.92)
Dominica	78821.77 (77869.81, 79785.47)	66584.57 (65766.67, 67411.43)	72265.75 (71649.14, 72887.03)	101876.30 (100791.53, 102971.44)	91801.14 (90760.11, 92852.84)	96986.95 (96234.56, 97744.59)	99355.29 (97045.34, 101231.80)	89844.58 (87808.09, 91455.91)	94729.71 (92765.76, 96367.39)	97712.01 (94619.48, 100652.20)	89452.66 (86821.38, 91510.78)	93629.79 (90867.08, 95934.93)

Dominican Republic	97926.98 (97809.10, 98045.00)	83174.43 (83067.54, 83281.47)	90420.29 (90340.85, 90499.80)	118415.25 (118320.80, 118509.75)	106238.02 (106149.15, 106326.94)	112369.30 (112304.50, 112434.14)	119990.61 (117283.68, 122365.31)	105616.53 (102674.28, 108064.38)	112831.48 (110142.37, 114992.99)	116555.41 (113386.51, 119454.06)	101729.27 (98553.13, 104636.14)	109148.89 (106142.28, 111870.41)
Ecuador	81706.83 (81621.02, 81792.73)	74678.93 (74597.67, 74760.28)	78156.42 (78097.37, 78215.52)	93908.63 (93842.81, 93974.49)	94677.28 (94611.78, 94742.81)	94410.77 (94364.31, 94457.26)	97298.99 (95744.15, 99213.84)	95474.25 (93759.43, 97200.14)	96446.22 (95138.35, 98075.09)	99105.88 (96190.75, 102463.44)	95150.92 (92269.41, 97961.75)	97219.22 (94416.93, 100089.96)
Egypt	81310.67 (81274.53, 81346.83)	79853.46 (79817.49, 79889.45)	80595.68 (80570.20, 80621.17)	102354.12 (102324.02, 102384.23)	104972.72 (104938.98, 105006.47)	103577.02 (103554.67, 103599.38)	101401.93 (99764.55, 103199.95)	103602.94 (101960.31, 105215.82)	102485.82 (100889.70, 103918.43)	102556.84 (99756.30, 106261.60)	104166.84 (101423.31, 107609.81)	103354.01 (100703.94, 106818.39)
El Salvador	126327.58 (126174.91, 126480.43)	106512.03 (106376.76, 106647.45)	116097.98 (115996.49, 116199.56)	93561.20 (93448.50, 93674.02)	86407.10 (86304.75, 86509.55)	89883.27 (89807.64, 89958.94)	93573.44 (86783.82, 99619.94)	85179.44 (79473.36, 91538.99)	89207.39 (83055.93, 95022.74)	89418.45 (82871.56, 95810.18)	84729.44 (79288.17, 89878.21)	87109.67 (81607.26, 92627.86)
Equatorial Guinea	115495.98 (114868.27, 116129.29)	112751.23 (112221.12, 113284.65)	113873.00 (113469.76, 114278.29)	118425.23 (118036.79, 118815.39)	94391.84 (94086.58, 94698.26)	105581.95 (105341.56, 105823.03)	112952.62 (110739.67, 114934.81)	90616.84 (88728.96, 92246.88)	101039.10 (99410.17, 102653.02)	104138.27 (100175.84, 107847.75)	80936.91 (77690.64, 83568.93)	92330.27 (88843.12, 95084.91)
Eritrea	137553.68 (137219.89, 137889.54)	140207.38 (139953.58, 140461.96)	138892.73 (138695.35, 139090.66)	170938.01 (170736.02, 171140.49)	135421.65 (135267.69, 135575.85)	152833.13 (152710.83, 152955.58)	165088.01 (162146.46, 167774.61)	134045.55 (131246.04, 136488.51)	148826.27 (146421.16, 150902.07)	158934.40 (153964.14, 163266.78)	133951.58 (129741.15, 137760.94)	146177.77 (142076.27, 149989.61)
Estonia	90183.76 (89963.57, 90404.44)	93078.43 (92860.56, 93296.72)	91718.83 (91564.64, 91873.23)	92184.88 (91924.91, 92445.50)	118043.21 (117748.27, 118338.82)	105152.20 (104956.28, 105348.44)	90430.59 (83352.02, 94281.75)	110985.30 (100370.53, 116446.84)	100731.11 (91937.85, 105009.39)	89381.52 (82878.66, 96043.38)	107325.24 (97801.50, 116859.71)	98259.10 (90884.08, 106129.52)
Ethiopia	140473.28 (140409.11, 140537.51)	153772.21 (153707.90, 153836.57)	147190.14 (147144.98, 147235.32)	128699.53 (128660.60, 128738.47)	117017.70 (116980.03, 117055.38)	122873.22 (122846.14, 122900.32)	129183.83 (127662.83, 131331.62)	117164.59 (115539.68, 119227.53)	123156.59 (121759.08, 125028.19)	131250.62 (129112.25, 133785.69)	120195.21 (117845.50, 122828.33)	125647.74 (123645.01, 128073.62)
Fiji	118253.91 (117809.42, 118700.82)	92856.02 (92458.45, 93255.71)	105677.48 (105379.48, 105976.60)	153704.57 (153314.27, 154096.25)	114564.45 (114236.36, 114893.49)	134383.41 (134129.93, 134637.45)	148217.45 (143507.49, 152289.03)	111980.43 (108631.81, 115806.60)	130313.30 (126521.88, 133865.34)	143756.07 (138874.16, 148402.11)	109272.05 (105715.90, 113039.36)	126570.17 (122742.30, 130320.54)

Finland	40072.20 (39985.62, 40158.93)	36432.64 (36352.40, 36513.03)	38353.10 (38294.38, 38411.89)	36899.80 (36815.82, 36983.95)	32935.68 (32855.69, 33015.84)	34939.90 (34881.89, 34998.00)	38080.31 (36532.83, 39734.89)	34633.99 (33125.88, 36639.88)	36392.66 (35000.92, 38194.90)	38604.18 (36855.96, 40954.17)	35357.84 (33473.84, 38243.62)	36980.61 (35297.09, 39543.40)
France	36912.53 (36888.85, 36936.22)	31759.23 (31737.79, 31780.68)	34362.14 (34346.23, 34378.07)	35157.77 (35135.28, 35180.28)	30325.38 (30304.56, 30346.22)	32738.29 (32722.97, 32753.62)	36465.19 (35450.55, 37869.69)	31819.13 (30686.12, 33120.64)	34142.34 (33195.22, 35383.45)	36555.66 (35472.94, 37893.10)	31519.04 (30377.55, 32918.08)	34048.84 (33005.62, 35265.72)
Gabon	91854.91 (91511.89, 92199.84)	80843.06 (80563.58, 81123.50)	86220.18 (86005.65, 86435.32)	123781.96 (123500.79, 124063.99)	91197.36 (90974.99, 91420.24)	107073.77 (106897.88, 107249.97)	119650.02 (116907.86, 122347.00)	88561.84 (85868.87, 91208.92)	103609.74 (101017.21, 105853.98)	103784.18 (98670.84, 108527.45)	79843.89 (76284.56, 83331.41)	91037.25 (87185.95, 94732.76)
Germany	42459.41 (42436.35, 42482.48)	37858.26 (37836.86, 37879.66)	40221.95 (40206.28, 40237.62)	46666.34 (46642.67, 46690.02)	42099.06 (42076.35, 42121.78)	44403.27 (44386.86, 44419.68)	47623.22 (46169.73, 48989.73)	43627.94 (42201.68, 45236.53)	45644.51 (44319.25, 47025.25)	46462.49 (44791.44, 48129.20)	42322.41 (40583.16, 43991.65)	44419.43 (42764.67, 45809.62)
Ghana	126636.25 (126529.96, 126742.68)	119247.18 (119150.41, 119344.05)	122846.64 (122775.05, 122918.28)	139884.79 (139811.15, 139958.49)	124532.58 (124470.15, 124595.04)	131612.04 (131564.45, 131659.64)	131811.27 (127055.54, 135850.59)	120942.97 (116624.20, 125071.39)	125926.64 (121462.87, 129724.00)	126934.80 (121261.47, 132242.70)	116941.96 (111597.58, 122239.65)	121496.74 (116065.38, 126152.80)
Greece	40777.65 (40716.83, 40838.55)	36009.00 (35951.94, 36066.14)	38432.75 (38391.03, 38474.52)	37697.08 (37632.05, 37762.20)	33707.62 (33646.03, 33769.31)	35732.21 (35687.38, 35777.08)	38819.19 (36649.73, 41428.63)	35175.38 (33138.39, 37736.75)	37014.11 (35072.79, 39573.66)	38930.27 (36827.94, 41246.18)	34877.38 (32706.58, 37272.30)	36931.06 (34862.94, 39154.82)
Greenland	51625.85 (50754.94, 52522.55)	41737.88 (40916.32, 42577.51)	46873.78 (46276.69, 47481.09)	56542.41 (55632.66, 57465.23)	42781.41 (41968.48, 43607.25)	49949.42 (49336.30, 50568.78)	55530.06 (52662.30, 58190.21)	43937.39 (41754.67, 46940.70)	49960.28 (47741.11, 53014.27)	55237.83 (52148.07, 58324.03)	42835.76 (40385.06, 45764.85)	49213.76 (46729.91, 51988.93)
Grenada	89474.27 (88513.59, 90444.51)	75957.04 (75134.83, 76787.71)	81900.81 (81278.46, 82527.60)	120846.97 (119842.16, 121861.01)	108193.17 (107259.48, 109133.77)	114627.50 (113947.56, 115310.88)	119421.28 (116397.63, 122182.21)	106023.08 (102709.15, 108416.05)	112925.65 (109971.85, 115210.76)	115430.80 (110736.94, 119453.04)	102392.70 (98452.78, 105822.87)	109049.28 (104987.13, 112658.34)
Guam	117123.39 (116129.06, 118130.81)	93865.39 (92978.89, 94761.97)	106258.36 (105593.12, 106929.21)	173777.58 (172905.18, 174653.65)	130751.90 (129981.95, 131525.56)	153011.40 (152428.02, 153596.56)	170392.99 (166628.50, 173556.71)	128783.55 (125721.51, 131309.54)	150215.91 (146849.97, 152912.95)	168949.17 (163967.83, 173073.65)	127983.36 (123723.41, 131124.87)	148790.05 (144739.73, 151772.87)
Guatemala	191029.37 (190869.53, 191189.39)	166075.74 (165925.29, 166226.37)	178383.02 (178273.38, 178492.75)	103005.97 (102932.73, 103079.26)	87910.41 (87845.48, 87975.38)	95288.71 (95240.04, 95337.41)	101596.12 (97416.32, 105699.40)	90413.52 (86042.33, 94460.55)	95946.15 (91794.67, 99740.05)	97834.02 (93359.58, 102474.03)	86188.91 (81949.70, 91023.51)	92029.27 (87829.12, 96516.16)

Guinea	129724.27 (129575.70, 129873.02)	128441.73 (128298.34, 128585.30)	129005.31 (128902.25, 129108.45)	150921.52 (150799.51, 151043.63)	137999.00 (137886.84, 138111.26)	144075.08 (143992.56, 144157.64)	145799.81 (141557.16, 149269.84)	129916.56 (126010.32, 133079.34)	137321.35 (133621.96, 140597.32)	133244.40 (127246.29, 139079.66)	120407.96 (115085.36, 125699.03)	126001.52 (120409.95, 131019.56)
Guinea-Bissau	121829.45 (121415.19, 122245.92)	130670.13 (130272.83, 131069.14)	126415.08 (126128.90, 126702.22)	151152.52 (150801.48, 151504.90)	145521.84 (145216.65, 145827.88)	148068.59 (147838.99, 148298.71)	147838.18 (145058.04, 150443.13)	139734.93 (137426.07, 142220.91)	143414.47 (141172.38, 145713.36)	151930.23 (148885.86, 154641.41)	141756.60 (138729.44, 144502.66)	146284.35 (143424.69, 148655.63)
Guyana	93273.59 (92894.55, 93654.41)	78109.81 (77781.15, 78439.91)	85453.24 (85203.91, 85703.36)	105744.46 (105389.01, 106101.19)	93609.63 (93288.25, 93932.00)	99571.60 (99333.09, 99810.67)	108657.16 (105042.35, 112241.35)	94509.64 (91067.33, 97359.68)	101449.24 (98225.17, 104272.29)	107331.82 (103714.81, 111518.31)	91596.96 (88930.24, 95235.59)	99216.77 (96560.53, 102459.19)
Haiti	113909.30 (113764.18, 114054.71)	103633.96 (103504.98, 103763.15)	108572.52 (108476.24, 108668.91)	128483.25 (128379.40, 128587.20)	116464.20 (116368.74, 116559.75)	122192.81 (122122.51, 122263.15)	128527.17 (124919.42, 132710.36)	111201.59 (107412.95, 114612.53)	119408.51 (116085.84, 122522.39)	124756.92 (120715.60, 129078.65)	106836.58 (102785.14, 110893.26)	115503.71 (111455.23, 119218.28)
Honduras	132622.15 (132452.79, 132791.76)	120371.15 (120206.27, 120536.27)	126450.40 (126332.25, 126568.68)	96386.09 (96291.72, 96480.56)	88515.62 (88426.04, 88605.28)	92469.36 (92404.33, 92534.43)	92881.39 (88982.74, 96439.48)	84262.40 (81016.85, 87507.67)	88492.67 (85060.54, 91678.59)	92996.63 (88786.94, 97018.32)	82662.96 (79256.23, 86404.40)	87846.40 (84189.52, 91252.92)
Hungary	115631.29 (115532.32, 115730.33)	132461.77 (132357.14, 132566.47)	124308.97 (124236.99, 124380.99)	102528.15 (102423.10, 102633.29)	117412.68 (117301.51, 117523.94)	110123.31 (110046.95, 110199.71)	56392.46 (51243.27, 62957.93)	59535.03 (53100.55, 67171.33)	58007.04 (52476.86, 64753.88)	50617.94 (45261.03, 56826.52)	49210.28 (42734.25, 56226.91)	49981.07 (44113.43, 56583.24)
Iceland	44768.78 (44393.44, 45146.64)	41695.76 (41335.38, 42058.64)	43287.60 (43027.43, 43549.03)	44373.65 (44038.91, 44710.58)	40323.12 (40001.43, 40647.05)	42346.86 (42114.71, 42580.10)	44088.51 (43056.12, 46267.16)	40532.26 (39620.27, 41884.16)	42306.97 (41445.82, 44073.94)	43192.55 (41395.25, 44637.16)	39933.96 (38187.85, 41638.42)	41552.55 (39807.81, 43002.03)
India	160386.47 (160372.55, 160400.38)	189589.35 (189573.99, 189604.70)	174335.39 (174325.07, 174345.71)	120102.52 (120094.02, 120111.01)	131756.50 (131747.62, 131765.39)	125854.72 (125848.58, 125860.86)	116635.36 (111546.26, 121528.13)	127083.52 (121543.99, 131842.58)	121788.20 (116722.97, 126374.67)	101303.19 (94492.63, 108081.03)	111163.33 (103755.87, 118142.59)	106132.79 (99573.09, 112869.70)
Indonesia	76291.02 (76268.21, 76313.84)	62493.62 (62474.36, 62512.89)	69143.06 (69128.28, 69157.85)	75576.45 (75559.23, 75593.67)	65853.69 (65838.25, 65869.13)	70619.60 (70608.13, 70631.08)	72863.77 (69773.93, 76504.53)	62540.77 (59421.17, 65519.29)	67497.74 (64864.03, 70587.26)	71068.30 (66421.62, 75997.94)	59734.69 (55594.74, 64116.40)	65145.77 (61141.99, 69615.87)

Iraq	76943.76 (76879.38, 77008.21)	77022.88 (76959.55, 77086.28)	76963.31 (76918.22, 77008.44)	102779.05 (102729.15, 102828.98)	102562.58 (102513.50, 102611.69)	102698.61 (102663.66, 102733.57)	103543.73 (101292.60, 105772.43)	102755.31 (100566.04, 104947.00)	103163.46 (101098.09, 105222.95)	102993.17 (100796.74, 105065.62)	102786.02 (100686.20, 104770.03)	102891.95 (100826.95, 104802.62)
Ireland	34132.19 (34044.65, 34219.93)	30651.08 (30569.04, 30733.30)	32448.72 (32388.79, 32508.73)	33359.30 (33281.78, 33436.98)	31249.07 (31175.01, 31323.28)	32358.19 (32304.55, 32411.91)	33710.00 (31997.01, 35901.52)	32473.30 (30713.89, 34913.57)	33138.77 (31604.21, 35387.26)	35724.15 (33612.41, 38363.52)	34523.77 (32617.87, 37718.44)	35165.19 (33319.78, 37924.31)
Israel	39679.95 (39601.53, 39758.50)	35441.34 (35368.12, 35514.69)	37609.59 (37555.95, 37663.31)	43747.98 (43688.97, 43807.05)	38837.05 (38782.31, 38891.85)	41310.28 (41270.09, 41350.50)	43385.66 (40592.66, 45977.26)	37155.83 (34595.67, 39551.15)	40262.66 (37733.82, 42528.40)	44746.09 (41354.29, 48117.61)	39469.78 (37064.72, 42174.99)	42135.83 (39491.59, 45090.82)
Italy	31045.81 (31021.74, 31069.90)	27673.54 (27651.04, 27696.05)	29392.96 (29376.50, 29409.43)	28640.57 (28615.18, 28665.99)	25398.34 (25374.23, 25422.46)	27071.99 (27054.46, 27089.54)	30541.72 (27607.71, 34301.85)	30351.98 (26935.93, 34864.06)	30468.62 (27489.29, 34385.36)	33504.24 (30257.40, 37967.25)	31631.66 (28041.03, 36472.11)	32598.04 (29389.47, 37053.58)
Jamaica	77725.30 (77553.71, 77897.22)	64172.44 (64023.43, 64321.76)	70639.53 (70526.71, 70752.51)	92454.04 (92289.51, 92618.81)	81286.69 (81133.25, 81440.38)	86796.66 (86684.40, 86909.05)	88401.58 (85546.12, 91289.06)	78714.52 (76084.85, 81104.31)	83497.38 (80998.84, 85932.17)	86105.33 (82570.47, 89529.94)	77918.30 (74494.51, 80949.32)	81990.44 (78811.98, 84981.74)
Japan	12536.84 (12528.30, 12545.39)	13229.54 (13221.07, 13238.01)	12831.01 (12825.04, 12836.99)	9020.63 (9013.52, 9027.74)	10846.43 (10838.60, 10854.28)	9904.80 (9899.52, 9910.08)	10756.69 (9080.21, 13691.40)	12409.85 (10601.86, 16320.62)	11560.68 (10110.92, 14869.84)	17036.82 (14373.25, 20432.45)	17848.67 (14802.54, 21650.37)	17437.46 (14840.18, 20972.52)
Jordan	66797.94 (66656.01, 66940.34)	67890.27 (67744.81, 68036.18)	67342.65 (67241.14, 67444.39)	95912.28 (95822.62, 96002.05)	98084.61 (97989.37, 98179.96)	96941.18 (96875.91, 97006.49)	97309.51 (91920.88, 99320.05)	98207.78 (92798.21, 100103.47)	97741.89 (92337.66, 99479.05)	97451.54 (91940.68, 99734.75)	97699.65 (91897.41, 99865.56)	97583.93 (91951.58, 99628.46)
Kazakhstan	60146.56 (60091.60, 60201.58)	62544.60 (62491.30, 62597.94)	61211.23 (61173.45, 61249.03)	76655.94 (76597.28, 76714.65)	81944.62 (81885.89, 82003.39)	79254.66 (79213.43, 79295.91)	75213.49 (73001.39, 77060.95)	80715.50 (78625.37, 82434.33)	77929.58 (75838.67, 79458.17)	74234.76 (71393.92, 76555.26)	80628.86 (77873.65, 82773.77)	77369.79 (74695.66, 79446.25)
Kenya	120660.45 (120574.02, 120746.94)	112492.16 (112410.61, 112573.78)	116504.47 (116445.12, 116563.86)	129341.73 (129283.18, 129400.33)	107490.72 (107440.58, 107540.88)	118349.29 (118311.15, 118387.46)	123840.58 (121118.37, 126612.96)	106644.09 (104021.64, 109399.23)	115150.68 (112723.78, 117554.38)	109447.51 (103116.30, 115495.20)	97592.63 (91265.14, 104137.03)	103291.88 (97295.24, 109332.56)
Kiribati	136236.04 (134609.16, 137899.30)	111555.55 (110242.80, 112890.51)	123543.81 (122518.69, 124582.49)	177045.74 (175675.75, 178435.59)	137912.92 (136837.28, 139000.20)	156537.51 (155688.28, 157393.94)	164880.73 (158958.61, 170850.31)	128697.29 (123362.42, 134133.22)	145815.73 (140450.20, 151200.51)	149875.31 (141933.76, 157190.19)	120823.02 (113981.06, 127093.57)	134347.83 (127393.95, 140348.35)
Kuwait	65362.96 (65157.60, 65569.42)	67306.11 (67079.64, 67533.61)	66352.04 (66201.27, 66503.34)	97368.97 (97215.34, 97522.84)	96518.34 (96346.30, 96690.71)	96998.40 (96884.33, 97112.61)	96708.04 (94917.57, 98431.89)	96245.85 (94577.85, 97553.10)	96516.34 (94969.43, 97974.16)	96361.33 (94042.09, 98631.62)	95778.24 (93839.68, 98009.54)	96096.73 (94151.24, 98222.25)

Kyrgyzstan	62762.19 (62654.90, 62869.71)	64770.17 (64665.07, 64875.45)	63652.50 (63578.11, 63726.99)	83516.95 (83414.99, 83619.05)	86780.00 (86679.38, 86880.74)	85084.26 (85013.03, 85155.56)	84064.57 (81996.53, 86964.59)	87550.70 (84525.77, 90816.68)	85771.66 (83425.70, 88572.78)	83717.66 (81264.83, 86468.66)	87871.09 (84866.53, 91003.18)	85766.38 (83391.00, 88560.86)
Latvia	106821.54 (106636.46, 107006.89)	111129.17 (110945.42, 111313.17)	109111.13 (108981.21, 109241.18)	103639.61 (103407.98, 103871.68)	106067.06 (105833.52, 106301.05)	104905.27 (104741.06, 105069.70)	102655.72 (98528.15, 106886.90)	112335.34 (107938.28, 117184.68)	107602.85 (103518.94, 111656.17)	76169.00 (65948.10, 84696.05)	86273.17 (75262.03, 95422.22)	81284.90 (70705.38, 89763.81)
Lebanon	78068.16 (77926.94, 78209.68)	76199.07 (76061.90, 76336.49)	77080.21 (76982.04, 77178.51)	112170.30 (112038.64, 112302.09)	109958.79 (109830.41, 110087.29)	111020.50 (110928.82, 111112.25)	114091.90 (110629.68, 117478.01)	109924.24 (106813.18, 112856.90)	112009.21 (108956.31, 114956.92)	113173.31 (109025.01, 117087.01)	108230.02 (104223.53, 112463.99)	110727.96 (106929.61, 114632.58)
Lesotho	153920.48 (153594.43, 154247.67)	115696.62 (115452.34, 115941.40)	134212.30 (134015.85, 134409.09)	162421.84 (162120.36, 162724.25)	124966.04 (124736.63, 125195.90)	142901.84 (142719.48, 143084.50)	140665.32 (133812.82, 149148.85)	110558.78 (105371.55, 117471.10)	124821.02 (119208.49, 132012.48)	132559.54 (125672.00, 138908.94)	108630.04 (102678.10, 114502.17)	119781.03 (114144.25, 124986.75)
Liberia	94578.36 (94349.80, 94807.54)	97812.47 (97575.19, 98050.41)	96317.41 (96153.01, 96482.11)	140415.36 (140228.97, 140602.06)	130458.37 (130276.67, 130640.35)	135632.47 (135502.20, 135762.90)	138624.62 (134867.99, 142415.10)	125699.65 (122045.14, 128847.19)	132275.91 (129017.39, 135223.62)	138619.42 (133472.79, 143794.71)	122027.29 (117050.13, 126381.67)	130245.73 (125576.12, 134738.59)
Libya	74063.61 (73935.10, 74192.38)	73347.56 (73216.76, 73478.62)	73714.96 (73623.48, 73806.57)	115481.20 (115354.77, 115607.75)	116444.51 (116314.68, 116574.46)	115944.77 (115854.21, 116035.40)	117003.77 (113572.53, 121247.65)	117931.14 (114633.86, 121799.57)	117447.12 (114168.77, 121568.28)	113931.26 (109551.13, 118631.56)	115541.00 (111232.69, 120385.48)	114712.15 (110509.30, 119158.63)
Lithuania	99016.87 (98867.42, 99166.52)	100506.43 (100359.79, 100653.25)	99860.74 (99756.32, 99965.24)	96596.97 (96412.39, 96781.86)	111341.29 (111144.56, 111538.34)	104201.94 (104067.30, 104336.73)	99805.79 (95576.05, 104408.04)	111780.12 (106988.18, 116748.55)	105992.98 (101473.83, 110601.18)	92741.62 (81374.79, 98597.32)	104637.89 (92545.00, 110729.37)	98794.50 (86960.35, 104489.99)
Luxembourg	44468.65 (44134.83, 44804.57)	40689.61 (40377.25, 41004.05)	42681.13 (42453.12, 42910.18)	45484.00 (45216.73, 45752.64)	41779.90 (41524.43, 42036.77)	43704.07 (43519.16, 43889.67)	48739.15 (46700.48, 51871.60)	45799.64 (43460.56, 49225.46)	47340.79 (45273.88, 50556.22)	46990.22 (45161.40, 49272.94)	43676.56 (41778.03, 46271.46)	45379.00 (43688.42, 47747.99)
Madagascar	125408.38 (125299.31, 125517.55)	116533.36 (116425.11, 116641.74)	120952.96 (120876.20, 121029.78)	148648.65 (148562.87, 148734.51)	123405.37 (123328.73, 123482.07)	135972.81 (135915.46, 136030.20)	142568.09 (139195.28, 145960.29)	121575.06 (118412.76, 124602.83)	132007.57 (128799.56, 134947.32)	129357.21 (124572.19, 133648.22)	110858.34 (106376.54, 114981.81)	120060.75 (115520.78, 123980.93)

Malawi	131881.37 (131743.44, 132019.54)	130332.19 (130203.81, 130460.75)	131078.57 (130984.64, 131172.59)	150574.25 (150465.42, 150683.21)	126899.75 (126811.30, 126988.27)	138271.39 (138202.69, 138340.13)	142774.13 (138432.28, 146149.89)	120833.35 (117115.08, 123555.53)	131269.18 (127389.92, 133867.18)	132329.98 (127206.87, 137037.67)	114378.58 (109241.32, 118123.43)	122820.39 (117939.32, 126550.23)
Malaysia	60192.11 (60128.94, 60255.36)	51117.59 (51062.22, 51173.02)	55529.85 (55488.04, 55571.70)	82438.12 (82389.81, 82486.45)	69170.92 (69125.79, 69216.08)	75936.29 (75903.21, 75969.37)	79760.74 (76653.09, 81576.95)	66714.22 (64246.38, 68920.35)	73324.55 (70607.69, 75020.30)	74511.19 (70132.82, 77978.48)	63963.26 (59917.08, 66858.60)	69265.58 (65151.78, 72310.01)
Maldives	72442.61 (71790.09, 73102.94)	59298.04 (58598.30, 60012.78)	66983.62 (66503.85, 67468.45)	81033.49 (80621.41, 81447.59)	66312.53 (65910.52, 66716.77)	74700.46 (74411.55, 74990.44)	84146.67 (79095.63, 89609.52)	68763.16 (64322.10, 74066.27)	77413.57 (73095.00, 82429.29)	81847.04 (75917.97, 89214.87)	71783.70 (65868.05, 78892.95)	77476.92 (72156.11, 84476.77)
Mali	134762.67 (134626.86, 134898.67)	138597.33 (138466.31, 138728.50)	136687.47 (136593.27, 136781.75)	163480.17 (163382.27, 163578.14)	145255.41 (145162.68, 145348.21)	154372.83 (154305.42, 154440.28)	159722.14 (156510.49, 163004.71)	142058.24 (139131.62, 144559.24)	150811.93 (148166.66, 153290.63)	151618.06 (147101.81, 156406.06)	137676.74 (133284.57, 141925.03)	144392.86 (140344.17, 148468.48)
Malta	33212.04 (32937.28, 33488.67)	29653.29 (29396.60, 29911.80)	31480.17 (31292.25, 31668.99)	31299.61 (31023.78, 31577.54)	28438.64 (28173.19, 28706.28)	29928.08 (29736.35, 30120.88)	31118.18 (29779.96, 32672.80)	28012.30 (26425.89, 29572.54)	29617.92 (28435.01, 31147.87)	37969.41 (34819.39, 41990.47)	32597.99 (29450.20, 36078.86)	35281.12 (32325.16, 38923.56)
Marshall Islands	122705.66 (120620.88, 124850.61)	106022.55 (104165.47, 107921.47)	114658.98 (113269.31, 116072.88)	153936.92 (152255.23, 155648.58)	121058.49 (119455.15, 122696.62)	137812.22 (136648.90, 138991.54)	151903.05 (148649.32, 154691.38)	120678.50 (118239.74, 122843.51)	136580.03 (134220.75, 138749.76)	141541.42 (135393.02, 146572.52)	111183.22 (106387.68, 114986.39)	126462.25 (121209.67, 130602.31)
Mauritania	134915.54 (134640.08, 135191.74)	130313.14 (130059.58, 130567.24)	132503.01 (132316.66, 132689.67)	178521.18 (178306.30, 178736.33)	156813.06 (156615.18, 157011.22)	167343.87 (167198.14, 167489.73)	165261.38 (159244.22, 170478.15)	148012.02 (142489.84, 152192.22)	156323.05 (150764.05, 160771.42)	146889.29 (139852.99, 154394.48)	135604.36 (129293.33, 141767.59)	141004.72 (134671.96, 147384.82)
Mauritius	73358.71 (73081.12, 73637.56)	55593.66 (55379.49, 55808.60)	63697.71 (63528.45, 63867.44)	82899.27 (82654.91, 83144.29)	66351.47 (66130.81, 66572.82)	74102.76 (73939.53, 74266.32)	82441.33 (78803.99, 86136.24)	69339.40 (65661.46, 73144.29)	75511.02 (72196.01, 78997.67)	90290.02 (85346.23, 95513.45)	78211.78 (73407.46, 83192.31)	84101.46 (79720.12, 89049.20)
Mexico	96223.49 (96189.21, 96257.79)	83114.69 (83083.56, 83145.83)	89511.94 (89488.85, 89535.04)	51523.23 (51504.69, 51541.77)	50173.10 (50155.00, 50191.21)	50956.42 (50943.44, 50969.39)	48437.94 (43596.22, 53296.60)	45069.32 (40278.92, 49923.73)	46784.47 (42492.40, 51286.59)	67007.90 (60708.79, 73106.31)	62248.14 (55967.15, 68910.61)	64617.42 (58798.31, 70489.19)
Mongolia	84747.99 (84571.12, 84925.54)	89385.29 (89203.89, 89567.11)	87017.89 (86891.78, 87144.26)	92450.32 (92299.17, 92601.85)	96711.45 (96561.59, 96861.57)	94584.45 (94478.46, 94690.59)	91907.27 (89613.94, 93862.12)	96346.68 (94096.53, 98445.68)	94107.09 (91955.89, 95992.16)	89699.40 (85784.43, 93398.40)	93180.79 (89852.76, 96638.04)	91382.78 (87927.78, 94670.62)

Montenegro	92907.38 (92566.88, 93249.06)	102037.15 (101680.58, 102394.71)	97586.68 (97340.22, 97833.66)	73570.92 (73240.54, 73902.59)	86569.36 (86210.19, 86929.86)	80089.16 (79845.37, 80333.60)	79467.69 (75360.24, 84988.67)	92027.00 (87819.14, 97164.05)	85767.18 (81754.43, 90669.26)	83801.77 (78791.69, 89718.17)	97266.01 (92141.36, 102618.50)	90400.65 (85807.94, 95452.84)
Morocco	82642.66 (82588.58, 82696.78)	82149.55 (82096.68, 82202.45)	82388.64 (82350.88, 82426.42)	104010.27 (103960.57, 104059.99)	102152.84 (102103.60, 102202.11)	103104.29 (103069.32, 103139.27)	102412.71 (100574.57, 104329.35)	100379.30 (98689.13, 101964.70)	101398.27 (99696.27, 102860.21)	101884.27 (99631.98, 104083.94)	99516.23 (97271.22, 101695.36)	100685.10 (98679.87, 102648.77)
Mozambique	117481.43 (117373.47, 117589.53)	113369.75 (113272.23, 113467.39)	115377.50 (115305.18, 115449.88)	129212.47 (129126.90, 129298.12)	101133.83 (101068.14, 101199.57)	114318.91 (114266.59, 114371.26)	124305.81 (121422.98, 126718.13)	98996.18 (96128.94, 101842.87)	110712.43 (108234.83, 113179.88)	123628.96 (119965.69, 126931.48)	100449.32 (97080.24, 103777.40)	110911.47 (107864.15, 113959.01)
Myanmar	88487.57 (88437.28, 88537.90)	74886.22 (74844.03, 74928.44)	81335.12 (81302.77, 81367.50)	82888.74 (82850.14, 82927.36)	66281.35 (66249.70, 66313.01)	73907.30 (73882.81, 73931.79)	81827.98 (78781.76, 86088.97)	68095.47 (65087.18, 73137.69)	74412.29 (71493.04, 78753.65)	80207.78 (76905.16, 84911.47)	65849.34 (62011.34, 71429.21)	72303.19 (68940.03, 77094.06)
Namibia	157478.21 (157114.43, 157843.25)	124753.62 (124457.77, 125050.29)	140529.50 (140297.97, 140761.52)	159256.01 (158989.42, 159523.14)	117564.21 (117358.37, 117770.38)	137223.57 (137058.92, 137388.42)	153916.69 (150163.94, 156750.31)	119380.29 (116715.98, 122385.03)	135448.94 (132820.09, 137961.63)	137457.86 (132956.82, 141071.61)	115886.14 (112552.55, 119247.10)	126237.37 (122904.20, 129093.24)
Nepal	163098.45 (163004.68, 163192.31)	194934.35 (194832.80, 195035.98)	178920.30 (178851.29, 178989.35)	140651.08 (140586.63, 140715.56)	155093.56 (155028.76, 155158.38)	148307.92 (148262.19, 148353.66)	139146.66 (133951.65, 142693.96)	152776.67 (147565.37, 157016.95)	146422.23 (141531.10, 150075.85)	129955.25 (122821.55, 136335.19)	143574.30 (135823.03, 150401.46)	137421.48 (130309.07, 143417.28)
Netherlands	33583.79 (33538.14, 33629.50)	29364.10 (29322.19, 29406.06)	31512.10 (31481.20, 31543.03)	35101.20 (35056.47, 35145.97)	31064.16 (31021.95, 31106.43)	33117.41 (33086.65, 33148.19)	36924.46 (35265.79, 39325.08)	33363.55 (31594.80, 36620.69)	35177.25 (33588.21, 37738.18)	35037.00 (34028.87, 36152.34)	31375.81 (30250.96, 32521.06)	33234.83 (32264.46, 34239.14)
New Zealand	44589.70 (44486.96, 44692.63)	42490.93 (42392.05, 42590.01)	43531.51 (43460.38, 43602.73)	46806.00 (46711.80, 46900.37)	44320.54 (44229.72, 44411.52)	45558.17 (45492.75, 45623.67)	40950.42 (37519.49, 43892.84)	37433.43 (34287.98, 40647.85)	39196.31 (36106.28, 42257.93)	37967.04 (34550.37, 41402.48)	35020.84 (31805.40, 38639.50)	36494.14 (33318.14, 39838.62)
Nicaragua	113157.02 (112972.21, 113342.26)	101231.90 (101064.42, 101399.66)	107089.84 (106966.03, 107213.82)	84187.32 (84080.38, 84294.41)	76055.05 (75955.84, 76154.37)	80324.91 (80251.99, 80397.90)	93415.27 (90447.29, 96676.19)	83927.36 (80957.31, 87003.51)	88786.59 (85982.90, 91876.86)	100172.68 (95404.77, 105472.31)	86693.64 (82618.38, 90718.76)	93473.87 (89347.37, 97989.79)

Niger	143219.00 (143056.32, 143382.00)	149498.08 (149336.36, 149660.06)	146515.34 (146401.03, 146629.79)	180635.73 (180525.95, 180745.62)	166598.35 (166497.49, 166699.31)	173308.38 (173234.04, 173382.77)	173912.10 (170212.82, 176886.58)	160744.76 (156884.53, 163388.64)	167044.40 (163614.84, 169706.48)	162766.73 (157439.17, 168622.05)	153971.12 (149041.98, 158624.82)	158136.46 (153417.80, 163191.54)
Nigeria	128088.64 (128048.82, 128128.49)	139618.92 (139577.46, 139660.38)	133643.73 (133615.40, 133672.06)	127588.86 (127560.85, 127616.88)	127227.50 (127201.02, 127253.99)	127268.10 (127248.90, 127287.31)	127561.71 (124950.80, 130033.57)	125419.20 (122662.96, 127748.22)	126338.11 (124066.19, 128480.24)	133830.96 (130473.70, 137509.23)	131533.31 (128372.79, 135170.91)	132494.03 (129377.65, 135932.46)
Northern Mariana Islands	107684.32 (105711.04, 109722.17)	86160.54 (84178.95, 88206.82)	98414.68 (97013.10, 99847.98)	170358.11 (168469.99, 172269.22)	126915.98 (125200.41, 128653.33)	149661.66 (148386.48, 150947.45)	163117.04 (158900.26, 166151.83)	123065.96 (119802.72, 125365.68)	143549.08 (140139.61, 145855.95)	163803.43 (158655.01, 168798.56)	121354.77 (117689.47, 124449.61)	142890.12 (139012.14, 146505.61)
Norway	42913.12 (42819.18, 43007.24)	38462.41 (38374.74, 38550.26)	40769.37 (40705.19, 40833.63)	44474.72 (44388.64, 44560.94)	39705.65 (39624.02, 39787.44)	42119.38 (42060.07, 42178.76)	45375.08 (43029.07, 48737.00)	40148.02 (38084.50, 43400.44)	42790.55 (40697.26, 45913.53)	45352.52 (42889.01, 48743.24)	39895.71 (37876.39, 42941.96)	42676.83 (40648.32, 45745.48)
Oman	76754.69 (76495.58, 77015.46)	74386.93 (74150.83, 74624.09)	75338.56 (75169.14, 75508.61)	117231.25 (117029.78, 117433.36)	116239.35 (116027.31, 116451.98)	116678.31 (116533.81, 116823.12)	111859.25 (106277.45, 115083.39)	112015.94 (106544.28, 115392.24)	111870.19 (106144.94, 114977.22)	100691.87 (97366.49, 103844.14)	100274.34 (97189.48, 103704.20)	100679.68 (97614.02, 103784.03)
Pakistan	162519.87 (162483.71, 162556.04)	181280.34 (181239.20, 181321.48)	171377.87 (171350.68, 171405.07)	141519.54 (141494.06, 141545.03)	144211.33 (144184.98, 144237.68)	142816.03 (142797.73, 142834.35)	138077.30 (132995.99, 142970.87)	137297.02 (131718.40, 142334.36)	137685.71 (132677.00, 142580.22)	132058.69 (126549.59, 137354.29)	131163.99 (125606.21, 136695.65)	131619.68 (126517.09, 136883.22)
Palestine	71234.61 (71043.81, 71426.08)	70895.88 (70717.81, 71074.46)	70989.73 (70860.01, 71119.74)	96799.08 (96649.20, 96949.30)	94975.30 (94834.93, 95115.90)	95917.59 (95815.69, 96019.62)	113943.34 (108697.23, 119376.99)	103107.79 (98685.15, 106944.38)	108621.84 (103971.62, 112972.17)	115131.28 (109370.20, 120623.36)	102184.31 (98272.65, 106086.40)	108764.32 (104537.19, 112942.46)
Panama	102452.20 (102261.06, 102643.70)	88527.18 (88343.85, 88710.86)	95547.63 (95415.18, 95680.25)	86384.48 (86257.59, 86511.51)	76548.95 (76428.89, 76669.16)	81562.77 (81475.36, 81650.25)	88816.36 (85836.67, 92361.97)	78004.25 (75318.34, 81361.01)	83491.10 (80877.81, 86768.29)	91067.59 (87681.14, 95342.97)	79073.75 (75515.70, 82845.22)	85166.06 (81937.03, 88873.84)
Papua New Guinea	133078.59 (132871.31, 133286.41)	104318.49 (104128.35, 104509.15)	119333.25 (119192.14, 119474.62)	176027.21 (175884.67, 176169.94)	129732.07 (129602.69, 129861.62)	153870.52 (153773.82, 153967.30)	171241.37 (168123.75, 173905.64)	125565.25 (122819.35, 128067.86)	149047.40 (146567.57, 151111.25)	166186.84 (162033.88, 169715.57)	125156.73 (121699.32, 128435.49)	145406.14 (142224.69, 148188.20)

Paraguay	77761.69 (77625.26, 77898.38)	69360.41 (69235.79, 69485.24)	73538.72 (73446.58, 73630.98)	90491.08 (90387.48, 90594.79)	88475.84 (88373.69, 88578.09)	89557.90 (89485.21, 89630.65)	92279.38 (89724.34, 95293.91)	86077.30 (83605.84, 89210.50)	89265.09 (86734.74, 91918.70)	94529.50 (91780.87, 97173.98)	88380.33 (85988.75, 90739.38)	91536.97 (89286.33, 93706.41)
Peru	75872.35 (75816.92, 75927.82)	72719.49 (72665.86, 72773.15)	74277.40 (74238.86, 74315.96)	111308.20 (111257.26, 111359.16)	104608.89 (104559.45, 104658.35)	108012.06 (107976.57, 108047.56)	105289.17 (100951.13, 108824.09)	98113.06 (93869.80, 101897.28)	101726.81 (97612.89, 105107.62)	101105.32 (96349.87, 105128.64)	92187.30 (87264.02, 96272.76)	96715.87 (91677.55, 100407.80)
Philippines	88610.50 (88569.50, 88651.53)	71900.12 (71864.12, 71936.14)	80190.67 (80163.47, 80217.88)	83222.99 (83195.14, 83250.85)	71691.04 (71666.89, 71715.19)	77443.75 (77425.51, 77461.99)	81947.72 (79584.58, 84158.08)	70061.94 (67838.65, 72529.50)	75907.33 (73784.29, 78133.71)	77053.24 (72830.30, 81345.03)	65047.80 (60472.82, 69324.11)	70877.24 (66635.56, 74847.39)
Poland	75777.65 (75737.34, 75817.98)	82861.75 (82820.01, 82903.51)	79476.36 (79447.36, 79505.36)	59488.43 (59447.44, 59529.44)	70085.59 (70041.75, 70129.45)	64815.97 (64785.99, 64845.97)	69553.61 (65408.78, 74998.72)	78397.01 (74095.27, 84647.67)	74029.09 (69979.14, 79697.35)	68506.93 (64239.46, 73445.23)	77452.89 (73200.42, 82648.38)	72995.66 (68932.88, 77573.44)
Portugal	35582.68 (35525.03, 35640.40)	31098.62 (31045.74, 31151.58)	33363.32 (33324.27, 33402.42)	32931.30 (32869.85, 32992.87)	29827.20 (29768.94, 29885.56)	31414.53 (31372.18, 31456.93)	34973.98 (32602.93, 37868.65)	31839.22 (29482.50, 35048.28)	33417.18 (31229.99, 36396.96)	37836.72 (34257.14, 44791.61)	35844.67 (30869.73, 44683.67)	36864.57 (32899.55, 44581.73)
Puerto Rico	73488.98 (73360.43, 73617.71)	68218.99 (68098.60, 68339.56)	70650.17 (70562.50, 70737.93)	104138.48 (103973.16, 104304.07)	98642.37 (98481.94, 98803.09)	101295.61 (101180.68, 101410.69)	101927.87 (97991.30, 104693.30)	96937.47 (92875.57, 99155.44)	99341.07 (95365.58, 101603.52)	101645.13 (97281.23, 104457.19)	97778.68 (93916.49, 100482.41)	99668.23 (95661.60, 102193.68)
Qatar	75540.58 (74928.83, 76166.55)	78494.87 (77861.28, 79137.40)	76815.68 (76391.45, 77245.20)	85905.79 (85604.35, 86209.58)	85747.25 (85256.96, 86245.02)	85704.38 (85452.03, 85958.49)	86452.12 (84487.27, 88398.69)	86398.58 (84293.79, 88155.83)	86378.41 (84584.44, 88054.06)	91128.96 (86707.40, 94503.20)	89029.48 (84552.95, 92306.25)	90422.47 (86038.77, 93471.96)
Romania	106672.56 (106611.37, 106733.77)	124550.39 (124484.65, 124616.15)	115735.48 (115690.59, 115780.39)	86674.49 (86606.49, 86742.54)	105392.91 (105318.04, 105467.84)	95986.78 (95936.30, 96037.28)	92123.34 (87901.63, 97149.37)	107663.72 (103288.11, 112834.54)	99834.70 (95732.25, 104856.38)	90252.80 (85908.99, 94912.36)	104352.70 (99445.44, 110074.14)	97229.24 (93332.07, 101706.20)
Russian Federation	101001.54 (100977.45, 101025.64)	106838.47 (106814.62, 106862.34)	103835.33 (103818.56, 103852.10)	101147.78 (101122.50, 101173.07)	115464.69 (115438.03, 115491.35)	108509.09 (108490.77, 108527.41)	101654.01 (100126.76, 103074.01)	114895.56 (112424.50, 116729.72)	108452.51 (106668.10, 109897.40)	100386.17 (95678.61, 104558.60)	109018.14 (103801.37, 113787.01)	104745.34 (100114.95, 108856.22)
Rwanda	129590.33 (129430.84, 129750.13)	136074.23 (135927.22, 136221.46)	132930.88 (132823.13, 133038.76)	132556.83 (132437.75, 132676.07)	111624.38 (111529.13, 111719.71)	121399.55 (121325.17, 121473.99)	130631.54 (127523.65, 133534.63)	111166.16 (107901.02, 114498.73)	120309.93 (117323.50, 123194.09)	129892.14 (125790.36, 134722.36)	112677.05 (108515.08, 117837.79)	121001.30 (117261.47, 125725.20)

Saint Lucia	88893.09 (88071.59, 89722.89)	75247.45 (74562.75, 75937.71)	81598.42 (81073.02, 82126.99)	123717.01 (122935.53, 124503.05)	110557.62 (109824.10, 111295.75)	117157.85 (116622.38, 117695.60)	123161.89 (120718.73, 125475.27)	109613.87 (107110.28, 111699.71)	116385.97 (114055.51, 118320.41)	117156.51 (113072.92, 121127.47)	102719.72 (98906.53, 106136.44)	109912.77 (106065.48, 113382.24)
Saint Vincent and the Grenadines	85606.21 (84719.59, 86501.92)	73203.91 (72437.95, 73977.16)	79128.12 (78548.48, 79711.53)	108623.36 (107758.61, 109494.05)	97938.31 (97100.88, 98781.91)	103403.86 (102801.31, 104009.44)	109552.92 (106911.75, 112274.65)	98295.01 (95746.84, 100250.55)	104032.36 (101714.51, 106306.98)	110564.11 (106987.06, 114010.71)	98083.89 (94856.22, 100918.90)	104324.01 (101043.76, 107278.74)
Samoa	100461.19 (99608.53, 101322.84)	82741.94 (81988.76, 83501.50)	91947.65 (91381.64, 92517.31)	156092.75 (155275.02, 156914.74)	123032.25 (122298.69, 123769.65)	140128.26 (139577.95, 140680.56)	151070.58 (147874.52, 154179.30)	119776.33 (116945.45, 122227.87)	136031.33 (133321.67, 138486.04)	147496.73 (143063.75, 150570.68)	117487.78 (113627.58, 120594.64)	133168.58 (129418.40, 136084.46)
Sao Tome and Principe	109059.36 (108048.93, 110081.38)	113479.10 (112518.45, 114448.31)	111271.68 (110576.87, 111971.38)	128296.29 (127468.34, 129130.31)	124681.05 (123891.38, 125475.91)	126512.91 (125941.35, 127087.27)	126860.00 (124528.14, 129847.08)	122521.44 (120364.30, 125099.73)	124703.86 (122795.17, 127333.29)	127020.23 (123743.92, 130073.44)	121774.05 (119289.64, 124609.45)	124354.31 (121880.90, 127037.13)
Saudi Arabia	90311.45 (90231.31, 90391.70)	88627.12 (88540.80, 88713.54)	89544.03 (89485.63, 89602.48)	103983.55 (103921.35, 104045.82)	104999.88 (104928.43, 105071.41)	104331.62 (104284.91, 104378.35)	104063.49 (102671.89, 105607.89)	104958.23 (103695.54, 106487.79)	104412.82 (103299.58, 105934.00)	103632.12 (101549.76, 106685.15)	103931.23 (102009.59, 106686.74)	103807.73 (101876.57, 106751.47)
Senegal	148605.41 (148451.74, 148759.27)	138154.21 (138010.54, 138298.06)	143309.50 (143204.46, 143414.65)	178435.84 (178322.12, 178549.65)	150756.75 (150656.29, 150857.29)	164117.71 (164042.16, 164193.30)	171802.96 (168958.43, 174102.14)	150551.41 (148349.51, 152819.63)	160743.59 (158505.20, 162607.78)	160926.82 (157304.30, 164133.38)	145430.73 (142437.26, 148013.06)	152935.00 (150109.47, 155557.57)
Serbia	98708.42 (98613.82, 98803.12)	97987.69 (97895.80, 98079.65)	98279.10 (98213.31, 98344.92)	82289.60 (82195.19, 82384.10)	82710.58 (82614.70, 82806.56)	82521.86 (82454.57, 82589.20)	84204.79 (81285.08, 88239.71)	87839.69 (84376.45, 93005.19)	86024.00 (82967.89, 90548.19)	85018.61 (81838.57, 89771.53)	92144.81 (87331.19, 98755.30)	88476.18 (84642.88, 93660.38)
Seychelles	61264.50 (60369.41, 62172.14)	48429.85 (47708.02, 49161.44)	54143.99 (53584.77, 54708.12)	75319.13 (74517.93, 76128.41)	60307.80 (59600.10, 61022.36)	67784.79 (67256.27, 68316.70)	98281.01 (91782.93, 104964.35)	84991.47 (79236.95, 90476.98)	91678.72 (85733.18, 97461.92)	93766.76 (84897.55, 104153.75)	79127.71 (71552.71, 86970.11)	86640.58 (78974.96, 95188.25)
Sierra Leone	129895.42 (129704.81, 130086.33)	119869.95 (119688.14, 120052.05)	124838.68 (124707.07, 124970.44)	164448.21 (164294.59, 164602.01)	137682.46 (137543.49, 137821.59)	151201.41 (151097.76, 151305.14)	159449.47 (155749.36, 162858.58)	134703.87 (131224.03, 138138.35)	147085.87 (143832.31, 150388.87)	155867.84 (150743.94, 160944.94)	133310.39 (128658.46, 137151.84)	144324.41 (140023.64, 148319.88)

Singapore	17773.92 (17701.02, 17847.18)	18834.71 (18763.61, 18906.06)	18276.00 (18225.41, 18326.73)	16550.85 (16507.71, 16594.12)	17657.31 (17612.58, 17702.19)	17007.24 (16976.42, 17038.13)	18163.15 (16232.44, 21303.43)	19355.09 (17710.34, 22114.58)	18699.24 (17073.17, 21294.10)	17596.29 (14653.79, 20773.03)	17886.94 (15544.30, 20394.01)	17721.30 (15305.96, 20459.37)
Slovakia	121164.06 (121028.42, 121299.83)	111432.21 (111303.65, 111560.89)	116461.53 (116368.11, 116555.00)	98398.65 (98263.37, 98534.09)	92799.32 (92669.06, 92929.74)	95590.78 (95496.90, 95684.74)	59037.86 (53662.22, 66836.57)	53674.11 (48033.80, 61880.62)	56372.36 (50869.90, 64490.96)	57082.59 (52131.40, 64307.35)	54665.99 (49515.14, 62355.38)	55913.87 (50976.11, 63076.10)
Slovenia	93584.63 (93384.11, 93785.53)	101575.83 (101369.67, 101782.36)	97760.93 (97617.31, 97904.75)	78337.46 (78135.94, 78539.43)	87007.04 (86794.09, 87220.45)	82557.51 (82411.16, 82704.10)	79749.87 (77227.35, 82711.23)	84483.18 (81196.86, 87764.58)	82008.17 (79523.19, 84801.94)	78852.55 (75419.85, 82233.62)	82808.99 (78634.20, 86663.10)	80756.68 (77166.24, 84005.33)
Solomon Islands	167816.50 (166986.59, 168654.36)	136552.71 (135699.06, 137416.90)	153180.32 (152585.91, 153779.24)	200143.18 (199544.74, 200744.52)	168025.96 (167456.76, 168598.36)	184367.97 (183954.64, 184782.82)	192186.30 (186605.07, 195457.46)	158247.09 (153672.47, 162017.09)	175488.75 (170871.42, 178929.12)	182279.79 (176034.49, 187258.16)	149062.77 (143632.31, 154009.18)	165746.22 (160425.57, 170277.33)
Somalia	127478.52 (127310.20, 127647.20)	136547.19 (136385.45, 136709.20)	131931.44 (131815.18, 132047.85)	148899.72 (148779.07, 149020.58)	140473.51 (140375.29, 140571.84)	144486.33 (144410.95, 144561.78)	142500.71 (139411.30, 145180.49)	136997.35 (134330.11, 139557.50)	139753.24 (137172.53, 142083.54)	141894.43 (137959.62, 145592.67)	133994.43 (130535.61, 137562.75)	138005.66 (134686.25, 141374.84)
South Africa	113161.65 (113102.36, 113220.98)	103687.54 (103637.10, 103738.00)	108086.74 (108048.40, 108125.10)	98857.21 (98815.69, 98898.75)	88857.03 (88821.45, 88892.62)	93666.07 (93639.18, 93692.96)	97805.38 (95080.78, 101043.56)	88432.12 (85976.96, 91276.97)	92932.52 (90665.44, 95652.81)	100792.79 (97506.69, 105534.54)	89588.01 (86305.03, 93879.20)	94855.85 (92025.19, 98987.24)
South Sudan	138330.44 (138166.08, 138495.08)	138543.53 (138368.96, 138718.34)	138583.53 (138464.31, 138702.87)	169937.85 (169790.32, 170085.55)	150326.21 (150184.84, 150467.74)	160008.02 (159906.10, 160110.04)	162940.68 (158387.53, 166444.92)	143591.36 (139748.04, 146634.90)	153295.80 (149301.17, 156339.91)	154134.08 (147580.81, 159675.26)	136072.23 (130117.97, 141142.67)	144709.20 (138451.73, 149707.49)
Spain	40752.57 (40721.03, 40784.12)	35549.26 (35520.10, 35578.44)	38192.64 (38171.20, 38214.10)	39876.27 (39845.65, 39906.92)	35507.74 (35478.80, 35536.71)	37719.23 (37698.16, 37740.32)	42256.36 (39719.39, 46943.30)	37632.47 (34693.64, 42721.19)	39971.98 (37373.54, 44989.72)	40580.14 (38164.34, 44490.27)	35604.91 (32934.63, 40226.40)	38127.89 (35665.13, 42197.46)
Sri Lanka	69309.68 (69244.14, 69375.29)	53634.99 (53577.99, 53692.05)	61498.79 (61455.35, 61542.26)	75993.48 (75938.79, 76048.20)	62211.84 (62164.58, 62259.13)	68621.35 (68585.64, 68657.07)	73957.48 (71544.92, 76297.54)	62022.40 (59473.70, 64412.10)	67537.58 (65243.88, 69838.90)	79052.94 (76067.30, 82393.09)	65912.94 (62893.33, 69168.12)	72104.99 (69192.69, 75106.50)

Sudan	96976.00 (96908.24, 97043.84)	95257.33 (95189.77, 95324.96)	96148.07 (96100.27, 96195.90)	133785.21 (133728.92, 133841.53)	128734.80 (128676.44, 128793.20)	131408.01 (131367.54, 131448.49)	128264.75 (123271.41, 131967.16)	123567.94 (118986.56, 127058.70)	126020.30 (121502.26, 129347.41)	132069.63 (124068.22, 140148.85)	123968.11 (116812.43, 131265.25)	127984.11 (120732.69, 135412.40)
Suriname	91817.95 (91340.21, 92298.13)	75792.00 (75373.53, 76212.52)	83699.51 (83383.78, 84016.30)	110052.27 (109657.00, 110448.76)	97917.63 (97556.43, 98279.93)	103985.66 (103718.71, 104253.16)	109673.79 (107067.24, 112150.09)	97534.74 (94749.93, 99937.21)	103588.11 (101120.09, 106033.51)	106749.22 (103827.01, 110594.34)	92637.71 (89707.24, 95727.66)	99527.30 (97019.72, 102839.80)
Sweden	42338.80 (42271.88, 42405.80)	38097.43 (38034.48, 38160.47)	40329.53 (40283.57, 40375.53)	46455.21 (46392.97, 46517.53)	41331.49 (41272.11, 41390.94)	43919.60 (43876.57, 43962.67)	45653.39 (44406.76, 46810.20)	40973.70 (39695.65, 42204.68)	43340.62 (42132.21, 44391.21)	44928.34 (43456.33, 46323.73)	40112.99 (38791.38, 41505.96)	42558.30 (41184.48, 43901.71)
Switzerland	37051.03 (36979.59, 37122.59)	33764.19 (33696.62, 33831.88)	35431.70 (35382.65, 35480.81)	37448.76 (37383.73, 37513.88)	34889.40 (34826.73, 34952.18)	36235.11 (36189.93, 36280.34)	37387.03 (36214.09, 38596.51)	34874.11 (33671.68, 36111.47)	36175.33 (35171.38, 37275.57)	37003.94 (35642.92, 38790.00)	34321.91 (32875.54, 36056.79)	35692.04 (34388.99, 37216.16)
Tajikistan	83448.73 (83334.15, 83563.53)	83403.27 (83292.37, 83514.34)	83303.85 (83224.65, 83383.14)	100190.52 (100092.91, 100288.32)	104140.01 (104044.18, 104235.98)	102057.44 (101989.46, 102125.50)	97793.65 (94864.64, 100338.06)	102022.91 (98755.04, 104461.15)	99825.51 (96978.47, 102145.30)	98196.30 (94289.44, 101504.00)	102475.21 (98604.40, 106000.70)	100236.98 (96514.57, 103457.85)
Thailand	67782.27 (67744.91, 67819.66)	54059.65 (54028.89, 54090.42)	60499.97 (60476.17, 60523.78)	75995.61 (75965.09, 76026.14)	56854.80 (56827.98, 56881.63)	65991.51 (65971.32, 66011.70)	71169.41 (65807.85, 76356.52)	51979.22 (47349.79, 57228.77)	61185.96 (56355.17, 66304.42)	75942.29 (70739.42, 81056.68)	57089.10 (52228.43, 62406.20)	66268.99 (61496.59, 70855.11)
Timor-Leste	90703.40 (90271.35, 91138.23)	78655.78 (78262.60, 79051.73)	84800.29 (84508.14, 85093.82)	93304.43 (93037.95, 93571.76)	73273.92 (73036.94, 73511.76)	83397.66 (83219.18, 83576.58)	102867.90 (97150.38, 108934.82)	83271.03 (77504.70, 89003.42)	93132.66 (87765.46, 98441.83)	95293.85 (88919.29, 101684.83)	82169.29 (75878.94, 87773.19)	88665.54 (82862.03, 94355.97)
Togo	135064.59 (134826.41, 135303.39)	130948.76 (130732.70, 131165.30)	132759.20 (132599.32, 132919.36)	166255.03 (166085.23, 166425.13)	150886.82 (150747.39, 151026.42)	157834.25 (157727.06, 157941.55)	161085.19 (158207.72, 163728.21)	146544.65 (143953.35, 148877.81)	153142.65 (150714.69, 155415.31)	156208.33 (152904.82, 159416.17)	141710.28 (138796.39, 144640.48)	148275.22 (145352.96, 151244.83)
Tonga	98034.86 (96980.72, 99102.22)	84314.42 (83364.10, 85275.33)	91063.13 (90356.48, 91775.60)	149703.83 (148574.45, 150841.01)	115326.28 (114368.57, 116290.51)	132363.48 (131626.23, 133104.13)	146212.81 (143444.43, 148419.89)	114780.93 (112878.35, 116619.06)	130531.90 (128688.21, 132098.60)	143460.99 (139902.58, 146945.68)	113656.21 (110881.26, 116329.45)	128544.00 (125621.62, 131277.79)
Trinidad and Tobago	62346.39 (62126.51, 62567.06)	53028.25 (52834.33, 53222.79)	57547.27 (57401.76, 57693.11)	85577.76 (85352.51, 85803.54)	75552.17 (75340.65, 75764.22)	80587.46 (80433.09, 80742.09)	82060.80 (78689.31, 85218.97)	72605.09 (69601.11, 75634.21)	77333.80 (74285.38, 80242.15)	83142.47 (79187.24, 86491.10)	75185.81 (71639.34, 78321.72)	79160.50 (75683.80, 82337.90)

Tunisia	74034.46 (73948.46, 74120.59)	74359.53 (74271.99, 74447.19)	74214.22 (74152.87, 74275.63)	100952.32 (100867.35, 101037.35)	100179.51 (100094.80, 100264.29)	100581.37 (100521.42, 100641.36)	98880.28 (95066.44, 100780.54)	97305.02 (92758.48, 99415.88)	98088.54 (94059.96, 99904.87)	96823.99 (91403.54, 99036.57)	96151.44 (90551.44, 98239.46)	96457.65 (90899.80, 98428.58)
Turkey	70604.14 (70572.39, 70635.92)	68094.07 (68063.43, 68124.72)	69356.25 (69334.24, 69378.27)	111787.62 (111752.66, 111822.58)	107428.73 (107394.63, 107462.84)	109602.45 (109578.08, 109626.84)	103595.88 (98923.13, 108585.00)	101391.16 (96175.94, 106117.00)	102413.07 (97846.42, 106769.42)	96894.70 (91436.73, 101705.56)	95511.28 (90168.91, 100869.84)	96117.64 (91184.21, 100982.99)
Turkmenistan	70802.63 (70671.91, 70933.76)	67669.95 (67550.85, 67789.30)	69044.38 (68957.25, 69131.64)	74051.07 (73944.01, 74158.33)	78848.45 (78737.64, 78959.40)	76235.18 (76158.75, 76311.70)	74408.76 (72113.96, 77163.42)	78214.73 (75935.74, 81047.24)	76133.94 (74031.53, 78855.70)	79405.41 (74054.48, 84515.67)	83647.77 (78436.39, 89061.32)	81539.11 (76438.49, 86552.39)
Uganda	120768.32 (120670.00, 120866.76)	117730.40 (117637.86, 117823.03)	119252.83 (119185.50, 119320.20)	133955.79 (133882.43, 134029.20)	110811.43 (110752.47, 110870.43)	121740.09 (121693.98, 121786.23)	128377.79 (125452.87, 131060.53)	107136.62 (104252.89, 110295.43)	117180.73 (114449.93, 119787.92)	126179.73 (122578.63, 129613.66)	108244.27 (104901.38, 111604.90)	116737.01 (113374.07, 119861.77)
Ukraine	74503.21 (74468.16, 74538.28)	75454.69 (75420.57, 75488.83)	75015.42 (74991.09, 75039.76)	84584.10 (84540.51, 84627.70)	87817.75 (87774.11, 87861.41)	86355.52 (86324.73, 86386.31)	81733.89 (78990.31, 84451.78)	86042.07 (83526.37, 89002.91)	84053.99 (81669.60, 86589.70)	84640.81 (81465.89, 87883.31)	89688.62 (85926.88, 93047.33)	87231.99 (84015.37, 90259.59)
United Arab Emirates	71676.53 (71366.79, 71989.45)	70715.30 (70361.87, 71073.24)	71205.71 (70974.51, 71438.76)	103509.53 (103323.10, 103696.64)	104165.71 (103916.90, 104415.89)	103842.51 (103694.94, 103990.53)	102103.59 (98167.47, 104485.74)	101674.60 (98307.88, 103893.78)	102139.17 (98435.77, 104281.49)	55848.28 (46263.28, 70853.05)	48594.91 (39954.84, 62233.55)	53543.93 (45066.11, 67789.57)
United Kingdom	36161.27 (36137.37, 36185.19)	31756.44 (31734.39, 31778.51)	34000.94 (33984.70, 34017.18)	36452.23 (36429.57, 36474.90)	32895.50 (32874.08, 32916.92)	34732.03 (34716.43, 34747.64)	36141.64 (35126.70, 36907.01)	32940.13 (31887.52, 33940.40)	34595.24 (33640.27, 35337.35)	36761.65 (35806.03, 37762.07)	33354.58 (32447.22, 34171.60)	35106.70 (34312.08, 35902.78)
Uruguay	43808.91 (43703.19, 43914.84)	33476.01 (33385.50, 33566.72)	38433.64 (38364.51, 38502.88)	50270.20 (50159.47, 50381.14)	37397.97 (37303.92, 37492.23)	43609.16 (43536.84, 43681.57)	52159.52 (49322.06, 55384.48)	42459.87 (39624.99, 46044.07)	47176.07 (44445.30, 50566.03)	51953.54 (47731.42, 57313.21)	45341.48 (40390.36, 51258.16)	48584.91 (44255.10, 54092.53)
Uzbekistan	65454.57 (65402.84, 65506.36)	65831.74 (65781.77, 65881.75)	65546.08 (65510.44, 65581.75)	78340.40 (78293.89, 78386.99)	81904.54 (81860.29, 81948.84)	80056.36 (80024.72, 80088.02)	79283.53 (75626.20, 82668.99)	81763.79 (77537.28, 86134.66)	80465.70 (76709.90, 84107.47)	74855.49 (72001.31, 77920.38)	77169.66 (73949.77, 80522.69)	75925.20 (73013.11, 79002.46)
Vanuatu	128221.35 (127222.92, 129230.22)	109426.27 (108406.92, 110458.98)	119395.10 (118681.55, 120114.48)	171764.73 (171014.54, 172518.86)	137019.47 (136322.02, 137721.27)	154794.56 (154281.24, 155309.94)	164839.82 (159566.80, 168249.13)	131298.82 (127576.17, 133866.07)	148252.35 (143971.28, 150882.48)	158797.08 (153903.66, 164265.56)	126027.81 (122531.13, 129947.20)	142082.76 (138036.27, 146507.18)

Yemen	102884.46 (102778.16, 102990.98)	104063.87 (103973.26, 104154.61)	103263.02 (103195.44, 103330.67)	129683.12 (129615.85, 129750.44)	133492.58 (133425.37, 133559.85)	131561.73 (131514.21, 131609.28)	133263.61 (130443.34, 136166.41)	133922.00 (131470.13, 136161.30)	133567.18 (131526.63, 135751.69)	133353.09 (128092.07, 139118.74)	133799.88 (128312.92, 139008.97)	133544.21 (128822.53, 138632.77)
Zambia	135151.02 (135001.14, 135301.12)	138492.35 (138337.00, 138647.94)	136917.26 (136809.53, 137025.11)	138814.22 (138709.68, 138918.87)	123848.11 (123754.04, 123942.27)	131329.27 (131259.23, 131399.37)	129209.07 (124639.59, 133030.22)	116680.00 (112847.49, 120135.42)	122932.14 (119058.52, 126286.78)	115557.66 (110990.56, 119775.60)	105937.69 (101488.95, 110030.62)	110648.19 (106404.09, 114500.36)
Zimbabwe	137509.63 (137368.80, 137650.71)	99895.70 (99785.46, 100006.09)	118353.78 (118265.92, 118441.74)	156782.03 (156664.40, 156899.79)	115176.62 (115088.17, 115265.15)	134354.16 (134282.74, 134425.64)	151230.75 (146830.12, 155230.67)	113806.65 (110373.80, 117143.57)	131117.84 (127766.49, 134040.78)	146914.85 (143648.17, 150108.45)	114618.43 (111792.72, 117428.94)	129641.41 (127011.57, 132114.21)
Bolivia (Plurinational State of)	81249.66 (81141.21, 81358.33)	80649.00 (80544.21, 80753.95)	81033.49 (80958.07, 81108.99)	113718.65 (113629.93, 113807.45)	108385.98 (108300.10, 108471.93)	111180.67 (111118.89, 111242.49)	114138.08 (110177.50, 118203.45)	108515.58 (104950.66, 111766.02)	111447.25 (108042.82, 114774.70)	109700.16 (105256.70, 114551.50)	104428.05 (100042.40, 108434.05)	107161.52 (103177.27, 111117.21)
Brunei Darussalam	16339.00 (16034.47, 16660.11)	17362.54 (17057.83, 17673.97)	16724.10 (16516.14, 16936.46)	16409.34 (16220.04, 16603.42)	16884.66 (16708.73, 17062.95)	16534.71 (16409.48, 16661.44)	15977.98 (13841.32, 18736.72)	16474.51 (14494.37, 19166.52)	16155.89 (14256.50, 18810.97)	16608.81 (14474.26, 20230.78)	16903.34 (15156.70, 20254.66)	16707.13 (14803.00, 20305.60)
Cabo Verde	119090.29 (118466.81, 119717.24)	110648.11 (110133.69, 111164.87)	114031.83 (113637.10, 114427.94)	127200.31 (126739.62, 127662.67)	114435.27 (114024.94, 114846.90)	120675.41 (120370.10, 120981.44)	119791.98 (113892.37, 125993.93)	107077.77 (101335.86, 112225.00)	113268.95 (107626.16, 118849.92)	120892.55 (115678.36, 126211.51)	106482.87 (101911.58, 110959.96)	113597.81 (108984.15, 118229.90)
Democratic Republic of the Congo	92773.17 (92709.93, 92836.48)	81088.41 (81029.07, 81147.83)	86637.45 (86594.27, 86680.66)	137319.93 (137271.61, 137368.28)	104942.52 (104904.61, 104980.44)	120913.50 (120883.43, 120943.57)	136126.29 (134141.65, 137882.47)	105005.78 (103274.38, 106802.29)	120294.19 (118832.53, 121845.68)	139228.44 (134387.00, 144584.27)	112545.12 (107180.04, 118853.78)	125564.03 (121053.87, 131073.20)
Cote d'Ivoire	118534.91 (118410.09, 118659.96)	119629.79 (119501.38, 119758.43)	119191.48 (119102.04, 119281.03)	145343.10 (145259.46, 145426.82)	140130.70 (140047.64, 140213.83)	142997.72 (142938.80, 143056.66)	130086.46 (123091.25, 135782.82)	124651.46 (118385.65, 130267.21)	127531.74 (121050.15, 132834.40)	124429.96 (117548.37, 130885.07)	115385.26 (108661.03, 122385.92)	119938.52 (113399.73, 126370.26)
Czechia	101352.61 (101260.82, 101444.47)	109772.40 (109677.86, 109867.01)	105647.93 (105582.14, 105713.76)	89532.59 (89441.04, 89624.23)	98537.26 (98440.97, 98633.63)	93959.74 (93893.44, 94026.09)	79827.70 (73728.96, 85830.73)	87377.58 (80917.06, 93550.48)	83537.86 (77357.18, 89249.09)	76892.68 (70367.45, 82864.68)	84517.76 (77151.88, 91047.32)	80626.75 (73852.03, 86747.30)

Micronesia (Federated States of)	113866.83 (112632.32, 115122.69)	99023.58 (97907.03, 100156.33)	106695.87 (105865.74, 107535.11)	149985.41 (148740.87, 151245.34)	118086.55 (117025.85, 119157.77)	134253.09 (133441.78, 135070.52)	146877.41 (143670.11, 149950.61)	115913.73 (113440.15, 118048.31)	131602.13 (128973.66, 133667.22)	141205.72 (136312.97, 145309.38)	110737.89 (106863.95, 114419.99)	126091.31 (122240.24, 129528.69)
Georgia	76827.89 (76720.15, 76935.78)	79746.63 (79640.10, 79853.28)	78266.09 (78190.67, 78341.56)	102218.76 (102061.13, 102376.59)	106751.10 (106586.82, 106915.59)	104360.20 (104246.69, 104473.81)	103090.76 (100829.19, 106208.85)	107618.69 (104781.99, 110615.45)	105253.17 (102922.64, 108004.63)	104308.29 (100642.16, 108327.20)	109862.47 (106153.86, 114384.59)	106925.40 (103450.00, 110962.47)
Iran (Islamic Republic of)	73053.54 (73011.98, 73095.15)	71496.09 (71457.54, 71534.67)	72271.67 (72243.54, 72299.83)	113593.84 (113559.96, 113627.74)	113977.03 (113942.61, 114011.46)	113774.64 (113750.49, 113798.78)	105969.53 (101974.66, 109229.67)	105142.31 (100834.32, 108425.89)	105545.86 (101541.61, 108532.16)	102724.44 (98209.58, 106518.78)	102567.05 (97840.61, 106713.82)	102630.80 (98048.74, 106380.04)
Lao People's Democratic Republic	80433.61 (80273.87, 80593.85)	69574.44 (69442.87, 69706.34)	74691.84 (74590.37, 74793.51)	77674.38 (77563.57, 77785.36)	60844.23 (60750.98, 60937.64)	69073.75 (69001.94, 69145.63)	80030.52 (75831.84, 84358.40)	62647.85 (59293.12, 67192.93)	71134.97 (67990.98, 75362.19)	77150.44 (73428.21, 81183.79)	61376.19 (57592.60, 65229.35)	69038.84 (65225.32, 72656.63)
North Macedonia	106684.40 (106482.69, 106886.49)	124889.23 (124669.25, 125109.56)	115757.71 (115608.57, 115907.04)	88165.56 (87967.63, 88363.87)	110332.96 (110110.95, 110555.37)	98975.38 (98827.12, 99123.83)	90855.15 (87736.13, 94477.72)	112679.11 (108576.79, 116089.19)	101520.67 (98237.36, 104915.30)	89554.08 (85916.78, 93773.32)	104679.74 (99969.83, 109555.56)	96939.47 (93370.62, 101022.90)
Republic of Moldova	85120.71 (84995.63, 85245.99)	88883.38 (88760.65, 89006.25)	87175.82 (87088.39, 87263.34)	89776.00 (89624.66, 89927.58)	98891.55 (98734.87, 99048.46)	94470.60 (94361.80, 94579.52)	89110.81 (85199.92, 92984.48)	96445.22 (92133.78, 100534.66)	92881.28 (89147.08, 96795.51)	86304.18 (81801.95, 91982.47)	92192.29 (87297.33, 98303.20)	89301.18 (84904.61, 94946.43)
Democratic People's Republic of Korea	65559.76 (65501.99, 65617.62)	61642.74 (61596.13, 61689.38)	63124.99 (63089.87, 63160.13)	89302.59 (89246.13, 89359.08)	78820.26 (78768.81, 78871.73)	83229.63 (83192.36, 83266.91)	84629.14 (80932.58, 88182.64)	78797.05 (74641.57, 83284.89)	81218.72 (77727.30, 84973.30)	87909.57 (82752.21, 93923.38)	83128.06 (77553.38, 89115.27)	85281.53 (80117.92, 90925.19)
Republic of Korea	20829.01 (20806.94, 20851.13)	21588.49 (21568.24, 21608.76)	21108.91 (21094.32, 21123.51)	20324.51 (20308.72, 20340.33)	20752.80 (20736.76, 20768.85)	20391.91 (20380.74, 20403.09)	19377.88 (16476.69, 22302.68)	20017.99 (17254.61, 23201.66)	19615.64 (16973.20, 22587.81)	29598.35 (25283.57, 33155.38)	29577.68 (25160.72, 33768.54)	29561.88 (25544.73, 33158.30)
Eswatini	153278.86 (152720.72, 153840.56)	122487.87 (122067.23, 122910.21)	136882.10 (136544.71, 137220.61)	162233.74 (161795.10, 162674.59)	127756.73 (127432.68, 128081.74)	143774.08 (143514.17, 144034.62)	153439.65 (147929.57, 157559.16)	124243.34 (120219.61, 127499.29)	137807.23 (133496.32, 141242.81)	143381.35 (138214.18, 148593.53)	116596.47 (112628.91, 120690.83)	128442.63 (124123.57, 132476.54)

Syrian Arab Republic	71765.62 (71692.23, 71839.10)	75919.39 (75841.39, 75997.48)	73781.44 (73727.99, 73834.94)	97348.86 (97270.39, 97427.39)	101569.27 (101489.36, 101649.25)	99529.50 (99473.70, 99585.32)	99700.26 (97458.28, 102187.38)	99873.36 (97343.46, 102257.86)	99810.88 (97652.50, 102036.07)	98127.38 (95364.14, 100855.75)	99039.71 (96305.89, 101497.65)	98566.55 (96061.20, 100912.56)
Taiwan (Province of China)	52836.94 (52787.47, 52886.46)	48293.67 (48246.15, 48341.23)	50663.27 (50629.05, 50697.51)	71495.55 (71441.64, 71549.51)	58057.05 (58006.01, 58108.12)	64441.35 (64404.34, 64478.38)	68270.92 (64855.60, 70462.54)	56285.54 (52773.65, 58646.10)	61961.17 (58758.41, 63960.87)	67227.55 (62865.73, 70025.19)	55346.09 (50949.53, 57792.29)	60922.08 (56794.99, 63260.35)
United Republic of Tanzania	114595.67 (114517.67, 114673.76)	108453.99 (108382.27, 108525.77)	111439.82 (111387.06, 111492.60)	121699.56 (121647.05, 121752.10)	105422.97 (105376.74, 105469.21)	113278.14 (113243.37, 113312.92)	121247.18 (117232.53, 124806.87)	105484.93 (101454.81, 109209.89)	113070.50 (109182.25, 116399.23)	111634.87 (107135.73, 116275.66)	95496.64 (90775.61, 100148.92)	103120.30 (98893.27, 107848.93)
Bahamas	73736.99 (73170.84, 74308.02)	60318.31 (59860.49, 60779.27)	66350.50 (65995.72, 66707.15)	99680.28 (99193.00, 100169.64)	87841.49 (87401.11, 88283.88)	93547.22 (93220.41, 93875.06)	99525.77 (96472.42, 102095.13)	88446.04 (85708.48, 91059.11)	93794.48 (91106.17, 96142.70)	96553.58 (93936.90, 99155.89)	86431.37 (83643.61, 89274.57)	91354.84 (88973.25, 93881.06)
Gambia	124362.63 (123935.11, 124792.58)	118688.94 (118281.79, 119097.78)	121859.99 (121566.86, 122154.10)	153791.57 (153501.08, 154082.71)	137827.19 (137563.12, 138091.77)	145737.24 (145541.50, 145933.27)	141862.84 (136804.11, 146558.31)	127747.60 (123140.57, 131903.50)	134686.02 (129858.72, 138882.43)	130575.41 (125713.88, 135969.59)	120072.56 (115353.71, 125032.76)	125092.41 (120616.43, 129761.32)
United States of America	44223.43 (44211.34, 44235.53)	45380.16 (45368.14, 45392.18)	44870.56 (44862.03, 44879.09)	46888.33 (46877.03, 46899.64)	48176.51 (48165.13, 48187.89)	47583.97 (47575.94, 47591.99)	45406.02 (43057.31, 48307.63)	48371.28 (45697.84, 51621.37)	46916.14 (44654.48, 49644.63)	46079.57 (43814.30, 49433.00)	48319.91 (45883.05, 51973.60)	47201.36 (45001.49, 50499.44)
Venezuela (Bolivarian Republic of)	94412.06 (94340.81, 94483.38)	80493.38 (80428.44, 80558.38)	87475.04 (87426.92, 87523.19)	92661.36 (92609.32, 92713.42)	78189.90 (78142.97, 78236.85)	85365.80 (85330.85, 85400.76)	93942.69 (89143.24, 98369.28)	79265.34 (74199.72, 84908.47)	86580.13 (81999.52, 91381.55)	91662.36 (85725.95, 97468.42)	76784.20 (70986.40, 83230.54)	84096.77 (78545.35, 90008.11)
Viet Nam	67065.46 (67031.29, 67099.64)	51189.54 (51164.46, 51214.62)	58059.53 (58039.39, 58079.68)	70747.85 (70720.78, 70774.93)	51594.20 (51572.86, 51615.54)	60265.24 (60248.56, 60281.94)	71509.47 (67533.76, 75791.67)	57178.01 (53366.81, 61786.95)	63584.41 (59643.44, 67887.42)	71385.07 (66853.06, 77679.76)	61013.88 (56164.00, 68065.41)	65993.55 (61665.96, 72591.28)
United States Virgin Islands	81216.07 (80383.45, 82057.82)	71568.08 (70832.09, 72311.01)	76084.42 (75533.60, 76639.14)	109640.98 (108690.07, 110599.03)	102608.13 (101721.39, 103501.85)	106032.36 (105384.47, 106683.78)	107502.30 (105012.11, 110438.30)	99137.09 (96708.93, 101103.37)	103189.97 (100956.17, 105263.08)	108070.05 (104217.22, 113683.00)	97559.93 (94888.65, 100522.93)	102732.83 (99788.30, 106618.07)
Monaco	37255.85 (36026.32, 38522.53)	33381.92 (32212.05, 34590.38)	35374.43 (34523.00, 36244.71)	35853.28 (34789.74, 36945.10)	31680.18 (30686.17, 32702.35)	33740.83 (33011.66, 34484.08)	35924.23 (34503.14, 37681.28)	32067.90 (30628.73, 33899.02)	33969.77 (32636.40, 35764.32)	36191.95 (34019.31, 39449.40)	32989.76 (31139.85, 37333.70)	34567.98 (32774.05, 38281.74)

San Marino	36951.38 (35760.43, 38175.83)	33394.52 (32278.50, 34543.89)	35235.87 (34418.17, 36070.21)	35850.62 (34792.51, 36936.54)	32422.36 (31426.26, 33446.58)	34154.23 (33426.15, 34896.24)	35634.62 (34488.33, 37014.62)	32447.16 (31288.27, 33601.38)	34055.84 (33053.98, 35292.09)	36019.03 (33561.49, 40179.76)	33302.45 (31205.83, 38193.94)	34680.07 (32393.02, 39065.22)
Saint Kitts and Nevis	102827.92 (101304.26, 104373.05)	83530.83 (82257.14, 84824.28)	92794.29 (91811.21, 93787.60)	118009.23 (116695.95, 119337.44)	100670.94 (99474.63, 101880.01)	109407.61 (108522.79, 110298.77)	115982.18 (113039.43, 118234.69)	99732.26 (96794.99, 101704.56)	107911.50 (105424.25, 109603.13)	111931.31 (108372.52, 114769.14)	97723.22 (94971.30, 100046.38)	104794.31 (101883.25, 107056.75)
Cook Islands	104564.56 (102326.37, 106855.52)	84113.30 (82024.81, 86250.21)	94855.83 (93318.79, 96417.83)	160512.16 (157888.00, 163172.60)	120515.86 (118285.36, 122782.39)	139966.28 (138252.58, 141697.83)	156996.07 (152976.99, 159560.57)	118350.44 (115799.47, 120657.69)	137113.53 (134251.95, 139156.50)	153955.85 (149049.63, 157837.37)	117203.00 (113666.66, 119946.95)	135075.87 (131175.02, 137950.39)
Nauru	106487.17 (102324.47, 110910.69)	88761.47 (84723.69, 93095.01)	98041.16 (95132.11, 101086.41)	151628.73 (146453.52, 157126.44)	119490.57 (114439.74, 125178.29)	135147.55 (131662.85, 138835.28)	149177.14 (146604.25, 151525.77)	117226.78 (114861.74, 119126.41)	132767.84 (130519.71, 134559.16)	146996.45 (142574.44, 150438.79)	115230.01 (110589.56, 118598.25)	130674.25 (126690.12, 133888.97)
Niue	110283.14 (104016.66, 116861.41)	88422.78 (83123.57, 93999.41)	99360.51 (95281.99, 103579.99)	166117.75 (157524.14, 175095.01)	124164.44 (116803.14, 131914.38)	145461.31 (139790.91, 151323.30)	161987.18 (157151.35, 164342.42)	122885.49 (120301.15, 124354.72)	142688.06 (139127.84, 144355.37)	155141.32 (148096.89, 160471.38)	119962.56 (115667.02, 123627.37)	137767.82 (132562.82, 141876.16)
Palau	114550.43 (111804.44, 117383.40)	94587.96 (92115.96, 97129.66)	104789.43 (102941.37, 106675.62)	167605.75 (164903.45, 170358.23)	128776.50 (126236.06, 131363.15)	149966.60 (148104.04, 151852.77)	165695.23 (162494.08, 168262.42)	126659.50 (123317.03, 128874.07)	147959.44 (144896.25, 150081.96)	163413.02 (157814.78, 168286.33)	125383.69 (121300.30, 129221.38)	146140.75 (141518.70, 150030.65)
Tokelau	108812.70 (101216.28, 116931.08)	88894.30 (82506.47, 95727.56)	98258.43 (93331.07, 103420.34)	145058.49 (136173.48, 154459.63)	113619.77 (105556.53, 122226.46)	129790.30 (123780.72, 136040.31)	144513.53 (142504.48, 146526.01)	112545.23 (110657.92, 114146.09)	128994.36 (127359.30, 130589.39)	143402.01 (138092.70, 147815.47)	110087.14 (105346.01, 114316.12)	127278.14 (122458.14, 131124.53)
Tuvalu	111168.17 (107494.37, 115034.01)	94658.55 (91748.71, 97669.96)	102247.02 (99970.47, 104588.00)	151716.42 (148461.58, 155045.12)	119260.79 (116356.13, 122231.21)	136016.38 (133830.64, 138236.58)	151228.29 (149758.81, 152786.62)	118577.41 (117307.38, 119932.23)	135433.98 (134151.97, 136778.70)	148780.34 (142501.79, 153391.30)	116125.01 (110547.27, 121507.94)	132936.83 (127172.21, 136994.21)
**Region**												
Global	85567.97 (85564.30, 85571.63)	86562.28 (86558.71, 86565.86)	85833.63 (85831.09, 85836.17)	87059.34 (87056.36, 87062.32)	85207.31 (85204.36, 85210.25)	86061.73 (86059.64, 86063.82)	87156.29 (83729.87, 90485.24)	85316.42 (81864.21, 88918.62)	86161.16 (82876.99, 89544.09)	85024.07 (80169.04, 89338.14)	81792.99 (76592.58, 86438.17)	83349.25 (78967.07, 88000.58)

Andean Latin America	78373.74 (78330.97, 78416.54)	74585.51 (74544.36, 74626.67)	76457.94 (76428.29, 76487.61)	107388.72 (107351.97, 107425.48)	102899.10 (102863.27, 102934.95)	105214.12 (105188.46, 105239.79)	105426.10 (102389.07, 107969.38)	99032.02 (95823.81, 101430.08)	102243.70 (99300.85, 104616.28)	96435.07 (91423.65, 101012.53)	89758.91 (84998.49, 94432.53)	93164.18 (88504.04, 97608.15)
Australasia	32681.16 (32644.94, 32717.42)	29178.90 (29145.05, 29212.78)	30908.51 (30883.79, 30933.24)	36555.45 (36522.64, 36588.29)	30390.02 (30360.14, 30419.93)	33454.63 (33432.43, 33476.84)	36563.51 (34777.82, 39081.85)	30202.73 (28249.40, 32554.62)	33366.34 (31673.92, 35750.49)	35979.84 (34126.95, 38386.38)	30188.29 (28385.98, 32389.25)	33082.95 (31369.27, 35417.95)
Caribbean	92948.10 (92900.16, 92996.06)	79820.29 (79777.03, 79863.58)	86224.07 (86191.87, 86256.28)	116890.16 (116845.81, 116934.52)	104024.18 (103983.05, 104065.31)	110389.48 (110359.29, 110419.67)	111926.99 (107489.29, 116397.00)	98974.49 (94550.49, 103608.44)	105357.46 (101047.12, 109842.77)	109244.25 (103825.86, 113938.82)	96251.42 (91235.22, 100886.29)	102690.05 (97763.12, 106990.77)
Central Asia	68964.62 (68936.10, 68993.15)	70751.06 (70723.36, 70778.77)	69752.54 (69732.85, 69772.24)	85055.78 (85028.57, 85082.99)	89407.72 (89380.57, 89434.88)	87143.83 (87124.74, 87162.92)	84656.83 (83200.62, 86060.56)	88781.01 (87239.47, 90345.59)	86636.41 (85293.17, 87988.29)	84535.92 (82555.83, 86313.12)	89515.81 (87335.19, 91468.09)	86903.44 (84999.78, 88454.02)
Central Europe	97479.36 (97453.85, 97504.88)	106937.90 (106911.41, 106964.38)	102329.91 (102311.54, 102348.29)	79515.18 (79488.41, 79541.96)	90249.79 (90221.42, 90278.16)	84878.28 (84858.80, 84897.76)	82266.64 (79833.38, 84717.30)	94397.82 (91786.64, 97218.11)	88310.32 (86056.09, 90660.14)	81998.69 (78455.27, 85753.92)	91662.59 (86924.27, 95909.05)	86796.12 (82846.68, 90507.57)
Central Latin America	103711.28 (103686.11, 103736.46)	89873.12 (89849.96, 89896.29)	96676.23 (96659.16, 96693.30)	69651.78 (69636.65, 69666.92)	63534.62 (63520.38, 63548.87)	66667.20 (66656.81, 66677.59)	73940.06 (70873.92, 77489.49)	67882.06 (64540.31, 71609.37)	70943.54 (68015.31, 74393.07)	77358.69 (73174.39, 81833.31)	70797.66 (66353.37, 75706.52)	74147.92 (70073.30, 78439.54)
Central Sub-Saharan Africa	97651.04 (97597.37, 97704.76)	88127.98 (88078.94, 88177.06)	92683.24 (92647.03, 92719.48)	139160.81 (139120.94, 139200.69)	108556.58 (108524.95, 108588.22)	123486.14 (123461.19, 123511.10)	135904.95 (133163.38, 137863.60)	106466.27 (103765.05, 108722.64)	120794.10 (118175.99, 122636.10)	132347.43 (128704.17, 136165.33)	105671.73 (101860.71, 109956.75)	118582.53 (115178.09, 122173.37)
East Asia	51829.03 (51822.55, 51835.52)	46900.16 (46894.28, 46906.04)	49294.30 (49289.98, 49298.62)	54210.14 (54204.44, 54215.84)	48367.11 (48361.45, 48372.77)	51250.62 (51246.61, 51254.62)	54143.76 (52610.67, 55850.42)	49316.85 (47765.06, 51066.48)	51690.12 (50202.83, 53327.11)	57053.50 (54627.49, 60274.44)	52912.81 (49671.00, 56471.68)	54882.91 (52308.42, 58066.13)
Eastern Europe	93580.96 (93562.12, 93599.81)	98263.12 (98244.45, 98281.81)	95911.86 (95898.70, 95925.03)	96678.61 (96657.80, 96699.41)	108554.67 (108532.91, 108576.44)	102804.21 (102789.19, 102819.23)	97161.84 (95730.22, 98793.44)	108572.57 (106116.57, 110781.80)	103064.15 (101452.58, 104829.70)	96257.61 (89920.30, 101616.59)	104732.72 (97507.66, 110436.24)	100582.75 (94279.79, 105413.50)

Eastern Sub-Saharan Africa	128289.13 (128258.91, 128319.37)	129271.46 (129242.17, 129300.75)	128815.87 (128794.86, 128836.89)	134631.67 (134610.65, 134652.70)	116051.93 (116033.34, 116070.52)	125169.19 (125155.23, 125183.16)	131961.07 (129458.43, 134583.63)	115537.04 (113127.40, 118040.59)	123574.19 (121477.94, 125896.27)	141801.47 (138251.03, 145507.26)	130223.29 (126371.65, 134503.35)	135790.04 (132441.50, 139722.92)
High-income Asia Pacific	14383.21 (14375.30, 14391.13)	15120.65 (15112.84, 15128.46)	14699.08 (14693.56, 14704.60)	12603.89 (12597.07, 12610.71)	14065.10 (14057.84, 14072.37)	13288.22 (13283.25, 13293.19)	11615.38 (10149.65, 14002.87)	13451.82 (11780.68, 16325.38)	12515.23 (11134.92, 14920.83)	14329.67 (11239.51, 17685.91)	15175.92 (12189.60, 19358.92)	14726.71 (11767.96, 18040.19)
High-income North America	45410.07 (45398.43, 45421.71)	44609.56 (44598.24, 44620.88)	45072.30 (45064.17, 45080.42)	49173.56 (49162.56, 49184.57)	47794.97 (47784.19, 47805.74)	48527.18 (48519.47, 48534.88)	47790.95 (45954.80, 49327.08)	48463.36 (46607.52, 50154.99)	48163.31 (46465.18, 49634.82)	48519.84 (46587.82, 50680.81)	49036.46 (46874.87, 51835.31)	48792.68 (46979.39, 51049.30)
North Africa and Middle East	78350.25 (78335.65, 78364.85)	77447.34 (77432.92, 77461.76)	77896.63 (77886.39, 77906.88)	111159.81 (111147.25, 111172.37)	111075.12 (111062.28, 111087.96)	111112.47 (111103.50, 111121.45)	110600.36 (108908.03, 112179.33)	111127.97 (109313.08, 112850.18)	110852.18 (109232.23, 112357.58)	108320.69 (105422.84, 111034.21)	111189.76 (108108.77, 114074.75)	109711.19 (106883.40, 112201.26)
Oceania	130072.25 (129911.45, 130233.38)	103062.28 (102915.45, 103209.41)	117094.96 (116985.82, 117204.25)	173865.90 (173747.89, 173984.03)	130112.62 (130007.38, 130217.97)	152728.21 (152648.91, 152807.57)	170837.02 (167391.43, 173067.43)	128402.63 (126351.50, 130337.86)	150115.21 (147841.70, 151877.43)	159711.62 (154681.73, 163586.53)	122144.39 (118297.02, 125983.42)	140770.67 (136163.59, 144100.92)
South Asia	159315.41 (159303.36, 159327.46)	187434.62 (187421.15, 187448.09)	172743.41 (172734.41, 172752.41)	124351.43 (124343.81, 124359.06)	135306.49 (135298.53, 135314.46)	129771.79 (129766.28, 129777.30)	122587.08 (118309.52, 126302.61)	131074.82 (126373.65, 135351.52)	126803.24 (122680.74, 130512.93)	116442.44 (111482.56, 120553.20)	126294.80 (120414.16, 130588.51)	121319.17 (116171.04, 125418.75)
Southeast Asia	76383.66 (76369.66, 76397.66)	61704.64 (61693.14, 61716.14)	68651.48 (68642.56, 68660.40)	77888.80 (77878.39, 77899.21)	63935.44 (63926.46, 63944.43)	70616.16 (70609.37, 70622.96)	74571.88 (72019.79, 77101.17)	61715.61 (59284.62, 64453.66)	67756.42 (65430.30, 70281.59)	75749.12 (71466.36, 80032.33)	66352.32 (62024.37, 71485.41)	70846.70 (66889.16, 75635.84)
Southern Latin America	50058.74 (50030.28, 50087.22)	38849.66 (38825.36, 38873.96)	44253.74 (44235.19, 44272.31)	57009.08 (56982.52, 57035.66)	42450.36 (42427.81, 42472.91)	49554.97 (49537.62, 49572.33)	58323.21 (56354.18, 60725.78)	46217.55 (44046.01, 49107.46)	52163.96 (50341.84, 54732.90)	58183.58 (55763.35, 60828.83)	46425.07 (43788.79, 50094.55)	52268.30 (49976.69, 55201.90)
Southern Sub-Saharan Africa	121874.37 (121821.88, 121926.88)	104989.26 (104945.65, 105032.89)	113000.81 (112967.21, 113034.42)	115577.20 (115538.64, 115615.78)	98267.08 (98235.06, 98299.12)	106561.41 (106536.81, 106586.02)	111827.10 (108389.22, 115164.04)	97020.04 (93776.94, 100243.98)	104052.10 (100784.18, 107196.87)	112744.72 (108294.24, 116812.55)	98828.38 (94954.36, 102989.35)	105448.05 (101479.97, 109347.99)
Tropical Latin America	99540.12 (99514.73, 99565.51)	94472.67 (94449.23, 94496.12)	96865.99 (96848.79, 96883.19)	85188.81 (85170.82, 85206.81)	87157.59 (87139.79, 87175.40)	86128.97 (86116.35, 86141.59)	90679.56 (87704.20, 94047.56)	92492.70 (89124.27, 96385.59)	91553.99 (88689.73, 94904.48)	87309.24 (83342.28, 92446.46)	87660.89 (83394.40, 92851.81)	87490.00 (83601.92, 92410.82)

Western Europe	38087.33 (38077.71, 38096.95)	33635.91 (33627.01, 33644.81)	35912.50 (35905.97, 35919.05)	38409.21 (38399.80, 38418.62)	34223.36 (34214.46, 34232.26)	36345.48 (36339.01, 36351.96)	38011.32 (36225.38, 39259.15)	34090.84 (32323.78, 35516.19)	36073.38 (34376.20, 37307.95)	38361.06 (35848.57, 40635.33)	35140.44 (32912.01, 37615.37)	36776.92 (34466.97, 39134.55)
Western Sub-Saharan Africa	129196.74 (129168.64, 129224.85)	133790.15 (133762.26, 133818.05)	131604.05 (131584.33, 131623.78)	144098.51 (144078.32, 144118.69)	135866.21 (135847.59, 135884.83)	139732.85 (139719.16, 139746.54)	142455.83 (138800.11, 144974.49)	133738.16 (130484.66, 136125.38)	137852.18 (134608.15, 140150.79)	139672.38 (134327.03, 144769.09)	133634.76 (128715.15, 138253.07)	136396.13 (131459.51, 141081.40)
High SDI	40089.49 (40083.05, 40095.94)	38387.65 (38381.45, 38393.86)	39253.13 (39248.67, 39257.60)	44477.85 (44471.45, 44484.26)	41739.84 (41733.57, 41746.10)	43109.82 (43105.34, 43114.30)	42202.77 (40161.61, 43946.89)	39568.55 (37177.68, 41608.82)	40892.55 (38668.90, 42674.24)	41841.12 (39742.53, 43746.52)	39396.40 (36986.34, 41472.53)	40619.42 (38550.56, 42595.25)
High-middle SDI	66817.02 (66810.09, 66823.95)	65963.93 (65957.23, 65970.63)	66216.14 (66211.36, 66220.92)	68636.85 (68630.32, 68643.38)	67189.55 (67182.98, 67196.12)	67807.72 (67803.10, 67812.34)	67614.21 (65339.77, 69312.08)	67640.96 (65943.33, 69175.38)	67525.18 (65843.53, 68938.30)	62877.35 (59289.54, 67480.47)	62968.40 (59316.36, 67793.49)	62828.53 (59378.18, 67415.07)
Middle SDI	77443.63 (77436.88, 77450.38)	72936.42 (72930.17, 72942.68)	75085.66 (75081.10, 75090.23)	79346.24 (79341.01, 79351.47)	75194.53 (75189.44, 75199.61)	77220.15 (77216.51, 77223.79)	78302.65 (76232.26, 79765.42)	74321.14 (72245.55, 75832.21)	76257.39 (74281.01, 77554.97)	77467.29 (74572.05, 80596.44)	73692.17 (70763.36, 77010.42)	75532.80 (72655.68, 78530.63)
Low-middle SDI	136134.16 (136123.15, 136145.17)	148269.10 (148257.70, 148280.51)	142032.31 (142024.39, 142040.22)	116108.78 (116101.26, 116116.30)	119118.96 (119111.48, 119126.44)	117600.90 (117595.60, 117606.19)	116500.17 (113574.46, 118749.29)	119031.90 (115929.25, 121491.71)	117767.81 (114915.88, 119983.25)	111620.10 (104272.16, 117078.20)	114345.06 (107430.76, 119953.40)	112988.00 (106351.20, 118294.41)
Low SDI	141665.52 (141647.82, 141683.22)	153426.69 (153408.45, 153444.93)	147493.70 (147481.00, 147506.39)	140749.10 (140737.12, 140761.08)	134464.17 (134452.71, 134475.63)	137592.10 (137583.82, 137600.37)	139444.12 (137674.41, 141407.10)	133182.91 (131108.54, 135181.85)	136297.78 (134611.39, 138012.04)	143310.69 (139707.01, 147196.56)	137291.19 (133602.58, 141254.67)	140288.48 (136862.57, 144076.65)

Abbreviations: ASIRs, age-standardized incidence rates; UIs, uncertainty intervals.

Table S2. EAPCs of ASIRs per 100,000 for diarrheal diseases for males, females, and both genders combined at the national, regional, and global levels from 1990 to 2019 and from 2020 to 2040

Location	EAPCs (%)					
	1990−2019			2020−2040		
	Male	Female	Both genders combined	Male	Female	Both genders combined
**Nation**						
Afghanistan	1.48 (1.36, 1.60)	1.68 (1.56, 1.79)	1.57 (1.45, 1.68)	0.07 (0.03, 0.11)	0.18 (0.16, 0.19)	0.12 (0.10, 0.15)
Albania	−0.52 (−0.56, −0.48)	−0.47 (−0.52, −0.41)	−0.48 (−0.52, −0.44)	−0.23 (−0.28, −0.19)	−0.50 (−0.56, −0.44)	−0.38 (−0.43, −0.34)
Algeria	0.90 (0.81, 0.99)	0.92 (0.85, 0.99)	0.91 (0.83, 0.99)	0.05 (0.04, 0.07)	0.03 (0.02, 0.04)	0.04 (0.03, 0.05)
American Samoa	1.25 (1.17, 1.34)	1.02 (0.94, 1.11)	1.13 (1.05, 1.22)	−0.13 (−0.21, −0.04)	−0.12 (−0.16, −0.07)	−0.12 (−0.19, −0.05)
Andorra	−0.09 (−0.11, −0.07)	−0.09 (−0.12, −0.07)	−0.10 (−0.12, −0.09)	0.06 (0.04, 0.08)	0.04 (0.01, 0.08)	0.06 (0.04, 0.09)
Angola	0.83 (0.75, 0.91)	0.03 (−0.02, 0.08)	0.42 (0.37, 0.48)	−0.57 (−0.68, −0.47)	−0.37 (−0.46, −0.29)	−0.47 (−0.57, −0.38)
Antigua and Barbuda	0.92 (0.87, 0.96)	1.17 (1.10, 1.24)	1.06 (1.00, 1.12)	−0.29 (−0.41, −0.18)	−0.34 (−0.50, −0.18)	−0.31 (−0.45, −0.17)
Argentina	0.64 (0.54, 0.74)	0.44 (0.30, 0.58)	0.56 (0.44, 0.68)	0.16 (−0.28, 0.59)	0.21 (−0.37, 0.80)	0.19 (−0.32, 0.69)
Armenia	1.29 (1.16, 1.42)	1.32 (1.20, 1.44)	1.31 (1.19, 1.43)	0.07 (0.05, 0.10)	0.02 (0.00, 0.04)	0.04 (0.02, 0.06)
Australia	0.43 (0.34, 0.51)	0.16 (0.03, 0.29)	0.31 (0.21, 0.41)	0.49 (0.24, 0.75)	0.61 (0.26, 0.95)	0.55 (0.25, 0.85)
Austria	0.02 (−0.04, 0.07)	0.14 (0.08, 0.20)	0.07 (0.02, 0.12)	1.12 (0.72, 1.52)	1.10 (0.61, 1.60)	1.11 (0.67, 1.55)
Azerbaijan	1.49 (1.34, 1.63)	1.50 (1.35, 1.66)	1.49 (1.35, 1.64)	−0.12 (−0.16, −0.08)	−0.27 (−0.34, −0.19)	−0.20 (−0.26, −0.14)
Bahrain	1.42 (1.28, 1.57)	1.50 (1.37, 1.62)	1.46 (1.33, 1.60)	0.15 (0.09, 0.21)	0.05 (0.01, 0.10)	0.12 (0.07, 0.17)

Bangladesh	−0.38 (−0.54, −0.22)	−0.68 (−0.82, −0.55)	−0.52 (−0.67, −0.37)	−0.46 (−0.51, −0.40)	−0.66 (−0.76, −0.56)	−0.56 (−0.63, −0.49)
Barbados	1.09 (1.00, 1.18)	1.51 (1.40, 1.62)	1.32 (1.22, 1.42)	0.02 (−0.03, 0.08)	0.04 (−0.04, 0.11)	0.03 (−0.03, 0.10)
Belarus	0.20 (0.12, 0.28)	0.46 (0.37, 0.55)	0.34 (0.26, 0.42)	0.08 (0.07, 0.10)	0.05 (0.01, 0.09)	0.06 (0.03, 0.09)
Belgium	0.44 (0.30, 0.58)	0.55 (0.36, 0.74)	0.48 (0.32, 0.65)	−0.21 (−0.27, −0.15)	−0.19 (−0.26, −0.12)	−0.20 (−0.26, −0.14)
Belize	0.67 (0.61, 0.74)	0.87 (0.79, 0.96)	0.76 (0.69, 0.83)	−0.08 (−0.14, −0.03)	−0.12 (−0.16, −0.07)	−0.11 (−0.16, −0.05)
Benin	0.43 (0.37, 0.50)	0.23 (0.14, 0.31)	0.33 (0.26, 0.41)	−0.19 (−0.35, −0.04)	−0.14 (−0.38, 0.10)	−0.17 (−0.37, 0.03)
Bermuda	0.93 (0.83, 1.04)	1.37 (1.21, 1.53)	1.17 (1.04, 1.30)	0.03 (−0.05, 0.11)	0.00 (−0.08, 0.08)	0.02 (−0.06, 0.10)
Bhutan	0.26 (0.18, 0.35)	−0.75 (−0.89, −0.60)	−0.25 (−0.37, −0.14)	−0.05 (−0.12, 0.02)	−0.08 (−0.14, −0.01)	−0.06 (−0.12, 0.01)
Bosnia and Herzegovina	−0.94 (−1.01, −0.88)	−0.86 (−0.95, −0.76)	−0.90 (−0.98, −0.82)	−0.14 (−0.19, −0.10)	−0.10 (−0.16, −0.03)	−0.12 (−0.18, −0.06)
Botswana	−0.31 (−0.44, −0.19)	0.05 (−0.08, 0.18)	−0.14 (−0.26, −0.01)	−0.48 (−0.55, −0.42)	−0.17 (−0.21, −0.13)	−0.31 (−0.36, −0.27)
Brazil	−0.52 (−0.58, −0.46)	−0.30 (−0.33, −0.27)	−0.41 (−0.45, −0.37)	−0.17 (−0.22, −0.13)	−0.23 (−0.28, −0.18)	−0.20 (−0.25, −0.16)
Bulgaria	−0.61 (−0.66, −0.57)	−0.46 (−0.51, −0.41)	−0.54 (−0.58, −0.50)	−4.13 (−5.19, −3.06)	−3.93 (−4.89, −2.95)	−4.02 (−5.03, −3.00)
Burkina Faso	0.73 (0.66, 0.80)	0.17 (0.09, 0.25)	0.43 (0.36, 0.50)	−0.19 (−0.21, −0.17)	−0.11 (−0.12, −0.10)	−0.14 (−0.16, −0.13)
Burundi	0.60 (0.51, 0.70)	0.02 (−0.06, 0.10)	0.33 (0.25, 0.42)	−0.20 (−0.24, −0.17)	−0.17 (−0.21, −0.14)	−0.21 (−0.24, −0.17)

Cambodia	−0.42 (−0.51, −0.33)	−0.71 (−0.81, −0.61)	−0.56 (−0.66, −0.47)	0.12 (0.07, 0.17)	0.28 (0.21, 0.34)	0.22 (0.16, 0.27)
Cameroon	0.68 (0.59, 0.77)	0.33 (0.23, 0.43)	0.51 (0.42, 0.60)	−0.44 (−0.52, −0.36)	−0.43 (−0.50, −0.37)	−0.44 (−0.52, −0.37)
Canada	0.50 (0.33, 0.67)	0.62 (0.49, 0.75)	0.55 (0.42, 0.68)	−0.06 (−0.07, −0.05)	0.05 (0.03, 0.07)	−0.01 (−0.02, 0.00)
Central African Republic	1.38 (1.30, 1.47)	0.79 (0.75, 0.84)	1.10 (1.03, 1.16)	0.38 (0.32, 0.44)	0.54 (0.47, 0.61)	0.43 (0.37, 0.50)
Chad	0.89 (0.80, 0.98)	0.64 (0.55, 0.72)	0.77 (0.69, 0.86)	−0.52 (−0.54, −0.50)	−0.52 (−0.54, −0.50)	−0.54 (−0.55, −0.52)
Chile	−0.37 (−0.49, −0.24)	0.16 (0.01, 0.31)	−0.11 (−0.23, 0.00)	−0.06 (−0.17, 0.05)	−0.04 (−0.20, 0.12)	−0.05 (−0.18, 0.08)
China	−0.18 (−0.41, 0.05)	−0.12 (−0.32, 0.07)	−0.15 (−0.36, 0.07)	0.35 (0.31, 0.39)	0.50 (0.45, 0.56)	0.41 (0.37, 0.46)
Colombia	−0.93 (−0.98, −0.88)	−0.90 (−0.96, −0.84)	−0.90 (−0.96, −0.85)	0.24 (0.21, 0.27)	0.32 (0.29, 0.35)	0.28 (0.25, 0.31)
Comoros	0.34 (0.26, 0.43)	−0.22 (−0.30, −0.14)	0.06 (−0.02, 0.14)	−0.09 (−0.14, −0.04)	−0.15 (−0.19, −0.10)	−0.12 (−0.16, −0.07)
Costa Rica	−0.59 (−0.72, −0.45)	−0.81 (−0.95, −0.67)	−0.69 (−0.83, −0.56)	0.09 (0.02, 0.15)	0.12 (0.07, 0.17)	0.11 (0.05, 0.16)
Croatia	−0.95 (−1.01, −0.89)	−0.67 (−0.72, −0.63)	−0.81 (−0.86, −0.76)	−0.04 (−0.07, 0.00)	−0.18 (−0.27, −0.09)	−0.11 (−0.17, −0.05)
Cuba	0.69 (0.59, 0.78)	0.65 (0.55, 0.76)	0.68 (0.58, 0.78)	0.02 (0.00, 0.04)	0.13 (0.11, 0.16)	0.07 (0.05, 0.09)
Cyprus	−0.07 (−0.17, 0.02)	−0.18 (−0.25, −0.11)	−0.14 (−0.21, −0.06)	−0.09 (−0.19, 0.02)	−0.03 (−0.15, 0.08)	−0.07 (−0.17, 0.04)
Congo	1.43 (1.28, 1.57)	0.69 (0.60, 0.77)	1.09 (0.98, 1.21)	−0.55 (−0.61, −0.49)	−0.42 (−0.48, −0.36)	−0.49 (−0.55, −0.44)

Denmark	0.26 (0.15, 0.37)	0.43 (0.32, 0.53)	0.34 (0.23, 0.45)	−0.14 (−0.20, −0.09)	−0.13 (−0.20, −0.07)	−0.14 (−0.20, −0.08)
Djibouti	0.10 (−0.05, 0.25)	−0.37 (−0.51, −0.23)	−0.10 (−0.25, 0.04)	0.09 (−0.01, 0.19)	0.15 (0.08, 0.22)	0.11 (0.02, 0.19)
Dominica	0.77 (0.69, 0.86)	1.06 (0.96, 1.15)	0.93 (0.84, 1.03)	0.00 (−0.05, 0.05)	0.02 (−0.03, 0.08)	0.01 (−0.04, 0.06)
Dominican Republic	0.60 (0.51, 0.68)	0.81 (0.73, 0.90)	0.71 (0.62, 0.79)	−0.15 (−0.17, −0.14)	−0.16 (−0.19, −0.13)	−0.16 (−0.18, −0.14)
Ecuador	0.43 (0.29, 0.57)	0.74 (0.61, 0.87)	0.59 (0.45, 0.72)	0.11 (0.09, 0.13)	−0.01 (−0.03, 0.01)	0.05 (0.03, 0.07)
Egypt	0.81 (0.73, 0.89)	1.06 (1.00, 1.11)	0.93 (0.86, 1.00)	0.02 (−0.04, 0.07)	−0.01 (−0.10, 0.07)	0.00 (−0.07, 0.07)
El Salvador	−1.38 (−1.51, −1.25)	−0.96 (−1.06, −0.87)	−1.18 (−1.29, −1.06)	−0.03 (−0.12, 0.07)	0.05 (0.00, 0.10)	0.02 (−0.05, 0.08)
Equatorial Guinea	−0.01 (−0.13, 0.11)	−0.84 (−1.00, −0.68)	−0.43 (−0.57, −0.28)	−0.35 (−0.37, −0.33)	−0.50 (−0.54, −0.47)	−0.39 (−0.42, −0.36)
Eritrea	0.68 (0.56, 0.80)	−0.30 (−0.45, −0.15)	0.21 (0.07, 0.34)	−0.09 (−0.15, −0.03)	0.08 (0.03, 0.12)	0.00 (−0.05, 0.06)
Estonia	0.31 (0.23, 0.40)	1.15 (1.02, 1.27)	0.76 (0.65, 0.86)	0.02 (−0.04, 0.08)	−0.10 (−0.16, −0.04)	−0.05 (−0.11, 0.01)
Ethiopia	−0.40 (−0.47, −0.33)	−1.15 (−1.23, −1.08)	−0.78 (−0.85, −0.71)	0.07 (0.06, 0.08)	0.11 (0.10, 0.12)	0.09 (0.08, 0.10)
Fiji	0.77 (0.66, 0.88)	0.57 (0.45, 0.70)	0.69 (0.58, 0.80)	−0.21 (−0.24, −0.18)	−0.21 (−0.25, −0.17)	−0.22 (−0.25, −0.19)
Finland	−0.24 (−0.27, −0.20)	−0.27 (−0.31, −0.23)	−0.26 (−0.30, −0.23)	−0.18 (−0.31, −0.05)	−0.16 (−0.32, 0.00)	−0.17 (−0.32, −0.03)
France	−0.10 (−0.17, −0.03)	−0.07 (−0.14, 0.01)	−0.09 (−0.16, −0.02)	0.02 (−0.03, 0.06)	0.02 (−0.02, 0.07)	0.02 (−0.02, 0.06)

Gabon	1.17 (1.03, 1.30)	0.43 (0.32, 0.55)	0.84 (0.71, 0.96)	−0.69 (−0.73, −0.65)	−0.48 (−0.53, −0.43)	−0.62 (−0.65, −0.58)
Germany	0.54 (0.46, 0.62)	0.63 (0.53, 0.73)	0.58 (0.49, 0.67)	0.00 (−0.05, 0.06)	−0.02 (−0.08, 0.04)	−0.01 (−0.06, 0.05)
Ghana	0.34 (0.21, 0.47)	0.22 (0.10, 0.33)	0.27 (0.15, 0.38)	−0.18 (−0.20, −0.16)	−0.18 (−0.20, −0.17)	−0.18 (−0.20, −0.16)
Greece	−0.24 (−0.27, −0.20)	−0.11 (−0.15, −0.06)	−0.18 (−0.21, −0.14)	−0.07 (−0.13, 0.00)	−0.13 (−0.20, −0.07)	−0.10 (−0.16, −0.03)
Greenland	0.00 (−0.13, 0.12)	0.08 (0.00, 0.16)	0.03 (−0.07, 0.13)	−0.01 (−0.07, 0.04)	−0.07 (−0.11, −0.03)	−0.05 (−0.09, 0.00)
Grenada	1.01 (0.92, 1.09)	1.26 (1.15, 1.36)	1.16 (1.07, 1.25)	−0.11 (−0.17, −0.06)	−0.12 (−0.17, −0.07)	−0.12 (−0.17, −0.07)
Guam	1.19 (1.10, 1.28)	0.96 (0.88, 1.05)	1.08 (0.99, 1.17)	−0.04 (−0.06, −0.01)	−0.03 (−0.06, 0.00)	−0.04 (−0.07, −0.02)
Guatemala	−2.30 (−2.37, −2.23)	−2.36 (−2.43, −2.29)	−2.33 (−2.40, −2.26)	−0.20 (−0.24, −0.15)	−0.22 (−0.26, −0.17)	−0.20 (−0.24, −0.16)
Guinea	0.56 (0.46, 0.66)	0.18 (0.11, 0.26)	0.37 (0.29, 0.45)	−0.36 (−0.39, −0.32)	−0.27 (−0.31, −0.23)	−0.33 (−0.37, −0.29)
Guinea−Bissau	0.67 (0.58, 0.77)	0.21 (0.13, 0.28)	0.43 (0.35, 0.51)	0.16 (0.12, 0.19)	0.10 (0.07, 0.13)	0.12 (0.09, 0.16)
Guyana	0.15 (0.05, 0.25)	0.33 (0.21, 0.46)	0.24 (0.13, 0.35)	−0.11 (−0.14, −0.08)	−0.24 (−0.28, −0.20)	−0.18 (−0.21, −0.15)
Haiti	0.30 (0.24, 0.36)	0.32 (0.27, 0.36)	0.31 (0.25, 0.36)	−0.13 (−0.18, −0.09)	−0.14 (−0.21, −0.07)	−0.13 (−0.19, −0.07)
Honduras	−1.23 (−1.35, −1.12)	−1.18 (−1.27, −1.08)	−1.21 (−1.31, −1.10)	−0.06 (−0.10, −0.02)	−0.15 (−0.18, −0.12)	−0.10 (−0.13, −0.06)
Hungary	−0.31 (−0.37, −0.24)	−0.25 (−0.32, −0.19)	−0.28 (−0.35, −0.22)	−0.60 (−0.83, −0.36)	−1.03 (−1.37, −0.68)	−0.81 (−1.09, −0.53)

Iceland	0.03 (−0.01, 0.07)	0.06 (0.00, 0.12)	0.04 (−0.01, 0.08)	−0.07 (−0.09, −0.06)	−0.07 (−0.09, −0.05)	−0.07 (−0.09, −0.06)
India	−1.46 (−1.78, −1.14)	−1.78 (−2.12, −1.45)	−1.62 (−1.94, −1.30)	−0.52 (−0.66, −0.37)	−0.46 (−0.61, −0.31)	−0.49 (−0.64, −0.34)
Indonesia	−0.16 (−0.47, 0.16)	−0.03 (−0.26, 0.21)	−0.10 (−0.38, 0.18)	−0.12 (−0.26, 0.02)	−0.13 (−0.20, −0.07)	−0.13 (−0.23, −0.03)
Iraq	0.95 (0.90, 1.00)	1.00 (0.97, 1.04)	0.98 (0.94, 1.02)	−0.03 (−0.04, −0.01)	−0.01 (−0.03, 0.00)	−0.02 (−0.04, 0.00)
Ireland	0.01 (−0.04, 0.05)	0.24 (0.18, 0.29)	0.12 (0.07, 0.16)	0.11 (0.02, 0.19)	0.11 (0.03, 0.19)	0.11 (0.02, 0.19)
Israel	0.47 (0.42, 0.53)	0.46 (0.40, 0.52)	0.46 (0.41, 0.52)	−0.02 (−0.10, 0.06)	0.04 (−0.06, 0.15)	0.01 (−0.08, 0.10)
Italy	−0.35 (−0.43, −0.26)	−0.16 (−0.23, −0.10)	−0.26 (−0.32, −0.20)	0.43 (0.32, 0.54)	0.21 (0.08, 0.33)	0.32 (0.21, 0.43)
Jamaica	0.45 (0.31, 0.58)	0.70 (0.62, 0.77)	0.57 (0.47, 0.68)	−0.07 (−0.13, −0.02)	0.03 (−0.03, 0.09)	−0.02 (−0.08, 0.04)
Japan	−1.90 (−2.24, −1.56)	−1.03 (−1.24, −0.82)	−1.42 (−1.68, −1.17)	2.02 (1.35, 2.69)	1.57 (1.07, 2.07)	1.79 (1.21, 2.38)
Jordan	1.34 (1.21, 1.47)	1.38 (1.26, 1.49)	1.36 (1.23, 1.48)	0.00 (−0.02, 0.03)	−0.03 (−0.07, 0.00)	−0.02 (−0.04, 0.01)
Kazakhstan	1.17 (1.00, 1.34)	1.29 (1.13, 1.46)	1.24 (1.07, 1.41)	0.01 (−0.02, 0.03)	0.02 (0.00, 0.03)	0.01 (−0.01, 0.03)
Kenya	−0.14 (−0.27, −0.02)	−0.56 (−0.71, −0.41)	−0.34 (−0.47, −0.20)	−0.39 (−0.57, −0.20)	−0.25 (−0.43, −0.06)	−0.33 (−0.51, −0.14)
Kiribati	0.78 (0.71, 0.86)	0.54 (0.46, 0.62)	0.66 (0.58, 0.74)	−0.34 (−0.41, −0.27)	−0.19 (−0.26, −0.11)	−0.28 (−0.35, −0.21)
Kuwait	1.27 (1.18, 1.37)	1.17 (1.10, 1.24)	1.22 (1.13, 1.31)	0.01 (0.00, 0.03)	0.00 (−0.02, 0.01)	0.01 (−0.01, 0.02)
Kyrgyzstan	1.33 (1.19, 1.47)	1.41 (1.25, 1.57)	1.38 (1.23, 1.53)	0.00 (−0.02, 0.03)	0.04 (0.03, 0.05)	0.02 (0.01, 0.04)

Latvia	0.15 (0.06, 0.24)	0.03 (−0.04, 0.10)	0.08 (0.01, 0.16)	−0.59 (−1.09, −0.09)	−0.61 (−1.01, −0.20)	−0.60 (−1.05, −0.16)
Lebanon	1.08 (0.90, 1.26)	1.13 (0.98, 1.29)	1.11 (0.94, 1.28)	−0.02 (−0.05, 0.01)	−0.04 (−0.09, 0.01)	−0.03 (−0.06, 0.01)
Lesotho	0.08 (−0.07, 0.23)	0.30 (0.14, 0.45)	0.16 (0.01, 0.32)	−0.28 (−0.29, −0.26)	−0.09 (−0.10, −0.07)	−0.20 (−0.21, −0.18)
Liberia	1.16 (0.99, 1.33)	0.77 (0.61, 0.92)	0.97 (0.81, 1.13)	0.04 (0.01, 0.07)	−0.04 (−0.09, 0.01)	−0.01 (−0.04, 0.03)
Libya	1.44 (1.31, 1.57)	1.66 (1.55, 1.78)	1.55 (1.43, 1.66)	−0.09 (−0.28, 0.10)	−0.06 (−0.26, 0.15)	−0.07 (−0.27, 0.12)
Lithuania	−0.03 (−0.08, 0.02)	0.47 (0.37, 0.57)	0.24 (0.16, 0.31)	−0.15 (−0.25, −0.06)	−0.09 (−0.19, 0.00)	−0.13 (−0.22, −0.03)
Luxembourg	0.25 (0.17, 0.33)	0.16 (0.01, 0.32)	0.20 (0.10, 0.31)	−0.09 (−0.19, 0.01)	−0.14 (−0.27, −0.01)	−0.12 (−0.23, −0.01)
Madagascar	0.61 (0.51, 0.72)	0.17 (0.07, 0.27)	0.41 (0.31, 0.51)	−0.47 (−0.54, −0.41)	−0.43 (−0.51, −0.35)	−0.45 (−0.52, −0.38)
Malawi	0.49 (0.42, 0.57)	−0.16 (−0.23, −0.08)	0.17 (0.10, 0.24)	−0.28 (−0.33, −0.22)	−0.20 (−0.23, −0.16)	−0.24 (−0.28, −0.20)
Malaysia	1.11 (1.08, 1.14)	1.08 (0.98, 1.17)	1.11 (1.05, 1.17)	−0.21 (−0.25, −0.16)	−0.08 (−0.13, −0.03)	−0.15 (−0.20, −0.11)
Maldives	0.23 (0.05, 0.42)	0.25 (−0.01, 0.51)	0.22 (0.00, 0.45)	−0.09 (−0.13, −0.06)	0.19 (0.13, 0.24)	0.03 (−0.01, 0.06)
Mali	0.70 (0.61, 0.79)	0.11 (0.04, 0.17)	0.41 (0.34, 0.49)	−0.21 (−0.26, −0.16)	−0.13 (−0.18, −0.09)	−0.18 (−0.23, −0.13)
Malta	−0.34 (−0.49, −0.20)	−0.20 (−0.33, −0.06)	−0.27 (−0.41, −0.14)	1.20 (0.83, 1.58)	0.99 (0.70, 1.27)	1.09 (0.76, 1.42)
Marshall Islands	0.58 (0.45, 0.71)	0.17 (0.06, 0.28)	0.40 (0.28, 0.51)	−0.33 (−0.36, −0.30)	−0.40 (−0.44, −0.36)	−0.37 (−0.40, −0.33)

Mauritania	1.03 (0.93, 1.13)	0.66 (0.55, 0.77)	0.85 (0.74, 0.95)	−0.43 (−0.51, −0.36)	−0.30 (−0.36, −0.23)	−0.37 (−0.44, −0.30)
Mauritius	0.21 (0.04, 0.39)	0.48 (0.36, 0.61)	0.35 (0.19, 0.50)	0.46 (0.34, 0.58)	0.59 (0.47, 0.72)	0.54 (0.42, 0.66)
Mexico	−2.36 (−2.50, −2.22)	−1.88 (−2.09, −1.68)	−2.12 (−2.29, −1.96)	1.93 (1.60, 2.26)	1.94 (1.58, 2.32)	1.93 (1.59, 2.28)
Mongolia	0.63 (0.52, 0.75)	0.60 (0.49, 0.71)	0.62 (0.51, 0.73)	−0.07 (−0.10, −0.03)	−0.10 (−0.15, −0.05)	−0.09 (−0.13, −0.04)
Montenegro	−0.94 (−0.99, −0.89)	−0.64 (−0.68, −0.61)	−0.79 (−0.83, −0.75)	0.18 (0.13, 0.23)	0.18 (0.12, 0.24)	0.17 (0.12, 0.23)
Morocco	0.83 (0.75, 0.90)	0.68 (0.60, 0.75)	0.75 (0.69, 0.82)	0.02 (−0.01, 0.05)	0.00 (−0.02, 0.03)	0.01 (−0.01, 0.04)
Mozambique	0.30 (0.20, 0.40)	−0.52 (−0.68, −0.36)	−0.10 (−0.23, 0.02)	−0.07 (−0.10, −0.03)	0.01 (−0.03, 0.05)	−0.04 (−0.08, −0.01)
Myanmar	−0.60 (−0.73, −0.46)	−0.94 (−1.11, −0.76)	−0.78 (−0.93, −0.62)	−0.14 (−0.20, −0.09)	−0.33 (−0.43, −0.22)	−0.25 (−0.33, −0.18)
Namibia	0.03 (−0.17, 0.22)	−0.26 (−0.45, −0.06)	−0.11 (−0.31, 0.09)	−0.55 (−0.58, −0.52)	−0.11 (−0.13, −0.09)	−0.32 (−0.34, −0.30)
Nepal	−0.44 (−0.58, −0.30)	−0.71 (−0.89, −0.53)	−0.58 (−0.74, −0.41)	−0.16 (−0.24, −0.08)	−0.16 (−0.23, −0.08)	−0.15 (−0.23, −0.08)
Netherlands	0.29 (0.24, 0.34)	0.36 (0.28, 0.43)	0.32 (0.25, 0.38)	−0.25 (−0.35, −0.15)	−0.32 (−0.46, −0.19)	−0.28 (−0.40, −0.17)
New Zealand	0.20 (0.18, 0.22)	0.25 (0.21, 0.29)	0.23 (0.20, 0.25)	−0.26 (−0.32, −0.20)	−0.28 (−0.35, −0.22)	−0.28 (−0.34, −0.22)
Nicaragua	−0.84 (−0.94, −0.74)	−0.80 (−0.89, −0.70)	−0.81 (−0.91, −0.71)	0.18 (0.08, 0.28)	0.05 (−0.01, 0.11)	0.12 (0.04, 0.20)
Niger	0.78 (0.67, 0.90)	0.22 (0.11, 0.33)	0.50 (0.39, 0.60)	−0.35 (−0.37, −0.32)	−0.23 (−0.26, −0.19)	−0.29 (−0.32, −0.26)

Nigeria	−0.11 (−0.19, −0.02)	−0.58 (−0.65, −0.51)	−0.34 (−0.41, −0.27)	0.26 (0.21, 0.31)	0.27 (0.21, 0.32)	0.26 (0.21, 0.31)
Northern Mariana Islands	1.47 (1.39, 1.55)	1.22 (1.15, 1.29)	1.33 (1.26, 1.41)	−0.08 (−0.12, −0.04)	−0.15 (−0.18, −0.12)	−0.11 (−0.14, −0.08)
Norway	0.24 (0.19, 0.30)	0.22 (0.15, 0.29)	0.23 (0.17, 0.28)	−0.08 (−0.12, −0.03)	−0.08 (−0.13, −0.03)	−0.08 (−0.12, −0.03)
Oman	1.45 (1.34, 1.56)	1.53 (1.38, 1.68)	1.50 (1.37, 1.63)	−0.11 (−0.29, 0.06)	−0.15 (−0.33, 0.02)	−0.12 (−0.29, 0.06)
Pakistan	−0.60 (−0.77, −0.43)	−0.93 (−1.05, −0.80)	−0.76 (−0.91, −0.61)	−0.11 (−0.17, −0.05)	−0.14 (−0.21, −0.08)	−0.13 (−0.19, −0.06)
Palestine	1.14 (1.04, 1.23)	1.13 (1.04, 1.21)	1.14 (1.04, 1.23)	0.32 (0.19, 0.45)	0.10 (0.03, 0.17)	0.22 (0.12, 0.31)
Panama	−0.51 (−0.57, −0.45)	−0.52 (−0.57, −0.47)	−0.51 (−0.56, −0.46)	0.23 (0.18, 0.27)	0.18 (0.12, 0.23)	0.21 (0.16, 0.25)
Papua New Guinea	0.80 (0.72, 0.88)	0.54 (0.46, 0.62)	0.69 (0.62, 0.77)	−0.11 (−0.13, −0.08)	0.02 (0.00, 0.05)	−0.08 (−0.10, −0.06)
Paraguay	0.41 (0.36, 0.46)	0.77 (0.71, 0.83)	0.59 (0.54, 0.64)	0.20 (0.16, 0.24)	0.17 (0.14, 0.20)	0.19 (0.15, 0.22)
Peru	1.28 (1.19, 1.36)	1.30 (1.22, 1.38)	1.29 (1.21, 1.37)	−0.17 (−0.20, −0.15)	−0.27 (−0.30, −0.25)	−0.22 (−0.24, −0.19)
Philippines	−0.40 (−0.53, −0.27)	−0.15 (−0.27, −0.04)	−0.29 (−0.41, −0.16)	−0.19 (−0.27, −0.12)	−0.28 (−0.35, −0.22)	−0.24 (−0.31, −0.17)
Poland	−0.78 (−0.88, −0.69)	−0.51 (−0.67, −0.36)	−0.65 (−0.77, −0.52)	−0.04 (−0.09, 0.02)	0.00 (−0.07, 0.08)	−0.02 (−0.08, 0.05)
Portugal	−0.25 (−0.30, −0.19)	−0.07 (−0.11, −0.03)	−0.16 (−0.20, −0.12)	0.13 (−0.06, 0.33)	0.20 (−0.01, 0.41)	0.17 (−0.03, 0.36)
Puerto Rico	1.21 (1.08, 1.33)	1.34 (1.22, 1.46)	1.28 (1.16, 1.40)	0.03 (0.00, 0.05)	0.07 (0.04, 0.09)	0.05 (0.03, 0.07)
Qatar	0.28 (0.14, 0.42)	0.47 (0.39, 0.54)	0.35 (0.26, 0.44)	0.33 (0.27, 0.38)	0.17 (0.13, 0.21)	0.28 (0.23, 0.33)

Romania	−0.82 (−0.86, −0.77)	−0.61 (−0.68, −0.55)	−0.71 (−0.77, −0.66)	0.12 (0.03, 0.21)	0.07 (−0.02, 0.17)	0.09 (0.00, 0.19)
Russian Federation	0.20 (0.09, 0.30)	0.52 (0.39, 0.65)	0.38 (0.26, 0.50)	−0.10 (−0.16, −0.05)	−0.25 (−0.32, −0.17)	−0.18 (−0.25, −0.12)
Rwanda	−0.07 (−0.19, 0.05)	−1.01 (−1.14, −0.88)	−0.56 (−0.68, −0.44)	0.06 (0.02, 0.11)	0.10 (0.07, 0.14)	0.09 (0.05, 0.13)
Saint Lucia	1.08 (1.03, 1.14)	1.33 (1.27, 1.39)	1.22 (1.16, 1.27)	−0.22 (−0.27, −0.17)	−0.28 (−0.34, −0.22)	−0.25 (−0.30, −0.20)
Saint Vincent and the Grenadines	0.74 (0.69, 0.79)	0.92 (0.87, 0.98)	0.84 (0.79, 0.89)	0.05 (0.01, 0.10)	0.02 (−0.02, 0.06)	0.03 (−0.01, 0.07)
Samoa	1.47 (1.36, 1.57)	1.29 (1.20, 1.38)	1.39 (1.29, 1.49)	−0.07 (−0.10, −0.04)	−0.07 (−0.09, −0.05)	−0.07 (−0.09, −0.04)
Sao Tome and Principe	0.50 (0.39, 0.61)	0.21 (0.10, 0.32)	0.35 (0.25, 0.46)	0.02 (0.01, 0.03)	−0.01 (−0.02, 0.00)	0.00 (−0.01, 0.02)
Saudi Arabia	0.47 (0.42, 0.53)	0.57 (0.54, 0.61)	0.51 (0.47, 0.56)	−0.01 (−0.03, 0.01)	−0.05 (−0.07, −0.02)	−0.02 (−0.04, 0.00)
Senegal	0.57 (0.46, 0.67)	0.20 (0.09, 0.30)	0.38 (0.28, 0.49)	−0.33 (−0.34, −0.31)	−0.12 (−0.14, −0.10)	−0.22 (−0.24, −0.20)
Serbia	−0.70 (−0.76, −0.65)	−0.74 (−0.82, −0.66)	−0.72 (−0.78, −0.65)	0.11 (0.08, 0.15)	0.42 (0.32, 0.51)	0.26 (0.20, 0.33)
Seychelles	0.69 (0.65, 0.74)	0.78 (0.71, 0.85)	0.78 (0.72, 0.84)	−0.24 (−0.38, −0.09)	−0.26 (−0.44, −0.08)	−0.24 (−0.40, −0.09)
Sierra Leone	0.82 (0.71, 0.92)	0.44 (0.35, 0.53)	0.65 (0.55, 0.74)	−0.11 (−0.16, −0.07)	−0.07 (−0.10, −0.04)	−0.10 (−0.14, −0.06)
Singapore	−0.20 (−0.29, −0.11)	−0.09 (−0.13, −0.04)	−0.16 (−0.22, −0.10)	−0.18 (−0.29, −0.07)	−0.39 (−0.49, −0.30)	−0.28 (−0.38, −0.18)
Slovakia	−0.65 (−0.71, −0.59)	−0.51 (−0.56, −0.46)	−0.59 (−0.65, −0.54)	−0.18 (−0.23, −0.12)	−0.02 (−0.10, 0.06)	−0.10 (−0.16, −0.04)

Slovenia	−0.77 (−0.87, −0.68)	−0.62 (−0.74, −0.49)	−0.71 (−0.82, −0.60)	−0.06 (−0.09, −0.03)	−0.07 (−0.10, −0.03)	−0.06 (−0.09, −0.03)
Solomon Islands	0.43 (0.35, 0.50)	0.46 (0.37, 0.55)	0.43 (0.34, 0.51)	−0.15 (−0.20, −0.10)	−0.15 (−0.21, −0.09)	−0.16 (−0.21, −0.10)
Somalia	0.40 (0.32, 0.49)	−0.10 (−0.18, −0.01)	0.15 (0.07, 0.23)	−0.02 (−0.05, 0.02)	−0.05 (−0.08, −0.02)	−0.03 (−0.06, 0.00)
South Africa	−0.54 (−0.58, −0.51)	−0.60 (−0.69, −0.51)	−0.56 (−0.62, −0.51)	0.19 (0.14, 0.23)	0.10 (0.06, 0.14)	0.14 (0.10, 0.17)
South Sudan	0.75 (0.69, 0.82)	0.26 (0.21, 0.31)	0.51 (0.46, 0.57)	−0.21 (−0.25, −0.18)	−0.19 (−0.23, −0.15)	−0.22 (−0.25, −0.18)
Spain	0.01 (−0.04, 0.05)	0.09 (0.04, 0.13)	0.04 (0.01, 0.08)	−0.17 (−0.24, −0.11)	−0.25 (−0.39, −0.10)	−0.21 (−0.30, −0.11)
Sri Lanka	0.07 (−0.05, 0.18)	0.25 (0.15, 0.36)	0.12 (0.01, 0.23)	0.37 (0.33, 0.41)	0.28 (0.23, 0.33)	0.34 (0.30, 0.38)
Sudan	1.04 (0.94, 1.13)	1.00 (0.92, 1.09)	1.02 (0.93, 1.11)	0.22 (0.16, 0.28)	0.12 (0.05, 0.19)	0.16 (0.10, 0.23)
Suriname	0.49 (0.42, 0.57)	0.71 (0.62, 0.80)	0.60 (0.51, 0.68)	−0.21 (−0.28, −0.13)	−0.32 (−0.38, −0.26)	−0.27 (−0.34, −0.20)
Sweden	0.48 (0.41, 0.54)	0.46 (0.39, 0.53)	0.46 (0.40, 0.53)	−0.07 (−0.09, −0.06)	−0.08 (−0.10, −0.06)	−0.08 (−0.09, −0.07)
Switzerland	0.12 (0.07, 0.16)	0.27 (0.21, 0.33)	0.19 (0.14, 0.24)	−0.01 (−0.04, 0.02)	−0.05 (−0.08, −0.02)	−0.03 (−0.06, 0.00)
Tajikistan	0.79 (0.68, 0.91)	0.97 (0.82, 1.12)	0.89 (0.76, 1.02)	0.04 (−0.01, 0.08)	0.01 (−0.02, 0.04)	0.02 (−0.01, 0.05)
Thailand	0.36 (0.33, 0.38)	0.07 (0.02, 0.11)	0.23 (0.20, 0.26)	0.18 (0.11, 0.24)	0.26 (0.17, 0.35)	0.23 (0.16, 0.30)
Timor−Leste	−0.02 (−0.07, 0.03)	−0.45 (−0.51, −0.39)	−0.21 (−0.26, −0.16)	−0.43 (−0.48, −0.37)	−0.19 (−0.25, −0.12)	−0.32 (−0.38, −0.27)

Togo	0.81 (0.70, 0.91)	0.54 (0.44, 0.64)	0.67 (0.57, 0.77)	−0.15 (−0.17, −0.13)	−0.16 (−0.17, −0.14)	−0.15 (−0.17, −0.13)
Tonga	1.41 (1.27, 1.55)	1.02 (0.90, 1.14)	1.24 (1.11, 1.36)	−0.09 (−0.12, −0.05)	−0.06 (−0.09, −0.02)	−0.07 (−0.10, −0.05)
Trinidad and Tobago	1.06 (1.02, 1.10)	1.14 (1.09, 1.20)	1.11 (1.06, 1.15)	0.03 (−0.02, 0.07)	0.09 (0.03, 0.14)	0.05 (0.00, 0.10)
Tunisia	1.26 (1.15, 1.38)	1.19 (1.08, 1.30)	1.23 (1.11, 1.34)	−0.11 (−0.13, −0.08)	−0.04 (−0.06, −0.02)	−0.08 (−0.10, −0.05)
Turkey	1.64 (1.53, 1.75)	1.68 (1.59, 1.78)	1.66 (1.56, 1.76)	−0.22 (−0.28, −0.17)	−0.25 (−0.29, −0.22)	−0.24 (−0.28, −0.19)
Turkmenistan	0.16 (0.08, 0.24)	0.73 (0.61, 0.86)	0.45 (0.35, 0.54)	0.46 (0.39, 0.53)	0.49 (0.41, 0.58)	0.49 (0.41, 0.57)
Uganda	0.23 (0.11, 0.34)	−0.48 (−0.60, −0.35)	−0.13 (−0.24, −0.01)	−0.11 (−0.13, −0.08)	−0.02 (−0.08, 0.04)	−0.06 (−0.11, −0.02)
Ukraine	0.54 (0.44, 0.63)	0.60 (0.51, 0.69)	0.58 (0.49, 0.67)	0.19 (0.13, 0.26)	0.23 (0.15, 0.30)	0.21 (0.14, 0.28)
United Arab Emirates	1.21 (1.11, 1.31)	1.35 (1.24, 1.47)	1.28 (1.18, 1.39)	−0.93 (−1.82, −0.04)	−1.18 (−2.23, −0.11)	−1.02 (−1.95, −0.07)
United Kingdom	0.08 (0.00, 0.15)	0.20 (0.11, 0.30)	0.14 (0.05, 0.22)	0.02 (−0.01, 0.05)	0.01 (−0.01, 0.04)	0.02 (−0.01, 0.04)
Uruguay	0.48 (0.40, 0.56)	0.50 (0.42, 0.57)	0.49 (0.43, 0.55)	0.17 (0.03, 0.30)	0.53 (0.34, 0.72)	0.34 (0.18, 0.50)
Uzbekistan	0.86 (0.72, 1.01)	1.05 (0.89, 1.21)	0.96 (0.81, 1.11)	−0.18 (−0.25, −0.10)	−0.22 (−0.30, −0.13)	−0.20 (−0.28, −0.12)
Vanuatu	0.85 (0.76, 0.94)	0.59 (0.51, 0.66)	0.72 (0.64, 0.80)	−0.08 (−0.13, −0.03)	−0.11 (−0.16, −0.06)	−0.11 (−0.16, −0.06)
Yemen	0.83 (0.76, 0.89)	0.87 (0.82, 0.92)	0.85 (0.80, 0.90)	0.04 (−0.01, 0.10)	0.04 (−0.01, 0.09)	0.04 (0.00, 0.09)
Zambia	0.03 (−0.07, 0.12)	−0.53 (−0.65, −0.40)	−0.25 (−0.35, −0.14)	−0.40 (−0.47, −0.32)	−0.35 (−0.41, −0.28)	−0.38 (−0.45, −0.31)

Zimbabwe	0.52 (0.34, 0.70)	0.68 (0.51, 0.84)	0.55 (0.38, 0.73)	−0.20 (−0.22, −0.17)	−0.04 (−0.07, 0.00)	−0.12 (−0.15, −0.09)
Bolivia (Plurinational State of)	1.15 (1.08, 1.22)	0.93 (0.87, 0.99)	1.04 (0.98, 1.11)	−0.03 (−0.14, 0.09)	−0.12 (−0.18, −0.06)	−0.07 (−0.16, 0.01)
Brunei Darussalam	0.11 (0.03, 0.18)	0.03 (−0.02, 0.09)	0.07 (0.03, 0.12)	0.08 (0.00, 0.17)	0.07 (0.00, 0.14)	0.08 (0.00, 0.16)
Cabo Verde	0.15 (−0.03, 0.33)	0.04 (−0.17, 0.25)	0.12 (−0.08, 0.32)	0.06 (0.01, 0.10)	−0.05 (−0.14, 0.03)	0.01 (−0.06, 0.07)
Democratic Republic of the Congo	1.52 (1.41, 1.62)	1.04 (0.93, 1.16)	1.31 (1.20, 1.41)	0.12 (0.09, 0.15)	0.38 (0.34, 0.43)	0.24 (0.20, 0.27)
Cote d'Ivoire	0.74 (0.63, 0.85)	0.55 (0.46, 0.64)	0.65 (0.55, 0.75)	−0.24 (−0.30, −0.18)	−0.33 (−0.39, −0.26)	−0.29 (−0.35, −0.23)
Czechia	−0.28 (−0.35, −0.22)	−0.19 (−0.25, −0.12)	−0.24 (−0.30, −0.18)	−0.22 (−0.34, −0.10)	−0.20 (−0.32, −0.09)	−0.21 (−0.33, −0.09)
Micronesia (Federated States of)	0.77 (0.63, 0.91)	0.37 (0.22, 0.52)	0.59 (0.44, 0.73)	−0.17 (−0.19, −0.15)	−0.22 (−0.24, −0.19)	−0.19 (−0.21, −0.17)
Georgia	1.30 (1.14, 1.46)	1.32 (1.20, 1.45)	1.31 (1.17, 1.45)	0.05 (0.02, 0.08)	0.08 (0.05, 0.12)	0.06 (0.03, 0.09)
Iran (Islamic Republic of)	1.45 (1.32, 1.59)	1.58 (1.43, 1.73)	1.51 (1.37, 1.65)	−0.10 (−0.13, −0.07)	−0.09 (−0.11, −0.06)	−0.09 (−0.12, −0.06)
Lao People's Democratic Republic	−0.19 (−0.25, −0.14)	−0.65 (−0.72, −0.58)	−0.40 (−0.46, −0.34)	0.03 (−0.06, 0.13)	0.13 (0.03, 0.23)	0.08 (−0.02, 0.17)
North Macedonia	−0.69 (−0.74, −0.65)	−0.39 (−0.44, −0.35)	−0.54 (−0.58, −0.51)	−0.04 (−0.08, 0.01)	−0.27 (−0.32, −0.22)	−0.16 (−0.20, −0.12)
Republic of Moldova	0.41 (0.32, 0.49)	0.65 (0.56, 0.74)	0.53 (0.44, 0.61)	−0.08 (−0.14, −0.03)	−0.12 (−0.19, −0.05)	−0.11 (−0.17, −0.04)
Democratic People's Republic of Korea	1.10 (0.97, 1.23)	0.92 (0.79, 1.05)	1.00 (0.88, 1.13)	0.23 (0.15, 0.31)	0.35 (0.27, 0.43)	0.31 (0.23, 0.39)
Republic of Korea	−0.02 (−0.12, 0.08)	0.05 (−0.01, 0.11)	0.01 (−0.05, 0.06)	1.64 (1.00, 2.27)	1.90 (1.42, 2.39)	1.78 (1.28, 2.29)

Eswatini	0.08 (−0.06, 0.22)	0.08 (−0.04, 0.21)	0.08 (−0.06, 0.21)	−0.24 (−0.30, −0.18)	−0.30 (−0.34, −0.26)	−0.29 (−0.34, −0.25)
Syrian Arab Republic	1.11 (0.93, 1.30)	1.12 (0.95, 1.29)	1.12 (0.95, 1.30)	0.01 (−0.10, 0.11)	−0.02 (−0.07, 0.04)	−0.01 (−0.08, 0.07)
Taiwan (Province of China)	1.17 (1.04, 1.29)	0.68 (0.57, 0.78)	0.91 (0.80, 1.02)	−0.06 (−0.08, −0.03)	−0.05 (−0.08, −0.02)	−0.06 (−0.08, −0.03)
United Republic of Tanzania	0.10 (−0.03, 0.22)	−0.27 (−0.39, −0.15)	−0.08 (−0.20, 0.04)	−0.35 (−0.45, −0.26)	−0.43 (−0.52, −0.34)	−0.40 (−0.49, −0.30)
Bahamas	0.88 (0.79, 0.98)	1.24 (1.13, 1.35)	1.08 (0.98, 1.18)	−0.12 (−0.15, −0.09)	−0.12 (−0.16, −0.08)	−0.12 (−0.16, −0.09)
Gambia	0.83 (0.73, 0.94)	0.56 (0.47, 0.65)	0.69 (0.59, 0.78)	−0.42 (−0.46, −0.38)	−0.34 (−0.38, −0.29)	−0.39 (−0.43, −0.35)
United States of America	0.04 (−0.23, 0.31)	0.53 (0.22, 0.84)	0.29 (0.01, 0.58)	0.17 (0.05, 0.28)	0.09 (−0.02, 0.21)	0.13 (0.01, 0.24)
Venezuela (Bolivarian Republic of)	−0.12 (−0.19, −0.05)	−0.25 (−0.32, −0.18)	−0.18 (−0.25, −0.11)	−0.14 (−0.20, −0.08)	−0.28 (−0.37, −0.19)	−0.21 (−0.28, −0.14)
Viet Nam	0.02 (−0.07, 0.11)	−0.06 (−0.16, 0.04)	−0.01 (−0.10, 0.09)	−0.05 (−0.13, 0.02)	0.32 (0.24, 0.40)	0.17 (0.09, 0.24)
United States Virgin Islands	1.04 (0.97, 1.10)	1.25 (1.17, 1.34)	1.15 (1.07, 1.23)	−0.04 (−0.08, 0.01)	−0.12 (−0.16, −0.07)	−0.07 (−0.12, −0.03)
Monaco	−0.10 (−0.13, −0.07)	−0.10 (−0.13, −0.07)	−0.11 (−0.13, −0.09)	0.00 (−0.06, 0.06)	0.05 (−0.04, 0.13)	0.02 (−0.05, 0.09)
San Marino	−0.08 (−0.12, −0.05)	−0.03 (−0.06, −0.01)	−0.06 (−0.09, −0.03)	−0.01 (−0.07, 0.06)	0.05 (−0.01, 0.11)	0.02 (−0.04, 0.08)
Saint Kitts and Nevis	0.26 (0.19, 0.33)	0.53 (0.48, 0.57)	0.40 (0.34, 0.46)	−0.10 (−0.13, −0.06)	−0.08 (−0.10, −0.06)	−0.09 (−0.12, −0.07)
Cook Islands	1.42 (1.34, 1.49)	1.18 (1.12, 1.24)	1.28 (1.21, 1.34)	−0.05 (−0.08, −0.02)	−0.03 (−0.06, 0.00)	−0.04 (−0.07, −0.01)

Nauru	1.05 (0.93, 1.17)	0.85 (0.76, 0.94)	0.92 (0.82, 1.03)	−0.07 (−0.08, −0.05)	−0.09 (−0.10, −0.07)	−0.08 (−0.09, −0.06)
Niue	1.33 (1.26, 1.40)	1.06 (0.99, 1.13)	1.22 (1.15, 1.29)	−0.17 (−0.20, −0.14)	−0.06 (−0.09, −0.03)	−0.12 (−0.15, −0.10)
Palau	1.15 (1.07, 1.23)	0.92 (0.85, 0.98)	1.08 (1.00, 1.15)	−0.14 (−0.18, −0.11)	−0.07 (−0.10, −0.04)	−0.11 (−0.14, −0.08)
Tokelau	0.78 (0.63, 0.92)	0.67 (0.55, 0.79)	0.77 (0.64, 0.90)	−0.05 (−0.08, −0.02)	−0.10 (−0.13, −0.07)	−0.07 (−0.10, −0.04)
Tuvalu	0.89 (0.77, 1.02)	0.60 (0.47, 0.73)	0.80 (0.67, 0.93)	−0.10 (−0.15, −0.06)	−0.10 (−0.13, −0.06)	−0.10 (−0.14, −0.07)
**Region**						
Global	−0.12 (−0.23, −0.01)	−0.22 (−0.33, −0.10)	−0.16 (−0.28, −0.05)	−0.02 (−0.10, 0.05)	−0.11 (−0.19, −0.03)	−0.07 (−0.14, 0.01)
Andean Latin America	1.05 (0.96, 1.13)	1.10 (1.02, 1.17)	1.08 (0.99, 1.16)	−0.31 (−0.41, −0.21)	−0.32 (−0.43, −0.21)	−0.31 (−0.41, −0.20)
Australasia	0.36 (0.29, 0.42)	0.16 (0.04, 0.27)	0.27 (0.18, 0.35)	−0.08 (−0.10, −0.06)	−0.03 (−0.04, −0.01)	−0.05 (−0.07, −0.04)
Caribbean	0.70 (0.62, 0.77)	0.85 (0.78, 0.93)	0.77 (0.70, 0.85)	−0.06 (−0.12, −0.01)	−0.07 (−0.13, −0.01)	−0.06 (−0.12, −0.01)
Central Asia	0.98 (0.86, 1.10)	1.10 (0.97, 1.24)	1.05 (0.92, 1.17)	−0.01 (−0.03, 0.01)	0.04 (0.02, 0.06)	0.01 (−0.01, 0.03)
Central Europe	−0.69 (−0.74, −0.64)	−0.54 (−0.61, −0.47)	−0.61 (−0.67, −0.56)	−0.03 (−0.07, 0.01)	−0.14 (−0.18, −0.09)	−0.09 (−0.13, −0.05)
Central Latin America	−1.50 (−1.59, −1.42)	−1.32 (−1.42, −1.21)	−1.41 (−1.50, −1.32)	0.19 (0.14, 0.24)	0.11 (0.05, 0.16)	0.15 (0.10, 0.20)
Central Sub−Saharan Africa	1.34 (1.26, 1.43)	0.78 (0.70, 0.86)	1.08 (1.00, 1.16)	−0.08 (−0.12, −0.04)	−0.04 (−0.09, 0.01)	−0.07 (−0.11, −0.03)

East Asia	−0.12 (−0.34, 0.10)	−0.08 (−0.26, 0.11)	−0.10 (−0.30, 0.11)	0.31 (0.28, 0.34)	0.44 (0.40, 0.48)	0.37 (0.34, 0.40)
Eastern Europe	0.29 (0.19, 0.39)	0.57 (0.46, 0.68)	0.45 (0.34, 0.55)	−0.08 (−0.13, −0.02)	−0.15 (−0.24, −0.07)	−0.12 (−0.18, −0.05)
Eastern Sub−Saharan Africa	0.05 (−0.02, 0.12)	−0.57 (−0.65, −0.49)	−0.25 (−0.33, −0.18)	0.42 (0.38, 0.45)	0.64 (0.61, 0.68)	0.52 (0.49, 0.56)
High−income Asia Pacific	−0.87 (−1.08, −0.65)	−0.36 (−0.48, −0.24)	−0.60 (−0.74, −0.45)	0.87 (0.20, 1.55)	0.63 (0.15, 1.10)	0.74 (0.18, 1.31)
High−income North America	0.11 (−0.13, 0.34)	0.53 (0.26, 0.81)	0.32 (0.08, 0.57)	0.04 (−0.05, 0.12)	0.05 (−0.02, 0.12)	0.04 (−0.03, 0.12)
North Africa and Middle East	1.21 (1.11, 1.30)	1.30 (1.22, 1.37)	1.25 (1.17, 1.34)	−0.09 (−0.11, −0.08)	0.01 (0.00, 0.02)	−0.04 (−0.06, −0.03)
Oceania	0.84 (0.77, 0.92)	0.60 (0.52, 0.68)	0.74 (0.66, 0.82)	−0.30 (−0.35, −0.26)	−0.28 (−0.32, −0.24)	−0.31 (−0.35, −0.28)
South Asia	−1.23 (−1.49, −0.98)	−1.55 (−1.83, −1.28)	−1.39 (−1.65, −1.13)	−0.18 (−0.22, −0.14)	−0.12 (−0.15, −0.09)	−0.15 (−0.18, −0.11)
Southeast Asia	−0.08 (−0.22, 0.05)	−0.07 (−0.18, 0.05)	−0.08 (−0.20, 0.05)	0.07 (0.03, 0.10)	0.22 (0.14, 0.30)	0.15 (0.10, 0.20)
Southern Latin America	0.35 (0.26, 0.44)	0.36 (0.22, 0.50)	0.36 (0.25, 0.48)	0.00 (−0.02, 0.02)	0.00 (−0.02, 0.02)	0.01 (−0.01, 0.03)
Southern Sub−Saharan Africa	−0.24 (−0.31, −0.17)	−0.25 (−0.29, −0.21)	−0.24 (−0.28, −0.20)	0.07 (0.01, 0.13)	0.12 (0.06, 0.17)	0.09 (0.04, 0.14)
Tropical Latin America	−0.50 (−0.56, −0.44)	−0.28 (−0.31, −0.25)	−0.39 (−0.43, −0.35)	−0.20 (−0.25, −0.15)	−0.26 (−0.32, −0.20)	−0.23 (−0.28, −0.17)
Western Europe	0.12 (0.08, 0.16)	0.20 (0.14, 0.27)	0.16 (0.11, 0.20)	0.11 (0.06, 0.15)	0.13 (0.08, 0.18)	0.12 (0.07, 0.16)
Western Sub−Saharan Africa	0.32 (0.25, 0.40)	−0.09 (−0.15, −0.03)	0.11 (0.04, 0.17)	−0.03 (−0.09, 0.02)	0.03 (−0.01, 0.07)	0.00 (−0.05, 0.05)
High SDI	0.31 (0.21, 0.41)	0.45 (0.34, 0.55)	0.38 (0.27, 0.48)	−0.05 (−0.07, −0.04)	−0.01 (−0.03, 0.01)	−0.03 (−0.05, −0.02)

High−middle SDI	−0.02 (−0.13, 0.10)	0.03 (−0.07, 0.12)	0.01 (−0.09, 0.11)	−0.44 (−0.54, −0.33)	−0.46 (−0.58, −0.34)	−0.45 (−0.56, −0.34)
Middle SDI	−0.11 (−0.24, 0.01)	−0.06 (−0.18, 0.05)	−0.09 (−0.21, 0.03)	0.09 (0.03, 0.16)	0.13 (0.05, 0.20)	0.11 (0.04, 0.18)
Low−middle SDI	−0.83 (−1.00, −0.66)	−1.07 (−1.26, −0.87)	−0.95 (−1.13, −0.77)	0.00 (−0.07, 0.07)	0.04 (−0.03, 0.12)	0.02 (−0.05, 0.10)
Low SDI	−0.16 (−0.24, −0.08)	−0.67 (−0.78, −0.55)	−0.41 (−0.51, −0.32)	0.13 (0.11, 0.14)	0.17 (0.15, 0.19)	0.15 (0.13, 0.16)

Abbreviations: ASIRs, age-standardized incidence rates; EAPCs, estimated annual percentage changes; UIs, uncertainty intervals.

Table S3. Incident cases for diarrheal diseases in 1990, 2019, 2020, and 2040 for males, females, and both genders combined at the national, regional, and global levels

Location	Incidence cases of diarrheal diseases (× 100,000)											
	1990			2019			2020			2040		
	Male	Female	Both genders combined	Male	Female	Both genders combined	Male	Female	Both genders combined	Male	Female	Both genders combined
**Nation**												
Afghanistan	58.46 (50.75, 66.76)	53.39 (46.90, 60.97)	111.85 (97.69, 126.80)	264.44 (226.52, 303.63)	245.93 (212.59, 281.88)	510.37 (443.39, 581.91)	251.68 (242.53, 259.61)	231.07 (222.55, 238.46)	482.75 (466.65, 496.92)	444.45 (426.86, 463.93)	416.48 (398.60, 435.55)	860.93 (827.09, 897.03)
Albania	19.62 (16.84, 22.63)	22.45 (19.12, 25.82)	42.06 (36.17, 48.26)	12.06 (10.48, 13.71)	15.02 (13.23, 17.21)	27.08 (24.01, 30.46)	12.32 (11.85, 12.81)	15.06 (14.38, 15.61)	27.38 (26.33, 28.35)	11.60 (11.07, 12.16)	13.70 (12.97, 14.41)	25.30 (24.10, 26.43)
Algeria	107.23 (93.26, 122.87)	108.05 (93.60, 122.59)	215.27 (186.34, 244.24)	202.21 (179.35, 227.13)	198.63 (175.80, 223.28)	400.83 (357.51, 447.43)	214.73 (211.03, 219.53)	210.67 (207.29, 214.18)	425.41 (419.29, 432.65)	298.72 (292.41, 308.26)	292.03 (285.93, 299.49)	590.75 (578.58, 606.28)
American Samoa	0.21 (0.19, 0.24)	0.16 (0.15, 0.18)	0.38 (0.34, 0.42)	0.39 (0.35, 0.44)	0.30 (0.26, 0.33)	0.69 (0.62, 0.76)	0.38 (0.36, 0.40)	0.29 (0.27, 0.30)	0.67 (0.63, 0.69)	0.52 (0.50, 0.55)	0.41 (0.39, 0.42)	0.93 (0.89, 0.96)
Andorra	0.09 (0.08, 0.10)	0.07 (0.06, 0.08)	0.16 (0.15, 0.18)	0.14 (0.12, 0.16)	0.12 (0.10, 0.13)	0.25 (0.23, 0.28)	0.14 (0.13, 0.14)	0.12 (0.11, 0.13)	0.25 (0.25, 0.27)	0.17 (0.17, 0.18)	0.15 (0.15, 0.16)	0.32 (0.31, 0.34)
Angola	56.92 (51.16, 62.62)	56.22 (50.50, 61.93)	113.14 (102.24, 123.37)	181.24 (163.95, 199.02)	164.51 (146.72, 182.59)	345.75 (312.73, 378.02)	169.61 (163.65, 175.36)	158.54 (152.84, 163.81)	328.16 (316.63, 339.07)	242.68 (228.50, 256.33)	233.39 (219.82, 246.44)	476.07 (448.77, 501.86)
Antigua and Barbuda	0.22 (0.20, 0.25)	0.22 (0.20, 0.24)	0.44 (0.40, 0.48)	0.45 (0.41, 0.51)	0.44 (0.39, 0.48)	0.89 (0.80, 0.98)	0.46 (0.45, 0.47)	0.44 (0.43, 0.45)	0.90 (0.88, 0.92)	0.54 (0.50, 0.59)	0.54 (0.49, 0.58)	1.08 (0.99, 1.16)
Argentina	77.56 (68.92, 87.03)	62.51 (55.37, 69.99)	140.07 (125.77, 155.41)	123.21 (108.96, 139.26)	93.78 (83.85, 104.97)	216.99 (194.50, 242.40)	152.34 (143.59, 159.51)	133.07 (123.38, 140.63)	285.41 (267.48, 299.79)	154.01 (141.03, 163.15)	129.22 (115.11, 139.57)	283.23 (256.97, 301.31)
Armenia	13.75 (12.06, 15.48)	15.01 (13.13, 16.96)	28.76 (25.45, 32.26)	14.75 (12.95, 16.72)	16.47 (14.30, 18.72)	31.21 (27.50, 35.09)	14.66 (14.32, 15.04)	16.40 (16.06, 16.82)	31.06 (30.44, 31.79)	14.06 (13.65, 14.58)	15.10 (14.71, 15.67)	29.15 (28.44, 30.18)
Australia	24.71 (22.05, 27.51)	22.41 (20.01, 24.97)	47.12 (42.09, 52.38)	43.10 (38.43, 47.95)	36.30 (32.30, 40.60)	79.40 (71.61, 87.90)	44.63 (42.15, 48.19)	37.89 (35.75, 41.88)	82.52 (78.54, 89.72)	66.22 (58.38, 78.39)	60.13 (51.33, 74.56)	126.35 (110.24, 152.36)

Austria	13.17 (11.72, 14.71)	14.23 (12.71, 15.92)	27.39 (24.63, 30.31)	16.44 (14.79, 18.19)	17.10 (15.48, 18.97)	33.54 (30.50, 37.02)	15.85 (15.26, 16.40)	16.87 (16.19, 17.60)	32.72 (31.60, 33.81)	21.13 (18.79, 23.69)	21.00 (18.47, 23.77)	42.13 (37.47, 47.28)
Azerbaijan	27.60 (24.21, 30.97)	29.85 (26.08, 33.46)	57.45 (50.70, 64.06)	50.97 (44.28, 58.18)	53.58 (46.61, 61.90)	104.55 (91.31, 118.75)	51.80 (50.04, 53.37)	56.45 (54.68, 58.42)	108.25 (105.15, 111.01)	51.74 (49.84, 53.65)	53.86 (51.61, 56.16)	105.60 (101.98, 109.41)
Bahrain	1.92 (1.66, 2.21)	1.55 (1.35, 1.77)	3.47 (3.03, 3.94)	7.39 (6.46, 8.42)	4.83 (4.24, 5.46)	12.22 (10.85, 13.68)	7.81 (7.47, 8.18)	5.26 (5.07, 5.45)	13.07 (12.58, 13.55)	14.26 (13.64, 14.81)	8.78 (8.44, 9.01)	23.04 (22.12, 23.78)
Bangladesh	785.84 (689.26, 885.89)	879.96 (776.59, 994.36)	1665.80 (1469.59, 1865.30)	986.62 (878.93, 1098.93)	1087.11 (975.63, 1214.41)	2073.73 (1870.50, 2298.53)	970.02 (907.84, 1019.05)	1055.85 (968.37, 1127.00)	2025.87 (1882.88, 2135.98)	1019.11 (947.12, 1083.45)	1102.75 (1004.85, 1187.31)	2121.86 (1969.37, 2260.81)
Barbados	0.97 (0.87, 1.08)	0.94 (0.84, 1.04)	1.91 (1.73, 2.10)	1.75 (1.56, 1.94)	1.84 (1.65, 2.05)	3.59 (3.24, 3.95)	1.70 (1.62, 1.76)	1.75 (1.65, 1.82)	3.45 (3.28, 3.58)	1.92 (1.85, 1.97)	2.00 (1.92, 2.07)	3.92 (3.78, 4.03)
Belarus	39.60 (34.49, 44.89)	47.59 (42.26, 53.99)	87.20 (77.00, 97.98)	35.97 (31.80, 40.93)	47.63 (41.96, 53.77)	83.60 (74.26, 93.91)	38.44 (36.56, 40.69)	49.73 (47.87, 52.01)	88.17 (84.71, 92.60)	34.51 (33.23, 36.01)	43.59 (42.08, 45.18)	78.10 (75.47, 81.04)
Belgium	21.17 (18.89, 23.67)	22.08 (19.76, 24.32)	43.24 (38.91, 47.60)	30.50 (27.40, 33.98)	32.46 (29.50, 35.94)	62.96 (57.44, 69.08)	30.97 (29.92, 31.92)	33.18 (32.03, 34.21)	64.16 (62.20, 66.05)	36.82 (35.43, 38.23)	37.85 (36.13, 39.41)	74.67 (71.76, 77.32)
Belize	0.73 (0.65, 0.82)	0.62 (0.54, 0.69)	1.34 (1.20, 1.50)	1.93 (1.71, 2.17)	1.68 (1.47, 1.87)	3.61 (3.20, 4.01)	2.09 (1.99, 2.18)	1.76 (1.67, 1.84)	3.85 (3.68, 4.01)	2.90 (2.78, 3.02)	2.61 (2.50, 2.70)	5.51 (5.28, 5.71)
Benin	27.45 (24.35, 30.69)	28.32 (25.22, 31.95)	55.77 (49.80, 62.04)	73.19 (66.01, 81.10)	72.79 (65.37, 80.86)	145.99 (132.32, 159.88)	70.70 (68.82, 72.59)	70.04 (68.38, 71.63)	140.74 (137.56, 143.93)	115.52 (110.66, 120.60)	110.45 (104.82, 115.82)	225.97 (215.86, 235.49)
Bermuda	0.22 (0.19, 0.24)	0.19 (0.17, 0.21)	0.40 (0.37, 0.45)	0.39 (0.35, 0.43)	0.40 (0.36, 0.45)	0.79 (0.71, 0.87)	0.38 (0.36, 0.39)	0.41 (0.39, 0.42)	0.79 (0.75, 0.81)	0.43 (0.41, 0.45)	0.51 (0.49, 0.53)	0.94 (0.91, 0.97)
Bhutan	4.45 (3.88, 5.06)	5.44 (4.75, 6.14)	9.88 (8.69, 11.11)	5.87 (5.20, 6.59)	5.55 (4.96, 6.21)	11.43 (10.22, 12.65)	6.90 (6.61, 7.15)	6.63 (6.37, 6.90)	13.53 (13.01, 14.01)	7.96 (7.60, 8.31)	8.08 (7.72, 8.44)	16.04 (15.36, 16.66)
Bosnia and Herzegovina	28.22 (24.38, 32.51)	31.01 (26.76, 35.51)	59.23 (51.55, 67.68)	13.88 (12.23, 15.68)	16.62 (14.63, 18.85)	30.50 (27.17, 34.37)	14.02 (13.54, 14.47)	16.75 (16.17, 17.26)	30.77 (29.80, 31.65)	12.00 (11.51, 12.50)	14.34 (13.78, 14.99)	26.34 (25.35, 27.37)
Botswana	8.45 (7.51, 9.51)	7.63 (6.82, 8.51)	16.08 (14.48, 17.82)	15.43 (13.65, 17.31)	13.60 (12.15, 15.12)	29.04 (25.98, 32.16)	15.74 (15.31, 16.13)	13.60 (13.21, 13.96)	29.34 (28.67, 29.97)	19.79 (19.07, 20.46)	17.31 (16.81, 17.78)	37.10 (35.96, 38.10)

Brazil	681.34 (612.48, 747.85)	679.36 (610.38, 751.90)	1360.70 (1223.27, 1497.38)	854.59 (777.84, 934.69)	935.07 (850.82, 1021.44)	1789.66 (1628.32, 1956.07)	949.52 (913.64, 996.87)	1029.40 (986.36, 1089.67)	1978.93 (1903.55, 2079.66)	1040.81 (980.04, 1098.03)	1153.30 (1085.46, 1222.51)	2194.11 (2072.58, 2316.74)
Bulgaria	41.90 (36.65, 47.73)	47.32 (41.65, 53.57)	89.22 (79.00, 100.83)	25.08 (22.08, 28.17)	30.49 (26.81, 34.64)	55.57 (49.28, 62.38)	27.88 (26.20, 29.92)	33.89 (31.93, 36.51)	61.76 (58.40, 66.13)	10.86 (9.38, 13.35)	14.36 (12.66, 16.86)	25.23 (22.16, 29.91)
Burkina Faso	58.52 (53.18, 63.90)	66.93 (60.69, 73.25)	125.45 (113.99, 136.02)	153.81 (136.45, 171.71)	158.53 (140.34, 176.33)	312.34 (279.42, 347.21)	154.82 (151.58, 157.29)	158.36 (155.11, 160.61)	313.17 (306.87, 317.12)	265.22 (258.39, 272.28)	268.46 (261.57, 275.48)	533.68 (521.47, 547.24)
Burundi	34.63 (31.08, 38.23)	36.87 (32.89, 40.67)	71.51 (64.43, 78.46)	85.25 (76.93, 92.90)	75.24 (67.50, 82.81)	160.48 (145.59, 174.37)	83.33 (81.36, 85.00)	73.08 (71.01, 74.71)	156.41 (152.77, 159.36)	140.25 (136.25, 143.90)	124.78 (120.97, 128.67)	265.03 (257.99, 272.04)
Cambodia	43.40 (38.58, 47.68)	43.24 (38.12, 48.00)	86.64 (77.07, 95.07)	57.00 (50.93, 62.99)	54.18 (48.80, 59.70)	111.18 (100.60, 122.09)	59.30 (56.81, 62.48)	56.13 (53.79, 59.27)	115.43 (110.96, 121.41)	79.34 (74.55, 85.02)	77.81 (73.54, 83.36)	157.15 (148.56, 167.70)
Cameroon	63.60 (57.92, 69.27)	64.86 (58.54, 70.89)	128.46 (116.87, 139.63)	193.72 (175.42, 214.32)	183.06 (164.81, 201.87)	376.79 (341.40, 413.85)	186.56 (179.92, 192.13)	173.91 (167.82, 178.93)	360.47 (348.55, 370.50)	236.15 (222.62, 250.36)	219.17 (206.52, 233.07)	455.32 (429.42, 481.31)
Canada	73.05 (64.03, 83.06)	51.21 (45.23, 58.64)	124.25 (110.69, 140.56)	122.90 (109.33, 137.86)	84.43 (75.15, 94.63)	207.33 (185.94, 230.45)	123.69 (120.89, 126.62)	90.12 (87.55, 94.83)	213.81 (209.59, 220.47)	144.77 (140.66, 151.26)	107.85 (103.82, 116.44)	252.62 (245.66, 266.98)
Central African Republic	13.67 (12.38, 14.99)	13.83 (12.49, 15.06)	27.50 (25.03, 29.86)	34.60 (30.74, 38.37)	31.59 (28.06, 35.08)	66.18 (58.82, 72.78)	30.17 (29.51, 31.23)	26.96 (26.25, 27.94)	57.14 (55.94, 59.01)	37.93 (36.76, 39.12)	35.60 (34.34, 36.86)	73.54 (71.40, 75.88)
Chad	41.65 (37.44, 45.84)	44.58 (39.98, 48.96)	86.23 (78.15, 94.35)	136.61 (123.80, 150.68)	134.11 (121.38, 147.24)	270.72 (245.51, 297.31)	136.99 (133.94, 139.23)	134.11 (130.78, 136.24)	271.10 (264.94, 275.18)	254.71 (245.61, 265.66)	247.77 (238.75, 257.38)	502.48 (485.33, 521.26)
Chile	36.99 (32.27, 41.89)	30.46 (26.83, 34.86)	67.45 (60.18, 75.87)	49.60 (44.42, 55.91)	43.70 (39.18, 48.92)	93.30 (84.34, 103.46)	49.27 (47.71, 50.82)	45.67 (43.91, 47.33)	94.94 (91.81, 97.82)	55.20 (52.80, 57.71)	52.17 (49.76, 55.21)	107.37 (102.85, 112.66)
China	2855.81 (2569.08, 3149.61)	2508.27 (2256.01, 2783.70)	5364.08 (4821.46, 5931.70)	3860.67 (3474.00, 4284.95)	3315.09 (2991.38, 3668.66)	7175.76 (6475.38, 7946.00)	3922.53 (3820.87, 4015.53)	3406.66 (3312.34, 3499.35)	7329.19 (7166.39, 7505.94)	4744.00 (4518.68, 5003.97)	4399.12 (4159.31, 4695.16)	9143.12 (8722.11, 9668.79)
Colombia	160.20 (142.64, 179.86)	134.91 (120.53, 149.43)	295.12 (264.71, 326.11)	177.88 (157.75, 199.52)	165.33 (146.36, 184.79)	343.21 (307.26, 379.27)	197.65 (191.89, 203.49)	179.96 (173.98, 186.48)	377.61 (367.39, 388.98)	248.10 (237.51, 259.09)	239.90 (227.96, 253.12)	487.99 (467.69, 508.75)

Comoros	2.98 (2.65, 3.31)	3.08 (2.75, 3.44)	6.06 (5.46, 6.66)	4.93 (4.38, 5.45)	4.40 (3.94, 4.89)	9.33 (8.38, 10.27)	4.71 (4.50, 4.92)	4.22 (4.01, 4.40)	8.93 (8.53, 9.30)	6.16 (5.88, 6.43)	5.36 (5.11, 5.58)	11.52 (11.02, 11.97)
Costa Rica	13.75 (12.02, 15.62)	11.45 (10.07, 12.84)	25.20 (22.29, 28.30)	17.12 (15.14, 19.21)	15.58 (13.87, 17.53)	32.70 (29.31, 36.36)	17.33 (16.52, 18.28)	15.90 (15.18, 16.62)	33.24 (31.81, 34.69)	21.23 (19.99, 22.80)	21.77 (20.65, 22.91)	43.00 (41.00, 45.45)
Croatia	22.98 (19.99, 26.21)	24.39 (21.39, 27.78)	47.37 (41.57, 53.30)	14.97 (13.23, 16.81)	17.24 (15.36, 19.31)	32.21 (28.85, 35.69)	15.04 (14.55, 15.56)	17.33 (16.71, 18.04)	32.37 (31.36, 33.46)	12.70 (12.22, 13.30)	14.13 (13.51, 14.83)	26.83 (25.86, 28.07)
Cuba	45.64 (41.52, 50.12)	36.99 (33.48, 40.67)	82.63 (76.05, 89.92)	70.47 (63.88, 78.73)	63.48 (57.91, 69.98)	133.95 (123.12, 146.88)	68.37 (66.29, 70.05)	62.73 (60.97, 64.66)	131.10 (127.65, 134.42)	75.19 (72.97, 77.01)	74.36 (72.21, 76.36)	149.55 (145.59, 153.17)
Cyprus	1.80 (1.59, 2.02)	1.63 (1.44, 1.83)	3.43 (3.05, 3.83)	2.79 (2.50, 3.09)	2.50 (2.26, 2.77)	5.29 (4.79, 5.84)	2.97 (2.70, 3.35)	2.67 (2.43, 3.10)	5.65 (5.13, 6.42)	3.29 (2.91, 3.76)	3.41 (3.01, 3.92)	6.70 (5.95, 7.62)
Congo	11.12 (9.93, 12.22)	11.01 (9.81, 12.18)	22.13 (19.83, 24.35)	32.99 (29.66, 36.30)	26.04 (23.31, 28.80)	59.04 (53.49, 64.57)	29.58 (28.55, 30.65)	23.72 (22.83, 24.66)	53.30 (51.57, 55.35)	35.20 (33.42, 37.01)	27.60 (26.21, 28.86)	62.80 (59.94, 65.69)
Denmark	10.94 (9.76, 12.17)	11.37 (10.27, 12.71)	22.31 (20.21, 24.62)	15.02 (13.46, 16.71)	16.15 (14.49, 17.94)	31.17 (28.33, 34.37)	14.75 (14.19, 15.41)	15.69 (14.88, 16.41)	30.44 (29.23, 31.70)	17.36 (16.60, 18.02)	17.92 (17.03, 18.64)	35.28 (33.69, 36.64)
Djibouti	2.95 (2.63, 3.28)	2.43 (2.15, 2.70)	5.38 (4.79, 5.96)	7.56 (6.74, 8.46)	5.50 (4.91, 6.10)	13.06 (11.73, 14.49)	7.72 (7.43, 8.05)	5.81 (5.59, 6.08)	13.52 (13.03, 14.09)	9.99 (9.43, 10.54)	7.84 (7.41, 8.29)	17.83 (16.87, 18.76)
Dominica	0.28 (0.25, 0.31)	0.27 (0.24, 0.30)	0.55 (0.49, 0.60)	0.36 (0.32, 0.40)	0.33 (0.29, 0.36)	0.69 (0.62, 0.75)	0.36 (0.35, 0.36)	0.32 (0.32, 0.33)	0.68 (0.66, 0.69)	0.38 (0.37, 0.39)	0.36 (0.35, 0.37)	0.74 (0.72, 0.76)
Dominican Republic	33.62 (30.59, 36.82)	29.33 (26.63, 32.27)	62.95 (57.45, 68.56)	61.80 (55.58, 68.77)	55.30 (49.80, 61.31)	117.10 (105.89, 129.23)	61.91 (60.46, 63.19)	54.83 (53.27, 56.12)	116.75 (113.90, 119.07)	69.71 (67.87, 71.52)	65.65 (63.84, 67.48)	135.36 (131.61, 138.64)
Ecuador	42.48 (37.99, 47.18)	38.98 (34.57, 43.47)	81.46 (72.92, 89.96)	79.79 (71.73, 87.90)	80.88 (72.55, 89.19)	160.68 (145.26, 175.56)	81.57 (80.22, 83.15)	80.66 (79.18, 82.13)	162.23 (159.94, 165.00)	102.62 (99.48, 106.02)	102.60 (99.60, 105.45)	205.22 (199.78, 210.87)
Egypt	259.34 (236.16, 287.08)	241.60 (218.87, 266.04)	500.93 (456.72, 551.56)	499.51 (444.69, 558.46)	469.27 (419.33, 524.75)	968.78 (868.01, 1075.73)	498.28 (489.36, 507.80)	484.27 (475.40, 492.49)	982.55 (965.52, 997.98)	658.08 (639.04, 683.92)	684.63 (666.06, 708.31)	1342.71 (1307.75, 1390.15)
El Salvador	32.39 (28.74, 36.34)	27.76 (24.46, 31.31)	60.15 (53.70, 67.21)	26.92 (23.81, 30.11)	27.66 (24.73, 30.66)	54.59 (49.27, 60.24)	25.95 (24.00, 27.67)	26.73 (24.82, 28.84)	52.67 (48.91, 56.29)	24.94 (23.03, 26.80)	29.49 (27.60, 31.30)	54.43 (50.80, 58.05)

Equatorial Guinea	2.47 (2.25, 2.69)	2.61 (2.37, 2.87)	5.08 (4.60, 5.53)	6.68 (5.92, 7.44)	5.19 (4.63, 5.87)	11.87 (10.60, 13.21)	6.59 (6.43, 6.76)	5.24 (5.10, 5.36)	11.83 (11.58, 12.08)	10.45 (9.96, 10.89)	7.72 (7.36, 8.00)	18.17 (17.35, 18.79)
Eritrea	19.87 (17.57, 22.10)	20.58 (18.41, 22.80)	40.46 (36.15, 44.77)	51.05 (44.80, 57.43)	41.09 (36.21, 45.69)	92.14 (81.89, 102.11)	44.68 (43.82, 45.51)	37.77 (36.90, 38.59)	82.45 (80.88, 83.80)	59.03 (56.77, 60.85)	50.86 (49.05, 52.48)	109.89 (106.19, 113.21)
Estonia	6.58 (5.77, 7.48)	7.52 (6.59, 8.49)	14.11 (12.44, 15.85)	5.52 (4.84, 6.27)	7.95 (7.03, 8.97)	13.48 (12.00, 15.11)	5.40 (4.96, 5.64)	7.52 (6.85, 7.88)	12.92 (11.85, 13.45)	5.08 (4.73, 5.44)	6.73 (6.24, 7.23)	11.81 (11.01, 12.65)
Ethiopia	349.82 (311.85, 390.71)	373.64 (332.48, 416.61)	723.46 (644.80, 808.08)	609.29 (549.83, 669.20)	557.83 (500.95, 617.36)	1167.11 (1053.99, 1276.89)	642.40 (632.26, 656.92)	585.38 (575.46, 598.37)	1227.78 (1209.20, 1253.24)	1063.45 (1041.96, 1088.05)	972.61 (950.74, 996.70)	2036.06 (1996.16, 2082.15)
Fiji	3.87 (3.42, 4.32)	2.90 (2.58, 3.26)	6.77 (6.06, 7.48)	6.74 (6.02, 7.56)	4.91 (4.41, 5.45)	11.65 (10.62, 12.80)	6.65 (6.42, 6.85)	4.91 (4.76, 5.09)	11.56 (11.20, 11.90)	7.67 (7.40, 7.94)	5.92 (5.73, 6.13)	13.60 (13.18, 14.01)
Finland	8.80 (7.81, 9.84)	9.62 (8.61, 10.77)	18.42 (16.52, 20.37)	9.90 (8.76, 11.08)	10.13 (9.07, 11.34)	20.04 (18.00, 22.17)	10.80 (10.14, 11.54)	11.56 (10.70, 12.56)	22.36 (20.91, 23.99)	12.83 (12.02, 13.95)	13.51 (12.40, 14.93)	26.34 (24.47, 28.65)
France	98.73 (89.09, 109.68)	100.54 (90.71, 111.65)	199.27 (180.59, 218.37)	117.24 (104.96, 130.20)	121.58 (107.78, 135.90)	238.82 (214.78, 263.96)	125.22 (121.21, 130.30)	132.19 (126.88, 138.25)	257.41 (248.78, 268.46)	154.35 (148.09, 161.87)	160.03 (152.51, 168.87)	314.38 (301.45, 330.82)
Gabon	4.28 (3.74, 4.77)	4.03 (3.54, 4.51)	8.31 (7.32, 9.24)	9.21 (8.24, 10.25)	7.26 (6.46, 8.10)	16.47 (14.86, 18.15)	9.15 (8.89, 9.39)	7.22 (6.97, 7.46)	16.36 (15.96, 16.78)	10.71 (10.16, 11.24)	9.05 (8.61, 9.46)	19.76 (18.87, 20.61)
Germany	146.44 (130.68, 163.74)	159.70 (142.70, 177.48)	306.14 (275.19, 338.83)	220.69 (197.82, 247.37)	235.90 (212.04, 263.34)	456.59 (416.50, 501.69)	219.67 (213.15, 225.76)	240.23 (232.60, 247.82)	459.90 (447.61, 472.36)	253.79 (244.97, 262.47)	267.10 (258.45, 275.91)	520.89 (505.72, 536.55)
Ghana	89.28 (81.76, 97.01)	88.33 (80.56, 96.64)	177.61 (162.52, 192.23)	183.41 (164.92, 203.19)	180.66 (162.69, 198.89)	364.06 (331.82, 398.06)	179.96 (173.15, 186.45)	180.55 (173.29, 187.36)	360.51 (347.30, 372.76)	245.40 (233.10, 257.51)	250.27 (237.83, 262.56)	495.67 (471.36, 518.13)
Greece	19.22 (16.96, 21.45)	18.20 (16.19, 20.41)	37.43 (33.52, 41.62)	18.38 (16.34, 20.65)	18.86 (16.68, 21.13)	37.24 (33.23, 41.45)	18.92 (17.74, 20.32)	19.05 (17.83, 20.40)	37.97 (35.76, 40.46)	19.79 (18.60, 21.08)	19.87 (18.61, 21.30)	39.66 (37.31, 42.15)
Greenland	0.16 (0.13, 0.18)	0.11 (0.09, 0.12)	0.26 (0.23, 0.30)	0.16 (0.14, 0.18)	0.11 (0.10, 0.13)	0.27 (0.24, 0.31)	0.16 (0.15, 0.17)	0.12 (0.11, 0.13)	0.28 (0.27, 0.30)	0.16 (0.15, 0.17)	0.13 (0.12, 0.13)	0.29 (0.27, 0.30)
Grenada	0.37 (0.33, 0.41)	0.35 (0.31, 0.40)	0.72 (0.64, 0.80)	0.61 (0.54, 0.69)	0.54 (0.48, 0.60)	1.15 (1.03, 1.28)	0.71 (0.70, 0.73)	0.60 (0.59, 0.62)	1.31 (1.28, 1.34)	0.75 (0.73, 0.77)	0.63 (0.61, 0.65)	1.38 (1.34, 1.42)
Guam	0.74 (0.65, 0.83)	0.53 (0.47, 0.60)	1.27 (1.14, 1.41)	1.56 (1.39, 1.75)	1.13 (1.00, 1.27)	2.68 (2.42, 3.00)	1.55 (1.51, 1.58)	1.12 (1.09, 1.14)	2.66 (2.61, 2.71)	1.81 (1.76, 1.85)	1.39 (1.35, 1.42)	3.21 (3.13, 3.27)

Guatemala	76.48 (69.39, 83.49)	65.51 (60.07, 71.77)	141.99 (131.76, 153.90)	85.95 (76.64, 95.30)	75.06 (66.77, 83.07)	161.01 (144.65, 177.03)	84.06 (80.47, 87.87)	77.38 (73.24, 81.07)	161.44 (153.90, 168.70)	101.01 (95.92, 106.12)	98.08 (93.21, 103.66)	199.10 (189.35, 208.94)
Guinea	39.21 (34.94, 43.56)	40.76 (36.50, 45.06)	79.97 (71.92, 88.38)	77.59 (69.22, 85.69)	79.73 (70.95, 88.49)	157.32 (140.62, 173.31)	77.64 (75.31, 79.87)	76.50 (74.20, 78.69)	154.15 (149.85, 158.43)	111.57 (106.00, 117.39)	110.41 (104.92, 115.78)	221.98 (211.39, 232.46)
Guinea-Bissau	5.69 (5.09, 6.35)	6.51 (5.83, 7.27)	12.21 (10.96, 13.53)	11.75 (10.55, 13.02)	12.52 (11.21, 13.95)	24.27 (21.89, 26.69)	12.25 (11.93, 12.52)	12.71 (12.43, 12.99)	24.96 (24.45, 25.48)	19.12 (18.65, 19.56)	19.34 (18.86, 19.77)	38.46 (37.58, 39.20)
Guyana	3.08 (2.77, 3.41)	2.73 (2.47, 3.01)	5.81 (5.28, 6.36)	3.70 (3.33, 4.10)	3.37 (3.03, 3.72)	7.07 (6.42, 7.76)	3.81 (3.67, 3.94)	3.44 (3.31, 3.55)	7.25 (7.00, 7.46)	4.43 (4.28, 4.61)	4.08 (3.97, 4.23)	8.51 (8.28, 8.78)
Haiti	35.10 (31.33, 38.87)	33.81 (30.24, 37.59)	68.91 (61.75, 75.88)	71.90 (65.01, 79.35)	68.21 (62.11, 74.63)	140.11 (128.25, 152.79)	71.66 (69.43, 73.93)	65.17 (62.64, 67.37)	136.82 (132.63, 140.79)	86.98 (83.72, 90.20)	78.54 (75.32, 81.78)	165.52 (159.42, 171.40)
Honduras	34.01 (29.78, 38.92)	29.28 (25.73, 33.22)	63.28 (55.68, 71.65)	45.31 (39.80, 51.67)	41.12 (36.43, 46.17)	86.43 (76.86, 97.17)	45.04 (42.98, 46.92)	40.81 (38.93, 42.45)	85.85 (82.29, 89.21)	58.92 (56.22, 61.54)	52.94 (50.75, 55.47)	111.86 (107.12, 116.34)
Hungary	55.35 (47.77, 63.31)	67.93 (59.50, 77.22)	123.27 (108.29, 139.61)	44.65 (39.79, 49.83)	59.34 (52.60, 66.43)	104.00 (93.67, 115.16)	24.82 (22.44, 27.60)	33.25 (30.05, 36.97)	58.07 (52.53, 64.42)	22.38 (20.32, 25.00)	27.66 (24.67, 30.95)	50.04 (45.28, 55.66)
Iceland	0.55 (0.49, 0.62)	0.53 (0.47, 0.60)	1.08 (0.96, 1.20)	0.77 (0.69, 0.86)	0.73 (0.65, 0.81)	1.50 (1.35, 1.65)	0.76 (0.74, 0.81)	0.74 (0.72, 0.77)	1.50 (1.47, 1.57)	0.94 (0.90, 0.98)	0.98 (0.93, 1.04)	1.93 (1.83, 2.02)
India	6777.47 (6042.51, 7565.51)	7411.15 (6638.17, 8186.15)	14188.62 (12689.06, 15767.86)	8140.41 (7408.02, 8937.50)	8657.86 (7928.82, 9460.98)	16798.27 (15330.80, 18386.31)	8069.92 (7682.38, 8430.49)	8517.89 (8120.80, 8861.46)	16587.81 (15825.70, 17234.54)	8346.53 (7771.24, 8928.99)	9046.74 (8434.01, 9614.10)	17393.27 (16267.38, 18440.02)
Indonesia	602.69 (548.99, 660.70)	518.29 (466.43, 570.76)	1120.98 (1015.72, 1230.51)	850.34 (785.40, 928.00)	741.17 (679.60, 807.02)	1591.51 (1462.52, 1732.77)	839.77 (798.18, 887.93)	728.17 (689.52, 767.10)	1567.93 (1500.03, 1648.37)	1106.45 (1037.31, 1188.64)	979.62 (910.64, 1044.81)	2086.07 (1965.47, 2232.49)
Iraq	80.22 (69.52, 90.24)	77.83 (66.86, 88.93)	158.04 (137.04, 178.22)	206.41 (181.61, 232.32)	198.16 (174.73, 223.70)	404.57 (358.84, 452.20)	242.25 (236.19, 248.01)	228.57 (222.91, 233.92)	470.83 (459.41, 480.92)	363.47 (355.71, 370.80)	349.27 (342.21, 356.10)	712.74 (698.66, 725.96)
Ireland	5.95 (5.19, 6.76)	5.58 (4.98, 6.24)	11.53 (10.19, 12.94)	7.84 (6.98, 8.76)	8.05 (7.26, 8.95)	15.88 (14.30, 17.56)	8.12 (7.70, 8.69)	8.47 (8.05, 9.06)	16.59 (15.86, 17.70)	10.93 (10.30, 11.64)	11.64 (11.09, 12.51)	22.58 (21.53, 24.00)
Israel	9.99 (8.81, 11.40)	9.09 (8.03, 10.19)	19.09 (16.94, 21.37)	21.31 (18.96, 23.77)	20.24 (18.13, 22.44)	41.56 (37.33, 45.95)	21.18 (19.76, 22.52)	19.32 (17.77, 20.80)	40.50 (37.56, 43.08)	31.68 (29.46, 34.04)	29.78 (28.09, 31.80)	61.46 (57.91, 65.84)

Italy	74.62 (66.24, 84.16)	76.49 (68.32, 85.66)	151.11 (134.44, 169.82)	70.08 (62.84, 77.02)	73.73 (67.05, 80.61)	143.81 (130.19, 157.68)	75.42 (66.56, 85.18)	86.05 (76.78, 98.69)	161.47 (144.41, 182.26)	86.13 (77.44, 97.53)	93.64 (83.57, 107.84)	179.76 (161.19, 203.59)
Jamaica	8.55 (7.73, 9.38)	7.54 (6.77, 8.34)	16.09 (14.66, 17.62)	12.49 (11.16, 13.89)	11.31 (10.04, 12.50)	23.80 (21.33, 26.15)	11.74 (11.34, 12.11)	10.81 (10.44, 11.15)	22.56 (21.85, 23.23)	12.36 (11.89, 12.81)	12.42 (11.90, 12.87)	24.78 (23.86, 25.58)
Japan	90.02 (78.38, 104.65)	103.42 (89.12, 120.42)	193.44 (168.03, 225.87)	79.05 (69.16, 89.56)	92.79 (82.06, 106.56)	171.84 (152.06, 195.59)	92.35 (78.83, 111.21)	110.53 (91.37, 133.90)	202.88 (172.25, 242.38)	149.01 (123.39, 178.23)	181.92 (148.43, 216.57)	330.93 (276.47, 392.26)
Jordan	14.67 (12.71, 16.65)	13.67 (11.86, 15.68)	28.34 (24.69, 32.18)	54.07 (47.69, 60.71)	49.56 (43.61, 55.49)	103.63 (91.86, 115.65)	53.51 (51.88, 54.71)	49.15 (47.59, 50.18)	102.66 (99.60, 104.68)	78.69 (74.41, 80.55)	73.65 (68.85, 75.32)	152.34 (143.40, 155.55)
Kazakhstan	50.14 (43.86, 56.70)	53.74 (47.43, 60.30)	103.87 (91.74, 115.92)	68.48 (59.92, 77.62)	76.47 (66.87, 86.28)	144.96 (127.67, 163.20)	67.77 (65.73, 69.44)	75.93 (74.02, 77.53)	143.70 (139.84, 146.52)	78.94 (75.83, 81.46)	88.95 (85.69, 91.43)	167.89 (161.74, 172.28)
Kenya	123.64 (111.49, 136.44)	120.57 (108.58, 133.55)	244.21 (220.01, 269.69)	277.72 (251.80, 303.62)	233.17 (211.62, 255.70)	510.89 (463.97, 559.06)	272.74 (266.35, 279.51)	238.39 (231.78, 245.74)	511.13 (499.40, 523.42)	342.99 (319.83, 365.21)	322.05 (298.91, 345.30)	665.04 (621.15, 708.13)
Kiribati	0.44 (0.39, 0.48)	0.38 (0.34, 0.42)	0.81 (0.74, 0.89)	0.91 (0.81, 1.01)	0.75 (0.67, 0.84)	1.66 (1.51, 1.82)	0.90 (0.86, 0.94)	0.75 (0.72, 0.79)	1.66 (1.59, 1.72)	1.20 (1.13, 1.27)	1.05 (0.99, 1.11)	2.25 (2.12, 2.36)
Kuwait	6.25 (5.41, 7.16)	5.37 (4.59, 6.26)	11.61 (10.09, 13.32)	19.16 (16.92, 21.68)	16.36 (14.35, 18.50)	35.52 (31.68, 39.83)	19.53 (19.13, 19.92)	16.52 (16.20, 16.79)	36.05 (35.38, 36.64)	27.87 (26.91, 28.57)	24.28 (23.62, 24.95)	52.16 (50.70, 53.38)
Kyrgyzstan	14.98 (13.14, 16.74)	15.62 (13.75, 17.46)	30.59 (27.02, 34.04)	27.77 (24.27, 31.83)	29.16 (25.51, 33.37)	56.94 (50.03, 64.67)	28.46 (27.74, 29.40)	29.88 (28.84, 31.01)	58.34 (56.74, 60.24)	35.30 (34.27, 36.48)	37.05 (35.82, 38.42)	72.35 (70.34, 74.71)
Latvia	13.11 (11.42, 14.98)	15.26 (13.34, 17.34)	28.36 (25.04, 32.04)	8.85 (7.76, 10.14)	10.58 (9.31, 11.90)	19.42 (17.19, 21.88)	8.59 (8.24, 8.94)	11.06 (10.63, 11.51)	19.65 (18.93, 20.37)	5.29 (4.66, 5.81)	7.43 (6.74, 8.05)	12.72 (11.39, 13.85)
Lebanon	13.92 (12.01, 15.78)	13.16 (11.44, 14.93)	27.08 (23.67, 30.55)	28.18 (25.07, 31.48)	28.79 (25.80, 31.97)	56.97 (50.97, 63.20)	47.17 (45.77, 48.63)	46.05 (44.73, 47.36)	93.22 (90.69, 95.84)	59.01 (56.82, 61.34)	57.23 (55.00, 59.45)	116.24 (111.98, 120.40)
Lesotho	12.06 (10.86, 13.49)	10.09 (9.06, 11.21)	22.15 (20.08, 24.47)	15.01 (13.37, 16.98)	12.36 (11.09, 13.75)	27.38 (24.61, 30.32)	13.10 (12.51, 13.61)	11.18 (10.63, 11.74)	24.28 (23.19, 25.28)	14.58 (13.78, 15.22)	13.21 (12.43, 13.87)	27.80 (26.42, 28.91)
Liberia	8.11 (7.37, 8.91)	8.48 (7.66, 9.41)	16.59 (15.20, 18.19)	29.42 (26.46, 32.43)	27.22 (24.39, 30.29)	56.65 (51.57, 62.19)	31.41 (30.51, 32.39)	28.37 (27.40, 29.17)	59.79 (58.13, 61.32)	46.69 (44.67, 48.61)	40.13 (38.24, 41.72)	86.82 (83.24, 90.14)

Libya	17.78 (15.52, 20.41)	17.23 (14.70, 19.86)	35.01 (30.34, 39.91)	35.32 (31.25, 39.64)	33.69 (29.94, 38.29)	69.01 (61.45, 76.87)	39.22 (37.88, 40.94)	37.73 (36.46, 39.19)	76.94 (74.41, 80.14)	48.39 (46.35, 50.41)	47.71 (45.88, 49.79)	96.10 (92.38, 100.18)
Lithuania	17.14 (14.92, 19.60)	18.99 (16.65, 21.58)	36.13 (31.81, 40.98)	12.15 (10.72, 13.83)	16.58 (14.72, 18.77)	28.73 (25.76, 32.21)	12.63 (12.08, 13.21)	16.73 (15.99, 17.42)	29.36 (28.09, 30.61)	10.29 (9.11, 10.94)	13.69 (12.40, 14.46)	23.99 (21.47, 25.34)
Luxembourg	0.75 (0.66, 0.84)	0.80 (0.72, 0.89)	1.55 (1.39, 1.71)	1.33 (1.20, 1.48)	1.38 (1.25, 1.52)	2.71 (2.47, 2.98)	1.44 (1.38, 1.54)	1.49 (1.41, 1.59)	2.93 (2.80, 3.13)	2.01 (1.94, 2.11)	1.95 (1.87, 2.05)	3.96 (3.82, 4.14)
Madagascar	74.53 (67.05, 82.51)	67.26 (60.26, 74.64)	141.80 (127.69, 155.95)	180.54 (160.84, 202.39)	148.65 (131.69, 165.85)	329.19 (294.75, 365.98)	188.28 (182.24, 194.04)	160.10 (155.12, 164.83)	348.38 (338.24, 357.82)	292.48 (279.22, 304.47)	249.94 (237.50, 260.50)	542.41 (517.93, 563.28)
Malawi	60.12 (53.99, 66.18)	62.27 (56.38, 68.53)	122.39 (111.08, 134.24)	117.57 (104.40, 130.29)	108.49 (97.01, 119.91)	226.06 (203.33, 248.12)	116.68 (112.95, 119.65)	108.25 (104.64, 111.05)	224.92 (217.92, 230.39)	164.55 (157.23, 171.23)	155.57 (147.74, 161.20)	320.12 (306.13, 331.60)
Malaysia	46.51 (40.81, 52.75)	41.20 (35.43, 47.05)	87.70 (76.83, 99.08)	118.26 (106.63, 131.59)	93.64 (83.28, 104.65)	211.89 (191.28, 235.10)	116.86 (112.10, 119.85)	93.26 (89.70, 96.69)	210.13 (202.20, 215.38)	156.39 (146.97, 163.55)	129.17 (120.63, 135.49)	285.56 (268.27, 297.77)
Maldives	0.85 (0.75, 0.94)	0.71 (0.61, 0.80)	1.56 (1.36, 1.74)	1.90 (1.69, 2.12)	1.16 (1.02, 1.30)	3.05 (2.74, 3.37)	1.94 (1.78, 2.11)	1.20 (1.11, 1.31)	3.14 (2.91, 3.40)	3.04 (2.81, 3.33)	2.05 (1.88, 2.28)	5.09 (4.72, 5.58)
Mali	57.43 (52.08, 62.66)	62.09 (56.12, 68.63)	119.52 (108.56, 130.62)	153.97 (138.33, 170.60)	142.99 (126.94, 159.44)	296.95 (265.37, 327.31)	155.30 (152.19, 159.75)	144.27 (140.97, 148.26)	299.56 (293.42, 307.71)	284.43 (274.08, 296.70)	264.14 (254.22, 275.68)	548.57 (529.77, 570.94)
Malta	0.57 (0.50, 0.64)	0.54 (0.47, 0.60)	1.11 (0.98, 1.24)	0.66 (0.59, 0.74)	0.67 (0.60, 0.75)	1.33 (1.20, 1.48)	0.65 (0.62, 0.68)	0.64 (0.61, 0.68)	1.29 (1.24, 1.36)	1.09 (0.92, 1.29)	1.01 (0.84, 1.22)	2.10 (1.78, 2.50)
Marshall Islands	0.24 (0.21, 0.27)	0.20 (0.18, 0.23)	0.45 (0.40, 0.49)	0.40 (0.36, 0.45)	0.30 (0.26, 0.33)	0.70 (0.63, 0.77)	0.41 (0.40, 0.42)	0.30 (0.30, 0.31)	0.71 (0.70, 0.73)	0.50 (0.48, 0.52)	0.39 (0.37, 0.40)	0.89 (0.85, 0.92)
Mauritania	13.36 (11.95, 14.74)	13.57 (12.11, 14.92)	26.93 (24.32, 29.58)	31.56 (28.43, 34.61)	29.28 (26.40, 32.40)	60.84 (55.29, 66.74)	31.36 (30.38, 32.27)	29.54 (28.56, 30.39)	60.90 (59.03, 62.56)	44.06 (41.92, 46.37)	41.59 (39.55, 43.56)	85.65 (81.65, 89.70)
Mauritius	3.31 (2.96, 3.67)	2.73 (2.39, 3.08)	6.04 (5.43, 6.68)	5.16 (4.67, 5.70)	4.30 (3.84, 4.78)	9.46 (8.62, 10.40)	5.11 (4.89, 5.35)	4.57 (4.35, 4.83)	9.68 (9.26, 10.13)	6.53 (6.20, 6.87)	6.31 (5.99, 6.64)	12.84 (12.24, 13.47)
Mexico	382.50 (344.63, 421.99)	335.05 (302.68, 369.14)	717.55 (647.77, 788.16)	299.91 (267.11, 336.18)	298.93 (268.91, 332.15)	598.84 (536.15, 668.05)	303.70 (272.14, 335.50)	289.68 (258.53, 321.80)	593.38 (536.71, 651.52)	541.56 (487.65, 590.95)	550.18 (495.27, 605.65)	1091.74 (982.78, 1193.31)
Mongolia	10.65 (9.26, 12.15)	10.88 (9.46, 12.41)	21.52 (18.84, 24.47)	15.78 (13.63, 17.91)	16.77 (14.53, 18.96)	32.55 (28.50, 36.67)	15.75 (15.34, 16.09)	16.74 (16.34, 17.11)	32.50 (31.74, 33.16)	17.92 (17.15, 18.68)	19.27 (18.54, 19.99)	37.19 (35.76, 38.58)
Montenegro	2.90 (2.51, 3.33)	3.18 (2.78, 3.60)	6.08 (5.33, 6.91)	2.07 (1.82, 2.37)	2.55 (2.24, 2.90)	4.63 (4.09, 5.22)	2.27 (2.14, 2.43)	2.74 (2.62, 2.88)	5.01 (4.78, 5.29)	2.33 (2.19, 2.49)	2.78 (2.64, 2.93)	5.11 (4.87, 5.39)

Morocco	113.64 (101.55, 126.42)	112.60 (100.58, 125.56)	226.24 (202.90, 249.75)	175.69 (155.53, 198.09)	170.48 (151.55, 191.23)	346.17 (308.59, 386.36)	177.99 (174.80, 181.52)	172.32 (169.35, 175.12)	350.31 (344.27, 355.52)	215.99 (210.81, 220.65)	211.43 (206.37, 216.01)	427.41 (418.29, 436.29)
Mozambique	71.85 (64.09, 79.33)	74.95 (67.09, 83.41)	146.80 (131.81, 161.51)	156.49 (139.07, 174.30)	136.34 (121.90, 152.95)	292.83 (262.96, 325.00)	160.27 (155.22, 165.17)	141.87 (136.77, 147.44)	302.13 (293.23, 311.24)	232.74 (224.84, 240.30)	211.18 (202.94, 219.16)	443.91 (429.42, 458.32)
Myanmar	168.86 (153.61, 183.20)	151.68 (134.88, 167.04)	320.54 (292.72, 348.13)	193.21 (175.49, 214.28)	172.61 (155.93, 190.12)	365.83 (335.88, 399.68)	187.65 (179.63, 199.56)	174.33 (165.95, 188.44)	361.99 (346.46, 386.87)	237.10 (226.89, 251.57)	225.74 (213.43, 243.51)	462.84 (441.53, 493.35)
Namibia	9.94 (8.95, 11.02)	8.67 (7.78, 9.64)	18.61 (16.91, 20.47)	16.70 (14.74, 18.75)	13.66 (12.32, 15.17)	30.36 (27.28, 33.48)	16.76 (16.34, 17.10)	14.57 (14.22, 14.96)	31.33 (30.70, 31.98)	21.23 (20.51, 21.82)	19.97 (19.39, 20.54)	41.20 (40.08, 42.17)
Nepal	159.77 (141.18, 179.96)	187.66 (164.59, 210.26)	347.43 (308.51, 387.31)	197.18 (176.76, 219.27)	233.48 (209.49, 259.35)	430.66 (389.31, 475.47)	198.49 (190.82, 203.99)	234.21 (225.43, 241.46)	432.71 (417.05, 444.26)	208.84 (196.39, 220.02)	264.82 (249.51, 277.41)	473.66 (447.69, 495.46)
Netherlands	22.41 (19.90, 24.98)	22.09 (19.84, 24.65)	44.51 (39.88, 49.26)	31.08 (27.98, 34.68)	30.82 (27.75, 34.48)	61.90 (56.07, 68.42)	33.07 (31.78, 35.05)	33.26 (31.86, 35.79)	66.32 (63.77, 70.58)	38.98 (37.78, 40.31)	37.76 (36.37, 39.02)	76.74 (74.19, 79.20)
New Zealand	7.36 (6.55, 8.31)	7.34 (6.52, 8.23)	14.70 (13.21, 16.47)	10.70 (9.71, 11.79)	10.78 (9.86, 11.79)	21.48 (19.71, 23.38)	9.89 (9.12, 10.57)	9.92 (9.13, 10.73)	19.81 (18.31, 21.22)	12.06 (11.08, 12.99)	12.56 (11.50, 13.66)	24.61 (22.70, 26.54)
Nicaragua	23.26 (20.45, 26.56)	20.78 (18.04, 23.52)	44.04 (38.80, 49.37)	26.24 (22.64, 29.94)	23.51 (20.61, 26.78)	49.75 (43.80, 56.20)	29.74 (28.76, 30.86)	26.67 (25.64, 27.66)	56.41 (54.59, 58.40)	39.05 (37.10, 41.25)	35.65 (34.07, 37.28)	74.70 (71.39, 78.27)
Niger	56.51 (51.04, 61.89)	60.35 (54.44, 66.65)	116.86 (106.08, 127.94)	181.05 (161.46, 199.26)	180.16 (158.73, 201.58)	361.21 (321.11, 399.50)	183.83 (180.18, 189.34)	181.15 (177.21, 185.43)	364.98 (357.84, 374.44)	399.65 (383.43, 418.90)	390.55 (376.78, 406.73)	790.20 (761.06, 824.80)
Nigeria	555.54 (502.51, 612.08)	599.15 (536.40, 661.15)	1154.69 (1039.28, 1273.82)	1162.80 (1045.98, 1283.69)	1282.22 (1154.08, 1414.49)	2445.02 (2202.29, 2696.38)	1246.06 (1218.87, 1272.70)	1324.88 (1295.67, 1353.34)	2570.94 (2519.09, 2620.55)	2348.10 (2282.65, 2419.84)	2450.13 (2387.89, 2521.00)	4798.23 (4674.50, 4934.53)
Northern Mariana Islands	0.22 (0.19, 0.24)	0.14 (0.12, 0.16)	0.36 (0.32, 0.39)	0.41 (0.37, 0.47)	0.27 (0.24, 0.31)	0.69 (0.62, 0.76)	0.42 (0.41, 0.43)	0.29 (0.28, 0.30)	0.71 (0.69, 0.73)	0.48 (0.46, 0.49)	0.39 (0.38, 0.40)	0.87 (0.84, 0.88)
Norway	8.88 (8.02, 9.84)	9.40 (8.54, 10.38)	18.29 (16.60, 20.21)	12.71 (11.57, 13.96)	12.58 (11.43, 13.72)	25.29 (23.06, 27.61)	13.07 (12.40, 13.93)	12.48 (11.84, 13.27)	25.55 (24.31, 27.16)	17.51 (16.63, 18.56)	16.31 (15.38, 17.43)	33.82 (32.16, 35.90)

Oman	8.17 (7.11, 9.23)	6.69 (5.73, 7.66)	14.87 (12.89, 16.78)	26.92 (23.41, 30.76)	17.12 (15.06, 19.37)	44.04 (38.73, 49.82)	28.28 (26.25, 29.28)	17.88 (16.90, 18.43)	46.15 (43.12, 47.59)	39.95 (38.47, 41.37)	25.05 (24.24, 26.02)	65.00 (62.97, 67.29)
Pakistan	976.29 (863.41, 1094.73)	978.43 (866.02, 1092.91)	1954.72 (1739.49, 2183.50)	1533.21 (1357.03, 1723.91)	1473.74 (1316.67, 1638.18)	3006.95 (2696.73, 3352.12)	1503.69 (1441.65, 1563.39)	1406.67 (1346.33, 1466.48)	2910.36 (2800.94, 3029.38)	1864.81 (1779.73, 1945.04)	1752.87 (1673.82, 1831.04)	3617.67 (3467.43, 3770.74)
Palestine	8.79 (7.52, 10.16)	8.59 (7.33, 10.05)	17.38 (14.97, 20.09)	23.36 (20.40, 26.62)	22.24 (19.40, 25.39)	45.60 (39.93, 51.88)	28.45 (27.11, 29.79)	24.85 (23.82, 25.74)	53.30 (51.01, 55.33)	42.11 (39.89, 44.14)	35.46 (33.98, 36.86)	77.56 (74.45, 80.68)
Panama	12.21 (10.77, 13.76)	9.88 (8.79, 10.93)	22.10 (19.76, 24.56)	17.83 (15.70, 19.97)	15.66 (14.01, 17.60)	33.49 (29.99, 37.28)	17.96 (17.35, 18.68)	15.74 (15.19, 16.42)	33.70 (32.65, 35.04)	25.32 (24.39, 26.44)	23.18 (22.24, 24.14)	48.50 (46.70, 50.39)
Papua New Guinea	24.62 (22.31, 27.33)	18.26 (16.40, 20.16)	42.88 (38.90, 47.06)	78.74 (70.59, 87.53)	54.63 (48.44, 60.86)	133.37 (120.63, 146.46)	76.14 (74.75, 77.29)	53.19 (51.82, 54.33)	129.33 (127.02, 131.32)	109.47 (106.74, 111.76)	82.26 (79.92, 84.59)	191.74 (187.59, 195.76)
Paraguay	15.59 (13.95, 17.39)	14.13 (12.59, 15.69)	29.72 (26.59, 32.85)	30.49 (26.93, 34.06)	29.24 (26.01, 32.84)	59.73 (53.40, 66.36)	32.61 (31.67, 33.66)	29.87 (29.00, 31.00)	62.48 (60.65, 64.46)	41.36 (40.10, 42.54)	39.43 (38.35, 40.49)	80.79 (78.66, 82.75)
Peru	87.37 (80.02, 94.81)	83.55 (76.45, 91.01)	170.92 (158.34, 184.57)	184.42 (167.31, 204.15)	172.88 (157.42, 189.91)	357.30 (326.36, 390.70)	184.07 (176.11, 190.33)	171.59 (164.11, 178.28)	355.67 (341.04, 367.65)	238.36 (227.68, 247.60)	222.01 (211.26, 230.58)	460.37 (440.23, 477.37)
Philippines	264.55 (237.47, 291.34)	215.20 (191.64, 237.13)	479.75 (428.35, 528.30)	414.54 (377.60, 454.38)	361.78 (329.14, 396.89)	776.32 (710.07, 848.83)	395.42 (381.76, 408.71)	344.79 (332.60, 358.34)	740.21 (716.30, 765.18)	507.44 (478.47, 538.23)	446.55 (415.38, 475.68)	953.99 (895.58, 1009.61)
Poland	138.91 (119.36, 159.07)	157.25 (137.23, 178.63)	296.16 (257.14, 337.28)	94.12 (86.34, 102.65)	121.19 (111.72, 131.33)	215.31 (199.34, 232.98)	114.62 (107.25, 124.11)	140.58 (132.73, 152.09)	255.20 (240.87, 276.39)	96.38 (89.23, 105.20)	120.77 (113.25, 130.12)	217.16 (202.21, 234.59)
Portugal	15.82 (14.05, 17.51)	15.40 (13.76, 17.04)	31.22 (27.98, 34.45)	16.16 (14.55, 17.80)	18.34 (16.47, 20.35)	34.50 (31.20, 37.67)	17.11 (15.82, 18.58)	18.92 (17.46, 20.82)	36.03 (33.51, 39.10)	18.49 (16.75, 20.99)	21.44 (18.76, 25.50)	39.92 (35.59, 45.99)
Puerto Rico	12.64 (11.21, 14.16)	12.45 (10.98, 13.98)	25.09 (22.34, 27.97)	20.59 (18.87, 22.46)	23.14 (21.30, 25.39)	43.73 (40.42, 47.47)	20.86 (20.16, 21.35)	23.41 (22.59, 23.85)	44.27 (42.72, 45.14)	21.74 (20.80, 22.22)	25.82 (24.09, 26.33)	47.55 (45.08, 48.45)
Qatar	1.96 (1.71, 2.23)	1.26 (1.07, 1.46)	3.23 (2.82, 3.66)	12.35 (10.66, 14.25)	5.05 (4.42, 5.76)	17.41 (15.27, 19.82)	12.77 (12.33, 13.22)	5.28 (5.13, 5.41)	18.05 (17.55, 18.58)	22.30 (21.10, 23.44)	8.52 (8.14, 8.88)	30.82 (29.37, 32.14)

Romania	119.91 (104.47, 135.69)	143.67 (125.36, 164.07)	263.58 (231.80, 298.04)	72.96 (64.39, 82.40)	95.21 (83.70, 107.36)	168.16 (148.93, 188.73)	78.15 (74.32, 82.68)	97.97 (93.69, 103.10)	176.12 (168.94, 185.36)	64.52 (61.24, 68.57)	80.90 (76.75, 85.90)	145.43 (138.56, 154.00)
Russian Federation	712.23 (631.63, 792.60)	823.61 (736.60, 918.91)	1535.84 (1371.55, 1707.66)	676.48 (601.17, 757.10)	876.28 (783.47, 982.74)	1552.76 (1393.19, 1742.09)	672.21 (662.31, 681.56)	861.42 (843.58, 875.91)	1533.62 (1509.05, 1555.19)	636.37 (604.06, 661.02)	794.07 (755.31, 830.11)	1430.45 (1365.73, 1485.40)
Rwanda	45.04 (40.32, 50.15)	49.64 (44.24, 55.36)	94.68 (84.74, 104.83)	70.90 (63.33, 78.60)	65.26 (58.93, 72.15)	136.16 (123.12, 149.29)	77.88 (75.88, 80.10)	72.05 (69.88, 74.57)	149.92 (146.25, 154.39)	128.71 (124.23, 133.82)	118.93 (114.38, 124.73)	247.64 (239.26, 258.18)
Saint Lucia	0.55 (0.49, 0.61)	0.51 (0.45, 0.57)	1.06 (0.94, 1.17)	1.05 (0.95, 1.18)	0.98 (0.88, 1.09)	2.04 (1.84, 2.24)	1.09 (1.07, 1.11)	0.99 (0.97, 1.01)	2.08 (2.04, 2.12)	1.26 (1.22, 1.29)	1.18 (1.14, 1.21)	2.43 (2.36, 2.50)
Saint Vincent and the Grenadines	0.42 (0.37, 0.47)	0.38 (0.34, 0.42)	0.81 (0.73, 0.89)	0.63 (0.57, 0.70)	0.55 (0.49, 0.60)	1.18 (1.08, 1.30)	0.65 (0.63, 0.66)	0.56 (0.55, 0.58)	1.21 (1.19, 1.24)	0.67 (0.65, 0.69)	0.64 (0.62, 0.65)	1.31 (1.28, 1.34)
Samoa	0.71 (0.63, 0.80)	0.54 (0.48, 0.61)	1.25 (1.12, 1.39)	1.54 (1.37, 1.74)	1.14 (1.02, 1.27)	2.68 (2.41, 2.98)	1.52 (1.48, 1.55)	1.16 (1.13, 1.18)	2.67 (2.62, 2.73)	2.20 (2.13, 2.25)	1.66 (1.60, 1.71)	3.86 (3.74, 3.96)
Sao Tome and Principe	0.64 (0.57, 0.71)	0.69 (0.62, 0.77)	1.33 (1.20, 1.46)	1.15 (1.03, 1.28)	1.14 (1.02, 1.27)	2.29 (2.07, 2.52)	1.15 (1.13, 1.18)	1.14 (1.12, 1.17)	2.29 (2.26, 2.35)	1.55 (1.50, 1.59)	1.50 (1.47, 1.54)	3.05 (2.98, 3.12)
Saudi Arabia	79.92 (70.61, 90.13)	68.56 (59.20, 78.52)	148.49 (130.65, 167.27)	169.23 (149.67, 190.74)	127.51 (111.84, 143.06)	296.74 (264.13, 332.73)	173.42 (170.74, 176.62)	133.45 (131.52, 135.76)	306.87 (302.57, 311.96)	244.58 (238.79, 257.32)	190.94 (186.71, 197.58)	435.52 (426.35, 453.84)
Senegal	53.42 (48.85, 58.74)	53.93 (48.87, 59.25)	107.36 (98.30, 117.63)	119.43 (109.21, 129.47)	105.66 (96.63, 115.45)	225.09 (206.79, 243.89)	121.36 (118.67, 123.34)	111.61 (109.47, 113.49)	232.98 (228.60, 236.11)	177.05 (172.81, 181.03)	165.58 (161.79, 168.79)	342.63 (335.39, 349.10)
Serbia	43.77 (37.92, 49.52)	45.54 (39.75, 51.71)	89.31 (78.31, 100.31)	32.92 (29.17, 37.04)	33.89 (29.98, 38.47)	66.81 (59.63, 74.96)	33.98 (32.68, 36.23)	36.43 (34.81, 39.21)	70.42 (67.59, 75.42)	29.87 (28.41, 32.84)	32.86 (30.90, 36.46)	62.73 (59.54, 69.02)
Seychelles	0.20 (0.17, 0.22)	0.18 (0.16, 0.20)	0.37 (0.33, 0.42)	0.37 (0.34, 0.41)	0.29 (0.26, 0.32)	0.66 (0.60, 0.73)	0.50 (0.47, 0.54)	0.42 (0.39, 0.45)	0.93 (0.87, 0.99)	0.59 (0.54, 0.66)	0.51 (0.46, 0.55)	1.10 (1.01, 1.21)
Sierra Leone	22.75 (20.43, 25.04)	22.03 (19.74, 24.38)	44.78 (40.45, 49.07)	58.19 (51.73, 64.15)	50.97 (45.34, 57.00)	109.16 (97.91, 120.07)	57.30 (55.66, 58.75)	50.36 (48.82, 51.89)	107.66 (104.96, 110.53)	84.09 (81.08, 87.07)	72.44 (69.52, 74.83)	156.53 (151.15, 161.49)
Singapore	2.53 (2.17, 2.94)	2.85 (2.48, 3.28)	5.39 (4.77, 6.09)	6.50 (5.63, 7.58)	6.58 (5.66, 7.71)	13.07 (11.56, 15.09)	6.38 (5.78, 7.39)	7.14 (6.61, 8.00)	13.53 (12.51, 15.15)	8.95 (7.76, 10.27)	9.33 (8.27, 10.45)	18.28 (16.09, 20.48)
Slovakia	31.10 (26.97, 35.68)	29.59 (25.85, 33.83)	60.69 (53.22, 69.07)	23.61 (20.73, 26.70)	23.98 (21.20, 26.97)	47.59 (42.40, 53.25)	13.43 (12.14, 15.38)	14.45 (12.95, 16.57)	27.88 (25.05, 31.93)	13.48 (12.29, 14.95)	15.52 (14.07, 17.25)	29.00 (26.42, 32.01)

Slovenia	8.71 (7.61, 9.94)	9.96 (8.76, 11.30)	18.68 (16.47, 21.13)	7.07 (6.21, 8.02)	8.28 (7.28, 9.38)	15.34 (13.64, 17.21)	7.31 (7.08, 7.61)	7.92 (7.60, 8.23)	15.24 (14.70, 15.73)	6.65 (6.34, 6.98)	7.11 (6.73, 7.46)	13.76 (13.12, 14.37)
Solomon Islands	2.55 (2.27, 2.84)	1.95 (1.74, 2.17)	4.50 (4.07, 4.97)	5.84 (5.35, 6.34)	4.71 (4.21, 5.27)	10.56 (9.62, 11.53)	5.85 (5.69, 5.95)	4.66 (4.52, 4.78)	10.51 (10.24, 10.71)	7.89 (7.61, 8.12)	6.32 (6.08, 6.54)	14.21 (13.71, 14.62)
Somalia	44.54 (39.11, 49.85)	45.47 (40.27, 51.37)	90.01 (79.57, 100.38)	138.76 (121.70, 155.84)	130.67 (114.58, 147.14)	269.44 (236.79, 300.32)	122.76 (119.94, 125.50)	115.59 (113.14, 118.09)	238.34 (233.50, 243.26)	206.74 (199.47, 214.01)	191.05 (184.99, 197.55)	397.79 (384.92, 410.75)
South Africa	174.79 (158.23, 191.66)	180.87 (164.21, 198.53)	355.67 (322.75, 389.21)	244.26 (222.77, 267.59)	243.31 (223.99, 265.95)	487.57 (447.73, 532.75)	249.17 (241.51, 258.23)	251.46 (244.53, 259.58)	500.62 (487.48, 516.10)	342.00 (330.88, 356.61)	343.17 (330.84, 356.28)	685.18 (665.41, 712.48)
South Sudan	40.21 (35.94, 44.65)	36.85 (32.99, 41.01)	77.07 (69.26, 84.96)	71.33 (63.47, 79.32)	64.56 (57.19, 72.27)	135.89 (121.34, 151.09)	80.59 (78.05, 82.51)	71.75 (69.63, 73.43)	152.33 (147.71, 155.72)	142.34 (135.83, 147.71)	130.98 (125.20, 136.25)	273.32 (261.15, 284.12)
Spain	70.46 (62.64, 79.03)	68.71 (61.63, 76.65)	139.17 (125.40, 154.33)	91.10 (81.53, 100.56)	98.43 (88.86, 109.42)	189.52 (172.88, 208.51)	97.21 (91.36, 107.21)	103.18 (96.08, 114.31)	200.39 (188.01, 222.68)	107.76 (101.04, 117.67)	112.74 (104.54, 125.24)	220.50 (206.09, 241.99)
Sri Lanka	49.82 (45.60, 54.85)	39.21 (35.17, 43.60)	89.02 (81.47, 97.36)	78.17 (70.41, 85.30)	71.59 (64.43, 79.07)	149.76 (136.29, 164.15)	77.10 (74.54, 79.62)	73.22 (70.34, 75.91)	150.32 (145.46, 155.13)	101.77 (98.83, 104.96)	104.63 (101.63, 108.23)	206.40 (200.85, 212.45)
Sudan	117.72 (106.21, 131.00)	110.52 (99.11, 123.54)	228.25 (205.29, 253.03)	280.23 (250.12, 312.38)	256.45 (226.47, 288.97)	536.68 (479.39, 598.46)	290.38 (278.54, 299.01)	267.90 (257.06, 275.96)	558.28 (536.23, 573.77)	388.73 (363.78, 413.40)	361.00 (338.51, 383.96)	749.73 (704.44, 795.40)
Suriname	1.60 (1.43, 1.77)	1.35 (1.21, 1.48)	2.95 (2.66, 3.23)	3.05 (2.73, 3.38)	2.87 (2.60, 3.16)	5.92 (5.40, 6.49)	3.09 (3.02, 3.17)	2.91 (2.83, 2.98)	6.01 (5.86, 6.15)	3.66 (3.56, 3.79)	3.52 (3.42, 3.63)	7.18 (7.01, 7.39)
Sweden	17.46 (15.53, 19.41)	18.37 (16.46, 20.54)	35.83 (32.17, 39.81)	28.38 (25.44, 31.58)	27.84 (25.04, 30.99)	56.22 (50.89, 62.06)	28.04 (27.14, 28.78)	27.69 (26.76, 28.45)	55.73 (54.04, 57.08)	33.50 (32.07, 34.73)	31.76 (30.31, 32.95)	65.27 (62.37, 67.55)
Switzerland	11.60 (10.26, 12.84)	12.28 (10.97, 13.62)	23.88 (21.46, 26.24)	16.87 (15.18, 18.78)	18.44 (16.58, 20.47)	35.31 (32.16, 38.73)	16.78 (16.24, 17.40)	17.90 (17.14, 18.59)	34.68 (33.56, 35.81)	21.61 (20.52, 22.81)	21.71 (20.37, 23.00)	43.32 (40.97, 45.59)
Tajikistan	26.04 (22.91, 29.31)	26.11 (22.87, 29.32)	52.16 (45.97, 58.56)	49.80 (43.61, 56.17)	50.91 (44.18, 58.27)	100.71 (89.15, 113.39)	50.73 (49.13, 52.11)	51.94 (50.27, 53.30)	102.67 (99.56, 105.18)	69.34 (66.62, 71.71)	71.34 (68.55, 73.81)	140.68 (135.25, 144.96)
Thailand	153.65 (137.61, 170.54)	130.37 (116.30, 145.21)	284.01 (255.94, 314.40)	285.70 (260.19, 315.84)	229.50 (205.51, 255.34)	515.20 (473.06, 563.48)	273.22 (253.21, 291.49)	219.04 (199.94, 238.58)	492.25 (455.31, 527.52)	375.40 (356.05, 394.60)	338.17 (315.37, 361.95)	713.57 (674.90, 754.36)

Timor-Leste	3.59 (3.25, 3.90)	3.20 (2.91, 3.46)	6.79 (6.20, 7.31)	5.74 (5.15, 6.32)	4.52 (4.02, 5.01)	10.26 (9.25, 11.23)	6.47 (6.08, 6.85)	5.31 (4.93, 5.69)	11.78 (11.08, 12.47)	8.48 (7.85, 9.08)	7.36 (6.74, 7.90)	15.83 (14.67, 16.94)
Togo	22.99 (20.67, 25.34)	24.16 (21.75, 26.48)	47.15 (42.73, 51.50)	55.68 (49.29, 61.97)	55.99 (49.97, 61.91)	111.67 (100.11, 123.16)	54.57 (53.40, 55.58)	54.70 (53.60, 55.67)	109.28 (107.28, 111.16)	75.38 (73.69, 77.10)	74.21 (72.63, 75.81)	149.60 (146.49, 152.75)
Tonga	0.42 (0.37, 0.48)	0.36 (0.31, 0.40)	0.78 (0.69, 0.87)	0.72 (0.64, 0.81)	0.57 (0.51, 0.64)	1.29 (1.15, 1.43)	0.74 (0.72, 0.75)	0.59 (0.58, 0.60)	1.32 (1.30, 1.34)	1.04 (1.02, 1.07)	0.86 (0.84, 0.88)	1.90 (1.86, 1.94)
Trinidad and Tobago	3.43 (3.07, 3.81)	3.02 (2.70, 3.34)	6.45 (5.79, 7.08)	6.06 (5.40, 6.79)	5.50 (4.90, 6.08)	11.55 (10.42, 12.77)	5.90 (5.66, 6.13)	5.43 (5.22, 5.64)	11.33 (10.89, 11.75)	6.83 (6.54, 7.09)	6.98 (6.69, 7.21)	13.80 (13.26, 14.25)
Tunisia	33.94 (29.86, 38.22)	32.88 (28.96, 37.11)	66.81 (58.99, 75.03)	55.67 (50.09, 61.75)	55.80 (50.14, 61.72)	111.46 (100.88, 122.96)	55.58 (53.51, 56.73)	55.38 (52.76, 56.57)	110.95 (106.44, 112.97)	67.40 (61.30, 69.07)	71.45 (63.07, 73.12)	138.85 (124.42, 141.94)
Turkey	224.59 (197.68, 252.28)	210.59 (185.39, 237.10)	435.17 (384.40, 486.46)	419.54 (374.03, 474.45)	411.32 (369.56, 459.07)	830.86 (747.76, 922.18)	406.66 (388.80, 425.80)	421.88 (401.39, 440.43)	828.53 (791.06, 861.96)	495.34 (466.08, 520.03)	545.60 (512.18, 575.35)	1040.94 (981.70, 1095.36)
Turkmenistan	14.54 (12.96, 16.25)	14.23 (12.60, 15.89)	28.77 (25.81, 32.05)	19.52 (16.95, 22.22)	19.68 (17.19, 22.26)	39.20 (34.41, 44.23)	19.98 (19.43, 20.70)	19.98 (19.38, 20.65)	39.95 (39.00, 41.24)	25.30 (23.68, 26.86)	26.66 (24.80, 28.58)	51.96 (48.55, 55.28)
Uganda	104.02 (93.99, 113.90)	104.31 (93.91, 114.49)	208.34 (189.10, 227.66)	235.25 (209.36, 261.79)	210.58 (187.85, 233.03)	445.83 (400.62, 492.80)	235.97 (227.54, 243.34)	211.78 (203.92, 219.98)	447.75 (432.97, 461.50)	392.90 (377.88, 405.81)	360.36 (346.64, 374.13)	753.26 (725.97, 777.99)
Ukraine	179.59 (158.27, 202.97)	205.41 (181.13, 231.47)	385.00 (343.63, 432.63)	165.78 (145.94, 189.38)	202.45 (176.55, 227.51)	368.23 (327.51, 415.26)	159.78 (153.50, 165.54)	199.18 (192.96, 205.52)	358.96 (347.03, 370.15)	133.49 (128.49, 138.35)	170.82 (163.99, 177.09)	304.30 (292.94, 315.00)
United Arab Emirates	7.48 (6.61, 8.42)	5.08 (4.39, 5.80)	12.56 (11.08, 14.14)	48.14 (41.71, 55.69)	20.87 (18.20, 23.90)	69.02 (60.49, 78.32)	51.52 (48.69, 53.62)	21.73 (20.75, 22.36)	73.25 (69.38, 75.74)	57.02 (49.24, 66.59)	17.35 (14.79, 20.60)	74.37 (64.56, 86.52)
United Kingdom	94.50 (84.70, 104.68)	96.10 (86.83, 105.80)	190.60 (172.16, 210.25)	120.40 (108.79, 132.24)	123.20 (112.19, 134.32)	243.60 (221.22, 266.33)	121.87 (119.08, 124.65)	125.05 (121.82, 128.43)	246.93 (241.48, 252.21)	147.53 (143.51, 152.59)	150.09 (145.85, 155.38)	297.62 (290.39, 307.17)
Uruguay	6.65 (5.91, 7.40)	5.44 (4.85, 6.00)	12.09 (10.85, 13.30)	8.38 (7.40, 9.33)	7.09 (6.34, 7.88)	15.47 (13.95, 17.07)	8.68 (8.25, 9.22)	7.98 (7.50, 8.59)	16.66 (15.79, 17.79)	9.40 (8.50, 10.65)	9.33 (7.99, 11.19)	18.73 (16.60, 21.77)
Uzbekistan	76.66 (67.11, 85.94)	76.83 (68.07, 85.94)	153.49 (136.15, 171.16)	135.70 (117.21, 155.23)	141.82 (123.38, 161.22)	277.51 (242.80, 315.41)	135.68 (129.34, 141.54)	140.23 (132.79, 147.74)	275.91 (262.53, 288.70)	148.07 (142.22, 153.93)	152.55 (146.06, 159.03)	300.62 (288.79, 312.53)
Vanuatu	0.90 (0.80, 1.00)	0.72 (0.64, 0.80)	1.61 (1.45, 1.78)	2.30 (2.07, 2.55)	1.77 (1.58, 1.97)	4.07 (3.69, 4.51)	2.30 (2.23, 2.35)	1.78 (1.73, 1.82)	4.08 (3.96, 4.15)	3.22 (3.12, 3.34)	2.59 (2.51, 2.68)	5.81 (5.64, 6.01)

Yemen	87.64 (77.83, 99.11)	87.64 (78.04, 99.88)	175.28 (156.48, 197.25)	210.73 (182.44, 240.31)	211.98 (183.54, 242.38)	422.71 (369.94, 480.05)	229.86 (224.50, 234.84)	226.01 (221.55, 230.31)	455.87 (447.49, 463.55)	297.55 (284.13, 311.21)	294.75 (281.69, 307.49)	592.30 (568.91, 616.33)
Zambia	51.78 (45.88, 57.50)	53.92 (47.96, 59.77)	105.71 (94.44, 116.47)	109.68 (97.97, 122.46)	101.29 (90.09, 112.90)	210.97 (189.96, 233.55)	108.68 (105.14, 111.88)	101.28 (97.85, 104.63)	209.96 (203.80, 215.89)	154.48 (147.69, 160.69)	148.26 (141.03, 154.72)	302.73 (289.50, 314.76)
Zimbabwe	61.26 (54.55, 67.93)	48.33 (42.92, 53.77)	109.59 (98.44, 121.23)	99.75 (89.09, 111.04)	82.69 (74.00, 92.29)	182.44 (164.64, 202.09)	102.63 (99.60, 105.36)	85.82 (83.04, 88.58)	188.44 (183.29, 192.89)	149.65 (145.76, 153.53)	127.42 (123.82, 130.92)	277.07 (270.66, 283.31)
Bolivia (Plurinational State of)	29.73 (26.43, 33.33)	29.23 (25.72, 32.98)	58.96 (52.73, 65.54)	68.22 (60.08, 76.09)	64.39 (58.01, 71.31)	132.61 (118.76, 146.84)	69.56 (67.14, 72.07)	65.95 (63.79, 68.01)	135.51 (131.21, 139.63)	89.39 (85.64, 93.51)	86.18 (82.59, 89.51)	175.57 (169.00, 182.20)
Brunei Darussalam	0.17 (0.15, 0.20)	0.16 (0.14, 0.18)	0.33 (0.29, 0.37)	0.38 (0.33, 0.43)	0.38 (0.33, 0.44)	0.75 (0.66, 0.85)	0.39 (0.33, 0.45)	0.39 (0.34, 0.44)	0.78 (0.68, 0.89)	0.59 (0.53, 0.69)	0.55 (0.50, 0.64)	1.14 (1.04, 1.34)
Cabo Verde	1.92 (1.72, 2.13)	2.07 (1.87, 2.26)	4.00 (3.62, 4.36)	3.26 (2.91, 3.65)	3.06 (2.76, 3.40)	6.32 (5.72, 6.98)	3.07 (2.89, 3.24)	2.88 (2.72, 3.02)	5.95 (5.62, 6.27)	4.26 (4.07, 4.45)	3.77 (3.61, 3.92)	8.04 (7.73, 8.35)
Democratic Republic of the Congo	169.07 (149.13, 188.45)	154.16 (133.96, 174.66)	323.23 (284.88, 360.18)	517.87 (465.10, 574.15)	416.67 (370.73, 466.06)	934.54 (839.16, 1032.56)	520.53 (511.43, 529.57)	423.28 (415.15, 432.01)	943.81 (929.67, 959.06)	870.87 (836.36, 907.85)	735.41 (699.83, 779.92)	1606.27 (1543.71, 1686.72)
Cote d'Ivoire	69.83 (62.50, 77.30)	68.58 (61.04, 76.25)	138.41 (124.30, 152.89)	172.56 (155.76, 189.77)	159.85 (142.83, 176.88)	332.41 (302.28, 363.30)	160.88 (151.35, 168.70)	148.70 (140.55, 156.03)	309.57 (292.33, 324.68)	235.32 (221.09, 249.14)	216.38 (202.43, 230.91)	451.70 (423.77, 479.42)
Czechia	48.95 (42.45, 55.64)	55.96 (48.96, 63.82)	104.91 (91.87, 118.55)	45.33 (40.25, 50.53)	54.11 (48.55, 60.56)	99.44 (89.58, 110.03)	40.27 (37.14, 43.13)	47.90 (44.68, 51.06)	88.17 (81.54, 94.00)	37.66 (34.78, 40.29)	43.61 (40.11, 46.67)	81.27 (75.03, 86.72)
Micronesia (Federated States of)	0.52 (0.46, 0.59)	0.44 (0.39, 0.49)	0.96 (0.86, 1.07)	0.70 (0.62, 0.79)	0.53 (0.47, 0.59)	1.23 (1.11, 1.37)	0.73 (0.71, 0.74)	0.56 (0.54, 0.57)	1.29 (1.26, 1.31)	0.96 (0.92, 0.99)	0.75 (0.73, 0.78)	1.71 (1.66, 1.76)
Georgia	19.97 (17.50, 22.42)	22.17 (19.39, 24.90)	42.14 (37.34, 47.03)	17.08 (14.94, 19.60)	18.63 (16.43, 21.08)	35.71 (31.70, 40.22)	17.26 (16.88, 17.76)	19.05 (18.51, 19.65)	36.31 (35.46, 37.36)	16.36 (15.72, 17.10)	18.32 (17.69, 19.15)	34.68 (33.55, 36.17)
Iran (Islamic Republic of)	223.56 (195.93, 253.80)	216.44 (189.63, 245.43)	440.00 (386.26, 498.53)	451.58 (406.71, 502.35)	440.28 (397.56, 489.35)	891.85 (805.70, 991.28)	423.79 (407.25, 436.47)	409.71 (392.70, 422.92)	833.50 (802.48, 856.58)	482.33 (459.96, 501.82)	478.69 (456.26, 501.22)	961.03 (916.58, 1001.91)
Lao People's Democratic Republic	16.75 (14.87, 18.35)	15.56 (13.75, 17.25)	32.31 (28.80, 35.56)	23.55 (21.14, 26.16)	18.90 (16.79, 21.06)	42.45 (38.19, 47.04)	24.78 (23.26, 26.43)	19.80 (18.57, 21.40)	44.59 (42.23, 47.70)	31.27 (29.62, 33.06)	25.90 (24.16, 27.57)	57.17 (53.72, 60.37)

North Macedonia	10.87 (9.47, 12.38)	12.48 (10.91, 14.19)	23.36 (20.64, 26.42)	8.72 (7.65, 9.94)	11.03 (9.68, 12.53)	19.75 (17.49, 22.32)	9.01 (8.68, 9.39)	11.43 (11.03, 11.82)	20.44 (19.78, 21.18)	8.18 (7.83, 8.61)	10.10 (9.64, 10.59)	18.28 (17.54, 19.07)
Republic of Moldova	18.23 (16.03, 20.65)	20.54 (18.02, 23.16)	38.77 (34.25, 43.43)	15.50 (13.51, 17.57)	18.88 (16.56, 21.55)	34.37 (30.34, 38.84)	15.34 (14.66, 15.99)	18.42 (17.64, 19.21)	33.76 (32.37, 35.13)	12.70 (12.08, 13.48)	15.59 (14.83, 16.54)	28.29 (27.03, 29.96)
Democratic People's Republic of Korea	67.96 (58.73, 76.88)	70.59 (61.30, 80.11)	138.55 (120.76, 155.91)	107.29 (95.70, 120.64)	102.65 (91.28, 114.89)	209.94 (188.87, 234.28)	98.49 (94.07, 102.92)	98.65 (93.48, 103.87)	197.14 (188.76, 205.96)	107.78 (101.65, 114.92)	104.03 (97.29, 111.29)	211.82 (199.53, 225.14)
Republic of Korea	40.57 (35.43, 46.81)	45.04 (38.82, 52.27)	85.61 (75.30, 96.83)	75.89 (65.37, 87.18)	77.76 (67.27, 89.73)	153.66 (134.85, 174.18)	72.07 (63.73, 80.22)	78.17 (69.19, 87.18)	150.24 (133.14, 166.87)	91.45 (78.30, 104.08)	93.92 (78.01, 109.06)	185.37 (157.79, 211.81)
Eswatini	5.34 (4.70, 5.99)	4.92 (4.35, 5.52)	10.26 (9.14, 11.40)	7.97 (7.17, 8.90)	6.95 (6.25, 7.69)	14.92 (13.53, 16.41)	7.75 (7.48, 7.99)	6.99 (6.75, 7.19)	14.75 (14.31, 15.13)	9.41 (9.02, 9.81)	8.67 (8.34, 9.00)	18.09 (17.41, 18.72)
Syrian Arab Republic	54.85 (46.92, 62.66)	55.50 (47.81, 63.26)	110.35 (95.20, 125.14)	65.67 (57.95, 73.93)	69.57 (61.52, 78.53)	135.24 (120.29, 151.06)	85.91 (83.88, 88.07)	83.30 (81.07, 85.36)	169.21 (165.39, 173.10)	99.24 (96.66, 101.82)	103.93 (101.43, 106.39)	203.17 (198.63, 207.68)
Taiwan (Province of China)	49.45 (43.64, 55.70)	42.55 (37.65, 48.06)	92.00 (81.91, 103.50)	90.64 (81.06, 101.09)	73.59 (65.31, 82.31)	164.23 (148.18, 182.00)	86.83 (82.43, 89.95)	72.28 (67.92, 75.99)	159.12 (151.17, 164.70)	106.02 (96.41, 112.18)	92.92 (83.19, 100.60)	198.94 (181.61, 210.64)
United Republic of Tanzania	135.45 (121.45, 150.25)	134.57 (120.09, 149.18)	270.01 (242.90, 298.37)	293.74 (262.47, 325.10)	272.70 (245.44, 301.79)	566.44 (511.38, 622.52)	310.40 (297.34, 323.31)	289.96 (277.53, 303.70)	600.36 (576.68, 625.04)	475.56 (450.91, 501.94)	437.47 (410.64, 466.11)	913.04 (866.18, 967.51)
Bahamas	0.79 (0.70, 0.89)	0.72 (0.63, 0.80)	1.51 (1.34, 1.68)	1.72 (1.52, 1.93)	1.65 (1.46, 1.84)	3.36 (3.01, 3.74)	1.77 (1.71, 1.82)	1.70 (1.65, 1.75)	3.47 (3.37, 3.56)	2.14 (2.08, 2.19)	2.15 (2.08, 2.20)	4.28 (4.18, 4.38)
Gambia	5.82 (5.15, 6.52)	5.71 (5.07, 6.34)	11.53 (10.26, 12.76)	14.63 (13.09, 16.21)	13.95 (12.50, 15.50)	28.57 (25.75, 31.44)	14.08 (13.56, 14.55)	13.31 (12.82, 13.77)	27.38 (26.39, 28.24)	19.21 (18.40, 20.08)	18.26 (17.42, 19.06)	37.46 (35.94, 39.08)
United States of America	527.55 (471.65, 588.26)	577.03 (515.51, 646.75)	1104.58 (989.06, 1237.09)	732.51 (680.09, 792.85)	795.69 (739.14, 862.60)	1528.20 (1420.47, 1649.74)	726.62 (690.54, 770.94)	817.27 (773.10, 871.91)	1543.90 (1466.50, 1633.47)	839.33 (790.83, 903.85)	926.32 (868.38, 996.85)	1765.65 (1666.31, 1894.16)
Venezuela (Bolivarian Republic of)	85.41 (75.65, 95.36)	70.75 (62.89, 79.73)	156.16 (139.54, 173.78)	123.84 (109.15, 139.01)	108.92 (96.58, 122.34)	232.77 (208.07, 259.19)	142.41 (134.67, 149.74)	123.02 (114.75, 132.24)	265.43 (250.53, 280.66)	173.40 (163.00, 184.02)	160.16 (149.35, 171.73)	333.56 (314.01, 355.07)

Viet Nam	205.60 (177.71, 233.49)	179.28 (154.62, 204.62)	384.88 (334.31, 434.91)	291.17 (261.67, 321.39)	236.17 (214.67, 261.23)	527.34 (479.53, 578.78)	309.24 (289.61, 330.44)	275.53 (256.55, 298.40)	584.77 (546.68, 627.63)	395.38 (372.59, 428.34)	386.76 (360.18, 425.45)	782.14 (734.30, 851.23)
United States Virgin Islands	0.39 (0.35, 0.44)	0.37 (0.33, 0.42)	0.77 (0.68, 0.86)	0.60 (0.53, 0.67)	0.66 (0.59, 0.73)	1.26 (1.14, 1.39)	0.59 (0.58, 0.60)	0.65 (0.64, 0.66)	1.24 (1.22, 1.26)	0.53 (0.51, 0.55)	0.68 (0.66, 0.69)	1.21 (1.18, 1.24)
Monaco	0.05 (0.05, 0.06)	0.06 (0.05, 0.07)	0.11 (0.10, 0.12)	0.07 (0.06, 0.08)	0.07 (0.06, 0.08)	0.14 (0.12, 0.15)	0.07 (0.07, 0.07)	0.07 (0.07, 0.07)	0.14 (0.13, 0.14)	0.08 (0.07, 0.08)	0.08 (0.08, 0.09)	0.16 (0.15, 0.17)
San Marino	0.04 (0.04, 0.04)	0.04 (0.04, 0.04)	0.08 (0.07, 0.09)	0.06 (0.05, 0.06)	0.06 (0.05, 0.06)	0.11 (0.10, 0.12)	0.06 (0.05, 0.06)	0.06 (0.06, 0.06)	0.11 (0.11, 0.12)	0.07 (0.06, 0.07)	0.07 (0.06, 0.08)	0.13 (0.13, 0.15)
Saint Kitts and Nevis	0.20 (0.18, 0.22)	0.18 (0.16, 0.20)	0.38 (0.35, 0.41)	0.35 (0.31, 0.39)	0.30 (0.27, 0.33)	0.64 (0.59, 0.71)	0.35 (0.34, 0.36)	0.30 (0.29, 0.30)	0.65 (0.63, 0.66)	0.41 (0.40, 0.42)	0.36 (0.35, 0.37)	0.77 (0.75, 0.79)
Cook Islands	0.09 (0.08, 0.11)	0.07 (0.06, 0.08)	0.16 (0.15, 0.18)	0.15 (0.14, 0.17)	0.12 (0.11, 0.14)	0.27 (0.25, 0.31)	0.15 (0.14, 0.15)	0.12 (0.11, 0.12)	0.27 (0.26, 0.27)	0.13 (0.13, 0.14)	0.10 (0.10, 0.11)	0.24 (0.23, 0.24)
Nauru	0.05 (0.04, 0.05)	0.04 (0.03, 0.04)	0.09 (0.08, 0.10)	0.07 (0.06, 0.08)	0.05 (0.05, 0.06)	0.12 (0.11, 0.13)	0.07 (0.06, 0.07)	0.05 (0.05, 0.05)	0.12 (0.11, 0.12)	0.07 (0.06, 0.07)	0.05 (0.05, 0.05)	0.12 (0.11, 0.12)
Niue	0.01 (0.01, 0.01)	0.01 (0.01, 0.01)	0.02 (0.02, 0.03)	0.01 (0.01, 0.02)	0.01 (0.01, 0.01)	0.03 (0.02, 0.03)	0.01 (0.01, 0.01)	0.01 (0.01, 0.01)	0.03 (0.02, 0.03)	0.01 (0.01, 0.02)	0.01 (0.01, 0.01)	0.03 (0.03, 0.03)
Palau	0.08 (0.07, 0.09)	0.06 (0.06, 0.07)	0.14 (0.13, 0.16)	0.18 (0.16, 0.20)	0.12 (0.10, 0.13)	0.29 (0.26, 0.33)	0.17 (0.17, 0.18)	0.11 (0.11, 0.11)	0.29 (0.28, 0.29)	0.16 (0.15, 0.16)	0.10 (0.10, 0.11)	0.26 (0.25, 0.27)
Tokelau	0.01 (0.01, 0.01)	0.01 (0.01, 0.01)	0.02 (0.02, 0.02)	0.01 (0.01, 0.01)	0.01 (0.01, 0.01)	0.02 (0.02, 0.02)	0.01 (0.01, 0.01)	0.01 (0.01, 0.01)	0.02 (0.02, 0.02)	0.01 (0.01, 0.01)	0.01 (0.01, 0.01)	0.02 (0.02, 0.02)
Tuvalu	0.05 (0.04, 0.05)	0.04 (0.04, 0.05)	0.09 (0.08, 0.10)	0.09 (0.08, 0.10)	0.07 (0.06, 0.07)	0.15 (0.14, 0.17)	0.09 (0.09, 0.09)	0.07 (0.07, 0.07)	0.15 (0.15, 0.16)	0.11 (0.10, 0.11)	0.08 (0.08, 0.08)	0.19 (0.18, 0.19)
**Region**												
Global	22871.76 (20762.22, 25091.33)	23177.75 (21107.51, 25383.22)	46049.51 (41921.76, 50436.56)	33150.58 (30444.45, 35992.51)	32666.25 (30112.07, 35390.74)	65816.83 (60548.98, 71426.07)	33883.55 (32584.70, 35109.47)	33379.24 (32156.44, 34647.51)	67262.79 (64818.61, 69744.49)	39771.12 (37768.26, 41615.55)	38959.60 (36517.83, 41198.99)	78730.72 (74774.28, 82849.14)
Andean Latin America	159.59 (146.99, 172.91)	151.75 (137.79, 165.60)	311.34 (285.66, 337.24)	332.43 (302.95, 363.97)	318.16 (290.87, 347.50)	650.59 (596.66, 708.87)	335.84 (325.68, 343.94)	316.15 (305.79, 324.00)	651.98 (632.53, 666.98)	403.67 (383.40, 422.49)	384.12 (365.44, 403.51)	787.79 (750.68, 824.29)
Australasia	32.07 (28.78, 35.63)	29.75 (26.69, 32.88)	61.82 (55.81, 68.47)	53.80 (48.45, 59.26)	47.08 (42.65, 52.09)	100.88 (91.76, 110.81)	54.85 (52.48, 59.39)	47.58 (44.57, 52.82)	102.43 (97.47, 111.78)	68.52 (64.68, 75.10)	61.11 (57.56, 69.33)	129.63 (122.70, 144.54)
Caribbean	153.94 (141.25, 166.00)	136.50 (125.67, 147.90)	290.44 (267.84, 313.53)	269.02 (247.70, 292.72)	251.03 (232.71, 271.24)	520.04 (480.63, 563.86)	257.87 (247.32, 268.27)	240.46 (230.01, 251.07)	498.33 (477.87, 518.95)	285.35 (272.50, 296.26)	276.22 (263.59, 287.78)	561.57 (536.49, 583.01)
Central Asia	254.32 (226.24, 281.18)	264.44 (235.61, 292.52)	518.76 (463.20, 573.42)	399.85 (352.51, 449.72)	423.49 (374.50, 476.69)	823.34 (729.80, 923.64)	400.88 (393.85, 407.78)	423.48 (416.05, 430.61)	824.36 (811.29, 837.20)	468.06 (456.66, 478.19)	497.28 (485.08, 508.80)	965.33 (943.43, 983.19)

Central Europe	573.20 (504.64, 646.35)	650.72 (575.16, 734.94)	1223.92 (1083.10, 1379.11)	397.44 (361.20, 435.90)	488.93 (445.23, 533.68)	886.37 (806.29, 969.94)	409.32 (395.95, 422.84)	508.30 (494.36, 524.35)	917.62 (892.35, 943.14)	363.60 (347.14, 381.75)	442.81 (420.97, 465.25)	806.41 (772.19, 841.48)
Central Latin America	820.22 (742.93, 898.02)	705.37 (641.02, 772.81)	1525.59 (1388.72, 1670.83)	821.01 (738.24, 907.79)	771.78 (697.67, 853.96)	1592.78 (1440.82, 1756.92)	935.48 (896.43, 981.79)	888.84 (844.87, 938.76)	1824.32 (1747.36, 1915.37)	1194.56 (1128.99, 1271.37)	1186.04 (1120.57, 1268.89)	2380.60 (2258.61, 2531.31)
Central Sub-Saharan Africa	257.54 (232.21, 281.87)	241.86 (214.98, 268.26)	499.40 (447.32, 548.96)	782.59 (707.62, 856.79)	651.26 (584.46, 718.87)	1433.85 (1296.65, 1565.49)	774.99 (754.20, 789.22)	646.56 (624.56, 662.31)	1421.56 (1379.94, 1449.66)	1206.27 (1167.15, 1247.49)	1010.02 (969.81, 1053.90)	2216.29 (2145.42, 2289.96)
East Asia	2973.21 (2672.57, 3281.33)	2621.42 (2358.71, 2906.99)	5594.63 (5027.21, 6180.61)	4058.60 (3654.76, 4498.98)	3491.33 (3151.61, 3863.35)	7549.93 (6817.14, 8351.81)	4147.24 (4033.02, 4260.93)	3624.03 (3519.08, 3737.99)	7771.27 (7559.87, 7995.65)	4978.46 (4729.93, 5264.45)	4637.24 (4359.43, 4935.91)	9615.70 (9108.82, 10173.20)
Eastern Europe	986.47 (879.11, 1096.14)	1138.93 (1018.92, 1266.93)	2125.40 (1897.55, 2362.42)	920.25 (823.68, 1029.60)	1180.35 (1057.51, 1318.68)	2100.59 (1891.89, 2352.07)	917.63 (904.35, 933.65)	1176.17 (1153.22, 1198.65)	2093.80 (2062.58, 2127.05)	844.39 (787.47, 893.14)	1080.84 (1009.48, 1140.84)	1925.23 (1812.16, 2029.47)
Eastern Sub-Saharan Africa	1162.29 (1049.12, 1275.92)	1187.30 (1070.28, 1301.15)	2349.59 (2120.29, 2573.57)	2411.99 (2184.19, 2637.94)	2157.49 (1964.52, 2366.31)	4569.49 (4151.38, 5003.54)	2461.14 (2389.48, 2535.61)	2225.95 (2160.99, 2295.84)	4687.10 (4567.73, 4818.73)	4402.76 (4253.31, 4550.47)	4197.42 (4045.95, 4354.01)	8600.17 (8314.63, 8886.56)
High-income Asia Pacific	133.30 (117.82, 152.48)	151.47 (131.76, 174.37)	284.77 (251.07, 325.90)	161.82 (143.35, 183.64)	177.51 (156.51, 200.09)	339.33 (300.74, 383.72)	150.24 (134.87, 174.78)	174.98 (154.17, 212.21)	325.22 (290.83, 381.47)	200.51 (158.25, 244.43)	225.32 (170.97, 281.12)	425.83 (336.25, 524.84)
High-income North America	600.77 (537.08, 671.67)	628.36 (561.69, 704.19)	1229.12 (1103.84, 1375.12)	855.59 (793.24, 924.75)	880.24 (818.32, 956.98)	1735.83 (1609.77, 1881.19)	845.63 (813.47, 874.04)	905.66 (868.20, 938.98)	1751.29 (1688.75, 1806.16)	960.73 (917.18, 1015.19)	1022.11 (968.32, 1084.69)	1982.84 (1894.60, 2088.97)
North Africa and Middle East	1523.07 (1366.05, 1682.61)	1449.15 (1297.77, 1605.62)	2972.22 (2670.24, 3289.51)	3259.10 (2934.43, 3600.17)	3056.99 (2759.53, 3377.26)	6316.09 (5700.69, 6973.06)	3380.64 (3326.56, 3431.82)	3230.34 (3174.28, 3283.50)	6610.99 (6511.73, 6708.66)	4452.60 (4333.02, 4564.59)	4484.69 (4360.59, 4599.23)	8937.29 (8722.02, 9136.88)

Oceania	37.81 (34.38, 41.66)	28.38 (25.63, 31.02)	66.19 (60.30, 72.40)	105.77 (95.85, 116.38)	74.91 (66.99, 82.60)	180.68 (164.74, 197.03)	104.57 (102.43, 106.07)	75.32 (73.94, 76.63)	179.89 (176.92, 182.56)	142.61 (137.71, 146.16)	108.69 (105.24, 112.24)	251.29 (243.44, 257.61)
South Asia	8703.82 (7773.31, 9698.77)	9462.65 (8489.32, 10463.04)	18166.46 (16220.80, 20169.76)	10863.29 (9868.41, 11953.08)	11457.74 (10469.09, 12524.72)	22321.03 (20332.80, 24479.92)	10980.97 (10575.71, 11345.87)	11344.79 (10926.07, 11725.13)	22325.77 (21562.99, 23003.74)	12498.10 (11917.96, 12973.47)	13391.11 (12733.50, 13892.47)	25889.20 (24728.22, 26836.77)
Southeast Asia	1561.84 (1420.97, 1702.24)	1342.63 (1208.40, 1477.10)	2904.47 (2624.47, 3176.51)	2328.16 (2152.41, 2537.08)	1992.42 (1826.42, 2161.68)	4320.58 (3979.43, 4688.03)	2256.84 (2169.67, 2345.16)	1966.48 (1884.58, 2057.33)	4223.32 (4066.65, 4398.33)	3075.58 (2909.18, 3248.86)	2952.20 (2763.12, 3177.45)	6027.77 (5710.28, 6419.40)
Southern Latin America	121.21 (108.17, 135.01)	98.41 (87.48, 109.56)	219.62 (197.90, 243.63)	181.20 (162.84, 201.84)	144.58 (129.84, 160.34)	325.77 (294.15, 360.03)	187.90 (181.46, 195.90)	158.58 (151.26, 168.59)	346.48 (333.91, 363.36)	212.48 (203.82, 222.72)	181.69 (171.70, 195.79)	394.17 (376.57, 417.94)
Southern Sub-Saharan Africa	271.84 (246.71, 297.92)	260.52 (236.85, 284.80)	532.36 (484.66, 582.21)	399.13 (365.37, 435.01)	372.58 (342.35, 405.03)	771.71 (708.41, 838.84)	398.68 (385.11, 412.61)	382.70 (369.38, 396.17)	781.38 (755.57, 808.63)	544.74 (522.59, 564.32)	532.34 (511.33, 553.60)	1077.08 (1036.11, 1114.17)
Tropical Latin America	696.93 (626.94, 764.60)	693.49 (623.26, 766.89)	1390.42 (1249.86, 1530.16)	885.07 (806.59, 967.65)	964.31 (877.49, 1053.27)	1849.39 (1684.86, 2020.59)	952.53 (918.43, 990.56)	1033.83 (995.07, 1084.51)	1986.36 (1919.00, 2061.22)	1052.03 (1001.90, 1113.84)	1161.15 (1106.28, 1227.06)	2213.17 (2115.21, 2336.33)
Western Europe	654.56 (588.86, 721.63)	673.48 (614.90, 739.37)	1328.04 (1206.25, 1458.97)	850.66 (775.62, 929.19)	890.14 (817.74, 967.27)	1740.81 (1596.17, 1895.23)	847.14 (804.97, 879.87)	886.36 (839.81, 921.64)	1733.50 (1651.20, 1797.12)	998.25 (938.83, 1049.47)	1039.45 (980.46, 1100.77)	2037.70 (1924.34, 2149.05)
Western Sub-Saharan Africa	1193.77 (1089.05, 1296.23)	1261.18 (1142.12, 1377.64)	2454.94 (2233.88, 2672.81)	2813.81 (2563.10, 3054.77)	2873.94 (2610.22, 3144.29)	5687.75 (5178.14, 6190.71)	2917.27 (2831.26, 2987.61)	2935.10 (2850.33, 2996.34)	5852.38 (5684.40, 5978.27)	5012.84 (4795.63, 5219.82)	5053.86 (4838.82, 5242.03)	10066.69 (9648.39, 10444.40)
High SDI	1543.19 (1396.53, 1701.33)	1591.62 (1451.79, 1752.70)	3134.81 (2849.97, 3454.55)	2237.35 (2078.22, 2404.23)	2212.49 (2063.05, 2376.78)	4449.84 (4144.44, 4783.76)	2137.48 (2029.16, 2229.51)	2126.46 (2000.46, 2234.20)	4263.94 (4042.26, 4454.18)	2440.79 (2310.29, 2561.26)	2443.51 (2301.26, 2586.81)	4884.31 (4617.86, 5133.75)

High-middle SDI	3720.22 (3378.71, 4074.21)	3760.99 (3411.19, 4127.65)	7481.21 (6780.19, 8187.60)	4746.45 (4345.50, 5189.94)	4779.46 (4384.98, 5214.53)	9525.91 (8732.10, 10419.68)	4723.39 (4559.06, 4854.26)	4866.34 (4751.25, 4981.54)	9589.73 (9348.98, 9805.68)	4988.11 (4717.48, 5360.03)	5182.66 (4913.58, 5543.19)	10170.77 (9664.87, 10857.64)
Middle SDI	6331.55 (5771.93, 6899.22)	5920.21 (5402.18, 6465.65)	12251.76 (11167.96, 13366.83)	9166.00 (8419.27, 9976.12)	8693.52 (8002.18, 9428.43)	17859.52 (16441.53, 19407.10)	9152.16 (8906.18, 9322.32)	8691.44 (8453.43, 8875.60)	17843.60 (17369.84, 18147.05)	10944.30 (10542.46, 11391.14)	10423.14 (10017.20, 10923.27)	21367.44 (20625.56, 22211.66)
Low-middle SDI	7580.37 (6810.59, 8363.86)	7992.88 (7208.50, 8793.48)	15573.24 (14033.98, 17167.69)	9732.32 (8917.81, 10629.25)	10010.85 (9193.79, 10887.94)	19743.17 (18139.34, 21504.83)	9951.01 (9699.86, 10153.63)	10173.59 (9907.37, 10394.37)	20124.59 (19642.11, 20538.95)	12473.71 (11622.36, 13112.51)	12770.83 (11977.00, 13416.96)	25244.54 (23669.85, 26490.66)
Low SDI	3684.65 (3317.67, 4059.91)	3901.69 (3508.34, 4274.77)	7586.34 (6829.08, 8348.40)	7245.26 (6595.13, 7932.90)	6949.62 (6315.38, 7632.35)	14194.88 (12933.46, 15539.51)	7390.77 (7274.76, 7507.87)	7095.69 (6973.40, 7220.15)	14486.46 (14260.28, 14702.17)	12705.59 (12297.70, 13149.62)	12217.04 (11841.33, 12667.07)	24922.63 (24174.38, 25759.99)

Abbreviations: UIs, uncertainty intervals.

Table S4. Incidence rates per 100,000 for diarrheal diseases in 1990, 2019, 2020, and 2040 for males, females, and both genders combined at the national, regional, and global levels

	**Incidence rate (95 % UI) of diarrheal diseases (per 100,000)**											
	**1990**			**2019**			**2020**			**2040**		
	**Male**	**Female**	**Both genders combined**	**Male**	**Female**	**Both genders combined**	**Male**	**Female**	**Both genders combined**	**Male**	**Female**	**Both genders combined**
**Nation**												
Afghanistan	103500.90 (89856.63, 118187.11)	92518.54 (81271.36, 105657.87)	97950.98 (85555.40, 111040.74)	134661.87 (115349.40, 154616.29)	131934.91 (114048.53, 151225.56)	133333.93 (115835.98, 152023.82)	136108.57 (131159.08, 140396.20)	130250.97 (125444.67, 134413.57)	133240.45 (128796.93, 137150.40)	130134.55 (124983.53, 135839.09)	128394.91 (122882.53, 134273.84)	129287.13 (124205.01, 134708.20)

Albania	115164.64 (98829.82, 132827.59)	139731.81 (119034.33, 160731.41)	127087.19 (109282.05, 145794.72)	88508.19 (76942.65, 100613.68)	110602.26 (97427.28, 126722.73)	99537.10 (88243.58, 111970.53)	88119.70 (84720.18, 91597.38)	107987.10 (103095.02, 111954.76)	98040.59 (94269.31, 101519.31)	83406.27 (79552.09, 87392.51)	96202.08 (91109.50, 101186.28)	89878.39 (85607.65, 93898.15)
Algeria	83869.62 (72948.40, 96107.67)	86454.78 (74892.38, 98087.94)	85147.53 (73701.01, 96602.63)	95374.24 (84593.29, 107130.76)	96206.02 (85151.12, 108148.65)	95784.61 (85431.07, 106919.14)	98911.93 (97207.62, 101121.23)	99573.01 (97972.35, 101231.79)	99238.21 (97811.51, 100928.43)	105775.86 (103541.89, 109153.99)	106722.58 (104495.49, 109450.56)	106241.74 (104052.28, 109033.91)
American Samoa	85383.66 (75505.50, 96228.86)	69579.65 (61940.02, 78411.82)	77698.47 (69326.90, 86921.48)	140212.85 (123768.19, 156722.13)	107465.01 (95370.24, 119931.03)	123948.25 (111187.16, 137427.66)	129405.21 (122691.15, 135641.30)	99824.17 (94925.69, 103907.06)	114749.49 (109230.93, 119438.77)	137430.30 (131462.61, 143843.98)	109079.56 (104509.66, 112924.61)	123425.42 (118783.81, 127983.87)
Andorra	32011.78 (28423.76, 35965.15)	28550.17 (25295.92, 32194.72)	30389.49 (27069.52, 33830.06)	32660.21 (29162.96, 36402.56)	28616.99 (25701.15, 31867.92)	30692.23 (27717.01, 34027.40)	33447.64 (32329.83, 35239.94)	30125.46 (29050.82, 31924.89)	31821.22 (30797.22, 33561.38)	45517.04 (43781.89, 47211.38)	40006.93 (38463.82, 41937.17)	42774.51 (41402.12, 44532.95)
Angola	109365.10 (98287.57, 120306.94)	109947.10 (98753.68, 121106.95)	109653.52 (99086.56, 119561.19)	123943.01 (112123.72, 136105.21)	106028.32 (94563.90, 117677.88)	114720.19 (103765.04, 125427.00)	112627.08 (108663.77, 116439.66)	99854.17 (96263.05, 103171.07)	106071.85 (102347.51, 109600.13)	95811.66 (90213.01, 101197.87)	89586.54 (84378.51, 94596.96)	92655.33 (87341.97, 97673.73)
Antigua and Barbuda	76522.47 (68498.32, 84964.14)	69375.35 (62272.68, 76640.57)	72821.39 (65841.48, 79821.82)	104816.99 (93731.20, 117317.79)	96199.03 (86641.49, 106948.85)	100410.94 (90896.41, 111012.47)	104428.11 (101143.47, 107348.42)	95730.66 (92616.64, 97468.84)	99966.91 (96882.67, 102147.99)	116770.65 (107185.08, 126131.62)	110460.20 (101767.49, 118589.26)	113549.53 (104544.43, 121717.20)
Argentina	47853.71 (42524.76, 53693.64)	36959.53 (32738.42, 41381.97)	42290.66 (37972.31, 46923.55)	56029.35 (49548.14, 63326.72)	40554.93 (36258.05, 45393.98)	48097.48 (43112.79, 53729.99)	68592.54 (64653.85, 71823.24)	57065.53 (52908.22, 60307.45)	62688.53 (58751.74, 65848.42)	60444.14 (55347.56, 64028.92)	48758.94 (43432.84, 52665.12)	54486.73 (49435.04, 57964.83)
Armenia	82167.05 (72062.04, 92489.65)	86222.91 (75418.69, 97412.76)	84235.11 (74519.87, 94485.32)	101168.70 (88868.91, 114749.32)	105419.35 (91548.67, 119850.94)	103367.73 (91062.49, 116204.45)	99977.55 (97606.73, 102553.51)	104421.63 (102292.86, 107122.74)	102275.28 (100244.10, 104686.96)	97910.22 (95093.58, 101580.08)	100125.18 (97560.62, 103892.80)	99044.97 (96621.82, 102515.39)

Australia	29475.50 (26303.97, 32819.26)	26435.89 (23610.36, 29456.97)	27947.21 (24963.95, 31067.22)	35541.52 (31691.17, 39540.71)	29176.35 (25963.16, 32633.84)	32318.34 (29148.40, 35778.55)	36279.02 (34263.40, 39168.31)	30107.00 (28407.49, 33276.87)	33157.88 (31556.86, 36047.99)	43849.10 (38654.36, 51904.57)	39068.75 (33351.73, 48446.06)	41436.32 (36152.32, 49966.63)
Austria	35272.17 (31386.73, 39413.73)	35251.65 (31486.97, 39444.47)	35261.51 (31702.00, 39015.93)	37405.56 (33658.71, 41390.59)	37824.31 (34225.44, 41948.86)	37617.95 (34205.35, 41520.05)	36263.04 (34909.30, 37525.02)	37482.76 (35969.56, 39107.53)	36881.78 (35623.10, 38112.93)	47231.41 (41997.99, 52945.64)	46197.42 (40620.81, 52292.30)	46710.29 (41542.84, 52415.43)
Azerbaijan	77143.45 (67690.17, 86572.42)	79521.67 (69478.55, 89140.42)	78361.18 (69156.47, 87387.82)	99129.78 (86119.75, 113139.95)	104299.08 (90730.07, 120507.03)	101713.16 (88830.09, 115527.22)	98827.53 (95469.27, 101814.39)	107238.16 (103879.51, 110984.56)	103041.74 (100091.45, 105672.88)	91051.81 (87713.51, 94411.51)	94077.82 (90147.50, 98093.91)	92570.47 (89394.53, 95907.42)
Bahrain	65379.20 (56376.26, 75042.77)	72378.25 (62954.32, 82723.87)	68323.80 (59649.50, 77543.91)	82349.96 (72036.48, 93860.97)	88561.29 (77779.83, 100099.33)	84698.62 (75200.22, 94829.41)	80701.53 (77158.87, 84492.23)	87960.40 (84835.41, 91160.77)	83471.22 (80346.02, 86508.63)	113931.99 (108979.83, 118300.67)	106410.12 (102312.38, 109239.64)	110944.51 (106526.70, 114509.00)
Bangladesh	140168.30 (122941.75, 158014.27)	166061.13 (146552.50, 187649.48)	152749.86 (134757.75, 171043.83)	125571.83 (111865.55, 139865.54)	134727.11 (120911.49, 150503.87)	130210.39 (117449.29, 144326.02)	121843.87 (114033.76, 128002.30)	129603.18 (118865.07, 138336.55)	125768.23 (116891.54, 132604.22)	119553.28 (111108.49, 127101.27)	124969.24 (113874.79, 134552.29)	122308.07 (113518.41, 130317.35)
Barbados	79807.57 (71447.91, 88361.07)	71357.89 (63865.80, 79211.80)	75418.11 (68285.26, 82683.26)	121738.11 (108641.98, 134989.14)	119236.61 (106822.59, 133110.76)	120442.35 (108959.58, 132633.38)	118288.43 (112483.05, 122480.88)	113761.85 (107284.61, 118162.61)	115945.44 (110248.55, 120245.45)	132551.40 (128033.17, 136437.27)	131775.99 (126300.78, 135888.83)	132153.99 (127398.39, 135788.44)
Belarus	80621.07 (70211.32, 91375.74)	85626.65 (76032.85, 97146.60)	83278.21 (73542.20, 93576.93)	81233.15 (71822.15, 92421.31)	93892.12 (82712.27, 106005.16)	87992.01 (78164.52, 98840.62)	87468.72 (83195.64, 92598.79)	98754.30 (95068.61, 103282.92)	93495.22 (89827.01, 98192.29)	86175.61 (82977.85, 89911.58)	94662.41 (91397.80, 98120.18)	90714.46 (87656.54, 94124.24)
Belgium	43297.26 (38648.37, 48420.22)	43364.13 (38812.12, 47772.51)	43331.37 (38992.17, 47693.71)	54281.89 (48765.51, 60465.68)	55963.66 (50856.53, 61969.53)	55136.13 (50299.64, 60497.86)	54626.22 (52763.64, 56290.28)	56748.82 (54780.10, 58511.80)	55703.82 (54006.65, 57345.82)	58183.49 (55979.07, 60407.80)	58962.36 (56281.69, 61389.39)	58575.69 (56288.94, 60652.73)

Belize	77287.23 (69217.57, 86769.42)	67234.07 (59299.72, 75458.43)	72320.86 (64674.44, 80553.64)	94738.22 (83892.20, 106209.04)	81443.72 (71546.85, 90625.78)	88060.28 (78012.31, 97695.46)	100903.25 (96231.49, 105026.01)	83977.82 (79902.96, 87867.60)	92393.67 (88235.75, 96227.02)	109013.64 (104488.61, 113568.79)	94586.48 (90795.75, 98177.77)	101675.51 (97436.43, 105412.55)
Benin	116165.67 (103061.82, 129891.51)	113750.73 (101305.36, 128314.95)	114926.55 (102618.78, 127848.12)	117547.88 (106008.30, 130240.80)	113050.20 (101514.04, 125578.63)	115261.33 (104473.27, 126230.74)	113632.04 (110605.74, 116659.55)	109034.22 (106448.00, 111505.79)	111296.50 (108775.47, 113811.39)	113190.12 (108431.78, 118168.18)	106864.60 (101416.30, 112064.61)	110007.46 (105088.33, 114642.50)
Bermuda	74500.80 (66693.31, 82500.11)	61970.41 (55239.62, 68996.57)	68058.83 (61540.03, 74960.12)	125634.70 (113239.90, 139715.88)	120218.83 (107228.28, 134506.73)	122833.37 (111478.34, 136033.48)	121045.73 (115223.38, 124556.88)	117378.57 (111712.78, 120630.96)	119123.47 (113618.48, 122122.71)	147321.12 (140848.28, 153308.60)	151002.98 (145261.96, 155923.84)	149287.82 (143579.70, 153982.78)
Bhutan	137716.06 (120165.03, 156727.19)	188005.24 (164342.88, 212184.15)	161474.55 (142007.00, 181515.67)	149489.63 (132442.84, 167796.68)	153635.56 (137247.95, 171797.36)	151476.57 (135443.21, 167764.62)	131357.38 (125801.06, 136030.97)	141161.53 (135560.71, 146766.70)	135986.46 (130709.67, 140826.02)	132275.96 (126321.77, 138041.80)	144219.67 (137823.51, 150659.31)	138034.41 (132215.56, 143384.82)
Bosnia and Herzegovina	124341.88 (107403.44, 143205.64)	136702.61 (118004.43, 156554.09)	130519.79 (113607.45, 149131.76)	86218.84 (75980.47, 97392.30)	98327.26 (86557.48, 111535.36)	92420.62 (82319.39, 104146.57)	84711.92 (81834.02, 87468.79)	97611.24 (94235.72, 100581.03)	91279.99 (88403.05, 93891.30)	80426.24 (77094.38, 83729.56)	93328.64 (89716.93, 97558.82)	86970.48 (83684.15, 90354.77)
Botswana	135944.48 (120791.98, 152988.70)	112248.49 (100214.87, 125092.21)	123562.77 (111278.45, 136893.65)	134103.41 (118592.74, 150451.60)	114511.88 (102297.70, 127281.82)	124151.33 (111072.10, 137523.56)	133240.24 (129580.04, 136528.23)	112039.62 (108817.84, 114951.23)	122494.54 (119688.89, 125117.59)	130150.56 (125394.72, 134562.20)	113433.70 (110154.35, 116477.58)	121776.45 (118039.21, 125035.29)
Brazil	92616.46 (83256.51, 101657.19)	90256.74 (81092.48, 99894.47)	91423.09 (82189.43, 100606.54)	80774.74 (73520.89, 88346.00)	84342.36 (76743.03, 92133.29)	82600.27 (75153.84, 90280.88)	89655.53 (86266.98, 94126.27)	92774.04 (88894.58, 98205.38)	91251.10 (87775.08, 95895.92)	91102.33 (85783.38, 96110.27)	95391.40 (89779.84, 101115.48)	93307.56 (88139.38, 98522.32)
Bulgaria	97896.54 (85636.71, 111513.58)	107514.66 (94637.82, 121722.79)	102772.51 (91005.49, 116145.11)	74487.38 (65581.87, 83665.68)	85458.48 (75130.35, 97093.89)	80132.45 (71056.56, 89947.84)	83514.60 (78496.49, 89637.48)	95436.57 (89910.01, 102805.94)	89659.88 (84772.91, 96002.05)	39698.30 (34266.13, 48766.17)	48927.31 (43120.13, 57437.57)	44474.72 (39066.04, 52734.92)

Burkina Faso	127083.41 (115488.42, 138768.22)	135139.95 (122524.61, 147901.50)	131258.41 (119264.16, 142309.70)	139388.02 (123652.23, 155606.17)	135995.97 (120389.29, 151264.91)	137645.46 (123137.53, 153011.62)	137581.00 (134701.64, 139777.59)	133347.70 (130617.60, 135245.63)	135407.38 (132683.98, 137112.21)	133563.49 (130124.31, 137122.17)	131163.45 (127793.43, 134590.74)	132345.29 (129316.59, 135708.31)
Burundi	127805.51 (114705.46, 141080.65)	128878.81 (114960.23, 142145.21)	128356.74 (115660.65, 140831.12)	143776.85 (129748.46, 156684.02)	125285.58 (112405.52, 137892.82)	134472.04 (121993.17, 146104.01)	140392.80 (137075.29, 143204.57)	121731.52 (118283.89, 124453.55)	131009.08 (127963.32, 133485.25)	133539.01 (129726.95, 137012.13)	116899.07 (113333.77, 120543.83)	125151.63 (121827.70, 128464.09)
Cambodia	87539.82 (77810.59, 96171.16)	79808.98 (70357.47, 88588.04)	83502.96 (74281.07, 91633.75)	69919.61 (62482.58, 77273.92)	64112.58 (57737.79, 70636.61)	66963.71 (60592.79, 73532.00)	71887.22 (68867.48, 75741.21)	65532.65 (62810.43, 69205.76)	68650.42 (65992.35, 72207.19)	80853.80 (75974.69, 86644.11)	77118.15 (72883.05, 82617.97)	78959.93 (74643.33, 84263.22)
Cameroon	124343.14 (113228.58, 135421.28)	122935.28 (110960.88, 134366.55)	123628.34 (112470.00, 134380.78)	133715.55 (121078.62, 147933.18)	125265.29 (112774.96, 138132.02)	129472.06 (117313.32, 142208.12)	125826.00 (121349.58, 129586.88)	116391.49 (112313.82, 119746.43)	121090.43 (117087.32, 124458.75)	114036.79 (107503.60, 120896.77)	104392.63 (98369.82, 111014.99)	109181.60 (102970.80, 115413.78)
Canada	54315.88 (47608.71, 61760.16)	37084.52 (32753.76, 42467.43)	45586.40 (40608.79, 51568.13)	68393.58 (60840.75, 76719.22)	45513.21 (40514.30, 51014.08)	56771.59 (50915.20, 63102.45)	67941.35 (66407.71, 69551.68)	47999.92 (46632.03, 50507.68)	57816.89 (56677.33, 59618.66)	69312.82 (67347.69, 72420.10)	50513.69 (48628.16, 54537.04)	59809.92 (58161.85, 63209.63)
Central African Republic	101351.88 (91796.79, 111124.29)	99114.51 (89555.22, 107989.63)	100214.52 (91226.59, 108831.83)	133324.11 (118473.66, 147872.23)	116770.44 (103751.90, 129694.19)	124875.52 (110980.72, 137319.83)	128665.50 (125835.59, 133198.71)	113005.99 (110029.48, 117079.13)	120767.57 (118238.95, 124725.65)	151929.17 (147220.82, 156689.38)	134308.74 (129525.81, 139026.53)	142854.82 (138700.67, 147409.27)
Chad	142566.41 (128158.40, 156904.62)	143772.37 (128940.56, 157889.63)	143187.35 (129776.08, 156677.67)	167892.22 (152148.05, 185174.65)	162324.17 (146911.96, 178215.17)	165087.02 (149710.94, 181298.84)	161881.81 (158278.38, 164534.14)	156241.23 (152365.59, 158720.72)	159041.45 (155427.00, 161435.50)	142584.02 (137488.70, 148716.36)	138628.15 (133578.05, 143999.93)	140605.55 (135806.24, 145860.41)
Chile	56695.71 (49457.17, 64197.67)	45093.07 (39719.23, 51601.44)	50793.54 (45316.97, 57133.20)	55536.50 (49742.10, 62609.93)	47152.97 (42270.59, 52780.88)	51266.91 (46343.17, 56849.17)	54663.18 (52936.83, 56386.82)	48861.08 (46979.13, 50637.08)	51709.56 (50006.79, 53277.43)	55638.67 (53212.90, 58159.12)	50797.20 (48454.79, 53758.81)	53176.20 (50933.91, 55793.08)

China	46802.23 (42103.15, 51617.09)	43736.59 (39337.88, 48539.25)	45316.92 (40732.70, 50112.30)	53263.67 (47929.07, 59117.34)	47526.22 (42885.43, 52595.07)	50449.99 (45525.89, 55865.26)	53956.09 (52557.82, 55235.32)	48766.88 (47416.73, 50093.74)	51413.22 (50271.24, 52653.14)	68986.72 (65710.23, 72767.27)	65109.09 (61559.70, 69490.51)	67064.99 (63976.84, 70920.78)
Colombia	99616.46 (88692.60, 111837.14)	81937.93 (73200.45, 90756.15)	90673.04 (81331.78, 100196.56)	76180.41 (67558.97, 85445.73)	67683.53 (59918.78, 75648.96)	71836.23 (64310.73, 79383.01)	76913.78 (74673.49, 79186.70)	67627.30 (65381.42, 70076.39)	72189.52 (70235.97, 74362.84)	83719.54 (80145.93, 87428.30)	78671.77 (74757.20, 83007.90)	81159.57 (77781.95, 84611.64)
Comoros	129318.57 (114993.22, 143552.30)	130846.57 (117013.24, 146364.03)	130089.84 (117151.59, 143068.90)	138318.38 (122908.16, 152873.74)	123052.15 (110295.81, 136700.59)	130676.56 (117343.21, 143817.67)	125759.64 (120027.95, 131307.56)	111896.23 (106346.02, 116759.27)	118805.48 (113419.46, 123724.82)	133483.95 (127280.83, 139247.41)	116190.67 (110768.56, 120882.41)	124841.69 (119348.71, 129682.20)
Costa Rica	90341.27 (78987.01, 102644.86)	75429.90 (66340.54, 84591.58)	82894.28 (73333.62, 93081.14)	74939.28 (66277.20, 84076.99)	64060.23 (57050.02, 72064.58)	69329.85 (62135.86, 77093.19)	74417.19 (70931.29, 78478.26)	64383.49 (61434.07, 67263.66)	69253.09 (66271.30, 72267.82)	81170.91 (76442.33, 87157.33)	77572.64 (73562.02, 81617.08)	79308.41 (75619.07, 83818.97)
Croatia	96559.34 (83987.49, 110131.31)	96751.52 (84870.49, 110201.71)	96658.18 (84833.89, 108770.44)	72685.56 (64224.85, 81629.87)	78762.47 (70173.38, 88252.68)	75816.10 (67921.03, 84011.68)	73652.75 (71286.17, 76228.03)	79967.88 (77089.90, 83244.06)	76904.58 (74513.72, 79506.42)	71835.61 (69103.24, 75235.85)	76206.01 (72872.25, 79970.48)	74072.86 (71388.66, 77488.96)
Cuba	83708.63 (76150.92, 91916.02)	68753.79 (62237.14, 75596.39)	76281.81 (70207.77, 83011.49)	124695.98 (113029.51, 139298.38)	111233.15 (101480.29, 122628.65)	117931.92 (108391.63, 129312.75)	121224.48 (117535.02, 124208.75)	110099.94 (107011.88, 113484.18)	115633.75 (112587.15, 118560.42)	147017.37 (142670.33, 150575.23)	141690.01 (137581.79, 145502.63)	144319.27 (140495.27, 147814.75)
Cyprus	45931.74 (40620.87, 51686.88)	42170.78 (37285.89, 47429.11)	44063.70 (39156.85, 49292.54)	42929.27 (38453.42, 47635.18)	37651.79 (34001.00, 41761.54)	40260.19 (36461.58, 44488.30)	46609.92 (42292.82, 52525.07)	41062.82 (37362.69, 47665.82)	43808.06 (39787.96, 49755.30)	49416.97 (43739.39, 56513.77)	48692.60 (43046.52, 55935.03)	49045.56 (43590.24, 55815.80)
Congo	93093.23 (83108.93, 102307.07)	88062.96 (78438.50, 97446.90)	90520.77 (81092.88, 99597.51)	126418.22 (113658.24, 139078.01)	98057.76 (87753.75, 108429.57)	112113.94 (101585.06, 122620.05)	115241.84 (111208.49, 119398.60)	91473.79 (88030.91, 95121.84)	103297.77 (99952.17, 107268.47)	111964.63 (106291.95, 117717.09)	87722.03 (83308.02, 91735.55)	99839.98 (95303.98, 104435.32)

Denmark	43179.23 (38493.20, 48016.29)	43567.26 (39339.99, 48702.68)	43376.08 (39288.13, 47864.76)	52006.62 (46612.85, 57865.77)	55415.49 (49718.11, 61567.55)	53718.60 (48821.13, 59237.64)	51100.46 (49170.06, 53371.34)	53835.22 (51050.79, 56313.15)	52474.34 (50384.42, 54636.07)	56211.61 (53760.62, 58334.95)	58131.21 (55255.41, 60488.62)	57170.40 (54606.18, 59380.77)
Djibouti	112546.67 (100255.22, 125221.89)	108438.04 (95721.98, 120688.25)	110652.14 (98501.93, 122719.92)	118192.65 (105451.05, 132234.22)	97608.00 (87224.79, 108303.26)	108550.75 (97555.74, 120432.76)	122470.39 (117943.01, 127857.13)	103447.09 (99623.53, 108414.23)	113507.47 (109407.02, 118287.34)	129944.04 (122685.88, 137089.07)	109839.79 (103770.83, 116197.52)	120266.70 (113771.71, 126567.83)
Dominica	76456.11 (67733.75, 85151.86)	71623.63 (63636.74, 79900.84)	74029.97 (66040.52, 81604.95)	102923.83 (91788.98, 114599.00)	96797.32 (86862.19, 106779.03)	99924.09 (90010.30, 109827.52)	100814.05 (98307.51, 103008.94)	95026.53 (92775.19, 96608.80)	97973.97 (95906.99, 99710.87)	109303.80 (106074.92, 112263.08)	103614.85 (100802.91, 105676.50)	106466.76 (103493.89, 108801.95)
Dominican Republic	95233.70 (86642.39, 104286.78)	79851.60 (72510.59, 87864.68)	87390.84 (79756.26, 95175.94)	112945.47 (101583.24, 125691.96)	102212.59 (92051.85, 113323.29)	107609.09 (97312.16, 118761.81)	114557.20 (111871.80, 116922.63)	101963.39 (99053.53, 104359.53)	108275.89 (105632.12, 110425.16)	117556.63 (114455.77, 120601.04)	108128.36 (105146.33, 111140.22)	112787.08 (109660.93, 115518.34)
Ecuador	85166.01 (76161.52, 94577.34)	77376.42 (68624.96, 86291.52)	81252.09 (72725.34, 89722.63)	91124.72 (81919.16, 100385.00)	91579.28 (82145.18, 100987.97)	91352.98 (82588.43, 99815.30)	94584.06 (93017.55, 96414.24)	92484.04 (90782.05, 94165.54)	93528.15 (92209.98, 95123.48)	99347.19 (96307.11, 102645.66)	98244.76 (95373.90, 100969.12)	98792.94 (96173.91, 101512.24)
Egypt	90973.52 (82841.86, 100704.51)	88851.07 (80492.68, 97840.19)	89937.36 (81998.56, 99027.35)	97254.18 (86580.90, 108730.36)	98362.86 (87896.17, 109992.80)	97788.07 (87616.12, 108583.55)	95354.65 (93647.75, 97176.66)	97983.60 (96187.02, 99645.37)	96632.53 (94957.33, 98150.19)	96322.92 (93536.23, 100104.28)	101814.64 (99053.52, 105337.02)	99046.95 (96468.40, 102546.52)
El Salvador	125950.02 (111745.96, 141300.93)	102983.85 (90736.96, 116130.26)	114196.39 (101948.03, 127597.56)	91842.87 (81225.17, 102730.28)	83203.65 (74376.83, 92208.33)	87251.35 (78753.40, 96282.07)	89934.84 (83192.82, 95918.05)	81522.76 (75714.41, 87986.34)	85460.33 (79360.23, 91335.93)	87875.29 (81139.58, 94418.58)	90689.36 (84873.74, 96243.55)	89377.93 (83422.44, 95326.95)
Equatorial Guinea	119693.50 (108764.49, 130160.80)	116736.77 (105885.09, 128197.52)	118156.92 (106978.78, 128513.30)	87095.80 (77200.30, 96980.97)	79479.91 (70870.69, 89859.12)	83593.86 (74664.50, 93069.02)	83712.12 (81623.08, 85808.01)	76451.80 (74459.68, 78236.32)	80334.36 (78619.76, 82044.27)	89379.14 (85198.35, 93146.96)	72960.49 (69599.72, 75604.83)	81581.17 (77931.82, 84394.78)

Eritrea	132436.58 (117059.11, 147246.18)	137148.83 (122642.47, 151927.48)	134792.87 (120442.75, 149154.45)	151267.83 (132754.73, 170182.30)	123153.72 (108537.80, 136951.62)	137291.49 (122019.55, 152145.50)	144418.48 (141620.34, 147109.11)	121146.28 (118367.42, 123783.01)	132738.43 (130223.22, 134923.18)	145043.06 (139495.76, 149517.66)	122897.65 (118520.27, 126809.23)	133877.70 (129375.82, 137929.83)
Estonia	89737.40 (78722.39, 101987.08)	90097.01 (78895.53, 101666.86)	89928.85 (79318.61, 101013.02)	89336.24 (78289.28, 101454.34)	114588.69 (101371.89, 129205.31)	102689.15 (91426.34, 115105.77)	87769.92 (80583.27, 91640.08)	108808.98 (99188.78, 113984.31)	98901.00 (90707.66, 103018.65)	87138.59 (81195.73, 93291.41)	106818.44 (98966.12, 114735.89)	97360.06 (90745.15, 104244.49)
Ethiopia	135563.92 (120849.88, 151410.89)	146028.74 (129940.27, 162822.53)	140774.17 (125468.04, 157240.20)	112198.31 (101250.01, 123231.24)	104684.18 (94010.38, 115856.37)	108476.79 (97962.46, 118680.19)	113202.66 (111416.17, 115762.32)	104956.07 (103178.22, 107285.12)	109115.04 (107464.19, 111377.63)	117950.72 (115566.49, 120679.19)	109625.00 (107160.09, 112340.85)	113821.37 (111590.99, 116397.86)
Fiji	100369.19 (88785.20, 112145.31)	77532.42 (68962.86, 87060.08)	89124.32 (79785.08, 98450.41)	146001.85 (130371.33, 163604.96)	109191.50 (98223.89, 121299.78)	127849.98 (116527.93, 140485.39)	141201.80 (136337.00, 145458.90)	107316.87 (103966.29, 111190.08)	124494.55 (120670.17, 128111.09)	145297.45 (140060.39, 150275.17)	113724.41 (109964.60, 117625.15)	129620.02 (125627.33, 133530.02)
Finland	36223.30 (32143.08, 40492.10)	37268.48 (33354.51, 41747.12)	36761.51 (32979.80, 40667.28)	36274.06 (32085.32, 40588.59)	36135.71 (32338.23, 40432.26)	36203.96 (32527.13, 40053.92)	39333.99 (36923.18, 42020.66)	40994.67 (37935.97, 44547.64)	40175.39 (37576.36, 43099.10)	45333.39 (42464.85, 49287.47)	46686.96 (42837.07, 51582.08)	46017.80 (42752.94, 50047.24)
France	35072.22 (31647.42, 38961.76)	33944.03 (30623.72, 37692.37)	34493.77 (31260.79, 37799.54)	36526.33 (32698.16, 40562.29)	35647.53 (31602.79, 39846.95)	36073.61 (32442.05, 39871.18)	38753.54 (37511.64, 40323.90)	38684.37 (37130.40, 40459.66)	38717.99 (37420.06, 40379.49)	44796.44 (42980.79, 46979.05)	44498.35 (42408.08, 46958.12)	44644.20 (42808.98, 46979.03)
Gabon	87130.79 (76179.54, 97069.12)	80536.68 (70726.71, 90123.73)	83803.69 (73813.05, 93216.00)	108955.97 (97516.27, 121273.41)	80208.01 (71403.96, 89546.81)	94093.97 (84914.71, 103717.24)	105631.39 (102666.92, 108460.40)	78011.29 (75386.34, 80612.37)	91366.49 (89089.39, 93674.75)	99040.35 (93889.30, 103884.63)	77244.21 (73522.31, 80788.96)	87709.17 (83767.98, 91477.39)
Germany	37931.44 (33849.63, 42413.17)	38635.27 (34522.73, 42936.28)	38295.37 (34424.49, 42385.08)	52294.09 (46875.06, 58615.81)	55230.08 (49643.44, 61654.11)	53770.91 (49050.12, 59081.57)	53229.70 (51649.77, 54704.56)	56555.24 (54758.27, 58340.74)	54916.48 (53447.86, 56403.29)	60264.93 (58171.09, 62326.40)	62428.06 (60405.59, 64486.60)	61355.07 (59567.49, 63198.78)

Ghana	119923.90 (109830.29, 130305.76)	116660.63 (106393.30, 127631.39)	118278.42 (108230.93, 128013.56)	119793.90 (107716.60, 132715.70)	111338.16 (100264.80, 122573.42)	115443.24 (105218.45, 126222.54)	115056.20 (110700.94, 119205.59)	109460.60 (105058.13, 113592.86)	112184.14 (108072.20, 115995.29)	114459.99 (108723.64, 120109.69)	111218.65 (105691.25, 116680.58)	112800.12 (107267.61, 117912.65)
Greece	37526.68 (33105.94, 41884.93)	34558.52 (30736.66, 38742.89)	36021.81 (32262.54, 40060.70)	36550.66 (32493.36, 41063.21)	35534.10 (31430.98, 39809.10)	36028.68 (32142.75, 40098.72)	37495.10 (35137.76, 40252.81)	36080.65 (33771.60, 38653.19)	36772.04 (34633.63, 39182.67)	42287.74 (39748.13, 45050.93)	40951.60 (38364.05, 43905.93)	41607.54 (39149.57, 44218.52)
Greenland	51787.57 (44855.39, 58956.86)	41977.29 (36806.08, 47637.76)	47287.25 (41533.85, 53467.08)	54612.43 (48413.62, 61730.38)	42373.03 (37552.69, 48196.47)	48818.83 (43376.64, 54492.46)	53375.64 (50564.03, 56186.89)	43499.61 (41267.68, 46525.63)	48679.15 (46349.69, 51688.69)	52882.29 (50057.33, 55819.77)	42583.60 (40042.59, 45281.70)	47805.75 (45334.66, 50364.65)
Grenada	87101.43 (77561.28, 96999.34)	80576.22 (70677.46, 90847.97)	83784.45 (75074.03, 93060.83)	115226.64 (101972.99, 129959.13)	107135.29 (95471.21, 119984.43)	111276.99 (99883.02, 123917.16)	125130.80 (122378.31, 127908.11)	112520.61 (109565.05, 114933.95)	119007.50 (116277.00, 121313.49)	131508.44 (127289.19, 135575.69)	115907.90 (111968.77, 119252.31)	123906.19 (120018.58, 127342.63)
Guam	101190.06 (89442.78, 114371.34)	83152.84 (73640.20, 93950.20)	92766.86 (83086.34, 103441.66)	177166.80 (158395.29, 198746.04)	136252.51 (121516.41, 153079.34)	157342.71 (142109.92, 175661.73)	174283.38 (170449.46, 177620.28)	133660.28 (130543.72, 136303.63)	154560.48 (151222.20, 157291.91)	179784.50 (174868.86, 183621.59)	142469.81 (138200.20, 145590.39)	161414.78 (157585.54, 164479.23)
Guatemala	193678.21 (175719.58, 211429.95)	163042.09 (149494.28, 178631.76)	178226.99 (165395.78, 193184.65)	99458.59 (88692.15, 110285.74)	82170.56 (73086.66, 90929.52)	90574.43 (81374.02, 99587.91)	96970.24 (92824.62, 101359.49)	84308.99 (79801.04, 88334.59)	90459.05 (86232.35, 94528.53)	91197.72 (86602.10, 95805.62)	83456.32 (79309.81, 88200.06)	87212.32 (82942.92, 91523.85)
Guinea	129604.20 (115485.52, 143983.98)	128944.39 (115463.62, 142547.66)	129267.04 (116259.78, 142860.94)	126631.59 (112972.31, 139853.70)	122356.26 (108880.61, 135810.27)	124428.21 (111219.53, 137078.44)	125307.32 (121539.61, 128900.03)	116247.00 (112742.50, 119567.41)	120640.78 (117276.26, 123990.71)	114971.65 (109235.28, 120974.62)	109487.95 (104039.85, 114809.81)	112177.02 (106823.24, 117474.54)
Guinea-Bissau	116700.89 (104347.00, 130205.02)	125360.03 (112148.31, 140029.79)	121165.95 (108830.57, 134367.45)	127089.94 (114036.42, 140755.98)	128180.56 (114757.07, 142912.49)	127650.09 (115161.40, 140389.16)	125001.39 (121809.53, 127812.27)	123634.57 (120937.49, 126349.10)	124301.46 (121756.22, 126907.88)	131610.57 (128370.27, 134616.94)	128396.42 (125176.26, 131229.59)	129974.24 (126992.36, 132469.21)

Guyana	80849.17 (72786.99, 89522.00)	70049.25 (63430.54, 77369.60)	75387.69 (68556.16, 82631.08)	96938.56 (87231.62, 107497.75)	86640.62 (77909.56, 95517.47)	91736.23 (83332.85, 100697.79)	101943.30 (98334.68, 105558.74)	89467.38 (86034.27, 92340.00)	95617.68 (92379.32, 98401.41)	109768.62 (105939.68, 114221.32)	96833.32 (94146.70, 100321.00)	103160.26 (100303.43, 106427.74)
Haiti	113673.63 (101460.03, 125882.13)	103457.25 (92532.17, 115037.49)	108420.59 (97155.83, 119395.53)	119548.87 (108079.96, 131922.41)	106781.28 (97238.87, 116836.05)	112973.06 (103411.92, 123199.90)	119424.63 (115713.90, 123212.25)	101975.76 (98023.89, 105422.93)	110425.66 (107039.84, 113628.62)	116690.49 (112320.53, 121006.60)	100569.99 (96444.97, 104711.71)	108442.45 (104443.18, 112296.91)
Honduras	145235.53 (127165.50, 166193.04)	123688.57 (108724.70, 140362.64)	134403.95 (118243.47, 152163.62)	94838.22 (83305.51, 108148.09)	81647.41 (72326.78, 91665.02)	88069.14 (78309.72, 99003.25)	91367.50 (87192.17, 95183.74)	79365.00 (75710.95, 82554.08)	85239.77 (81705.15, 88578.21)	88071.48 (84037.12, 91991.69)	77868.88 (74651.76, 81586.03)	82929.03 (79420.35, 86250.23)
Hungary	110846.14 (95665.47, 126795.99)	125798.14 (110196.58, 143009.16)	118614.60 (104203.52, 134331.58)	96898.03 (86351.71, 108144.35)	117135.57 (103817.86, 131118.27)	107495.98 (96820.31, 119033.53)	54077.85 (48897.07, 60135.65)	65876.15 (59536.47, 73252.58)	60257.43 (54514.76, 66851.02)	53021.57 (48142.43, 59218.98)	61106.35 (54521.33, 68375.91)	57204.57 (51768.78, 63637.00)
Iceland	43191.35 (38207.85, 48649.73)	41911.12 (37172.63, 47198.27)	42553.67 (37949.28, 47437.83)	44057.12 (39705.38, 49336.44)	42641.32 (38260.48, 47280.42)	43356.39 (39235.14, 47955.31)	43937.13 (42882.67, 46482.29)	43128.31 (41968.15, 44810.19)	43534.96 (42458.51, 45534.30)	48931.10 (46709.15, 50913.03)	50308.52 (47418.04, 52944.61)	49625.24 (47090.60, 51902.84)
India	152187.96 (135684.39, 169883.25)	180649.64 (161808.03, 199540.47)	165835.22 (148308.42, 184293.14)	114141.47 (103872.17, 125317.94)	127787.34 (117026.88, 139641.13)	120789.43 (110237.44, 132208.38)	110578.83 (105268.54, 115519.64)	123069.54 (117332.19, 128033.53)	116658.74 (111298.93, 121207.04)	102327.19 (95274.19, 109468.11)	116748.24 (108840.96, 124069.95)	109352.86 (102274.28, 115933.86)
Indonesia	65163.20 (59357.40, 71434.93)	55797.27 (50214.74, 61446.14)	60470.17 (54792.05, 66378.70)	64961.53 (60000.50, 70893.96)	57648.75 (52859.76, 62770.63)	61338.01 (56366.53, 66782.11)	62891.57 (59777.48, 66498.56)	55351.63 (52413.64, 58311.23)	59149.67 (56588.22, 62184.08)	74340.06 (69694.92, 79862.52)	65541.14 (60926.58, 69903.01)	69931.32 (65888.52, 74839.98)
Iraq	89354.19 (77438.98, 100523.77)	90292.14 (77570.05, 103176.63)	89813.62 (77876.58, 101283.52)	95696.94 (84199.91, 107710.94)	96427.48 (85026.78, 108856.13)	96053.37 (85195.54, 107361.08)	101023.11 (98495.53, 103425.18)	100626.51 (98132.42, 102981.35)	100830.18 (98385.66, 102992.50)	101883.71 (99707.16, 103938.65)	101808.54 (99751.91, 103799.49)	101846.86 (99835.14, 103735.81)

Ireland	33178.72 (28909.29, 37697.51)	30862.21 (27572.60, 34532.32)	32016.54 (28299.86, 35935.07)	32269.26 (28732.79, 36074.50)	32421.67 (29245.07, 36084.41)	32346.28 (29115.48, 35753.52)	32969.87 (31260.52, 35275.01)	33733.27 (32073.65, 36082.73)	33355.22 (31891.12, 35592.70)	39621.38 (37328.27, 42173.44)	41678.03 (39694.29, 44768.67)	40656.08 (38766.50, 43222.60)
Israel	40711.10 (35892.04, 46460.63)	36265.77 (32018.05, 40655.24)	38464.93 (34140.67, 43062.65)	45995.07 (40922.32, 51290.39)	43294.11 (38765.07, 47998.58)	44638.54 (40096.61, 49356.95)	45328.16 (42276.79, 48190.85)	41019.03 (37732.66, 44160.69)	43165.10 (40032.29, 45907.42)	50038.13 (46538.44, 53766.89)	47534.56 (44827.16, 50763.00)	48792.83 (45974.87, 52271.54)
Italy	27069.41 (24028.17, 30531.52)	26166.51 (23371.61, 29302.98)	26604.72 (23668.97, 29898.26)	23882.73 (21417.85, 26250.92)	23806.15 (21650.26, 26026.78)	23843.40 (21586.26, 26144.30)	25696.17 (22676.18, 29020.74)	27794.44 (24800.11, 31877.18)	26773.24 (23944.74, 30219.62)	31322.86 (28165.60, 35471.64)	32856.60 (29323.30, 37839.18)	32103.46 (28787.62, 36358.30)
Jamaica	73673.58 (66632.12, 80814.04)	62683.45 (56301.77, 69290.10)	68078.70 (62001.65, 74543.40)	89523.72 (79990.24, 99547.56)	79904.23 (70954.24, 88305.62)	84680.87 (75873.65, 93046.98)	85378.00 (82471.66, 88058.20)	76932.64 (74282.39, 79333.93)	81109.65 (78575.35, 83530.19)	95447.19 (91827.14, 98953.01)	91296.61 (87483.70, 94600.09)	93320.37 (89850.63, 96321.18)
Japan	14548.75 (12666.83, 16912.89)	16162.14 (13926.52, 18817.62)	15369.01 (13349.98, 17945.21)	12695.08 (11106.61, 14383.09)	14162.84 (12525.02, 16264.53)	13447.61 (11899.14, 15306.07)	14921.26 (12737.26, 17969.46)	16945.85 (14008.89, 20529.19)	15960.12 (13550.73, 19067.74)	27221.90 (22541.62, 32558.88)	31511.02 (25709.76, 37511.85)	29423.55 (24580.62, 34876.03)
Jordan	74274.52 (64356.96, 84313.46)	76041.08 (65963.26, 87200.61)	75116.23 (65439.75, 85300.16)	87000.44 (76741.86, 97693.96)	91411.06 (80426.64, 102338.40)	89055.53 (78941.31, 99385.45)	89195.20 (86469.83, 91188.27)	92522.27 (89585.77, 94467.20)	90757.63 (88053.03, 92540.17)	95217.17 (90043.38, 97472.87)	96822.45 (90516.09, 99026.64)	95986.54 (90353.43, 98013.54)
Kazakhstan	63225.80 (55309.46, 71502.09)	63685.38 (56208.36, 71466.95)	63462.72 (56047.93, 70822.67)	76876.08 (67266.22, 87136.50)	80636.11 (70509.40, 90977.29)	78814.90 (69418.20, 88732.76)	75399.99 (73137.25, 77266.36)	79260.03 (77264.53, 80923.11)	77391.63 (75311.50, 78911.46)	72986.12 (70104.72, 75309.27)	77464.73 (74629.19, 79626.93)	75292.37 (72533.04, 77258.64)
Kenya	107427.13 (96874.01, 118546.52)	103187.94 (92932.03, 114296.04)	105291.53 (94858.35, 116279.38)	110802.16 (100460.63, 121136.13)	92663.25 (84100.10, 101616.54)	101714.96 (92372.45, 111305.23)	106255.14 (103766.86, 108891.95)	92395.52 (89833.04, 95244.49)	99307.41 (97027.24, 101694.83)	97691.03 (91093.00, 104019.08)	89847.84 (83394.16, 96335.69)	93728.89 (87543.74, 99802.18)

Kiribati	118863.14 (107142.58, 131229.26)	100552.06 (90149.17, 111470.07)	109630.88 (99593.72, 120696.20)	155810.57 (139749.00, 173127.53)	124023.29 (110693.94, 138226.46)	139608.44 (126897.04, 153247.38)	145088.03 (139167.61, 150806.61)	117629.23 (112225.10, 123269.32)	131135.77 (125955.24, 136015.88)	133379.77 (125453.46, 140535.63)	114067.09 (107245.21, 120347.77)	123625.73 (116765.23, 129812.39)
Kuwait	61976.18 (53637.16, 71004.91)	71420.09 (61054.20, 83299.47)	66008.44 (57349.11, 75688.65)	80795.01 (71355.19, 91405.99)	79590.68 (69803.30, 90023.47)	80235.88 (71577.85, 89973.58)	81035.84 (79383.28, 82626.66)	80648.54 (79087.59, 81983.71)	80857.91 (79360.01, 82184.05)	99370.71 (95942.86, 101859.50)	95730.05 (93098.28, 98359.26)	97641.82 (94915.65, 99940.03)
Kyrgyzstan	68692.27 (60262.82, 76793.22)	68440.29 (60267.26, 76521.61)	68563.41 (60555.75, 76279.86)	85907.13 (75064.71, 98464.00)	88310.07 (77246.61, 101048.02)	87121.35 (76548.66, 98945.95)	86247.32 (84049.71, 89080.28)	88674.46 (85595.42, 92032.94)	87473.57 (85066.58, 90312.01)	83803.69 (81350.46, 86599.63)	86627.46 (83748.47, 89832.83)	85226.35 (82858.58, 88000.86)
Latvia	106084.43 (92397.33, 121242.20)	107209.08 (93752.04, 121852.12)	106686.38 (94180.76, 120514.85)	100258.94 (87985.49, 114983.53)	102374.74 (90117.59, 115148.43)	101400.13 (89740.41, 114247.40)	98950.24 (94888.77, 102999.93)	108418.32 (104138.84, 112828.30)	104066.61 (100251.71, 107872.27)	78198.90 (68927.03, 85895.67)	94896.29 (86090.69, 102878.43)	87154.42 (78061.87, 94910.68)
Lebanon	84156.40 (72609.99, 95354.39)	81176.92 (70576.46, 92093.88)	82682.05 (72269.13, 93266.97)	110605.64 (98395.46, 123581.43)	109486.62 (98113.63, 121577.51)	110037.29 (98453.04, 122086.43)	105777.83 (102636.02, 109050.77)	104967.32 (101946.56, 107937.95)	105375.86 (102511.99, 108338.75)	107954.02 (103946.75, 112217.16)	106994.35 (102837.50, 111159.83)	107479.40 (103542.76, 111327.90)
Lesotho	136543.66 (122955.49, 152743.93)	109172.73 (97973.35, 121321.21)	122545.15 (111113.03, 135414.92)	145331.83 (129403.10, 164349.42)	116810.56 (104796.10, 129907.09)	130897.39 (117662.10, 144963.33)	134303.76 (128232.33, 139531.92)	107984.69 (102713.04, 113397.54)	120755.44 (115344.83, 125698.90)	128681.94 (121584.09, 134250.36)	105646.53 (99387.90, 110919.28)	116598.45 (110826.78, 121290.65)
Liberia	83909.86 (76270.77, 92106.67)	85014.53 (76851.90, 94332.76)	84470.74 (77403.94, 92619.52)	121982.25 (109689.26, 134435.12)	114486.48 (102581.78, 127398.51)	118261.34 (107670.66, 129832.23)	123014.95 (119478.68, 126832.59)	112458.26 (108598.95, 115613.97)	117768.44 (114500.43, 120786.81)	126840.26 (121345.70, 132035.50)	110967.01 (105736.56, 115369.52)	118974.34 (114062.38, 123517.24)
Libya	80268.10 (70060.51, 92113.59)	85227.61 (72716.83, 98255.06)	82634.59 (71619.03, 94207.54)	101529.56 (89838.91, 113940.71)	103443.29 (91930.20, 117585.37)	102454.86 (91225.26, 114130.09)	106361.20 (102738.20, 111026.99)	108205.71 (104581.35, 112396.84)	107257.64 (103723.00, 111711.67)	111119.19 (106425.76, 115752.92)	112885.19 (108554.12, 117815.06)	111988.96 (107654.38, 116736.85)

Lithuania	98683.95 (85928.46, 112896.31)	98040.67 (85938.60, 111429.26)	98344.75 (86601.47, 111547.09)	94326.09 (83197.95, 107388.56)	110115.77 (97773.85, 124605.02)	102836.25 (92195.49, 115275.05)	97860.53 (93616.39, 102351.32)	110686.41 (105762.48, 115272.39)	104779.29 (100255.47, 109234.39)	90679.98 (80263.46, 96410.29)	104581.84 (94708.58, 110450.39)	98126.71 (87855.01, 103666.30)
Luxembourg	40015.31 (35426.32, 44927.83)	41206.13 (36872.14, 45630.26)	40622.82 (36584.58, 44905.57)	42905.11 (38621.02, 47698.07)	44728.08 (40572.04, 49553.32)	43811.39 (39878.70, 48140.76)	46869.28 (44863.69, 50084.85)	48976.66 (46506.64, 52421.05)	47916.13 (45803.49, 51083.25)	54835.73 (52748.87, 57544.29)	54330.83 (52178.65, 57080.73)	54586.15 (52688.52, 57096.26)
Madagascar	125001.96 (112453.41, 138377.13)	112314.07 (100626.30, 124633.96)	118644.05 (106838.40, 130489.25)	135250.80 (120494.80, 151617.56)	111413.86 (98701.77, 124306.93)	123335.35 (110432.35, 137119.20)	131258.60 (127046.59, 135272.48)	112157.00 (108669.04, 115475.76)	121731.15 (118187.83, 125031.97)	116909.23 (111609.35, 121702.16)	101417.04 (96369.29, 105701.96)	109221.26 (104291.72, 113422.84)
Malawi	128433.05 (115326.76, 141374.36)	127750.83 (115665.55, 140596.29)	128085.05 (116246.78, 140482.84)	131091.48 (116410.80, 145269.87)	114521.88 (102395.77, 126568.63)	122579.74 (110249.92, 134541.40)	128802.33 (124686.09, 132085.51)	112559.11 (108811.20, 115475.45)	120437.92 (116688.29, 123365.30)	117526.84 (112304.28, 122299.86)	104504.53 (99244.73, 108283.42)	110815.95 (105973.88, 114790.46)
Malaysia	52178.39 (45784.11, 59178.20)	47126.09 (40532.84, 53824.77)	49676.85 (43517.77, 56119.54)	73021.52 (65839.81, 81252.79)	61984.57 (55131.21, 69273.91)	67694.94 (61108.39, 75108.44)	71055.59 (68157.13, 72873.29)	60404.89 (58096.99, 62625.94)	65898.42 (63410.46, 67546.19)	77631.71 (72958.90, 81185.93)	66819.30 (62402.87, 70086.54)	72336.91 (67956.79, 75429.38)
Maldives	74555.56 (65779.23, 83044.28)	65790.91 (56091.89, 74336.66)	70281.92 (61300.61, 78240.58)	63222.18 (56231.89, 70677.55)	58262.19 (51274.76, 65548.25)	61248.88 (54944.45, 67696.68)	68663.87 (63166.30, 74791.08)	59918.42 (55400.00, 65327.53)	65029.05 (60341.23, 70550.16)	88380.59 (81591.19, 96724.76)	76645.59 (70506.80, 85242.95)	83256.16 (77199.42, 91347.22)
Mali	134705.43 (122156.48, 146957.36)	140822.36 (127276.50, 155644.49)	137815.28 (125171.66, 150611.63)	141399.52 (127041.84, 156680.00)	129648.43 (115096.35, 144566.83)	135486.46 (121074.99, 149339.13)	139783.63 (136989.58, 143793.27)	128698.39 (125761.31, 132258.56)	134216.19 (131465.45, 137865.67)	137173.10 (132182.76, 143088.23)	127963.47 (123160.82, 133555.62)	132578.71 (128036.19, 137984.93)
Malta	31305.92 (27587.22, 35219.79)	28622.08 (25257.05, 32156.98)	29946.78 (26444.30, 33504.16)	30062.36 (26786.65, 33506.45)	30562.54 (27409.84, 34042.42)	30312.69 (27393.24, 33626.76)	29621.17 (28203.33, 31270.95)	29429.43 (27885.44, 31254.09)	29525.34 (28223.69, 31170.46)	50325.47 (42857.27, 59697.98)	47084.12 (39095.78, 56838.88)	48708.65 (41348.49, 57980.96)

Marshall Islands	103229.65 (90608.71, 115649.16)	91166.18 (81199.20, 100926.36)	97341.45 (86578.18, 107746.86)	138457.64 (123907.86, 156116.34)	106381.08 (94817.56, 118469.94)	122796.81 (110432.95, 135722.76)	135406.27 (132442.60, 138090.18)	105935.59 (103531.55, 108094.61)	120993.85 (118632.03, 123202.92)	135027.37 (128623.40, 140308.28)	107258.07 (102328.44, 111225.59)	121331.13 (115869.65, 125656.98)
Mauritania	130483.83 (116796.72, 144041.23)	130165.19 (116158.88, 143036.22)	130323.02 (117676.94, 143125.89)	159914.68 (144067.77, 175378.25)	143478.07 (129390.69, 158787.91)	151558.99 (137732.38, 166268.39)	150464.59 (145765.13, 154819.80)	137674.82 (133109.69, 141650.64)	143977.34 (139553.04, 147910.53)	136068.39 (129465.05, 143224.56)	127598.55 (121339.53, 133647.71)	131819.54 (125666.61, 138054.62)
Mauritius	60208.51 (53887.79, 66790.56)	49689.79 (43482.40, 55875.32)	54945.59 (49385.10, 60701.88)	81948.91 (74137.27, 90474.63)	66457.32 (59419.73, 73907.31)	74103.47 (67503.23, 81460.64)	81010.71 (77501.99, 84817.07)	70398.90 (66989.86, 74442.55)	75627.28 (72365.10, 79160.84)	105313.69 (100009.38, 110821.71)	96446.91 (91579.76, 101518.32)	100760.08 (96101.13, 105763.86)
Mexico	90879.99 (81881.73, 100262.05)	77202.58 (69743.54, 85056.82)	83936.49 (75774.02, 92195.90)	49075.72 (43708.26, 55010.92)	46833.65 (42129.76, 52037.93)	47930.31 (42912.26, 53469.37)	47080.25 (42187.55, 52008.67)	43063.77 (38432.52, 47838.77)	45029.95 (40729.27, 49441.95)	69300.41 (62401.54, 75620.40)	67691.66 (60935.48, 74517.05)	68480.24 (61645.79, 74851.44)
Mongolia	98956.70 (86088.17, 112913.03)	100912.21 (87724.74, 115141.82)	99935.47 (87459.74, 113617.49)	94439.18 (81573.99, 107168.37)	97682.76 (84640.24, 110447.97)	96082.75 (84124.98, 108255.27)	93817.69 (91375.12, 95840.58)	97116.93 (94766.85, 99277.56)	95488.93 (93254.80, 97437.98)	88681.56 (84836.44, 92418.61)	89694.17 (86324.34, 93075.44)	89203.29 (85775.91, 92525.43)
Montenegro	93104.10 (80666.93, 106887.06)	101171.16 (88591.45, 114702.04)	97153.16 (85116.06, 110369.27)	67674.03 (59218.20, 77243.37)	81300.63 (71424.13, 92472.59)	74565.41 (65868.98, 84134.41)	73229.31 (69136.32, 78471.68)	86400.12 (82621.13, 91038.26)	79884.07 (76285.17, 84373.21)	76673.96 (72240.45, 82071.91)	90159.46 (85615.83, 94970.52)	83465.65 (79564.81, 88040.18)
Morocco	90143.25 (80551.72, 100281.26)	88729.33 (79260.54, 98946.94)	89433.95 (80206.80, 98729.36)	97183.60 (86034.51, 109575.59)	95376.09 (84785.52, 106983.53)	96284.96 (85833.61, 107465.41)	96959.07 (95222.85, 98880.57)	94783.11 (93152.84, 96324.06)	95876.37 (94224.00, 97301.41)	103071.52 (100603.87, 105295.64)	101708.91 (99276.52, 103910.49)	102392.95 (100208.25, 104518.09)
Mozambique	115012.13 (102588.72, 126984.79)	109836.82 (98308.64, 122231.70)	112310.26 (100844.72, 123565.48)	110202.90 (97933.15, 122743.30)	88950.44 (79530.69, 99789.41)	99170.97 (89055.61, 110065.38)	103662.72 (100399.58, 106834.95)	84521.18 (81486.66, 87845.22)	93698.95 (90936.88, 96523.03)	105100.64 (101536.72, 108517.46)	88083.57 (84647.19, 91412.40)	96254.38 (93111.20, 99377.76)

Myanmar	82888.83 (75403.64, 89929.19)	73162.15 (65059.85, 80567.82)	77982.81 (71215.40, 84695.10)	73466.02 (66725.33, 81476.07)	60829.07 (54950.01, 66996.23)	66907.50 (61430.14, 73098.05)	71783.85 (68715.38, 76338.60)	62056.58 (59072.75, 67078.22)	66745.26 (63883.05, 71332.87)	78704.02 (75315.04, 83507.98)	69893.62 (66083.15, 75394.87)	74145.54 (70731.74, 79033.56)
Namibia	144369.96 (129996.27, 159963.24)	120250.11 (107869.74, 133637.04)	132032.40 (119972.32, 145235.93)	143536.99 (126699.23, 161175.33)	110210.70 (99354.74, 122371.66)	126346.49 (113532.43, 139308.80)	139493.44 (136050.95, 142351.04)	113543.26 (110808.27, 116550.44)	126088.85 (123548.46, 128688.83)	130482.76 (126090.29, 134139.52)	114026.09 (110736.00, 117289.60)	121951.46 (118635.09, 124831.20)
Nepal	163146.46 (144163.52, 183758.42)	192603.25 (168922.98, 215797.40)	177837.33 (157912.81, 198249.59)	136006.12 (121920.48, 151245.08)	146668.99 (131600.07, 162923.15)	141586.59 (127992.18, 156321.18)	134130.57 (128944.10, 137845.64)	145347.57 (139897.33, 149845.85)	139977.75 (134910.97, 143716.09)	126069.98 (118554.75, 132820.25)	147551.41 (139019.07, 154565.96)	137240.83 (129714.01, 143554.90)
Netherlands	30395.25 (26988.02, 33878.18)	29267.04 (26277.20, 32653.82)	29824.52 (26721.61, 33009.12)	36516.22 (32874.47, 40744.64)	35653.58 (32106.53, 39887.07)	36081.59 (32679.91, 39879.45)	38814.50 (37308.81, 41145.07)	38525.39 (36903.64, 41461.50)	38668.98 (37182.45, 41148.14)	44810.54 (43431.80, 46334.59)	43546.32 (41942.77, 45001.15)	44179.48 (42711.75, 45595.44)
New Zealand	43592.38 (38799.76, 49206.52)	42446.35 (37726.41, 47601.58)	43012.55 (38642.83, 48183.81)	48935.74 (44424.60, 53929.38)	46688.82 (42719.79, 51075.62)	47781.61 (43834.08, 52009.41)	44374.26 (40936.21, 47432.05)	42272.93 (38934.61, 45735.79)	43296.56 (40018.24, 46382.01)	46285.56 (42545.26, 49853.06)	46441.60 (42527.70, 50499.55)	46365.04 (42749.11, 49995.15)
Nicaragua	121741.21 (107038.55, 139030.69)	105122.83 (91274.58, 119008.38)	113291.52 (99814.91, 127011.67)	81657.53 (70463.94, 93197.20)	71307.07 (62501.94, 81217.11)	76415.31 (67274.21, 86321.62)	90317.52 (87341.25, 93697.78)	79243.91 (76196.91, 82198.89)	84720.85 (81995.57, 87711.91)	100989.72 (95955.20, 106672.10)	90752.23 (86740.02, 94894.28)	95830.62 (91585.92, 100412.24)
Niger	142081.76 (128314.15, 155602.32)	149182.79 (134586.75, 164750.90)	145662.21 (132220.16, 159467.56)	156540.63 (139603.11, 172283.36)	153597.60 (135327.53, 171856.27)	155058.76 (137841.37, 171492.24)	153455.35 (150405.93, 158054.19)	149840.05 (146583.59, 153381.20)	151639.45 (148670.68, 155567.46)	149141.74 (143090.41, 156328.17)	147353.88 (142159.45, 153457.86)	148252.71 (142785.27, 154745.12)
Nigeria	123008.10 (111264.69, 135525.81)	133074.00 (119137.46, 146845.37)	128033.28 (115235.82, 141242.30)	112602.06 (101290.31, 124309.14)	114937.67 (103451.08, 126794.27)	113814.94 (102516.31, 125516.07)	113693.52 (111212.87, 116124.40)	114045.28 (111530.95, 116495.10)	113874.52 (111577.73, 116071.93)	119387.65 (116059.66, 123035.27)	122177.04 (119073.28, 125711.07)	120795.90 (117680.80, 124227.21)

Northern Mariana Islands	90388.14 (79435.64, 101882.49)	65036.29 (57414.13, 73636.40)	78335.57 (69821.11, 86932.04)	188855.07 (168251.19, 214702.40)	133388.09 (119045.14, 151156.67)	162037.16 (145904.42, 179160.78)	179857.72 (173557.85, 184021.15)	130414.12 (126602.61, 133316.77)	155618.82 (151125.68, 158497.64)	190193.97 (184766.93, 195291.32)	153241.40 (148346.49, 156961.78)	171592.17 (167290.69, 175406.56)
Norway	42325.32 (38218.50, 46901.64)	43784.19 (39773.24, 48342.78)	43063.17 (39081.27, 47598.70)	47121.28 (42868.18, 51725.10)	47448.04 (43128.15, 51767.20)	47283.20 (43120.46, 51617.52)	47863.74 (45417.25, 51039.57)	46515.45 (44128.77, 49481.31)	47195.49 (44903.91, 50186.47)	54406.55 (51654.33, 57659.59)	51724.80 (48773.48, 55279.00)	53079.59 (50470.87, 56342.50)
Oman	72079.54 (62714.84, 81442.32)	82685.63 (70785.44, 94583.73)	76498.03 (66321.15, 86343.10)	91108.22 (79212.59, 104120.66)	105072.84 (92448.09, 118883.94)	96071.61 (84484.86, 108683.80)	88890.62 (82523.72, 92047.93)	103743.42 (98067.12, 106958.55)	94109.29 (87935.07, 97038.68)	91430.29 (88041.47, 94679.63)	92627.09 (89608.40, 96194.56)	91887.89 (89015.31, 95118.38)
Pakistan	165133.48 (146040.56, 185167.97)	182150.01 (161221.84, 203462.33)	173234.18 (154159.68, 193509.89)	133501.15 (118160.58, 150106.49)	134937.43 (120555.43, 149993.42)	134201.25 (120355.94, 149606.29)	129423.94 (124084.50, 134562.81)	127665.98 (122189.33, 133094.28)	128568.25 (123734.68, 133826.41)	125245.60 (119531.47, 130634.23)	124450.18 (118838.47, 130000.21)	124858.93 (119673.43, 130141.84)
Palestine	84229.59 (72055.95, 97451.65)	83641.47 (71363.87, 97812.23)	83937.80 (72324.18, 97042.77)	92501.13 (80801.78, 105411.61)	91463.24 (79798.03, 104422.10)	91992.01 (80553.88, 104674.54)	107567.93 (102506.03, 112632.99)	97694.66 (93629.14, 101186.15)	102726.94 (98301.03, 106642.92)	111896.08 (106016.40, 117301.43)	98020.13 (93950.45, 101898.09)	105095.17 (100881.38, 109320.44)
Panama	100979.83 (89060.01, 113752.91)	83816.01 (74589.70, 92717.42)	92507.61 (82728.56, 102824.57)	85091.04 (74913.76, 95318.56)	75816.27 (67847.76, 85236.94)	80487.89 (72082.72, 89610.54)	87274.14 (84343.30, 90802.47)	77173.47 (74468.83, 80467.05)	82244.99 (79674.03, 85512.80)	96631.76 (93081.60, 100900.49)	88421.73 (84834.87, 92092.37)	92525.66 (89098.88, 96125.02)
Papua New Guinea	115986.09 (105120.47, 128743.74)	92936.90 (83454.73, 102585.50)	104906.99 (95158.79, 115143.66)	154226.23 (138264.96, 171450.31)	114739.93 (101735.93, 127819.75)	135171.02 (122259.24, 148441.14)	149015.00 (146293.34, 151276.89)	110677.08 (107820.02, 113046.51)	130432.18 (128096.24, 132439.18)	146551.48 (142890.07, 149608.59)	113464.24 (110234.79, 116669.19)	130254.84 (127439.22, 132988.11)
Paraguay	76593.29 (68514.13, 85413.97)	70305.32 (62634.91, 78067.86)	73469.74 (65720.30, 81192.96)	86921.82 (76787.39, 97123.49)	85425.47 (75990.41, 95927.82)	86182.73 (77053.89, 95749.96)	89203.31 (86624.87, 92073.80)	83659.66 (81214.85, 86801.50)	86463.91 (83931.34, 89194.99)	93770.76 (90934.05, 96459.71)	90383.65 (87888.97, 92797.56)	92086.31 (89652.84, 94325.22)

Peru	80801.74 (74001.38, 87680.01)	76528.70 (70026.61, 83365.39)	78655.02 (72865.46, 84938.77)	108270.76 (98223.70, 119854.65)	101923.48 (92810.31, 111959.62)	105103.78 (96000.48, 114927.41)	104794.42 (100261.18, 108355.02)	98418.03 (94126.34, 102252.42)	101618.06 (97438.10, 105040.54)	105097.77 (100388.14, 109171.24)	98714.64 (93933.69, 102522.38)	101919.57 (97460.10, 105682.26)
Philippines	83021.45 (74522.14, 91427.94)	68484.93 (60987.01, 75465.60)	75804.05 (67681.63, 83475.14)	72907.65 (66410.71, 79914.61)	65439.63 (59534.91, 71791.27)	69226.05 (63318.22, 75691.82)	71722.01 (69244.92, 74131.79)	64291.64 (62018.32, 66818.57)	68058.19 (65860.24, 70354.32)	71780.65 (67682.55, 76137.13)	64387.27 (59892.81, 68587.00)	68119.28 (63948.37, 72090.53)
Poland	74787.57 (64261.28, 85645.26)	80294.94 (70072.89, 91209.35)	77614.18 (67388.55, 88389.86)	50580.77 (46396.83, 55163.95)	61126.78 (56352.55, 66242.02)	56020.77 (51863.82, 60616.49)	62002.07 (58014.13, 67135.07)	71250.73 (67274.42, 77088.90)	66776.76 (63028.56, 72322.76)	57347.87 (53092.93, 62591.73)	67251.32 (63062.77, 72455.22)	62463.68 (58163.41, 67478.94)
Portugal	32311.45 (28699.90, 35760.29)	29385.78 (26247.64, 32507.34)	30798.90 (27604.68, 33983.09)	31913.17 (28729.87, 35150.45)	32817.77 (29484.35, 36430.18)	32387.69 (29289.97, 35365.64)	33980.83 (31417.71, 36900.40)	34151.26 (31517.89, 37595.20)	34070.10 (31690.04, 36974.80)	40601.65 (36800.06, 46106.56)	42965.87 (37610.77, 51106.88)	41837.80 (37297.69, 48201.54)
Puerto Rico	72115.84 (63973.19, 80805.60)	66926.61 (59038.46, 75155.53)	69443.88 (61818.67, 77421.04)	123034.46 (112760.00, 134201.44)	125204.88 (115273.55, 137414.12)	124173.46 (114775.66, 134807.73)	120679.35 (116643.16, 123523.37)	123670.97 (119336.03, 126016.90)	122243.00 (117979.78, 124644.68)	142657.82 (136488.44, 145796.71)	153151.87 (142929.40, 156196.62)	148169.37 (140478.53, 150963.45)
Qatar	65990.83 (57535.67, 75008.28)	85577.68 (72563.48, 98758.45)	72492.23 (63243.92, 82134.47)	57799.74 (49856.64, 66688.59)	69473.08 (60815.69, 79140.74)	60763.17 (53318.37, 69191.14)	59576.27 (57537.35, 61658.24)	71947.00 (69870.75, 73745.99)	62731.30 (60992.89, 64557.31)	93170.71 (88178.99, 97928.12)	84903.00 (81127.46, 88496.80)	90728.97 (86475.58, 94634.18)
Romania	104047.92 (90652.70, 117737.38)	121014.91 (105590.96, 138195.32)	112657.41 (99074.37, 127385.21)	77937.12 (68781.97, 88029.89)	96399.99 (84754.16, 108708.54)	87415.84 (77418.40, 98106.72)	84180.88 (80051.25, 89066.53)	99952.87 (95581.22, 105185.43)	92281.18 (88519.01, 97122.89)	80969.74 (76855.63, 86045.94)	95089.70 (90211.25, 100965.01)	88260.85 (84093.94, 93462.32)
Russian Federation	100723.24 (89325.83, 112089.05)	102550.64 (91716.33, 114416.93)	101695.03 (90817.15, 113072.42)	99028.39 (88004.02, 110830.20)	111762.21 (99924.31, 125340.29)	105833.35 (94957.45, 118737.96)	98984.14 (97527.23, 100360.61)	110355.10 (108069.68, 112211.86)	105064.88 (103381.21, 106542.02)	98656.07 (93647.00, 102477.96)	107394.32 (102151.07, 112268.45)	103322.99 (98648.51, 107292.34)

Rwanda	128888.91 (115390.81, 143507.14)	134942.98 (120279.21, 150501.03)	131993.67 (118137.59, 146151.14)	114896.80 (102631.25, 127384.26)	100134.78 (90412.02, 110697.90)	107313.98 (97035.68, 117657.67)	117874.88 (114854.93, 121237.54)	103120.53 (100019.16, 106732.52)	110291.62 (107589.34, 113576.17)	121510.77 (117289.99, 126336.85)	108311.20 (104166.88, 113593.67)	114792.19 (110907.88, 119682.32)
Saint Lucia	81704.36 (73021.90, 90558.52)	72405.35 (64465.79, 81142.57)	76937.46 (68736.25, 85410.61)	121377.93 (108937.30, 135774.11)	111991.95 (100555.45, 123881.13)	116659.59 (105383.67, 128433.58)	122330.39 (119915.93, 124727.38)	110586.52 (108128.65, 112609.74)	116435.45 (114229.06, 118284.27)	134660.42 (130731.85, 138307.94)	122790.36 (118849.32, 126268.61)	128650.32 (124868.16, 131996.37)
Saint Vincent and the Grenadines	77175.28 (68161.04, 86298.88)	69569.42 (62110.61, 76974.59)	73361.41 (66142.37, 81062.10)	109408.07 (98488.24, 120896.79)	98734.89 (88861.95, 109254.89)	104192.80 (95020.81, 114645.15)	111533.95 (108937.36, 114205.58)	100956.50 (98444.28, 103005.40)	106340.99 (104073.70, 108632.51)	126727.82 (122945.30, 130429.73)	117768.79 (114598.26, 120769.27)	122204.84 (119165.56, 125311.50)
Samoa	82453.40 (73196.87, 92945.35)	69829.90 (62172.61, 77861.40)	76448.79 (68695.40, 85058.12)	141510.51 (125898.86, 159594.88)	111265.15 (99260.46, 124257.02)	126827.95 (114121.43, 140968.58)	140434.07 (137164.46, 143592.20)	113571.03 (110608.08, 116150.60)	127406.00 (124687.84, 129953.19)	137529.90 (133325.37, 140621.62)	111839.07 (108064.63, 115277.70)	125168.98 (121353.32, 128269.25)
Sao Tome and Principe	105977.31 (95108.85, 117530.96)	113018.49 (101383.11, 125201.58)	109529.25 (99023.89, 120257.88)	111512.93 (99877.99, 123912.14)	111414.70 (99709.25, 123874.71)	111463.97 (100933.19, 122611.40)	110205.33 (107845.48, 113317.25)	109420.49 (107104.85, 112090.34)	109812.97 (107990.99, 112424.52)	119102.84 (115884.53, 122160.29)	114870.87 (112409.79, 117763.67)	116978.77 (114445.30, 119761.86)
Saudi Arabia	89117.49 (78732.76, 100491.94)	96888.09 (83651.65, 110951.37)	92544.69 (81428.51, 104252.25)	81151.46 (71769.76, 91466.53)	85701.12 (75171.79, 96155.35)	83045.90 (73920.47, 93118.01)	83197.18 (81912.31, 84733.85)	87769.50 (86497.90, 89289.10)	85125.69 (83930.49, 86535.62)	100486.36 (98107.89, 105720.54)	100304.86 (98082.24, 103792.33)	100406.70 (98293.49, 104629.69)
Senegal	143865.27 (131553.35, 158169.86)	138030.21 (125085.03, 151644.63)	140873.55 (128986.57, 154360.82)	157409.19 (143946.61, 170643.91)	140000.15 (128033.81, 152977.06)	148727.66 (136640.43, 161153.48)	152617.83 (149225.94, 155101.79)	140672.29 (137976.42, 143036.93)	146651.81 (143898.10, 148624.77)	145755.70 (142263.30, 149029.22)	136601.98 (133475.66, 139254.44)	141183.72 (138202.56, 143848.95)
Serbia	94990.35 (82293.79, 107467.17)	95082.53 (82994.86, 107971.88)	95037.33 (83335.67, 106744.35)	75892.76 (67233.30, 85393.85)	76860.34 (68003.23, 87267.46)	76380.46 (68170.13, 85697.46)	78244.77 (75246.68, 83429.26)	82947.72 (79247.39, 89283.08)	80609.44 (77379.89, 86334.96)	76292.20 (72565.48, 83864.45)	84353.43 (79332.16, 93603.51)	80312.37 (76233.74, 88362.76)

Seychelles	54106.31 (47771.63, 60667.09)	48456.94 (42807.14, 54546.76)	51275.73 (45251.75, 57407.37)	69330.90 (62539.19, 76880.94)	59855.88 (53443.44, 66984.08)	64841.75 (58816.36, 71449.30)	93925.08 (87225.88, 100885.19)	86567.98 (80761.88, 92108.17)	90414.95 (84465.65, 96302.78)	106448.21 (96972.29, 117695.46)	95081.56 (86721.12, 103810.81)	100899.71 (92430.54, 110427.91)
Sierra Leone	127494.16 (114509.32, 140359.88)	117963.42 (105697.73, 130519.30)	122619.48 (110769.52, 134372.20)	141856.48 (126108.86, 156386.43)	121861.43 (108395.13, 136285.54)	131761.78 (118186.29, 144930.45)	137503.23 (133563.64, 140983.31)	118462.74 (114843.39, 122070.89)	127888.51 (124689.59, 131299.47)	140992.26 (135949.47, 145989.20)	120326.58 (115482.59, 124309.01)	130611.50 (126120.26, 134749.40)
Singapore	16523.31 (14166.68, 19137.66)	18853.50 (16368.66, 21661.30)	17680.36 (15658.49, 19984.26)	22428.49 (19424.93, 26165.82)	23732.63 (20432.48, 27811.05)	23066.10 (20395.83, 26620.51)	22273.01 (20178.30, 25789.75)	23730.72 (21948.45, 26570.63)	23019.79 (21293.96, 25781.27)	24457.65 (21213.69, 28076.17)	24275.58 (21517.09, 27182.41)	24364.36 (21449.55, 27305.35)
Slovakia	120529.76 (104532.95, 138301.31)	109478.99 (95634.37, 125142.26)	114875.62 (100733.69, 130744.18)	88967.43 (78096.73, 100601.86)	86155.01 (76179.72, 96900.86)	87527.88 (77974.84, 97944.14)	50758.97 (45892.80, 58144.52)	52109.97 (46698.28, 59745.71)	51450.52 (46230.74, 58921.57)	54606.47 (49789.79, 60564.31)	60383.02 (54732.85, 67085.71)	57553.56 (52435.86, 63526.34)
Slovenia	91020.95 (79451.11, 103862.01)	98273.41 (86364.79, 111471.02)	94751.12 (83581.85, 107185.94)	68685.28 (60392.23, 77943.23)	79161.61 (69585.51, 89676.47)	73965.70 (65752.29, 82946.89)	71200.00 (68879.19, 74082.48)	76087.07 (72970.00, 78992.91)	73660.35 (71076.22, 76055.49)	68844.46 (65579.90, 72217.12)	72344.43 (68444.96, 75893.29)	70609.24 (67307.35, 73712.03)
Solomon Islands	144380.76 (128969.92, 160929.23)	118917.09 (106122.30, 132303.53)	132106.73 (119544.38, 145875.32)	174815.90 (160174.01, 189701.43)	146613.80 (130866.54, 163972.22)	160993.72 (146671.93, 175847.01)	168893.20 (164258.72, 171863.52)	140249.23 (136018.69, 143914.05)	154871.23 (150867.00, 157897.75)	165388.51 (159625.24, 170162.70)	137842.06 (132626.80, 142719.37)	151890.78 (146614.52, 156315.95)
Somalia	120760.52 (106030.34, 135156.90)	131451.39 (116414.91, 148498.97)	125934.76 (111329.93, 140443.67)	133748.28 (117299.73, 150205.86)	131091.19 (114943.93, 147613.18)	132446.30 (116400.12, 147624.95)	130013.05 (127027.74, 132921.90)	128715.15 (125990.14, 131506.59)	129380.37 (126749.12, 132048.09)	129443.21 (124887.96, 133994.56)	123901.66 (119969.66, 128115.96)	126721.16 (122619.80, 130847.65)
South Africa	98302.75 (88988.31, 107788.20)	94955.28 (86206.28, 104224.86)	96571.42 (87634.95, 105677.65)	89711.28 (81818.44, 98279.36)	85790.98 (78977.33, 93773.40)	87711.16 (80543.03, 95837.44)	89303.83 (86557.75, 92552.12)	86184.00 (83810.23, 88966.82)	87709.06 (85405.99, 90420.44)	101395.75 (98099.64, 105725.71)	96744.61 (93267.35, 100439.82)	99011.61 (96154.82, 102957.47)

South Sudan	129729.82 (115938.47, 144058.83)	133534.42 (119528.53, 148579.17)	131521.84 (118194.57, 144999.86)	153083.48 (136212.41, 170235.14)	139634.79 (123695.88, 156309.23)	146385.48 (130709.38, 162765.33)	143994.53 (139458.87, 147434.33)	132993.32 (129070.85, 136117.42)	138594.81 (134390.77, 141673.56)	135802.52 (129590.63, 140924.20)	126241.13 (120665.63, 131316.61)	131046.08 (125210.19, 136223.19)
Spain	37077.88 (32963.93, 41590.82)	34739.97 (31160.62, 38753.59)	35885.49 (32334.19, 39795.25)	40439.11 (36191.90, 44639.96)	41893.71 (37820.95, 46574.20)	41181.71 (37565.67, 45307.86)	42710.86 (40143.53, 47104.07)	43618.01 (40615.85, 48318.81)	43173.20 (40504.83, 47976.19)	49523.89 (46436.70, 54079.63)	50043.82 (46402.43, 55591.14)	49788.37 (46534.32, 54639.96)
Sri Lanka	57540.91 (52671.84, 63352.87)	45780.96 (41068.30, 50907.54)	51693.09 (47308.16, 56531.40)	74005.46 (66662.08, 80764.58)	63399.12 (57053.64, 70019.19)	68525.09 (62361.40, 75110.81)	73087.04 (70665.83, 75478.46)	64794.56 (62245.06, 67173.86)	68798.17 (66572.28, 70998.18)	96376.59 (93583.26, 99395.35)	91016.38 (88413.85, 94152.62)	93582.85 (91065.28, 96323.58)
Sudan	115420.09 (104135.57, 128437.48)	110530.79 (99113.69, 123548.50)	112999.67 (101637.36, 125272.24)	135500.79 (120941.06, 151046.99)	127413.17 (112517.86, 143573.28)	131511.86 (117472.11, 146652.18)	132206.58 (126817.62, 136135.18)	124819.91 (119771.17, 128574.12)	128555.88 (123478.96, 132123.22)	123706.40 (115767.47, 131557.53)	116510.44 (109251.55, 123919.83)	120133.72 (112877.27, 127452.26)
Suriname	82226.51 (73486.71, 91035.68)	70184.34 (63114.95, 77264.92)	76243.85 (68892.55, 83430.08)	106955.85 (95978.58, 118570.13)	98706.98 (89267.48, 108430.58)	102786.72 (93771.19, 112629.08)	106630.02 (104007.73, 109203.41)	98638.43 (95896.79, 101041.67)	102598.19 (100108.36, 105059.14)	113982.73 (110796.99, 118062.97)	105321.62 (102246.29, 108752.01)	109565.36 (106936.41, 112823.67)
Sweden	41181.94 (36641.74, 45786.69)	42228.25 (37846.32, 47227.08)	41711.84 (37450.82, 46355.89)	55252.04 (49521.88, 61481.38)	54743.18 (49225.85, 60926.16)	54998.86 (49777.72, 60706.64)	54440.16 (52682.29, 55871.33)	54269.45 (52452.25, 55765.68)	54355.21 (52709.68, 55669.66)	58265.99 (55771.46, 60398.03)	56408.70 (53823.73, 58507.09)	57347.05 (54806.25, 59350.47)
Switzerland	34291.03 (30323.02, 37965.91)	35262.43 (31489.03, 39112.99)	34783.83 (31255.92, 38226.66)	38602.03 (34740.16, 42965.13)	41854.69 (37633.31, 46479.70)	40234.77 (36647.35, 44134.28)	38210.66 (36968.53, 39611.32)	40471.76 (38765.11, 42038.13)	39344.96 (38070.22, 40632.52)	44250.46 (42021.46, 46696.64)	44789.73 (42024.04, 47450.52)	44519.10 (42102.02, 46847.06)
Tajikistan	97407.60 (85696.62, 109603.72)	96653.16 (84655.69, 108533.97)	97028.41 (85522.31, 108938.33)	103777.41 (90873.40, 117057.74)	108472.28 (94124.89, 124157.93)	106098.79 (93921.96, 119453.98)	101773.02 (98554.78, 104546.47)	106161.81 (102748.23, 108942.39)	103946.93 (100801.14, 106484.08)	98777.30 (94902.13, 102153.78)	103132.85 (99094.28, 106702.72)	100939.18 (97043.66, 104012.85)

Thailand	54476.91 (48791.17, 60468.72)	45468.03 (40560.82, 50643.80)	49935.38 (44999.32, 55278.86)	83510.99 (76053.83, 92320.93)	63927.09 (57244.19, 71124.46)	73483.18 (67473.04, 80368.36)	78694.92 (72932.98, 83957.88)	60066.33 (54830.53, 65427.00)	69152.03 (63961.91, 74106.19)	110944.65 (105226.83, 116620.81)	92946.47 (86677.94, 99479.92)	101619.09 (96112.38, 107428.27)
Timor-Leste	88923.67 (80430.98, 96629.43)	84344.12 (76841.82, 91360.20)	86706.13 (79160.17, 93410.30)	84956.14 (76230.20, 93588.93)	68537.14 (61023.84, 75891.34)	76843.86 (69309.34, 84161.02)	93881.60 (88249.49, 99484.56)	78916.96 (73384.73, 84567.48)	86490.26 (81353.11, 91602.63)	87031.05 (80632.17, 93248.33)	76685.73 (70280.54, 82331.16)	81897.37 (75906.74, 87618.40)
Togo	128741.20 (115742.29, 141892.40)	128716.79 (115859.75, 141046.81)	128728.69 (116648.69, 140590.28)	143580.74 (127121.60, 159800.72)	138461.73 (123574.34, 153104.68)	140967.60 (126382.04, 155480.63)	138793.90 (135818.27, 141355.33)	133831.31 (131130.10, 136193.79)	136264.49 (133772.32, 138616.90)	142360.58 (139168.50, 145602.37)	134759.02 (131874.13, 137654.81)	138485.16 (135607.56, 141406.55)
Tonga	86557.59 (76036.37, 98520.26)	74317.82 (65226.14, 83544.84)	80482.63 (71368.67, 90076.14)	140848.75 (125402.55, 158388.35)	110647.64 (98660.50, 123974.97)	125673.06 (112835.66, 139873.11)	136958.44 (134346.18, 139209.88)	108750.65 (106798.02, 110627.02)	122833.36 (120940.70, 124441.54)	138543.81 (134927.35, 142014.33)	113877.71 (111134.23, 116533.44)	126187.61 (123240.88, 128937.80)
Trinidad and Tobago	56976.27 (50979.42, 63278.36)	50207.65 (44969.00, 55553.62)	53594.55 (48148.23, 58844.22)	87001.03 (77603.96, 97497.45)	79527.92 (70915.82, 87957.50)	83277.67 (75131.97, 92050.13)	83878.77 (80384.72, 87152.67)	77309.15 (74280.02, 80295.24)	80596.48 (77468.44, 83533.09)	97681.44 (93585.05, 101379.41)	96409.89 (92450.95, 99622.76)	97034.69 (93249.72, 100178.12)
Tunisia	79321.50 (69788.90, 89323.95)	79016.44 (69603.12, 89184.22)	79171.10 (69903.58, 88900.25)	96598.52 (86915.52, 107158.24)	96053.85 (86312.33, 106253.69)	96325.10 (87181.01, 106257.21)	94891.40 (91369.10, 96854.79)	93752.35 (89316.04, 95775.72)	94319.45 (90479.47, 96029.43)	102478.55 (93191.19, 105014.37)	106679.21 (94162.49, 109172.24)	104597.91 (93724.81, 106920.20)
Turkey	74099.06 (65222.91, 83237.38)	71473.37 (62922.79, 80472.90)	72804.78 (64309.54, 81385.72)	102069.30 (90997.04, 115429.30)	102175.82 (91801.81, 114035.97)	102122.00 (91907.60, 113346.13)	97424.14 (93145.67, 102011.76)	100810.32 (95915.45, 105243.54)	99119.41 (94636.54, 103118.12)	98242.99 (92438.24, 103139.84)	105546.62 (99082.32, 111303.46)	101940.30 (96139.08, 107269.44)
Turkmenistan	79492.33 (70868.21, 88839.38)	75860.63 (67159.95, 84690.24)	77653.35 (69657.71, 86504.48)	75192.86 (65307.92, 85604.42)	79132.10 (69131.07, 89510.36)	77120.34 (67689.59, 87022.95)	75408.34 (73331.30, 78124.94)	78330.50 (76001.06, 80980.94)	76841.72 (75006.55, 79315.08)	78226.45 (73222.49, 83057.72)	83910.79 (78078.97, 89967.81)	81043.12 (75725.50, 86230.88)

Uganda	122138.91 (110359.07, 133731.88)	118556.26 (106731.52, 130122.47)	120318.41 (109211.93, 131479.36)	116330.13 (103529.13, 129453.41)	100780.34 (89898.81, 111524.80)	108428.07 (97431.08, 119849.87)	111130.93 (107162.31, 114604.78)	96646.24 (93057.41, 100388.12)	103774.40 (100348.91, 106960.98)	107777.96 (103657.64, 111318.56)	95841.08 (92193.13, 99502.24)	101717.22 (98031.91, 105056.74)
Ukraine	73646.37 (64905.12, 83234.10)	72640.14 (64052.97, 81854.80)	73106.06 (65250.01, 82150.35)	81707.72 (71926.74, 93338.24)	85231.22 (74326.37, 95781.74)	83608.00 (74362.93, 94286.16)	79402.99 (76279.75, 82262.11)	84108.77 (81483.67, 86788.70)	81946.99 (79223.07, 84502.83)	80115.29 (77113.82, 83034.19)	84853.42 (81462.19, 87970.69)	82707.74 (79618.76, 85613.84)
United Arab Emirates	61737.37 (54544.07, 69492.49)	76838.51 (66499.57, 87831.79)	67066.96 (59159.78, 75534.44)	71733.47 (62150.59, 82970.12)	82497.57 (71914.54, 94450.26)	74680.35 (65454.46, 84747.12)	71324.64 (67412.90, 74230.36)	79920.91 (76319.91, 82224.50)	73675.53 (69782.82, 76184.89)	84875.30 (73303.59, 99127.49)	56091.96 (47819.18, 66602.95)	75800.04 (65802.63, 88179.19)
United Kingdom	33891.53 (30375.48, 37541.57)	32477.59 (29346.39, 35757.44)	33163.59 (29955.52, 36582.27)	36202.72 (32711.30, 39764.34)	36275.33 (33032.08, 39547.74)	36239.40 (32910.22, 39620.23)	36354.95 (35521.65, 37184.24)	36566.37 (35621.57, 37555.24)	36461.71 (35657.92, 37241.60)	40543.89 (39437.34, 41933.90)	40946.42 (39790.42, 42389.71)	40745.89 (39755.68, 42053.80)
Uruguay	43557.11 (38700.23, 48492.84)	33708.94 (30072.94, 37191.49)	38497.78 (34557.24, 42373.38)	50857.68 (44886.49, 56639.86)	39629.49 (35453.37, 44072.00)	45014.75 (40593.89, 49682.17)	52318.66 (49674.41, 55566.21)	44438.49 (41795.14, 47834.94)	48224.68 (45700.24, 51501.39)	53974.08 (48786.67, 61121.25)	50358.12 (43096.03, 60359.79)	52110.13 (46164.19, 60548.26)
Uzbekistan	73992.50 (64781.03, 82955.05)	72565.54 (64295.70, 81169.04)	73271.28 (64994.87, 81709.04)	80731.79 (69734.47, 92351.71)	84071.00 (73142.96, 95573.94)	82404.40 (72097.30, 93655.72)	81060.70 (77277.98, 84563.07)	83040.90 (78633.60, 87487.70)	82055.20 (78076.22, 85859.73)	73566.54 (70662.27, 76481.86)	74858.72 (71674.35, 78037.30)	74216.65 (71294.88, 77155.84)
Vanuatu	115049.66 (102390.05, 128040.28)	97527.14 (87408.84, 108493.51)	106544.48 (95784.71, 117653.27)	154292.30 (138815.41, 170874.01)	121715.13 (108662.05, 135362.03)	138227.69 (125207.86, 152956.77)	147911.49 (143582.93, 151092.27)	117419.77 (114206.60, 119913.74)	132847.90 (129141.94, 135228.88)	144021.89 (139398.49, 149229.94)	116425.47 (112905.15, 120275.04)	130266.36 (126439.10, 134579.04)
Yemen	125304.19 (111274.42, 141702.82)	130121.68 (115867.76, 148301.64)	127667.44 (113974.28, 143672.11)	132294.09 (114537.46, 150870.20)	136109.89 (117845.77, 155626.80)	134180.54 (117429.25, 152384.33)	137792.72 (134581.02, 140779.48)	138743.66 (136005.91, 141384.16)	138262.54 (135721.59, 140592.48)	123761.19 (118179.67, 129444.31)	125631.16 (120065.84, 131058.66)	124684.75 (119761.30, 129744.37)

Zambia	132775.49 (117647.75, 147432.24)	133379.23 (118624.11, 147833.65)	133082.79 (118902.32, 146633.72)	121642.67 (108651.87, 135817.15)	109844.02 (97705.02, 122442.06)	115677.32 (104160.32, 128056.36)	115989.40 (112212.14, 119403.91)	105597.76 (102028.03, 109087.58)	110732.95 (107487.95, 113860.36)	103065.33 (98536.29, 107211.38)	95454.18 (90804.22, 99613.61)	99191.98 (94855.40, 103131.19)
Zimbabwe	121061.47 (107790.85, 134239.03)	91574.09 (81323.02, 101880.18)	106007.39 (95224.26, 117267.58)	138408.55 (123618.97, 154062.28)	105960.12 (94825.98, 118268.95)	121539.57 (109677.53, 134626.60)	134788.56 (130811.88, 138374.80)	104815.64 (101420.86, 108188.52)	119258.20 (115998.66, 122076.20)	131266.57 (127857.57, 134673.55)	105332.65 (102357.30, 108227.05)	117915.45 (115190.16, 120572.98)
Bolivia (Plurinational State of)	93628.36 (83245.36, 104958.98)	90041.61 (79235.98, 101601.91)	91815.25 (82125.63, 102073.66)	113460.93 (99925.54, 126546.77)	107332.24 (96693.48, 118866.90)	110400.01 (98872.35, 122247.22)	113554.96 (109602.18, 117651.47)	107972.76 (104445.39, 111351.07)	110767.88 (107253.67, 114140.04)	106708.85 (102230.79, 111619.69)	103716.10 (99392.58, 107729.91)	105218.60 (101282.74, 109191.16)
Brunei Darussalam	12655.68 (10953.83, 14446.41)	12938.69 (11399.80, 14711.41)	12788.96 (11310.06, 14475.30)	16462.01 (14225.10, 18946.65)	18161.59 (15688.02, 21064.80)	17271.11 (15152.70, 19533.66)	16579.45 (14200.05, 19416.67)	18044.59 (15972.96, 20593.47)	17280.21 (15193.54, 19771.23)	21970.08 (19535.61, 25740.72)	22009.84 (19900.88, 25656.96)	21989.28 (19912.37, 25699.41)
Cabo Verde	115463.33 (103367.29, 127732.45)	112128.83 (101083.34, 122345.69)	113709.80 (102901.96, 123891.68)	114708.45 (102525.96, 128510.20)	109526.85 (98717.79, 121642.09)	112136.44 (101582.98, 123925.67)	108388.03 (102323.03, 114607.99)	102924.98 (97110.97, 107896.03)	105670.12 (99824.37, 111305.95)	129195.52 (123321.09, 134681.21)	114290.78 (109451.53, 118692.98)	121740.79 (117094.98, 126489.03)
Democratic Republic of the Congo	88819.80 (78349.09, 99002.08)	78830.99 (68499.98, 89313.46)	83757.91 (73820.58, 93332.17)	118012.05 (105986.09, 130836.05)	95157.49 (84665.03, 106435.83)	106597.17 (95717.37, 117777.22)	118252.92 (116185.46, 120306.51)	96054.61 (94209.91, 98035.37)	107147.72 (105541.83, 108878.47)	122809.40 (117943.69, 128024.06)	103081.01 (98093.82, 109320.94)	112915.36 (108517.22, 118570.71)
Cote d'Ivoire	111727.58 (100005.48, 123688.49)	114743.38 (102126.72, 127569.47)	113201.82 (101668.49, 125051.92)	127724.60 (115292.74, 140461.51)	126254.86 (112809.81, 139706.09)	127013.58 (115498.34, 138813.06)	115765.90 (108908.04, 121397.61)	113206.15 (107008.37, 118790.72)	114522.10 (108143.63, 120110.91)	112422.34 (105622.40, 119024.27)	105546.09 (98745.71, 112637.76)	109020.01 (102279.19, 115711.20)
Czechia	97994.62 (84982.65, 111389.87)	105547.87 (92339.82, 120360.93)	101883.83 (89219.07, 115124.83)	86521.11 (76820.82, 96448.00)	100118.21 (89834.29, 112061.68)	93425.12 (84162.50, 103379.60)	77154.16 (71158.64, 82628.19)	88956.70 (82974.22, 94815.15)	83147.74 (76895.78, 88645.45)	75573.30 (69797.64, 80850.40)	85575.51 (78714.51, 91577.39)	80630.34 (74439.35, 86040.38)

Micronesia (Federated States of)	97812.38 (85791.76, 110137.17)	86473.08 (76570.80, 96562.82)	92297.46 (82403.68, 102573.78)	134810.74 (118598.95, 152355.08)	106023.55 (94272.41, 118363.92)	120681.53 (108778.38, 134169.36)	132305.91 (129278.83, 135227.89)	104753.24 (102504.59, 106915.67)	118779.36 (116409.92, 120787.03)	137010.59 (132081.65, 141174.70)	110156.97 (106268.78, 113820.55)	123737.71 (119746.05, 127189.65)
Georgia	76367.22 (66926.10, 85726.10)	76623.59 (67030.62, 86069.12)	76501.87 (67790.34, 85369.60)	97306.14 (85127.98, 111690.59)	97563.26 (86045.01, 110372.95)	97440.12 (86495.59, 109739.54)	98382.82 (96239.25, 101231.60)	99886.06 (97029.97, 103041.60)	99165.92 (96838.65, 102029.58)	98934.94 (95077.73, 103447.79)	101415.54 (97891.44, 105991.44)	100230.22 (96973.86, 104532.03)
Iran (Islamic Republic of)	74713.35 (65480.34, 84820.04)	75630.05 (66263.91, 85762.44)	75161.48 (65982.43, 85159.68)	105465.51 (94987.57, 117323.33)	106140.84 (95843.33, 117971.62)	105797.82 (95578.13, 117591.93)	98816.68 (94958.84, 101772.00)	98054.05 (93982.38, 101216.68)	98440.33 (94776.14, 101165.49)	101923.95 (97196.49, 106042.10)	102274.36 (97480.76, 107087.05)	102098.19 (97376.79, 106441.95)
Lao People's Democratic Republic	82184.45 (72982.75, 90035.82)	73610.03 (65039.68, 81602.88)	77818.97 (69367.74, 85660.42)	65467.91 (58757.75, 72717.80)	53068.87 (47162.72, 59152.36)	59300.45 (53346.33, 65707.53)	67704.12 (63549.28, 72208.72)	54461.98 (51066.13, 58858.54)	61105.19 (57881.52, 65378.79)	70971.47 (67221.48, 75043.16)	58472.45 (54539.25, 62231.83)	64705.13 (60800.75, 68329.18)
North Macedonia	107059.53 (93268.01, 121932.40)	124874.14 (109126.89, 141973.35)	115896.31 (102402.34, 131066.87)	79756.73 (69948.27, 90969.55)	104091.49 (91356.93, 118226.36)	91734.00 (81232.13, 103679.02)	82214.78 (79196.59, 85685.19)	105005.75 (101322.93, 108593.09)	93568.64 (90519.56, 96934.56)	78926.43 (75547.16, 83011.29)	93778.02 (89592.34, 98399.64)	86490.94 (83001.53, 90239.61)
Republic of Moldova	85936.51 (75586.25, 97346.64)	88336.11 (77513.56, 99594.17)	87191.33 (77033.51, 97668.24)	88086.20 (76820.35, 99869.14)	97860.53 (85826.04, 111700.44)	93198.53 (82256.80, 105317.52)	87313.46 (83457.12, 91056.38)	95140.77 (91097.04, 99209.40)	91417.67 (87664.81, 95120.24)	82402.30 (78382.63, 87468.24)	89218.22 (84836.72, 94650.24)	86023.89 (82198.17, 91102.18)
Democratic People's Republic of Korea	68234.13 (58970.98, 77197.38)	63623.33 (55247.87, 72199.56)	65804.31 (57355.69, 74047.51)	82209.58 (73325.25, 92438.85)	77873.07 (69248.64, 87157.27)	80030.54 (71996.82, 89307.07)	76959.12 (73506.33, 80423.73)	75689.34 (71725.42, 79693.92)	76318.44 (73074.99, 79734.00)	86233.58 (81326.62, 91940.10)	81456.01 (76173.74, 87139.77)	83819.03 (78955.86, 89089.07)
Republic of Korea	18219.23 (15910.17, 21019.48)	20407.23 (17593.03, 23684.05)	19308.27 (16982.96, 21839.38)	28127.82 (24226.95, 32310.10)	29436.98 (25467.03, 33968.41)	28775.47 (25254.42, 32618.90)	26917.57 (23803.14, 29961.98)	29589.78 (26191.69, 33001.58)	28244.74 (25031.23, 31370.67)	34252.17 (29327.39, 38982.63)	35307.23 (29327.36, 40998.90)	34778.74 (29603.73, 39739.08)

Eswatini	139457.26 (122683.28, 156468.93)	116113.62 (102617.19, 130268.95)	127194.68 (113319.05, 141272.13)	142919.93 (128549.69, 159610.63)	118905.79 (106957.39, 131697.32)	130635.03 (118432.09, 143692.94)	135091.93 (130289.94, 139159.15)	115061.19 (111120.96, 118345.55)	124788.63 (121101.98, 128005.21)	129724.88 (124304.35, 135206.09)	109714.37 (105513.98, 113811.37)	119290.77 (114851.50, 123457.50)
Syrian Arab Republic	83154.01 (71139.58, 94993.61)	88115.81 (75903.09, 100425.04)	85577.75 (73829.47, 97043.63)	93041.10 (82108.46, 104740.35)	93596.85 (82768.44, 105640.81)	93326.17 (83005.84, 104240.92)	93129.67 (90921.63, 95464.51)	93115.54 (90632.58, 95424.17)	93122.71 (91023.40, 95266.05)	100392.07 (97783.59, 103003.75)	102454.06 (99989.89, 104876.60)	101436.38 (99170.22, 103687.07)
Taiwan (Province of China)	46906.11 (41398.21, 52838.64)	43167.45 (38195.04, 48750.47)	45099.43 (40151.29, 50737.00)	77377.73 (69203.54, 86303.00)	61808.43 (54855.39, 69127.22)	69529.66 (62735.80, 77054.08)	73914.24 (70161.22, 76566.88)	60451.67 (56800.66, 63553.36)	67123.61 (63771.65, 69477.17)	95268.19 (86635.83, 100800.79)	78998.05 (70729.34, 85526.68)	86907.94 (79336.09, 92019.26)
United Republic of Tanzania	107518.04 (96405.90, 119266.11)	101148.51 (90267.71, 112129.76)	104246.43 (93780.34, 115195.85)	106478.79 (95142.85, 117847.31)	93553.92 (84199.74, 103530.92)	99838.36 (90132.85, 109722.48)	107678.41 (103145.62, 112157.10)	95672.50 (91571.76, 100206.69)	101525.14 (97520.63, 105697.81)	96582.32 (91575.23, 101939.80)	85834.73 (80569.95, 91453.67)	91115.88 (86439.78, 96551.69)
Bahamas	63189.97 (55647.58, 70897.36)	54661.80 (48396.79, 61338.78)	58839.58 (52443.32, 65425.57)	94133.62 (83494.49, 106131.74)	84561.81 (75075.07, 94733.85)	89189.48 (79836.03, 99296.44)	95070.59 (92104.53, 97752.52)	85787.02 (82986.00, 88328.93)	90280.13 (87543.36, 92660.54)	105388.23 (102433.85, 108090.78)	98726.68 (95897.84, 101338.31)	101941.55 (99491.98, 104365.94)
Gambia	117505.29 (104046.68, 131539.62)	115058.90 (102035.86, 127683.30)	116280.66 (103458.83, 128586.86)	132386.25 (118470.56, 146737.07)	122220.38 (109510.57, 135871.60)	127221.34 (114668.41, 139997.82)	124910.10 (120287.09, 129108.76)	114522.83 (110372.15, 118480.59)	119637.61 (115311.60, 123375.22)	118021.16 (113052.85, 123418.40)	109827.17 (104777.83, 114657.64)	113880.42 (109244.34, 118788.05)
United States of America	42608.32 (38093.13, 47511.74)	44458.11 (39718.57, 49829.77)	43555.01 (38999.85, 48780.08)	45415.97 (42165.60, 49157.31)	47734.69 (44342.52, 51749.00)	46594.42 (43309.79, 50300.07)	44599.70 (42384.93, 47319.99)	48679.37 (46048.12, 51933.35)	46670.17 (44330.47, 49377.95)	47253.10 (44522.43, 50885.25)	50925.02 (47739.67, 54802.69)	49110.88 (46347.82, 52685.23)
Venezuela (Bolivarian Republic of)	91026.67 (80626.58, 101624.92)	74893.98 (66574.81, 84400.77)	82933.08 (74108.12, 92291.03)	89908.18 (79243.27, 100920.48)	76198.61 (67563.78, 85587.04)	82926.45 (74129.71, 92341.81)	89540.99 (84678.21, 94148.23)	76590.63 (71441.21, 82331.00)	83033.75 (78372.90, 87797.93)	95873.07 (90123.83, 101746.73)	85386.80 (79623.36, 91554.23)	90534.46 (85228.90, 96372.52)

Viet Nam	62403.27 (53938.69, 70867.83)	51230.81 (44183.41, 58470.96)	56648.61 (49205.47, 64011.79)	60979.66 (54803.08, 67308.62)	48570.82 (44148.11, 53723.99)	54718.81 (49757.77, 60055.95)	63314.48 (59295.83, 67655.03)	55289.55 (51480.81, 59879.13)	59261.67 (55401.67, 63604.92)	75235.70 (70898.48, 81507.94)	70954.10 (66078.57, 78051.24)	73055.78 (68586.87, 79509.20)
United States Virgin Islands	76931.65 (68044.56, 86438.80)	68207.50 (60305.13, 76557.60)	72430.82 (64457.07, 81109.78)	121706.71 (107635.09, 135319.64)	120994.06 (108578.93, 134044.36)	121332.01 (109278.61, 133837.25)	120402.00 (117596.86, 123135.97)	119167.87 (116355.85, 121100.07)	119750.61 (117312.31, 121631.09)	121399.23 (117391.13, 126674.21)	131543.61 (128333.92, 134554.38)	126897.31 (123682.92, 130326.08)
Monaco	35352.31 (31696.85, 39421.16)	36294.07 (32108.32, 40787.42)	35846.12 (32007.18, 39773.16)	36907.79 (32840.03, 41740.48)	35026.73 (31230.94, 39237.31)	35942.34 (32268.34, 40029.57)	37074.65 (35583.98, 38655.99)	35417.74 (34030.32, 37074.82)	36224.24 (34907.72, 37605.44)	37192.31 (35121.66, 40022.23)	36204.09 (34367.58, 39863.09)	36685.11 (34988.36, 39841.84)
San Marino	34049.97 (30311.85, 38027.71)	33030.72 (29610.32, 36919.93)	33536.42 (30074.14, 37028.73)	34778.00 (31107.00, 38741.21)	33157.76 (29677.56, 36763.78)	33937.66 (30517.18, 37403.45)	34546.69 (33300.31, 35956.15)	33002.06 (31816.95, 34208.76)	33745.56 (32621.74, 34929.16)	35198.63 (32536.92, 39443.88)	34230.44 (31973.52, 39081.31)	34696.47 (32382.48, 39268.61)
Saint Kitts and Nevis	96996.21 (88114.20, 106634.32)	84984.89 (77441.46, 92763.07)	90879.76 (83589.16, 98908.18)	117106.86 (105125.55, 129250.13)	99599.44 (89696.56, 110963.59)	108379.08 (98495.97, 118871.54)	115038.68 (111896.81, 117458.11)	98970.34 (96238.62, 100909.86)	107028.31 (104407.10, 108936.92)	109313.40 (105458.47, 112481.32)	96048.90 (93280.21, 98405.84)	102700.79 (99597.93, 105207.54)
Cook Islands	94595.98 (83681.96, 106624.89)	74800.24 (66254.48, 83426.61)	85086.76 (76441.89, 94450.82)	176385.20 (155946.73, 198175.84)	130446.32 (117290.06, 147065.54)	152863.64 (138234.57, 169890.93)	172428.87 (167724.30, 175155.18)	128310.89 (125467.56, 130667.20)	149839.65 (146543.72, 151903.74)	169289.61 (163823.23, 173039.89)	126990.12 (123149.41, 129784.53)	147631.49 (143397.76, 150646.56)
Nauru	91156.39 (80272.90, 103338.76)	75641.62 (66780.18, 85116.58)	83607.63 (74347.18, 93772.77)	126410.61 (111323.11, 142810.43)	99620.21 (87745.10, 112479.64)	113149.47 (101086.05, 126771.49)	123988.34 (121449.15, 126238.55)	97653.53 (95528.68, 99601.45)	110952.72 (108744.32, 112905.28)	121728.27 (117271.16, 125201.39)	95678.33 (91004.52, 99195.14)	108833.65 (104586.14, 111954.80)
Niue	103330.68 (92443.79, 115169.33)	94312.22 (86404.37, 102318.78)	98848.99 (90372.06, 108008.38)	175480.63 (154981.28, 198375.00)	137264.65 (123509.25, 153642.39)	156467.29 (140506.27, 173340.86)	170087.13 (163806.24, 173117.28)	135778.11 (132904.98, 137202.30)	153017.59 (148656.20, 154936.90)	161886.17 (152878.64, 168734.85)	132279.67 (127684.56, 136137.44)	147156.24 (141017.47, 151968.15)

Palau	99437.59 (87771.74, 111031.67)	83146.54 (75017.88, 92515.64)	91528.42 (82179.13, 101352.75)	182132.44 (159723.99, 207585.51)	140598.80 (124588.07, 158757.26)	163257.96 (146175.57, 183385.19)	180013.74 (176254.08, 182729.59)	138001.74 (134397.24, 140163.04)	160921.87 (157718.63, 163023.37)	176270.53 (169290.43, 181445.62)	136037.91 (130489.62, 140016.03)	157987.28 (152091.68, 162232.19)
Tokelau	107610.79 (96579.39, 120105.73)	94584.14 (84083.73, 105673.65)	100982.64 (90514.98, 111884.08)	142838.52 (127835.96, 158141.00)	114012.27 (101978.84, 127389.16)	128776.97 (115961.67, 141548.56)	142292.88 (140365.71, 144232.44)	112917.67 (110990.80, 114564.64)	127963.54 (126440.41, 129481.75)	141239.66 (136221.25, 145586.46)	110608.03 (105905.94, 114788.59)	126297.44 (121684.32, 130167.63)
Tuvalu	103619.66 (91410.83, 116974.22)	91315.99 (82457.62, 100728.99)	97225.30 (87769.28, 107818.33)	141566.15 (126331.07, 157274.73)	115392.87 (103274.42, 128290.14)	128980.41 (116243.92, 141657.39)	141108.52 (139657.84, 142714.05)	114761.46 (113519.15, 116111.59)	128439.21 (127159.74, 129821.29)	138430.02 (131918.84, 143168.56)	112282.86 (106616.42, 117722.89)	125856.84 (120013.97, 130124.04)
**Region**												
Global	84906.78 (77075.53, 93146.48)	87262.41 (79468.11, 95565.82)	86076.30 (78360.66, 94276.62)	85418.19 (78445.37, 92740.92)	84704.62 (78081.55, 91769.29)	85062.53 (78254.29, 92311.98)	85619.44 (82337.40, 88717.19)	84951.02 (81838.97, 88178.80)	85286.43 (82187.31, 88433.12)	86582.30 (82222.06, 90597.66)	84960.06 (79635.24, 89843.55)	85771.87 (81461.60, 90258.62)
Andean Latin America	84095.14 (77456.28, 91115.04)	79035.50 (71765.80, 86250.03)	81550.50 (74825.71, 88335.27)	104531.13 (95261.01, 114447.05)	100070.46 (91488.82, 109298.98)	102301.11 (93821.04, 111465.88)	103925.93 (100784.43, 106433.80)	97984.81 (94776.37, 100418.60)	100957.67 (97945.37, 103280.34)	97537.05 (92638.64, 102084.65)	93135.56 (88605.67, 97837.44)	95340.12 (90849.40, 99758.34)
Australasia	31842.00 (28579.02, 35376.28)	29148.15 (26149.99, 32213.69)	30486.12 (27521.98, 33768.21)	37587.50 (33846.95, 41398.24)	31918.06 (28915.59, 35314.77)	34710.25 (31570.36, 38126.72)	37747.33 (36113.77, 40874.02)	31865.65 (29851.69, 35372.93)	34766.45 (33083.90, 37939.33)	38698.58 (36528.52, 42412.73)	33771.06 (31811.49, 38315.07)	36208.14 (34270.53, 40371.58)
Caribbean	88398.70 (81112.64, 95325.73)	76428.51 (70362.56, 82811.40)	82337.87 (75931.88, 88885.63)	115589.88 (106430.58, 125777.15)	105060.23 (97395.50, 113517.70)	110255.81 (101899.68, 119546.37)	110897.29 (106356.98, 115368.43)	100447.15 (96082.82, 104877.36)	105596.31 (101259.85, 109964.38)	116010.17 (110785.41, 120447.32)	107930.31 (102994.11, 112447.22)	111890.07 (106893.36, 116161.56)
Central Asia	74987.55 (66706.90, 82906.94)	74803.37 (66648.39, 82747.50)	74893.55 (66871.97, 82784.63)	86230.63 (76020.92, 96984.68)	89797.28 (79409.49, 101076.93)	88029.03 (78027.28, 98752.78)	85656.82 (84153.71, 87130.84)	88694.75 (87138.78, 90188.51)	87190.96 (85809.22, 88548.90)	83281.66 (81254.85, 85084.64)	86601.82 (84477.46, 88608.88)	84959.56 (83031.91, 86530.76)

Central Europe	95227.04 (83836.30, 107380.00)	103660.84 (91624.02, 117078.44)	99532.44 (88080.86, 112153.40)	71436.28 (64922.76, 78349.22)	83452.99 (75994.23, 91091.19)	77599.91 (70588.69, 84915.76)	73840.17 (71428.48, 76279.00)	87029.39 (84643.42, 89776.68)	80606.99 (78387.14, 82848.62)	72634.42 (69346.15, 76260.32)	84070.49 (79924.11, 88330.96)	78497.88 (75166.73, 81911.40)
Central Latin America	101188.94 (91654.20, 110787.50)	84917.91 (77170.45, 93036.65)	92953.91 (84614.65, 101803.10)	67196.33 (60422.29, 74299.27)	60370.55 (54573.64, 66799.20)	63706.18 (57627.93, 70270.91)	71809.13 (68811.58, 75364.24)	65639.10 (62391.55, 69325.28)	68664.42 (65767.79, 72091.19)	76784.28 (72569.77, 81721.40)	73353.71 (69304.87, 78477.96)	75035.94 (71190.96, 79786.31)
Central Sub-Saharan Africa	93714.08 (84499.45, 102569.56)	86259.17 (76671.89, 95676.19)	89949.20 (80570.19, 98876.15)	119803.66 (108327.51, 131162.93)	98344.86 (88258.27, 108554.75)	109000.90 (98571.40, 119008.10)	118060.10 (114891.97, 120227.56)	97178.68 (93871.18, 99545.19)	107549.14 (104400.75, 109675.10)	115838.72 (112082.43, 119797.12)	95809.11 (91994.39, 99971.19)	105762.41 (102380.41, 109278.32)
East Asia	47142.40 (42375.45, 52027.76)	44098.35 (39678.97, 48902.23)	45665.39 (41033.92, 50448.39)	54144.49 (48756.96, 60019.43)	48315.12 (43613.91, 53463.29)	51283.20 (46305.66, 56730.02)	54294.91 (52799.51, 55783.28)	49279.94 (47852.77, 50829.47)	51834.99 (50424.92, 53331.58)	68863.91 (65426.09, 72819.73)	65161.49 (61257.79, 69358.44)	67027.27 (63494.00, 70913.42)
Eastern Europe	93208.22 (83064.06, 103569.97)	94384.16 (84438.87, 104991.93)	93834.69 (83775.32, 104298.81)	94309.63 (84413.23, 105516.61)	105019.22 (94090.14, 117327.37)	100042.27 (90102.76, 112018.86)	94641.59 (93272.30, 96294.26)	105077.08 (103026.60, 107085.42)	100233.41 (98738.99, 101825.23)	94761.48 (88373.51, 100233.07)	104788.20 (97869.81, 110605.69)	100140.92 (94259.84, 105562.79)
Eastern Sub-Saharan Africa	123289.63 (111285.24, 135342.73)	123815.15 (111611.72, 135687.50)	123554.63 (111496.54, 135332.66)	118014.35 (106868.43, 129069.65)	104027.88 (94723.32, 114096.30)	110969.92 (100816.19, 121510.75)	115806.92 (112434.81, 119310.98)	103235.75 (100222.69, 106476.83)	109475.86 (106687.77, 112550.45)	129425.91 (125032.56, 133768.09)	121611.92 (117223.53, 126148.97)	125490.57 (121324.03, 129669.43)
High-income Asia Pacific	15533.53 (13728.99, 17768.24)	17272.38 (15024.77, 19883.54)	16412.37 (14470.33, 18782.76)	17517.23 (15517.52, 19879.43)	18702.11 (16489.46, 21081.53)	18117.69 (16057.32, 20488.11)	16372.36 (14697.61, 19046.65)	18444.85 (16251.44, 22369.24)	17425.85 (15582.96, 20439.83)	23488.45 (18537.66, 28633.80)	25480.60 (19334.39, 31789.87)	24502.09 (19347.74, 30198.97)
High-income North America	43757.13 (39118.63, 48921.15)	43748.75 (39106.95, 49028.75)	43752.85 (39293.16, 48949.97)	47720.49 (44242.98, 51577.91)	47511.49 (44169.06, 51653.56)	47614.28 (44156.54, 51601.60)	46679.11 (44903.55, 48247.06)	48510.30 (46503.80, 50295.04)	47608.48 (45908.21, 49099.96)	48388.20 (46195.11, 51131.29)	50280.35 (47634.08, 53358.67)	49345.43 (47149.64, 51986.67)

North Africa and Middle East	86173.93 (77290.03, 95200.54)	86113.46 (77117.69, 95411.52)	86144.44 (77391.98, 95340.50)	103066.86 (92799.34, 113853.05)	104511.87 (94342.32, 115461.15)	103761.22 (93651.46, 114553.98)	103378.87 (101725.13, 104943.73)	106088.27 (104247.16, 107834.05)	104685.26 (103113.57, 106232.00)	105728.71 (102889.17, 108388.01)	111477.44 (108392.52, 114324.66)	108537.31 (105922.96, 110961.14)
Oceania	112961.47 (102725.77, 124482.71)	90888.65 (82083.40, 99328.78)	102307.65 (93194.62, 111910.24)	154577.94 (140080.51, 170094.42)	116427.91 (104115.33, 128376.40)	136089.18 (124088.12, 148404.96)	151265.36 (148161.95, 153429.28)	115074.42 (112975.23, 117075.04)	133664.71 (131459.81, 135650.11)	144960.05 (139986.59, 148573.04)	113694.82 (110087.53, 117412.69)	129551.59 (125501.95, 132807.94)
South Asia	152528.16 (136221.77, 169964.10)	179560.69 (161091.12, 198543.98)	165506.94 (147780.81, 183758.15)	117887.94 (107091.56, 129714.25)	129655.37 (118467.85, 141729.28)	123648.50 (112634.62, 135607.78)	116706.21 (112399.03, 120584.38)	126003.47 (121352.83, 130227.71)	121252.46 (117109.81, 124934.60)	117135.97 (111698.79, 121591.28)	130965.45 (124534.00, 135868.78)	123903.51 (118347.16, 128438.46)
Southeast Asia	67362.60 (61286.69, 73418.04)	57149.10 (51435.79, 62872.87)	62222.18 (56223.81, 68050.04)	69162.09 (63940.99, 75368.41)	59094.49 (54171.00, 64114.57)	64124.30 (59061.07, 69577.90)	66501.33 (63932.57, 69103.73)	57746.92 (55341.99, 60414.68)	62116.62 (59812.32, 64690.59)	80204.97 (75865.66, 84723.71)	75735.60 (70884.97, 81514.31)	77951.97 (73846.07, 83016.57)
Southern Latin America	49961.45 (44586.00, 55650.33)	38925.56 (34600.44, 43336.59)	44329.65 (39945.14, 49176.46)	55632.51 (49998.37, 61972.25)	42295.54 (37982.67, 46905.42)	48802.88 (44065.43, 53933.89)	57140.49 (55181.55, 59574.33)	46017.58 (43892.37, 48920.72)	51448.75 (49580.95, 53955.17)	57202.28 (54868.92, 59957.05)	47036.05 (44451.72, 50687.06)	52019.81 (49696.81, 55156.62)
Southern Sub-Saharan Africa	106950.67 (97063.82, 117211.56)	96220.73 (87479.04, 105186.38)	101416.22 (92329.01, 110911.24)	104104.16 (95298.00, 113460.59)	92600.18 (85087.45, 100666.61)	98213.43 (90157.95, 106757.38)	101067.67 (97627.30, 104597.22)	92205.79 (88997.31, 95453.07)	96524.12 (93336.20, 99891.14)	108652.15 (104233.66, 112557.02)	100654.57 (96683.78, 104675.43)	104546.58 (100569.78, 108147.50)
Tropical Latin America	92184.97 (82926.87, 101135.70)	89737.91 (80650.59, 99236.35)	90948.01 (81753.96, 100088.40)	80971.98 (73791.37, 88526.42)	84374.80 (76777.95, 92158.69)	82711.31 (75353.17, 90368.16)	86938.72 (83826.48, 90409.31)	90268.18 (86883.16, 94692.89)	88640.34 (85634.18, 91980.75)	88661.34 (84436.87, 93870.65)	92695.44 (88315.39, 97957.05)	90733.03 (86716.83, 95781.93)
Western Europe	34955.58 (31446.98, 38537.34)	34128.59 (31159.74, 37467.28)	34531.25 (31364.48, 37935.42)	39649.02 (36151.11, 43309.29)	40140.08 (36875.27, 43617.90)	39898.60 (36583.52, 43438.01)	39493.04 (37527.32, 41018.97)	39919.39 (37823.12, 41508.34)	39709.90 (37824.56, 41167.31)	45064.74 (42382.34, 47376.82)	45929.03 (43322.69, 48638.50)	45501.52 (42970.27, 47988.00)

Western Sub-Saharan Africa	124851.51 (113899.94, 135568.41)	130064.37 (117786.39, 142075.14)	127476.22 (115997.08, 138789.07)	126221.60 (114975.29, 137030.35)	123140.72 (111841.16, 134724.58)	124645.85 (113477.97, 135668.08)	125543.44 (121841.95, 128570.29)	121776.51 (118259.39, 124317.22)	123625.55 (120077.26, 126285.01)	125701.16 (120254.53, 130891.35)	124909.26 (119594.63, 129560.07)	125302.35 (120095.60, 130003.71)
High SDI	38118.57 (34495.91, 42024.77)	38152.62 (34800.73, 42013.74)	38135.85 (34670.60, 42025.56)	44241.74 (41095.18, 47541.67)	43580.78 (40637.18, 46817.05)	43910.62 (40897.03, 47205.79)	41874.07 (39752.16, 43677.06)	41497.05 (39038.21, 43599.56)	41685.20 (39518.03, 43545.09)	41794.28 (39559.63, 43856.99)	41678.86 (39252.45, 44123.08)	41736.46 (39459.69, 43867.99)
High-middle SDI	65031.36 (59061.53, 71219.35)	65027.88 (58979.81, 71367.49)	65029.61 (58936.09, 71169.83)	66429.88 (60818.20, 72636.74)	66761.73 (61251.38, 72838.95)	66595.96 (61046.41, 72844.36)	65516.16 (63236.82, 67331.31)	67367.60 (65774.42, 68962.43)	66442.78 (64774.78, 67939.00)	61179.10 (57859.81, 65740.67)	63441.64 (60147.73, 67854.94)	62311.47 (59212.04, 66519.58)
Middle SDI	72540.44 (66128.92, 79044.22)	70150.82 (64012.50, 76613.96)	71365.75 (65052.71, 77861.00)	76010.53 (69818.17, 82728.57)	73013.01 (67206.79, 79185.22)	74521.28 (68604.55, 80978.78)	75110.84 (73092.14, 76507.31)	72240.67 (70262.42, 73771.32)	73684.86 (71728.48, 74937.96)	74437.14 (71703.99, 77476.25)	71797.77 (69001.55, 75242.85)	73125.83 (70586.86, 76014.98)
Low-middle SDI	131560.79 (118200.90, 145158.55)	144416.38 (130244.17, 158881.81)	137859.27 (124233.18, 151973.82)	109786.36 (100598.28, 119904.27)	114083.17 (104771.98, 124078.37)	111923.84 (102831.78, 121910.69)	110491.82 (107703.20, 112741.61)	114118.33 (111132.12, 116594.93)	112295.85 (109603.57, 114607.95)	106072.71 (98833.05, 111504.90)	109709.78 (102890.30, 115260.46)	107882.00 (101152.58, 113207.26)
Low SDI	138311.94 (124536.48, 152398.07)	149067.94 (134039.50, 163321.73)	143642.46 (129304.27, 158071.52)	127971.13 (116487.94, 140116.64)	123545.96 (112270.79, 135683.18)	125765.70 (114589.60, 137679.01)	127088.48 (125093.58, 129102.15)	122806.38 (120689.93, 124960.39)	124954.35 (123003.44, 126814.99)	132287.57 (128040.75, 136910.78)	128026.55 (124089.36, 132742.56)	130163.95 (126256.06, 134537.28)

Abbreviations: UIs, uncertainty intervals.

Table S5. Temporal trends of incident cases and incidence rates for diarrheal diseases for males, females, and both genders combined in all age groups (four age groups) between 1990 and 2040 at the global level

Year	<20 years, incidence cases × 100,000 (incident rate per 100,000)	20-39 years, incidence cases × 100,000 (incident rate per 100,000)	40-59 years, incidence cases × 100,000 (incident rate per 100,000)	≥60 years, incidence cases × 100,000 (incident rate per 100,000)

	Male	Female	Both genders combined	Male	Female	Both genders combined	Male	Female	Both genders combined	Male	Female	Both genders combined
1990	12083.01 (103737.76)	11916.94 (107475.38)	23999.95 (105560.57)	4993.14 (59054.54)	5203.75 (62818.51)	10196.88 (60917.26)	3295.37 (70807.20)	3331.60 (73821.00)	6626.97 (72290.94)	2500.25 (114652.23)	2725.47 (101846.24)	5225.71 (107596.20)
1991	12006.42 (102316.30)	11838.82 (106067.12)	23845.24 (104144.77)	5084.90 (58968.86)	5257.18 (62314.62)	10342.08 (60623.45)	3378.76 (70886.95)	3399.46 (73510.65)	6778.22 (72178.97)	2575.52 (114829.75)	2799.16 (102235.18)	5374.68 (107906.56)
1992	11944.48 (101024.17)	11763.26 (104717.41)	23707.73 (102823.53)	5177.52 (58960.17)	5318.67 (61962.97)	10496.19 (60444.47)	3460.13 (70857.50)	3466.33 (73109.71)	6926.46 (71967.00)	2649.87 (115103.81)	2872.43 (102709.91)	5522.30 (108305.88)
1993	11906.27 (99934.88)	11702.40 (103528.85)	23608.67 (101684.61)	5269.42 (59040.55)	5387.83 (61775.11)	10657.25 (60392.07)	3540.53 (70726.47)	3531.75 (72615.59)	7072.29 (71657.41)	2724.25 (115433.67)	2948.94 (103263.42)	5673.19 (108770.19)
1994	11905.46 (99138.68)	11672.93 (102609.53)	23578.40 (100827.14)	5360.00 (59206.87)	5465.59 (61743.67)	10825.58 (60461.03)	3622.54 (70539.44)	3600.14 (72094.91)	7222.68 (71306.28)	2799.75 (115812.02)	3029.67 (103934.78)	5829.42 (109319.37)
1995	11949.82 (98705.98)	11684.58 (102043.29)	23634.39 (100328.17)	5449.94 (59441.59)	5551.00 (61839.21)	11000.94 (60627.71)	3706.00 (70320.62)	3671.41 (71593.18)	7377.41 (70948.21)	2878.48 (116256.66)	3117.21 (104717.77)	5995.69 (109957.32)
1996	12038.69 (98618.55)	11716.45 (101627.58)	23755.14 (100080.06)	5564.77 (59960.29)	5658.33 (62171.36)	11223.10 (61055.03)	3785.87 (70068.50)	3736.02 (71039.08)	7521.89 (70547.24)	2958.61 (116622.33)	3207.45 (105403.45)	6166.06 (110504.11)
1997	12154.70 (98762.46)	11741.85 (101168.87)	23896.55 (99930.40)	5714.74 (60834.18)	5792.51 (62757.44)	11507.25 (61787.34)	3857.93 (69725.84)	3787.94 (70326.89)	7645.87 (70022.32)	3036.78 (116850.64)	3294.61 (105867.45)	6331.38 (110865.59)
1998	12280.84 (99040.77)	11764.00 (100738.51)	24044.84 (99864.18)	5880.22 (61828.63)	5940.36 (63435.57)	11820.59 (62625.88)	3926.63 (69374.90)	3832.70 (69549.29)	7759.33 (69460.93)	3113.18 (117005.96)	3380.16 (106227.74)	6493.34 (111136.03)
1999	12403.51 (99361.65)	11791.19 (100417.52)	24194.69 (99873.44)	6039.88 (62721.23)	6087.47 (64062.74)	12127.35 (63387.52)	3998.82 (69078.08)	3879.15 (68776.77)	7877.96 (68929.38)	3191.31 (117233.57)	3466.42 (106643.99)	6657.73 (111470.46)
2000	12510.09 (99623.17)	11833.83 (100289.40)	24343.92 (99945.92)	6171.24 (63294.87)	6218.41 (64507.57)	12389.65 (63897.77)	4080.43 (68868.19)	3934.75 (68102.44)	8015.18 (68490.13)	3272.39 (117536.25)	3557.15 (107130.78)	6829.54 (111876.49)
2001	12596.45 (99782.88)	11881.80 (100252.41)	24478.25 (100010.24)	6246.85 (63294.27)	6293.72 (64400.99)	12540.56 (63844.90)	4158.72 (68488.74)	3992.61 (67338.57)	8151.34 (67920.51)	3354.07 (117844.74)	3649.55 (107662.45)	7003.62 (112309.77)

2002	12665.93 (99865.82)	11917.11 (100142.84)	24583.04 (99999.92)	6267.63 (62758.88)	6300.21 (63646.86)	12567.84 (63200.90)	4221.11 (67696.04)	4042.30 (66284.99)	8263.41 (66998.35)	3434.62 (118195.30)	3740.55 (108218.09)	7175.17 (112774.98)
2003	12714.45 (99847.51)	11936.32 (99937.43)	24650.78 (99891.03)	6249.38 (61893.34)	6259.85 (62511.98)	12509.23 (62201.38)	4277.27 (66670.68)	4093.31 (65097.91)	8370.59 (65892.19)	3515.84 (118548.42)	3831.65 (108827.73)	7347.49 (113272.14)
2004	12738.29 (99700.20)	11936.85 (99617.74)	24675.14 (99660.29)	6211.74 (60877.05)	6199.14 (61241.93)	12410.88 (61058.76)	4334.33 (65599.33)	4149.17 (63933.18)	8483.50 (64773.72)	3602.11 (118992.50)	3929.20 (109528.17)	7531.31 (113859.55)
2005	12732.69 (99405.88)	11915.18 (99181.71)	24647.87 (99297.38)	6179.11 (59908.60)	6149.37 (60102.65)	12328.48 (60005.23)	4400.20 (64661.79)	4216.98 (62946.49)	8617.18 (63810.85)	3694.01 (119524.88)	4032.71 (110299.54)	7726.72 (114525.54)
2006	12640.57 (98511.61)	11825.43 (98257.31)	24466.01 (98388.53)	6132.41 (58715.77)	6090.47 (58825.33)	12222.88 (58770.31)	4461.85 (63805.14)	4284.04 (62077.04)	8745.89 (62946.80)	3792.94 (119838.41)	4145.64 (110952.42)	7938.58 (115027.58)
2007	12453.61 (96911.97)	11660.91 (96750.84)	24114.52 (96833.99)	6053.63 (57171.42)	6000.11 (57222.41)	12053.74 (57196.79)	4505.70 (62898.49)	4336.85 (61199.47)	8842.54 (62053.57)	3902.45 (119590.54)	4273.68 (111312.87)	8176.13 (115115.96)
2008	12233.94 (95108.31)	11472.26 (95088.45)	23706.20 (95098.70)	5973.93 (55659.13)	5910.68 (55667.72)	11884.61 (55663.40)	4553.33 (62083.51)	4394.94 (60465.09)	8948.28 (61277.94)	4013.72 (119333.67)	4409.09 (111676.91)	8422.81 (115199.17)
2009	12046.86 (93588.25)	11313.57 (93691.14)	23360.43 (93638.05)	5928.44 (54564.66)	5858.42 (54537.87)	11786.86 (54551.34)	4614.57 (61578.77)	4466.91 (60032.45)	9081.48 (60808.35)	4135.64 (118950.45)	4557.63 (112010.04)	8693.27 (115207.91)
2010	11966.16 (92866.79)	11246.11 (93015.21)	23212.27 (92938.64)	5959.83 (54256.04)	5884.86 (54194.66)	11844.69 (54225.53)	4716.18 (61490.38)	4572.22 (59981.24)	9288.39 (60738.13)	4257.42 (119006.44)	4712.58 (112635.84)	8969.99 (115572.26)
2011	11965.13 (92682.39)	11246.66 (92819.17)	23211.78 (92748.61)	6080.37 (54801.07)	6008.60 (54799.87)	12088.97 (54800.47)	4851.29 (61992.55)	4714.47 (60570.19)	9565.76 (61283.29)	4413.95 (119272.03)	4904.55 (113461.40)	9318.51 (116141.52)
2012	11971.30 (92466.92)	11253.75 (92591.62)	23225.05 (92527.30)	6253.00 (55832.29)	6198.23 (56036.08)	12451.23 (55933.55)	5026.33 (63004.61)	4905.45 (61773.69)	9931.78 (62390.57)	4586.05 (119834.02)	5115.03 (114560.54)	9701.09 (116994.43)
2013	11987.51 (92272.09)	11269.86 (92380.93)	23257.37 (92324.80)	6451.64 (57100.50)	6419.61 (57570.97)	12871.25 (57334.19)	5220.36 (64290.28)	5120.59 (63295.97)	10340.95 (63794.04)	4768.70 (120452.10)	5338.01 (115682.29)	10106.71 (117884.89)
2014	12016.24 (92165.10)	11296.54 (92245.95)	23312.78 (92204.26)	6648.54 (58352.44)	6635.66 (59060.13)	13284.21 (58703.81)	5412.58 (65590.10)	5333.05 (64808.26)	10745.62 (65199.73)	4960.02 (121057.94)	5566.01 (116670.78)	10526.03 (118697.76)

2015	12073.13 (92236.26)	11346.68 (92267.90)	23419.81 (92251.59)	6814.13 (59320.74)	6806.14 (60139.20)	13620.27 (59726.93)	5581.08 (66628.18)	5514.56 (65968.39)	11095.64 (66298.62)	5149.76 (121443.38)	5784.78 (117291.70)	10934.54 (119211.05)
2016	12320.51 (93693.62)	11557.72 (93537.01)	23878.22 (93617.75)	7018.28 (60598.61)	6991.85 (61329.31)	14010.13 (60961.08)	5844.01 (68773.20)	5733.16 (67565.40)	11577.17 (68169.73)	5447.82 (124299.02)	6057.00 (118956.70)	11504.82 (121427.99)
2017	12584.93 (95288.02)	11783.84 (94940.49)	24368.76 (95119.65)	7227.61 (61905.62)	7183.01 (62551.14)	14410.62 (62225.71)	6136.22 (71191.23)	5967.32 (69292.21)	12103.54 (70242.14)	5778.06 (127771.04)	6349.08 (120957.09)	12127.14 (124110.63)
2018	12674.26 (95629.08)	11856.00 (95173.39)	24530.26 (95408.29)	7383.87 (62763.43)	7325.85 (63359.92)	14709.72 (63059.09)	6354.86 (72666.76)	6163.62 (70501.95)	12518.48 (71584.52)	6037.92 (129636.32)	6629.01 (122699.10)	12666.94 (125910.82)
2019	12736.24 (95799.81)	11899.06 (95206.99)	24635.31 (95512.55)	7537.22 (63612.72)	7457.27 (64095.50)	14994.49 (63851.91)	6579.00 (74132.49)	6369.28 (71757.79)	12948.28 (72945.05)	6298.12 (131433.96)	6940.63 (124919.47)	13238.76 (127936.16)
2020	13038.04 (96233.77)	12185.35 (96023.59)	25223.38 (96132.12)	7526.78 (62675.45)	7414.54 (62901.87)	14941.32 (62787.60)	6680.98 (73548.92)	6474.22 (71244.30)	13155.20 (72396.38)	6637.75 (134546.07)	7305.14 (127543.49)	13942.90 (130783.98)
2021	12305.53 (90523.76)	11385.93 (89456.23)	23691.46 (90007.55)	7547.44 (62429.51)	7450.29 (62875.12)	14997.73 (62650.08)	6544.81 (70995.86)	6353.36 (68865.92)	12898.17 (69930.48)	6751.72 (133141.45)	7429.02 (126112.84)	14180.74 (129364.36)
2022	12968.78 (95122.40)	12115.14 (94930.88)	25083.92 (95029.80)	7640.15 (62818.40)	7504.92 (63055.53)	15145.08 (62935.68)	6836.15 (73089.57)	6633.30 (70839.19)	13469.45 (71963.73)	7013.28 (134477.67)	7705.91 (127132.25)	14719.19 (130529.37)
2023	12370.30 (90509.26)	11499.09 (89892.69)	23869.39 (90211.17)	7664.00 (62696.98)	7543.31 (63168.37)	15207.31 (62929.92)	6756.58 (71228.30)	6561.20 (69064.30)	13317.79 (70145.48)	7270.29 (135343.92)	7974.88 (127692.53)	15245.17 (131230.52)
2024	12942.18 (94517.09)	12116.00 (94531.64)	25058.17 (94524.13)	7658.47 (62387.24)	7486.34 (62551.23)	15144.81 (62468.20)	7022.86 (73036.94)	6797.41 (70560.75)	13820.27 (71797.69)	7564.90 (136517.54)	8291.31 (128677.74)	15856.21 (132302.58)
2025	12439.68 (90734.59)	11542.87 (89930.04)	23982.56 (90345.57)	7798.60 (63288.61)	7595.06 (63358.13)	15393.66 (63322.89)	6956.64 (71404.30)	6717.67 (68794.91)	13674.31 (70098.12)	7781.72 (136078.33)	8503.26 (127873.40)	16284.98 (131667.00)
2026	13067.92 (95256.37)	12185.06 (94848.99)	25252.98 (95059.36)	7809.29 (63140.84)	7553.37 (62922.44)	15362.65 (63033.27)	7225.20 (73222.25)	6967.03 (70408.36)	14192.23 (71813.33)	8056.22 (136592.30)	8803.52 (128360.17)	16859.74 (132166.33)
2027	12397.43 (90362.00)	11504.25 (89521.98)	23901.68 (89955.72)	7846.64 (63190.77)	7606.64 (63263.95)	15453.28 (63226.77)	7108.94 (71158.33)	6884.07 (68665.76)	13993.00 (69909.86)	8246.74 (135760.08)	9011.11 (127564.50)	17257.84 (131353.68)

2028	12460.89<br>(90874.82)	11608.66<br>(90365.00)	24069.54 (90628.22)	7838.30<br>(62871.21)	7560.72<br>(62788.87)	15399.02 (62830.76)	7161.44<br>(70823.78)	6924.36<br>(68167.11)	14085.80 (69492.41)	8472.63<br>(135522.50)	9252.15<br>(127276.91)	17724.78<br>(131089.46)
2029	12299.50<br>(89808.90)	11411.82<br>(88920.66)	23711.32 (89379.20)	7869.53<br>(62883.59)	7596.19<br>(63017.51)	15465.73 (62949.30)	7256.13<br>(70923.32)	7050.26<br>(68504.62)	14306.39 (69710.39)	8711.63<br>(135394.64)	9526.63<br>(127381.41)	18238.26<br>(131087.22)
2030	12675.70<br>(92725.07)	11799.83<br>(92086.94)	24475.53 (92416.32)	8130.84<br>(64719.56)	7714.44<br>(63928.04)	15845.28 (64331.77)	7410.00<br>(71616.55)	7120.14<br>(68313.24)	14530.14 (69958.85)	9003.22<br>(136052.73)	9829.03<br>(127831.97)	18832.26<br>(131634.48)
2031	12394.67<br>(90878.32)	11472.78<br>(89715.38)	23867.45 (90315.57)	8143.16<br>(64538.18)	7705.85<br>(63752.01)	15849.02 (64153.54)	7469.76<br>(71429.54)	7170.08<br>(67979.54)	14639.84 (69697.16)	9251.53<br>(136104.95)	10083.11<br>(127689.33)	19334.63<br>(131582.35)
2032	12516.99<br>(92019.77)	11612.55<br>(91024.17)	24129.54 (91537.93)	8198.12<br>(64645.13)	7742.26<br>(63882.78)	15940.38 (64272.60)	7558.11<br>(71562.59)	7258.83<br>(68083.87)	14816.94 (69815.03)	9484.29<br>(136074.27)	10356.26<br>(127884.47)	19840.55<br>(131672.77)
2033	12277.38<br>(90538.08)	11334.25<br>(89094.60)	23611.63 (89839.37)	8205.12<br>(64324.54)	7775.86<br>(63925.05)	15980.98 (64129.54)	7595.69<br>(71264.87)	7334.35<br>(68127.14)	14930.04 (69688.15)	9712.91<br>(136103.43)	10623.45<br>(128079.67)	20336.36<br>(131790.48)
2034	12310.68<br>(91110.87)	11360.98<br>(89603.96)	23671.66 (90381.37)	8304.36<br>(64699.17)	7803.63<br>(63885.35)	16107.99 (64302.34)	7662.33<br>(71278.05)	7371.96<br>(67861.91)	15034.29 (69561.03)	9960.49<br>(136433.50)	10878.60<br>(128158.52)	20839.09<br>(131984.75)
2035	12209.80<br>(90722.98)	11242.78<br>(88999.19)	23452.57 (89888.37)	8351.66<br>(64656.14)	7845.75<br>(63944.48)	16197.41 (64309.46)	7687.54<br>(70932.95)	7398.05<br>(67531.77)	15085.58 (69223.22)	10220.89<br>(136965.29)	11166.93<br>(128651.77)	21387.82<br>(132495.00)
2036	12166.83<br>(90782.58)	11190.03<br>(88923.34)	23356.86 (89882.24)	8450.57<br>(65016.76)	7943.09<br>(64448.48)	16393.66 (64740.17)	7796.59<br>(71374.16)	7490.00<br>(67833.95)	15286.58 (69594.53)	10472.77<br>(137410.81)	11423.84<br>(128807.30)	21896.61<br>(132783.64)
2037	12108.37<br>(90759.84)	11150.89<br>(88979.56)	23259.26 (89897.54)	8500.22<br>(64998.96)	7977.48<br>(64432.88)	16477.70 (64723.66)	7794.14<br>(70797.96)	7480.76<br>(67247.46)	15274.90 (69013.47)	10709.06<br>(137689.29)	11673.79<br>(128918.16)	22382.85<br>(132970.89)
2038	12088.30<br>(90956.26)	11127.03<br>(89102.14)	23215.33 (90058.05)	8515.58<br>(64804.66)	7982.91<br>(64239.85)	16498.49 (64530.13)	7834.39<br>(70606.09)	7505.95<br>(66996.14)	15340.33 (68792.40)	10941.39<br>(137964.93)	11941.64<br>(129263.37)	22883.04<br>(133282.77)
2039	12214.61<br>(92276.54)	11266.98<br>(90561.52)	23481.59 (91445.60)	8673.60<br>(65716.40)	8186.43<br>(65639.40)	16860.03 (65678.99)	7891.88<br>(70568.44)	7609.50<br>(67466.42)	15501.38 (69010.82)	11226.20<br>(138915.48)	12315.47<br>(130750.08)	23541.67<br>(134520.69)
2040	11983.07<br>(90906.74)	11002.11<br>(88782.00)	22985.18 (89877.16)	8525.45<br>(64333.64)	7970.33<br>(63684.23)	16495.78 (64018.22)	7877.48<br>(69906.37)	7550.78<br>(66533.98)	15428.26 (68214.20)	11385.11<br>(138299.85)	12436.39<br>(129546.30)	23821.50<br>(133587.36)

Table S6. Temporal trends of incident cases and incidence rates for diarrheal diseases for males, females, and both genders combined in all age groups (17 age groups) between 1990 and 2040 at the global level

Age group	Year	Incidence cases × 100,000 (incident rate per 100,000) of diarrheal diseases		
		Male	Female	Both genders combined
<5 years				
	1990	5601.74 (172154.35)	5244.80 (170993.11)	10846.54 (171590.87)
	1991	5478.08 (167470.79)	5126.91 (166475.56)	10604.99 (166988.17)
	1992	5355.61 (163396.31)	5008.89 (162527.13)	10364.50 (162975.10)
	1993	5244.37 (160146.24)	4901.33 (159392.41)	10145.70 (159781.18)
	1994	5158.56 (157943.39)	4818.28 (157301.26)	9976.84 (157632.62)
	1995	5109.35 (156992.44)	4770.36 (156460.38)	9879.71 (156735.09)
	1996	5084.23 (156685.86)	4743.29 (156174.08)	9827.53 (156438.43)
	1997	5063.24 (156360.46)	4717.11 (155735.82)	9780.35 (156058.57)
	1998	5046.83 (156081.87)	4694.40 (155291.95)	9741.24 (155700.20)
	1999	5038.11 (155965.51)	4680.63 (155048.18)	9718.74 (155522.37)
	2000	5042.66 (156143.01)	4683.67 (155235.51)	9726.34 (155704.69)
	2001	5064.60 (156745.37)	4703.35 (155853.16)	9767.94 (156314.49)
	2002	5100.95 (157653.95)	4731.99 (156626.07)	9832.93 (157157.62)
	2003	5144.12 (158583.39)	4764.49 (157328.30)	9908.61 (157977.40)
	2004	5184.67 (159249.69)	4794.61 (157759.46)	9979.27 (158530.20)
	2005	5212.08 (159403.18)	4815.28 (157759.71)	10027.36 (158609.71)
	2006	5216.70 (158697.33)	4817.94 (156997.66)	10034.64 (157876.69)
	2007	5202.49 (157232.84)	4804.51 (155519.25)	10007.00 (156405.43)
	2008	5174.41 (155366.40)	4779.78 (153676.32)	9954.19 (154550.25)
	2009	5138.75 (153426.50)	4749.80 (151800.71)	9888.55 (152641.25)

	2010	5107.64 (151769.77)	4725.95 (150249.95)	9833.58 (151035.54)
	2011	5072.96 (150120.55)	4700.18 (148746.39)	9773.14 (149456.52)
	2012	5025.61 (148133.75)	4662.86 (146930.84)	9688.47 (147552.37)
	2013	4972.47 (146076.04)	4619.69 (145041.02)	9592.17 (145575.72)
	2014	4922.57 (144313.80)	4578.09 (143397.42)	9500.65 (143870.77)
	2015	4896.44 (143163.83)	4555.58 (142293.71)	9452.02 (142743.14)
	2016	4952.20 (144198.42)	4595.92 (142985.49)	9548.12 (143612.03)
	2017	5006.84 (145538.60)	4635.47 (143984.38)	9642.32 (144787.25)
	2018	4974.24 (144888.76)	4601.61 (143222.40)	9575.85 (144083.19)
	2019	4927.49 (143910.85)	4549.21 (141965.85)	9476.70 (142970.56)
	2020	4954.98 (141871.86)	4587.22 (140486.27)	9542.20 (141202.37)
	2021	4461.74 (128242.84)	4029.03 (123809.98)	8490.77 (126100.45)
	2022	4817.38 (139176.74)	4444.23 (137141.10)	9261.61 (138192.44)
	2023	4434.83 (128383.81)	4020.68 (124255.57)	8455.51 (126387.12)
	2024	4786.09 (138931.55)	4428.41 (137163.74)	9214.50 (138076.31)
	2025	4438.04 (129287.90)	4006.71 (124488.77)	8444.75 (126965.60)
	2026	4805.79 (140590.54)	4424.61 (137992.55)	9230.40 (139333.09)
	2027	4403.46 (129428.36)	3975.81 (124529.50)	8379.27 (127056.76)
	2028	4496.97 (132849.25)	4102.79 (129110.01)	8599.76 (131038.68)
	2029	4308.87 (127956.58)	3886.95 (122911.40)	8195.82 (125513.20)
	2030	4468.92 (133393.97)	4068.09 (129259.27)	8537.01 (131391.19)
	2031	4263.87 (127909.10)	3809.75 (121618.36)	8073.61 (124861.49)
	2032	4404.91 (132781.34)	3973.97 (127440.94)	8378.88 (130193.76)
	2033	4205.76 (127383.15)	3732.00 (120223.63)	7937.76 (123913.73)
	2034	4237.72 (128947.71)	3762.25 (121736.68)	7999.97 (125452.96)

	2035	4172.63 (127533.46)	3685.51 (119765.66)	7858.14 (123768.55)
	2036	4152.02 (127445.99)	3670.20 (119763.71)	7822.22 (123722.31)
	2037	4132.07 (127353.97)	3661.40 (119957.43)	7793.47 (123768.65)
	2038	4129.18 (127777.20)	3645.90 (119925.37)	7775.08 (123971.10)
	2039	4143.77 (128749.14)	3654.17 (120682.75)	7797.95 (124838.99)
	2040	4123.16 (128635.98)	3613.61 (119834.20)	7736.78 (124369.35)
**5-9 years**				
	1990	2738.02 (91106.72)	2767.79 (97240.53)	5505.81 (94090.31)
	1991	2738.52 (89819.64)	2763.76 (95964.36)	5502.28 (92804.47)
	1992	2740.01 (88604.72)	2755.98 (94594.30)	5495.98 (91510.29)
	1993	2742.52 (87519.93)	2746.16 (93231.56)	5488.68 (90287.40)
	1994	2747.12 (86650.05)	2736.88 (92012.68)	5484.00 (89245.89)
	1995	2752.88 (86080.49)	2728.42 (91078.80)	5481.30 (88497.99)
	1996	2759.35 (85950.97)	2715.10 (90419.44)	5474.46 (88110.56)
	1997	2766.22 (86236.82)	2694.20 (89929.24)	5460.42 (88020.00)
	1998	2771.91 (86810.04)	2669.95 (89645.82)	5441.86 (88178.59)
	1999	2776.62 (87509.63)	2648.42 (89576.81)	5425.04 (88506.74)
	2000	2779.93 (88173.81)	2634.69 (89740.16)	5414.62 (88929.09)
	2001	2784.58 (88695.62)	2629.75 (89975.37)	5414.33 (89312.62)
	2002	2792.78 (89129.74)	2629.75 (90147.87)	5422.53 (89620.61)
	2003	2802.03 (89450.95)	2631.48 (90216.94)	5433.52 (89820.29)
	2004	2810.24 (89633.12)	2632.11 (90136.35)	5442.35 (89875.79)
	2005	2816.27 (89643.80)	2630.32 (89866.58)	5446.59 (89751.25)
	2006	2800.31 (88869.71)	2612.38 (88955.83)	5412.69 (88911.26)
	2007	2756.39 (87130.46)	2574.78 (87294.77)	5331.17 (87209.74)

	2008	2704.17 (85058.77)	2532.24 (85389.51)	5236.42 (85218.39)
	2009	2662.56 (83272.11)	2498.73 (83734.59)	5161.29 (83495.37)
	2010	2650.00 (82372.56)	2487.59 (82809.49)	5137.59 (82583.54)
	2011	2657.99 (82073.46)	2491.95 (82365.41)	5149.93 (82214.47)
	2012	2667.02 (81753.81)	2497.23 (81904.70)	5164.25 (81826.70)
	2013	2678.39 (81507.18)	2505.01 (81528.29)	5183.40 (81517.38)
	2014	2693.04 (81415.78)	2516.54 (81329.78)	5209.58 (81374.21)
	2015	2712.99 (81565.62)	2533.95 (81397.92)	5246.94 (81484.55)
	2016	2800.87 (83809.87)	2610.69 (83425.03)	5411.56 (83623.77)
	2017	2898.50 (86338.66)	2693.75 (85657.94)	5592.25 (86009.42)
	2018	2940.15 (87236.27)	2727.01 (86351.92)	5667.16 (86808.47)
	2019	2973.19 (87977.50)	2753.76 (86936.86)	5726.96 (87474.03)
	2020	3144.40 (90682.13)	2956.71 (91141.98)	6101.11 (90904.40)
	2021	3016.62 (86713.17)	2830.84 (86980.44)	5847.46 (86842.35)
	2022	3153.74 (90342.29)	2973.95 (91104.30)	6127.70 (90710.52)
	2023	3028.75 (86854.10)	2851.67 (87458.97)	5880.43 (87146.38)
	2024	3128.96 (89935.55)	2952.46 (90762.27)	6081.41 (90335.02)
	2025	3024.57 (87209.49)	2841.96 (87627.04)	5866.53 (87411.27)
	2026	3133.82 (90694.82)	2941.24 (90981.41)	6075.05 (90833.35)
	2027	2992.92 (87049.90)	2811.31 (87314.01)	5804.23 (87177.62)
	2028	2970.94 (86572.00)	2792.61 (86848.15)	5763.55 (86705.58)
	2029	3006.50 (87834.85)	2808.28 (87518.31)	5814.78 (87681.69)
	2030	3045.11 (89267.18)	2867.22 (89620.09)	5912.33 (89437.98)
	2031	3014.78 (88737.13)	2830.98 (88808.57)	5845.75 (88771.71)
	2032	2997.77 (88640.01)	2802.26 (88273.24)	5800.02 (88462.43)

	2033	2971.60 (88300.72)	2780.15 (87975.46)	5751.75 (88143.21)
	2034	2965.99 (88581.91)	2772.13 (88134.65)	5738.13 (88365.27)
	2035	2950.36 (88556.79)	2748.35 (87787.27)	5698.71 (88183.99)
	2036	2934.69 (88513.86)	2734.46 (87739.95)	5669.15 (88138.87)
	2037	2915.26 (88342.54)	2716.90 (87563.57)	5632.16 (87965.05)
	2038	2907.53 (88516.96)	2710.06 (87727.64)	5617.59 (88134.41)
	2039	2942.25 (89978.86)	2744.01 (89210.13)	5686.26 (89606.25)
	2040	2891.88 (88821.51)	2692.22 (87891.44)	5584.10 (88370.66)
**10-14 years**				
	1990	2149.48 (78228.22)	2247.54 (85810.67)	4397.02 (81928.65)
	1991	2187.73 (78629.94)	2285.98 (86264.10)	4473.71 (82354.04)
	1992	2234.07 (79060.13)	2327.40 (86585.31)	4561.47 (82728.68)
	1993	2286.22 (79507.46)	2369.91 (86813.56)	4656.13 (83065.62)
	1994	2341.58 (79949.71)	2412.25 (86975.21)	4753.83 (83366.78)
	1995	2397.83 (80329.94)	2453.59 (87046.91)	4851.42 (83592.20)
	1996	2457.22 (80677.58)	2488.36 (86770.75)	4945.58 (83632.46)
	1997	2518.20 (81028.61)	2511.60 (86043.19)	5029.80 (83457.35)
	1998	2575.27 (81324.76)	2525.64 (85073.91)	5100.90 (83138.88)
	1999	2623.70 (81557.30)	2533.72 (84118.69)	5157.42 (82795.86)
	2000	2658.35 (81736.92)	2538.86 (83451.83)	5197.21 (82565.76)
	2001	2675.18 (81949.09)	2539.29 (83232.01)	5214.47 (82568.86)
	2002	2676.81 (82181.02)	2532.84 (83279.06)	5209.64 (82711.23)
	2003	2667.17 (82405.41)	2520.73 (83471.79)	5187.90 (82920.12)
	2004	2650.39 (82550.40)	2504.60 (83653.11)	5155.00 (83082.51)
	2005	2629.54 (82562.63)	2484.63 (83676.34)	5114.17 (83099.98)

2006	2590.01 (81802.76)	2449.84 (82991.84)	5039.85 (82376.48)
2007	2527.94 (80121.93)	2398.93 (81533.84)	4926.87 (80803.24)
2008	2461.92 (78169.12)	2346.07 (79851.03)	4807.99 (78980.87)
2009	2411.92 (76618.98)	2306.65 (78510.93)	4718.56 (77532.33)
2010	2400.48 (76162.40)	2298.68 (78101.19)	4699.16 (77098.62)
2011	2417.88 (76507.54)	2312.51 (78308.94)	4730.38 (77377.70)
2012	2438.80 (76890.91)	2326.61 (78451.04)	4765.40 (77644.78)
2013	2464.02 (77318.40)	2343.17 (78584.63)	4807.19 (77930.46)
2014	2493.34 (77796.80)	2363.52 (78770.93)	4856.86 (78267.82)
2015	2525.76 (78344.91)	2388.28 (79083.86)	4914.04 (78702.31)
2016	2586.69 (79724.08)	2441.11 (80280.31)	5027.80 (79993.17)
2017	2655.13 (81247.50)	2502.12 (81667.69)	5157.26 (81450.82)
2018	2704.12 (82150.57)	2545.57 (82458.46)	5249.69 (82299.58)
2019	2748.44 (82950.55)	2585.97 (83189.71)	5334.41 (83066.32)
2020	2836.29 (84376.77)	2652.32 (84360.65)	5488.60 (84368.98)
2021	2712.80 (80124.53)	2528.79 (79841.91)	5241.59 (79987.93)
2022	2860.87 (83952.95)	2681.52 (84114.44)	5542.39 (84031.01)
2023	2756.76 (80438.50)	2590.75 (80802.34)	5347.51 (80614.37)
2024	2866.46 (83221.38)	2691.61 (83512.32)	5558.07 (83362.02)
2025	2770.77 (80103.93)	2601.65 (80367.23)	5372.42 (80231.22)
2026	2910.76 (83871.55)	2726.75 (83955.79)	5637.52 (83912.28)
2027	2775.23 (79686.38)	2609.91 (80113.71)	5385.13 (79892.91)
2028	2760.95 (79357.05)	2594.87 (79740.39)	5355.82 (79542.32)
2029	2756.98 (79422.98)	2589.59 (79761.34)	5346.57 (79586.50)
2030	2839.00 (82040.28)	2658.22 (82117.50)	5497.22 (82077.60)

	2031	2808.20 (81448.26)	2633.68 (81619.40)	5441.88 (81531.00)
	2032	2794.08 (81440.17)	2624.23 (81652.65)	5418.31 (81542.94)
	2033	2800.37 (81772.69)	2620.77 (81650.18)	5421.13 (81713.42)
	2034	2798.56 (81928.25)	2631.31 (82147.40)	5429.87 (82034.31)
	2035	2787.79 (81889.04)	2618.92 (82000.43)	5406.71 (81942.96)
	2036	2776.13 (81875.12)	2595.86 (81571.12)	5372.00 (81727.94)
	2037	2756.12 (81653.90)	2575.27 (81258.17)	5331.39 (81462.27)
	2038	2752.29 (81940.80)	2575.51 (81633.19)	5327.79 (81791.81)
	2039	2791.98 (83542.11)	2630.54 (83767.82)	5422.52 (83651.45)
	2040	2710.23 (81500.18)	2538.12 (81200.29)	5248.35 (81354.88)
**15-19 years**				
	1990	1593.77 (60352.93)	1656.81 (64838.51)	3250.58 (62558.83)
	1991	1602.09 (60862.13)	1662.17 (65132.36)	3264.26 (62964.16)
	1992	1614.78 (61456.57)	1670.99 (65529.07)	3285.78 (63462.34)
	1993	1633.15 (62092.08)	1685.00 (65998.37)	3318.16 (64016.17)
	1994	1658.21 (62724.27)	1705.53 (66491.43)	3363.73 (64579.42)
	1995	1689.76 (63311.68)	1732.22 (66950.10)	3421.97 (65102.64)
	1996	1737.89 (64214.80)	1769.70 (67517.30)	3507.58 (65839.62)
	1997	1807.03 (65631.07)	1818.95 (68320.74)	3625.99 (66953.32)
	1998	1886.83 (67228.08)	1874.01 (69209.98)	3760.84 (68201.26)
	1999	1965.08 (68637.27)	1928.42 (70005.44)	3893.50 (69308.16)
	2000	2029.14 (69425.06)	1976.61 (70484.25)	4005.75 (69943.70)
	2001	2072.09 (69327.60)	2009.42 (70247.58)	4081.51 (69777.50)
	2002	2095.40 (68548.24)	2022.54 (69256.06)	4117.93 (68894.07)
	2003	2101.12 (67323.30)	2019.62 (67801.54)	4120.74 (67556.84)

2004	2092.99 (65920.89)	2005.53 (66204.66)	4098.52 (66059.44)
2005	2074.80 (64584.88)	1984.95 (64762.93)	4059.75 (64671.82)
2006	2033.55 (63013.64)	1945.28 (63204.45)	3978.83 (63106.78)
2007	1966.78 (61023.18)	1882.69 (61297.40)	3849.48 (61156.98)
2008	1893.44 (59095.13)	1814.17 (59462.23)	3707.61 (59274.19)
2009	1833.63 (57706.57)	1758.39 (58141.79)	3592.02 (57918.80)
2010	1808.04 (57379.44)	1733.90 (57834.55)	3541.94 (57601.33)
2011	1816.31 (57997.36)	1742.02 (58489.56)	3558.32 (58237.28)
2012	1839.88 (58971.53)	1767.05 (59576.05)	3606.93 (59266.15)
2013	1872.62 (60125.65)	1801.99 (60878.71)	3674.61 (60492.60)
2014	1907.30 (61248.66)	1838.39 (62133.38)	3745.68 (61679.71)
2015	1937.94 (62130.45)	1868.88 (63072.95)	3806.81 (62589.61)
2016	1980.75 (63302.87)	1910.01 (64268.00)	3890.75 (63773.01)
2017	2024.45 (64432.96)	1952.49 (65435.85)	3976.94 (64921.46)
2018	2055.76 (65087.90)	1981.81 (66077.15)	4037.57 (65569.74)
2019	2087.12 (65677.86)	2010.12 (66613.33)	4097.24 (66133.50)
2020	2102.37 (65154.06)	1989.10 (65503.78)	4091.47 (65323.61)
2021	2114.37 (65057.80)	1997.27 (65443.27)	4111.65 (65244.47)
2022	2136.78 (65268.20)	2015.44 (65667.49)	4152.22 (65461.40)
2023	2149.96 (65174.89)	2035.98 (65903.57)	4185.94 (65527.29)
2024	2160.67 (64991.68)	2043.52 (65658.48)	4204.19 (65314.09)
2025	2206.30 (65856.82)	2092.55 (66718.03)	4298.86 (66273.23)
2026	2217.55 (65713.89)	2092.46 (66222.22)	4310.01 (65959.70)
2027	2225.82 (65529.96)	2107.22 (66253.06)	4333.04 (65879.63)
2028	2232.03 (65336.10)	2118.39 (66220.24)	4350.42 (65763.66)

	2029	2227.15 (64863.79)	2127.00 (66141.22)	4354.15 (65481.59)
	2030	2322.67 (67356.91)	2206.30 (68303.09)	4528.97 (67814.54)
	2031	2307.83 (66700.54)	2198.38 (67831.86)	4506.21 (67247.71)
	2032	2320.24 (66821.45)	2212.10 (68044.56)	4532.34 (67412.87)
	2033	2299.66 (66293.41)	2201.33 (67785.81)	4500.99 (67015.01)
	2034	2308.41 (66693.88)	2195.29 (67753.12)	4503.70 (67206.03)
	2035	2299.02 (66626.97)	2189.99 (67787.05)	4489.01 (67187.92)
	2036	2303.99 (67013.56)	2189.51 (67986.80)	4493.50 (67484.28)
	2037	2304.92 (67370.94)	2197.33 (68501.49)	4502.24 (67918.01)
	2038	2299.31 (67327.58)	2195.56 (68533.00)	4494.87 (67911.03)
	2039	2336.61 (68591.84)	2238.26 (70007.73)	4574.86 (69277.34)
	2040	2257.79 (66500.41)	2158.15 (67698.52)	4415.95 (67080.60)
**20-24 years**				
	1990	1346.77 (54237.83)	1413.67 (57850.33)	2760.44 (56029.63)
	1991	1364.02 (54277.57)	1418.17 (57551.90)	2782.20 (55898.65)
	1992	1379.46 (54446.51)	1424.86 (57455.26)	2804.32 (55934.79)
	1993	1392.19 (54708.13)	1433.06 (57492.54)	2825.26 (56085.92)
	1994	1403.24 (55078.04)	1443.76 (57687.92)	2847.00 (56371.35)
	1995	1414.48 (55577.05)	1457.39 (58057.39)	2871.87 (56808.68)
	1996	1437.68 (56615.33)	1481.27 (58922.41)	2918.95 (57763.06)
	1997	1478.70 (58301.81)	1519.66 (60341.79)	2998.37 (59318.19)
	1998	1529.46 (60219.62)	1566.08 (61977.79)	3095.54 (61096.45)
	1999	1580.90 (61924.09)	1613.02 (63449.43)	3193.92 (62685.15)
	2000	1622.47 (62955.08)	1652.05 (64366.49)	3274.52 (63659.34)
	2001	1647.66 (63068.39)	1674.24 (64349.91)	3321.90 (63707.84)

2002	1660.85 (62495.08)	1680.05 (63526.12)	3340.91 (63009.35)
2003	1664.57 (61438.60)	1674.04 (62186.42)	3338.61 (61811.31)
2004	1661.16 (60101.86)	1661.03 (60606.52)	3322.18 (60353.12)
2005	1656.17 (58700.25)	1648.98 (59061.48)	3305.14 (58879.92)
2006	1643.34 (56918.66)	1631.67 (57230.84)	3275.02 (57073.76)
2007	1616.78 (54655.41)	1602.06 (54952.56)	3218.85 (54802.90)
2008	1586.78 (52445.32)	1569.77 (52733.63)	3156.55 (52588.30)
2009	1564.56 (50786.63)	1545.98 (51053.91)	3110.54 (50919.12)
2010	1562.34 (50117.64)	1542.97 (50347.72)	3105.31 (50231.70)
2011	1580.52 (50463.46)	1561.73 (50737.66)	3142.25 (50599.37)
2012	1606.16 (51350.49)	1591.41 (51817.35)	3197.57 (51581.78)
2013	1634.77 (52553.95)	1625.45 (53286.94)	3260.22 (52916.86)
2014	1661.82 (53820.96)	1657.10 (54809.65)	3318.91 (54310.10)
2015	1681.67 (54868.75)	1677.61 (55990.64)	3359.28 (55423.34)
2016	1709.90 (56071.98)	1704.38 (57269.02)	3414.28 (56663.21)
2017	1741.17 (57239.43)	1735.48 (58550.00)	3476.64 (57886.23)
2018	1763.68 (58008.13)	1755.94 (59353.13)	3519.61 (58671.44)
2019	1786.05 (58680.43)	1773.52 (59961.51)	3559.56 (59311.80)
2020	1804.24 (58178.65)	1765.17 (59233.77)	3569.41 (58695.70)
2021	1814.05 (58110.18)	1770.21 (59313.73)	3584.25 (58698.43)
2022	1858.62 (59141.60)	1779.93 (59498.47)	3638.55 (59315.64)
2023	1864.98 (58952.22)	1792.44 (59730.08)	3657.42 (59330.89)
2024	1869.49 (58691.82)	1781.50 (59141.03)	3650.98 (58910.16)
2025	1922.79 (59941.45)	1822.64 (60238.13)	3745.43 (60085.45)
2026	1926.63 (59628.48)	1815.35 (59692.35)	3741.98 (59659.44)

	2027	1945.80 (59779.34)	1837.64 (60081.79)	3783.44 (59925.86)
	2028	1947.19 (59366.24)	1835.96 (59630.68)	3783.15 (59494.28)
	2029	1964.44 (59423.18)	1862.64 (60045.86)	3827.08 (59724.62)
	2030	2057.60 (61760.27)	1917.66 (61341.04)	3975.26 (61557.32)
	2031	2063.78 (61493.17)	1939.67 (61582.75)	4003.45 (61536.54)
	2032	2079.71 (61560.04)	1955.49 (61674.85)	4035.20 (61615.62)
	2033	2075.47 (61078.04)	1964.81 (61607.84)	4040.28 (61334.54)
	2034	2103.07 (61573.22)	1984.91 (61908.39)	4087.98 (61735.51)
	2035	2103.53 (61319.34)	1991.70 (61841.56)	4095.23 (61572.21)
	2036	2118.87 (61553.89)	2014.12 (62326.28)	4132.99 (61927.89)
	2037	2127.13 (61570.22)	2027.40 (62540.05)	4154.53 (62039.71)
	2038	2116.24 (61311.18)	2019.04 (62345.85)	4135.28 (61812.03)
	2039	2152.70 (62502.83)	2061.83 (63808.45)	4214.52 (63134.82)
	2040	2086.88 (60774.88)	1983.09 (61548.53)	4069.97 (61149.39)
**25-29 years**				
	1990	1291.18 (57969.46)	1357.83 (61689.10)	2649.01 (59818.26)
	1991	1318.56 (57474.77)	1375.68 (60758.72)	2694.24 (59105.95)
	1992	1344.74 (57153.17)	1393.66 (60128.89)	2738.40 (58629.86)
	1993	1368.77 (57083.74)	1411.12 (59865.13)	2779.89 (58462.54)
	1994	1391.04 (57201.55)	1429.00 (59873.39)	2820.04 (58524.96)
	1995	1412.82 (57447.96)	1448.45 (60073.21)	2861.27 (58747.61)
	1996	1441.69 (57988.11)	1474.39 (60521.96)	2916.08 (59242.15)
	1997	1478.53 (59007.05)	1507.47 (61317.73)	2986.00 (60151.40)
	1998	1515.75 (60224.30)	1542.17 (62233.84)	3057.92 (61221.26)
	1999	1547.98 (61412.00)	1575.01 (63139.34)	3122.99 (62271.17)

2000	1570.53 (62339.04)	1602.52 (63918.49)	3173.05 (63126.85)
2001	1577.56 (62738.89)	1613.74 (64207.94)	3191.30 (63473.24)
2002	1571.57 (62559.55)	1606.73 (63802.82)	3178.30 (63181.94)
2003	1559.07 (61953.87)	1589.61 (62923.82)	3148.67 (62439.78)
2004	1545.52 (61085.15)	1569.81 (61789.36)	3115.32 (61437.98)
2005	1536.01 (60151.95)	1554.78 (60657.58)	3090.79 (60405.24)
2006	1524.87 (58916.04)	1539.27 (59274.13)	3064.14 (59095.39)
2007	1508.11 (57271.45)	1517.60 (57505.46)	3025.71 (57388.59)
2008	1492.99 (55635.87)	1497.77 (55778.87)	2990.76 (55707.39)
2009	1487.68 (54405.56)	1488.30 (54485.63)	2975.97 (54445.58)
2010	1504.45 (53944.93)	1501.04 (53984.13)	3005.49 (53964.50)
2011	1547.64 (54273.46)	1542.82 (54382.48)	3090.46 (54327.83)
2012	1604.99 (55005.17)	1602.93 (55319.13)	3207.92 (55161.61)
2013	1666.51 (55901.13)	1669.60 (56456.82)	3336.12 (56177.85)
2014	1723.17 (56777.47)	1731.36 (57529.45)	3454.53 (57151.88)
2015	1765.96 (57460.17)	1776.00 (58291.28)	3541.95 (57873.92)
2016	1808.92 (58552.02)	1815.57 (59324.22)	3624.49 (58936.30)
2017	1844.40 (59763.23)	1847.85 (60508.62)	3692.25 (60133.96)
2018	1862.48 (60676.24)	1862.83 (61412.69)	3725.31 (61042.28)
2019	1879.63 (61672.46)	1874.73 (62346.87)	3754.36 (62007.39)
2020	1853.17 (60654.10)	1841.42 (61197.30)	3694.59 (60923.63)
2021	1842.17 (60584.36)	1828.36 (61260.09)	3670.53 (60919.08)
2022	1840.80 (60609.27)	1830.04 (61604.10)	3670.84 (61101.17)
2023	1847.42 (60665.50)	1831.41 (61741.38)	3678.82 (61196.37)
2024	1846.10 (60342.99)	1815.15 (61217.52)	3661.25 (60773.42)

	2025	1886.46 (61296.76)	1842.41 (62098.83)	3728.88 (61690.45)
	2026	1898.41 (61276.31)	1830.91 (61615.11)	3729.32 (61442.18)
	2027	1913.11 (61336.60)	1846.43 (61987.37)	3759.55 (61654.50)
	2028	1930.38 (61477.93)	1835.44 (61423.05)	3765.81 (61451.17)
	2029	1940.30 (61369.10)	1846.39 (61552.00)	3786.69 (61458.15)
	2030	2015.07 (63282.33)	1882.27 (62465.57)	3897.34 (62885.21)
	2031	2026.91 (63191.22)	1883.07 (62170.56)	3909.99 (62695.51)
	2032	2041.97 (63188.09)	1898.18 (62308.95)	3940.16 (62761.48)
	2033	2048.18 (62892.21)	1911.76 (62336.17)	3959.94 (62622.53)
	2034	2078.98 (63332.86)	1929.48 (62440.18)	4008.46 (62900.00)
	2035	2102.92 (63561.69)	1951.13 (62647.65)	4054.05 (63118.47)
	2036	2133.75 (64016.78)	2000.68 (63755.78)	4134.43 (63890.21)
	2037	2143.38 (63877.21)	2010.36 (63637.10)	4153.75 (63760.78)
	2038	2151.66 (63746.26)	2016.59 (63458.19)	4168.24 (63606.57)
	2039	2192.45 (64616.49)	2082.22 (65172.28)	4274.66 (64886.03)
	2040	2151.11 (63117.39)	2026.29 (63133.38)	4177.40 (63125.14)
**30-34 years**				
	1990	1207.76 (61788.93)	1251.46 (65809.82)	2459.22 (63771.73)
	1991	1230.20 (62086.60)	1268.75 (65708.46)	2498.95 (63874.14)
	1992	1256.73 (62129.27)	1290.58 (65354.04)	2547.31 (63722.29)
	1993	1289.26 (61870.99)	1318.88 (64731.54)	2608.13 (63285.19)
	1994	1324.99 (61452.49)	1351.27 (64029.97)	2676.26 (62727.41)
	1995	1359.53 (61008.68)	1383.75 (63385.70)	2743.28 (62184.97)
	1996	1394.96 (60876.53)	1416.29 (63010.71)	2811.25 (61933.33)
	1997	1433.51 (61128.62)	1449.75 (62952.17)	2883.26 (62032.13)

1998	1471.18 (61704.21)	1481.93 (63230.74)	2953.11 (62460.93)
1999	1505.62 (62390.08)	1512.33 (63696.84)	3017.94 (63038.14)
2000	1534.87 (62958.05)	1541.08 (64218.88)	3075.95 (63583.49)
2001	1552.46 (62975.43)	1558.43 (64241.29)	3110.89 (63603.28)
2002	1554.64 (62496.22)	1557.50 (63595.08)	3112.14 (63041.36)
2003	1544.73 (61711.66)	1543.56 (62519.27)	3088.29 (62112.69)
2004	1528.83 (60856.23)	1524.46 (61351.30)	3053.28 (61102.41)
2005	1513.84 (60173.04)	1508.30 (60435.23)	3022.14 (60303.61)
2006	1495.47 (59436.16)	1489.10 (59563.59)	2984.56 (59499.67)
2007	1471.20 (58400.88)	1462.77 (58430.57)	2933.97 (58415.68)
2008	1449.94 (57361.92)	1438.70 (57330.26)	2888.64 (57346.14)
2009	1439.99 (56629.73)	1426.10 (56556.18)	2866.09 (56593.11)
2010	1450.63 (56534.51)	1434.67 (56422.77)	2885.30 (56478.89)
2011	1485.49 (57174.47)	1470.55 (57110.94)	2956.04 (57142.84)
2012	1537.42 (58269.08)	1527.13 (58401.32)	3064.55 (58334.90)
2013	1599.38 (59571.84)	1594.83 (59968.00)	3194.21 (59768.98)
2014	1662.89 (60810.11)	1662.41 (61440.18)	3325.30 (61123.47)
2015	1720.10 (61676.90)	1719.51 (62409.93)	3439.61 (62041.19)
2016	1790.59 (62804.12)	1782.34 (63384.07)	3572.93 (63092.10)
2017	1863.23 (63892.23)	1847.08 (64292.86)	3710.32 (64091.04)
2018	1919.09 (64432.84)	1898.24 (64722.00)	3817.32 (64576.30)
2019	1970.44 (64993.70)	1944.05 (65114.63)	3914.49 (65053.70)
2020	1948.37 (64043.04)	1934.45 (64046.81)	3882.81 (64044.92)
2021	1947.99 (63694.10)	1947.01 (64024.31)	3895.00 (63858.74)
2022	1963.49 (64042.39)	1958.94 (64239.42)	3922.42 (64140.64)

	2023	1958.22 (63965.53)	1957.86 (64371.52)	3916.08 (64167.86)
	2024	1940.80 (63707.60)	1927.67 (63825.32)	3868.47 (63766.20)
	2025	1955.38 (64572.23)	1932.25 (64547.37)	3887.63 (64559.87)
	2026	1942.66 (64457.35)	1910.69 (64347.04)	3853.35 (64402.61)
	2027	1938.90 (64403.23)	1910.35 (64634.98)	3849.26 (64518.04)
	2028	1929.74 (63924.54)	1894.81 (64200.87)	3824.55 (64061.15)
	2029	1940.06 (63966.18)	1901.83 (64461.14)	3841.89 (64210.25)
	2030	2000.75 (65572.01)	1926.17 (65242.79)	3926.93 (65410.11)
	2031	2007.15 (65341.43)	1915.74 (64785.11)	3922.89 (65068.56)
	2032	2028.73 (65595.94)	1920.97 (64801.51)	3949.70 (65207.14)
	2033	2032.79 (65284.66)	1928.68 (64851.55)	3961.46 (65073.08)
	2034	2066.31 (65899.88)	1929.21 (64616.46)	3995.52 (65273.88)
	2035	2080.00 (65861.36)	1939.18 (64653.57)	4019.17 (65273.04)
	2036	2108.18 (66262.66)	1957.18 (64914.04)	4065.36 (65606.47)
	2037	2128.64 (66403.54)	1968.55 (64911.34)	4097.19 (65678.12)
	2038	2136.19 (66120.11)	1977.63 (64771.79)	4113.82 (65465.00)
	2039	2183.14 (67032.38)	2032.92 (66076.77)	4216.06 (66568.17)
	2040	2159.62 (65785.92)	1995.48 (64348.96)	4155.09 (65087.90)
**35-39 years**				
	1990	1147.43 (64100.46)	1180.78 (67963.12)	2328.20 (66002.95)
	1991	1172.12 (63896.67)	1194.57 (67212.97)	2366.69 (65528.60)
	1992	1196.59 (63915.47)	1209.56 (66785.07)	2406.16 (65326.51)
	1993	1219.20 (64211.95)	1224.77 (66764.80)	2443.97 (65466.40)
	1994	1240.73 (64711.33)	1241.55 (67028.68)	2482.28 (65850.01)
	1995	1263.11 (65251.38)	1261.41 (67381.50)	2524.52 (66298.62)

1996	1290.43 (65713.57)	1286.39 (67582.18)	2576.82 (66633.31)
1997	1323.99 (65971.70)	1315.63 (67461.92)	2639.62 (66706.12)
1998	1363.82 (65897.53)	1350.19 (66978.22)	2714.01 (66430.77)
1999	1405.39 (65583.87)	1387.12 (66325.03)	2792.51 (65949.94)
2000	1443.36 (65147.66)	1422.77 (65676.26)	2866.12 (65408.99)
2001	1469.17 (64511.72)	1447.31 (64851.53)	2916.49 (64679.90)
2002	1480.58 (63555.25)	1455.93 (63670.22)	2936.51 (63612.20)
2003	1481.01 (62541.34)	1452.64 (62433.76)	2933.65 (62488.02)
2004	1476.24 (61572.90)	1443.85 (61275.42)	2920.09 (61425.45)
2005	1473.09 (60784.42)	1437.32 (60377.59)	2910.41 (60582.82)
2006	1468.73 (59880.84)	1430.42 (59463.34)	2899.16 (59674.12)
2007	1457.53 (58818.75)	1417.67 (58394.64)	2875.20 (58608.87)
2008	1444.22 (57855.18)	1404.44 (57410.57)	2848.66 (57635.12)
2009	1436.21 (57286.06)	1398.05 (56814.95)	2834.26 (57052.70)
2010	1442.41 (57409.08)	1406.18 (56910.02)	2848.59 (57161.63)
2011	1466.73 (58351.07)	1433.50 (57926.34)	2900.23 (58140.37)
2012	1504.43 (59808.73)	1476.76 (59607.95)	2981.19 (59709.11)
2013	1550.99 (61494.88)	1529.72 (61592.53)	3080.70 (61543.33)
2014	1600.66 (63103.36)	1584.80 (63472.79)	3185.46 (63286.62)
2015	1646.40 (64318.04)	1633.03 (64824.90)	3279.43 (64569.44)
2016	1708.86 (65938.41)	1689.57 (66204.75)	3398.43 (66070.56)
2017	1778.81 (67611.83)	1752.60 (67601.58)	3531.41 (67606.74)
2018	1838.63 (68701.78)	1808.85 (68578.84)	3647.48 (68640.76)
2019	1901.09 (69754.57)	1864.98 (69475.79)	3766.07 (69616.24)
2020	1921.00 (68354.72)	1873.50 (67438.00)	3794.50 (67899.00)

	2021	1943.23 (67737.28)	1904.72 (67085.55)	3847.94 (67413.10)
	2022	1977.24 (67794.09)	1936.02 (66979.72)	3913.26 (67388.73)
	2023	1993.38 (67487.41)	1961.60 (66881.83)	3954.99 (67185.69)
	2024	2002.08 (67078.73)	1962.03 (66044.98)	3964.11 (66563.07)
	2025	2033.96 (67602.75)	1997.76 (66562.18)	4031.72 (67083.10)
	2026	2041.58 (67490.36)	1996.42 (66058.53)	4038.00 (66774.78)
	2027	2048.82 (67555.43)	2012.21 (66392.93)	4061.04 (66974.38)
	2028	2031.00 (67061.75)	1994.51 (65976.95)	4025.51 (66519.84)
	2029	2024.73 (67178.44)	1985.33 (66133.78)	4010.06 (66657.15)
	2030	2057.41 (68669.57)	1988.33 (66822.60)	4045.75 (67749.26)
	2031	2045.32 (68586.52)	1967.37 (66654.73)	4012.69 (67625.59)
	2032	2047.71 (68737.21)	1967.61 (66970.43)	4015.32 (67859.95)
	2033	2048.69 (68578.02)	1970.61 (67165.56)	4019.31 (67878.16)
	2034	2056.01 (68496.11)	1960.03 (66824.75)	4016.04 (67670.08)
	2035	2065.21 (68385.13)	1963.74 (66903.37)	4028.95 (67654.79)
	2036	2089.77 (68729.66)	1971.11 (67043.03)	4060.88 (67900.52)
	2037	2101.07 (68626.83)	1971.17 (66875.30)	4072.24 (67767.69)
	2038	2111.49 (68496.55)	1969.66 (66604.71)	4081.15 (67570.27)
	2039	2145.31 (69103.09)	2009.47 (67681.54)	4154.78 (68408.17)
	2040	2127.85 (68042.20)	1965.46 (65892.84)	4093.31 (66992.92)
**40-44 years**				
	1990	980.35 (67062.69)	991.18 (70744.00)	1971.53 (68864.28)
	1991	1028.43 (67088.58)	1036.05 (70420.61)	2064.48 (68720.38)
	1992	1064.08 (67200.98)	1067.41 (70056.84)	2131.49 (68601.43)
	1993	1100.61 (67178.60)	1098.23 (69582.01)	2198.84 (68357.88)

1994	1134.73 (67288.74)	1127.99 (69228.74)	2262.72 (68242.07)
1995	1178.63 (67185.43)	1163.73 (68707.89)	2342.35 (67933.29)
1996	1202.13 (66984.74)	1177.64 (68050.25)	2379.77 (67507.81)
1997	1224.91 (66974.07)	1191.50 (67628.85)	2416.41 (67295.34)
1998	1245.93 (67243.67)	1204.76 (67559.07)	2450.69 (67398.35)
1999	1266.33 (67735.93)	1218.59 (67719.89)	2484.92 (67728.06)
2000	1288.25 (68285.92)	1234.93 (67925.54)	2523.18 (68109.07)
2001	1307.78 (68349.88)	1251.05 (67670.70)	2558.83 (68016.13)
2002	1321.21 (67570.69)	1262.96 (66648.00)	2584.16 (67116.58)
2003	1333.52 (66132.10)	1274.55 (65030.32)	2608.08 (65589.04)
2004	1345.83 (64450.75)	1286.39 (63228.83)	2632.22 (63847.74)
2005	1358.79 (62925.01)	1298.84 (61603.98)	2657.64 (62272.39)
2006	1367.52 (61592.31)	1307.73 (60184.57)	2675.25 (60896.03)
2007	1369.73 (60278.66)	1311.02 (58854.38)	2680.75 (59573.61)
2008	1369.18 (59246.63)	1311.72 (57849.86)	2680.90 (58554.89)
2009	1372.66 (58641.11)	1315.91 (57292.15)	2688.57 (57973.02)
2010	1388.34 (58641.31)	1331.21 (57350.35)	2719.55 (58002.21)
2011	1421.35 (59281.70)	1364.96 (58165.77)	2786.30 (58729.73)
2012	1465.39 (60478.94)	1413.01 (59644.14)	2878.39 (60066.23)
2013	1512.87 (61964.64)	1466.95 (61421.35)	2979.82 (61695.98)
2014	1557.83 (63492.31)	1518.97 (63181.22)	3076.80 (63338.35)
2015	1594.71 (64814.90)	1561.04 (64620.49)	3155.76 (64718.59)
2016	1649.59 (66988.98)	1608.66 (66453.57)	3258.25 (66723.56)
2017	1708.75 (69322.24)	1658.90 (68412.36)	3367.65 (68871.03)
2018	1753.68 (70933.92)	1701.30 (69943.73)	3454.98 (70442.85)

	2019	1803.50 (72504.24)	1747.80 (71426.74)	3551.31 (71969.91)
	2020	1828.68 (71886.95)	1770.40 (70439.21)	3599.08 (71167.44)
	2021	1811.41 (70485.83)	1765.20 (69412.17)	3576.61 (69951.81)
	2022	1870.09 (71821.34)	1821.44 (70589.83)	3691.53 (71208.37)
	2023	1873.39 (70681.04)	1832.03 (69665.10)	3705.42 (70175.06)
	2024	1924.07 (71045.81)	1875.07 (69706.08)	3799.14 (70378.21)
	2025	1950.10 (70407.16)	1893.15 (68740.94)	3843.25 (69576.42)
	2026	2010.07 (71079.81)	1948.73 (69224.39)	3958.80 (70154.21)
	2027	2009.89 (69896.88)	1956.46 (68257.90)	3966.34 (69078.71)
	2028	2018.03 (69284.61)	1966.22 (67596.27)	3984.25 (68441.01)
	2029	2043.12 (69405.66)	1995.37 (67716.47)	4038.49 (68560.65)
	2030	2082.28 (70158.77)	2012.39 (67589.71)	4094.68 (68872.21)
	2031	2088.74 (69985.63)	2016.06 (67238.46)	4104.79 (68608.86)
	2032	2098.30 (70115.64)	2024.54 (67325.25)	4122.83 (68717.08)
	2033	2089.28 (69905.46)	2026.23 (67550.57)	4115.52 (68725.88)
	2034	2083.11 (70030.65)	2004.80 (67302.69)	4087.91 (68665.70)
	2035	2066.98 (69897.00)	1985.78 (67255.13)	4052.75 (68577.09)
	2036	2075.02 (70493.00)	1983.73 (67729.36)	4058.74 (69114.64)
	2037	2065.06 (70220.60)	1968.72 (67524.72)	4033.79 (68878.47)
	2038	2062.23 (69921.76)	1962.08 (67386.42)	4024.31 (68662.24)
	2039	2072.41 (69926.28)	1974.94 (67844.59)	4047.36 (68894.78)
	2040	2061.59 (69131.70)	1943.97 (66728.51)	4005.56 (67944.14)
**45-49 years**				
	1990	844.76 (71275.63)	849.43 (74584.06)	1694.19 (72896.89)
	1991	857.83 (71322.56)	856.13 (74095.77)	1713.96 (72681.35)

1992	884.57 (70924.58)	875.90 (73274.47)	1760.47 (72074.59)
1993	913.67 (70545.94)	899.71 (72372.20)	1813.39 (71440.37)
1994	948.38 (69815.95)	927.35 (71217.12)	1875.73 (70501.72)
1995	975.49 (69277.52)	952.70 (70392.83)	1928.19 (69824.13)
1996	1018.70 (69054.30)	994.87 (70012.72)	2013.58 (69524.53)
1997	1048.61 (68885.23)	1022.00 (69477.43)	2070.61 (69176.26)
1998	1078.98 (68552.69)	1046.93 (68719.09)	2125.91 (68634.54)
1999	1107.40 (68413.90)	1069.69 (68036.28)	2177.08 (68227.84)
2000	1146.87 (68147.44)	1098.19 (67198.75)	2245.06 (67680.06)
2001	1164.34 (67638.59)	1106.07 (66198.25)	2270.41 (66929.15)
2002	1174.86 (66969.84)	1111.11 (65263.14)	2285.97 (66129.27)
2003	1179.70 (66370.72)	1114.07 (64582.66)	2293.76 (65490.07)
2004	1181.99 (65890.88)	1116.97 (64099.59)	2298.96 (65008.23)
2005	1186.71 (65530.23)	1123.28 (63739.16)	2310.00 (64646.88)
2006	1194.27 (64981.93)	1133.63 (63194.58)	2327.90 (64099.08)
2007	1203.14 (63999.10)	1145.89 (62250.34)	2349.03 (63133.92)
2008	1218.16 (62765.14)	1163.75 (61058.72)	2381.91 (61919.67)
2009	1240.45 (61655.14)	1187.63 (59972.26)	2428.07 (60820.36)
2010	1270.61 (61004.47)	1217.82 (59289.52)	2488.43 (60152.96)
2011	1310.34 (61134.34)	1258.29 (59402.12)	2568.63 (60273.33)
2012	1358.19 (61867.00)	1309.40 (60259.25)	2667.59 (61067.25)
2013	1408.47 (63029.03)	1364.16 (61630.77)	2772.63 (62333.23)
2014	1457.46 (64322.18)	1417.09 (63155.67)	2874.55 (63741.78)
2015	1500.79 (65421.85)	1462.34 (64449.90)	2963.13 (64938.55)
2016	1570.49 (67543.57)	1519.03 (66185.79)	3089.52 (66869.10)

	2017	1645.36 (69979.28)	1578.16 (68083.83)	3223.52 (69038.30)
	2018	1695.94 (71540.32)	1624.34 (69480.93)	3320.27 (70517.79)
	2019	1743.88 (73157.89)	1670.60 (70959.90)	3414.48 (72065.72)
	2020	1744.33 (72377.20)	1671.61 (70087.98)	3415.94 (71238.57)
	2021	1706.53 (70508.00)	1641.57 (68437.13)	3348.11 (69477.22)
	2022	1757.48 (72163.66)	1692.58 (70040.09)	3450.05 (71105.99)
	2023	1737.64 (70826.59)	1683.29 (69063.68)	3420.93 (69948.03)
	2024	1784.38 (72198.95)	1717.47 (69853.20)	3501.85 (71029.12)
	2025	1777.57 (71323.36)	1718.02 (69190.88)	3495.58 (70259.10)
	2026	1826.37 (72524.37)	1760.44 (70064.51)	3586.80 (71295.83)
	2027	1818.08 (71239.66)	1765.50 (69243.56)	3583.59 (70242.07)
	2028	1841.09 (70853.64)	1782.10 (68569.29)	3623.19 (69711.35)
	2029	1883.38 (70917.36)	1829.67 (68811.74)	3713.05 (69863.91)
	2030	1941.69 (71470.07)	1861.21 (68356.86)	3802.90 (69911.75)
	2031	1973.18 (71118.16)	1890.18 (67903.34)	3863.36 (69508.11)
	2032	2006.53 (71107.22)	1920.67 (67756.21)	3927.21 (69427.91)
	2033	2023.17 (70766.10)	1950.34 (67787.50)	3973.51 (69272.08)
	2034	2039.58 (70570.05)	1961.02 (67272.47)	4000.60 (68914.19)
	2035	2049.82 (70329.37)	1973.58 (66995.50)	4023.40 (68653.55)
	2036	2069.43 (70593.02)	1988.64 (67026.25)	4058.07 (68798.91)
	2037	2062.80 (70164.72)	1980.80 (66562.66)	4043.60 (68352.76)
	2038	2056.96 (70048.07)	1971.41 (66409.95)	4028.37 (68219.14)
	2039	2049.74 (70125.96)	1971.23 (66865.66)	4020.97 (68488.84)
	2040	2018.66 (69460.39)	1934.10 (66186.53)	3952.76 (67818.97)
**50-54 years**				

1990	781.35 (72580.82)	789.67 (75250.92)	1571.02 (73898.82)
1991	792.85 (72870.35)	796.42 (75071.91)	1589.28 (73957.22)
1992	800.28 (73084.29)	799.91 (74914.79)	1600.19 (73988.01)
1993	804.15 (73094.75)	798.80 (74590.06)	1602.95 (73832.34)
1994	807.27 (73032.00)	797.80 (74140.59)	1605.07 (73578.85)
1995	810.73 (72991.09)	796.90 (73771.76)	1607.62 (73375.99)
1996	818.68 (72628.43)	801.03 (73064.29)	1619.71 (72843.33)
1997	837.93 (71594.27)	814.68 (71699.61)	1652.61 (71646.16)
1998	858.50 (70548.60)	830.77 (70157.21)	1689.27 (70355.57)
1999	885.13 (69231.65)	850.74 (68413.88)	1735.86 (68828.44)
2000	906.51 (68324.71)	870.37 (67180.27)	1776.88 (67759.30)
2001	942.79 (67777.08)	905.20 (66430.84)	1848.00 (67110.90)
2002	964.70 (67179.83)	926.36 (65578.67)	1891.07 (66385.83)
2003	985.45 (66339.74)	946.40 (64582.24)	1931.85 (65466.96)
2004	1003.63 (65670.97)	965.50 (63740.02)	1969.13 (64709.79)
2005	1032.33 (64924.25)	991.51 (62859.64)	2023.84 (63896.09)
2006	1045.17 (64200.56)	1003.01 (62077.90)	2048.18 (63143.24)
2007	1056.24 (63599.33)	1014.84 (61536.18)	2071.08 (62571.36)
2008	1066.08 (63300.68)	1027.46 (61401.74)	2093.55 (62354.27)
2009	1077.64 (63343.62)	1042.76 (61616.51)	2120.40 (62482.34)
2010	1096.18 (63749.03)	1064.56 (62129.98)	2160.75 (62940.94)
2011	1128.16 (64561.40)	1100.48 (63035.82)	2228.64 (63798.96)
2012	1173.55 (65570.16)	1150.67 (64187.56)	2324.22 (64878.30)
2013	1229.33 (66436.17)	1211.10 (65203.86)	2440.44 (65818.85)
2014	1287.77 (67031.33)	1272.96 (65917.99)	2560.73 (66473.22)

2015	1339.77 (67269.72)	1326.11 (66172.11)	2665.88 (66719.22)
2016	1416.56 (69034.50)	1387.76 (67126.40)	2804.31 (68076.88)
2017	1501.60 (71369.03)	1453.30 (68503.92)	2954.91 (69930.55)
2018	1560.35 (72780.96)	1504.04 (69573.25)	3064.40 (71170.44)
2019	1616.58 (74295.95)	1554.80 (70920.67)	3171.38 (72601.95)
2020	1648.39 (73658.60)	1585.06 (70671.51)	3233.45 (72163.39)
2021	1586.51 (69926.57)	1527.75 (67316.35)	3114.25 (68621.26)
2022	1672.20 (72839.20)	1610.73 (70210.43)	3282.93 (71525.27)
2023	1627.94 (70292.61)	1567.30 (67713.13)	3195.24 (69003.24)
2024	1706.45 (73273.75)	1638.21 (70323.63)	3344.66 (71798.48)
2025	1652.93 (70680.56)	1583.16 (67594.24)	3236.09 (69136.23)
2026	1726.34 (73489.71)	1656.13 (70302.29)	3382.47 (71893.75)
2027	1667.87 (70545.86)	1607.30 (67718.30)	3275.17 (69129.31)
2028	1679.06 (70483.27)	1614.08 (67420.34)	3293.14 (68948.00)
2029	1694.93 (70612.46)	1642.45 (68002.86)	3337.38 (69303.61)
2030	1732.62 (71563.32)	1657.23 (67936.12)	3389.86 (69742.89)
2031	1747.61 (71417.49)	1674.03 (67808.48)	3421.64 (69605.01)
2032	1777.77 (71667.18)	1704.52 (68028.24)	3482.28 (69838.58)
2033	1805.12 (71448.52)	1735.50 (67938.65)	3540.62 (69683.90)
2034	1846.68 (71493.23)	1768.94 (67670.93)	3615.62 (69570.68)
2035	1872.98 (70858.90)	1795.22 (67051.40)	3668.20 (68942.94)
2036	1922.05 (71181.48)	1841.42 (67259.52)	3763.47 (69206.96)
2037	1928.62 (70207.57)	1848.64 (66295.86)	3777.26 (68237.07)
2038	1943.90 (69825.98)	1862.19 (65785.37)	3806.09 (67788.84)
2039	1963.02 (69730.65)	1900.53 (66255.00)	3863.55 (67976.51)

	2040	1958.14 (68952.57)	1889.53 (65172.13)	3847.67 (67042.77)
**55-59 years**				
	1990	688.91 (74041.56)	701.32 (75922.86)	1390.22 (74978.81)
	1991	699.65 (74218.63)	710.85 (75873.58)	1410.49 (75043.55)
	1992	711.20 (74270.08)	723.11 (75757.23)	1434.31 (75012.46)
	1993	722.10 (74265.41)	735.01 (75679.87)	1457.11 (74972.24)
	1994	732.17 (74303.98)	746.99 (75757.86)	1479.16 (75031.16)
	1995	741.16 (74335.10)	758.09 (75747.96)	1499.25 (75042.86)
	1996	746.35 (74188.45)	762.48 (75400.52)	1508.83 (74796.05)
	1997	746.48 (73804.85)	759.76 (74695.29)	1506.24 (74251.32)
	1998	743.22 (73128.38)	750.24 (73563.60)	1493.46 (73346.37)
	1999	739.97 (72392.54)	740.13 (72208.30)	1480.10 (72300.29)
	2000	738.79 (71816.03)	731.26 (71008.98)	1470.05 (71412.29)
	2001	743.81 (71087.41)	730.29 (69753.37)	1474.09 (70420.19)
	2002	760.33 (69770.18)	741.87 (68168.29)	1502.20 (68969.78)
	2003	778.60 (68528.48)	758.29 (66660.02)	1536.89 (67593.68)
	2004	802.88 (67074.64)	780.31 (65131.47)	1583.19 (66102.62)
	2005	822.37 (66076.21)	803.34 (64201.67)	1625.71 (65136.42)
	2006	854.89 (65417.13)	839.66 (63674.06)	1694.55 (64541.66)
	2007	876.58 (64912.79)	865.10 (63196.78)	1741.68 (64048.94)
	2008	899.91 (64360.06)	892.01 (62737.67)	1791.92 (63542.08)
	2009	923.82 (64162.66)	920.61 (62569.25)	1844.43 (63357.32)
	2010	961.05 (64072.80)	958.62 (62488.17)	1919.67 (63271.57)
	2011	991.45 (64497.22)	990.73 (62994.11)	1982.18 (63737.07)
	2012	1029.20 (65569.06)	1032.38 (64269.16)	2061.58 (64911.60)

2013	1069.69 (67130.48)	1078.37 (66128.67)	2148.06 (66623.79)
2014	1109.52 (68848.61)	1124.02 (68127.75)	2233.54 (68483.94)
2015	1145.81 (70278.25)	1165.06 (69735.25)	2310.87 (70003.43)
2016	1207.38 (72824.31)	1217.71 (71540.02)	2425.08 (72173.72)
2017	1280.51 (75359.33)	1276.96 (73072.94)	2557.46 (74200.11)
2018	1344.89 (76482.42)	1333.94 (73679.26)	2678.83 (75060.40)
2019	1415.04 (77425.14)	1396.08 (74160.86)	2811.12 (75768.85)
2020	1459.59 (77146.32)	1447.14 (74361.30)	2906.73 (75734.17)
2021	1440.36 (73505.41)	1418.84 (70432.66)	2859.20 (71947.80)
2022	1536.38 (76127.99)	1508.56 (72777.11)	3044.94 (74430.15)
2023	1517.62 (73456.27)	1478.58 (69795.50)	2996.20 (71602.95)
2024	1607.96 (76317.55)	1566.66 (72690.95)	3174.62 (74483.70)
2025	1576.04 (73578.02)	1523.34 (69699.75)	3099.38 (71619.36)
2026	1662.42 (76531.20)	1601.74 (72418.89)	3264.16 (74456.49)
2027	1613.10 (73372.46)	1554.80 (69535.47)	3167.90 (71437.75)
2028	1623.26 (73175.41)	1561.96 (69232.21)	3185.22 (71187.15)
2029	1634.70 (73267.25)	1582.78 (69701.40)	3217.47 (71468.62)
2030	1653.40 (73782.40)	1589.31 (69607.62)	3242.71 (71675.48)
2031	1660.23 (73740.43)	1589.82 (69223.30)	3250.05 (71459.42)
2032	1675.52 (73925.59)	1609.09 (69531.20)	3284.61 (71705.51)
2033	1678.12 (73464.68)	1622.27 (69492.86)	3300.39 (71457.19)
2034	1692.96 (73537.16)	1637.20 (69510.61)	3330.16 (71500.92)
2035	1697.76 (73093.55)	1643.47 (69079.70)	3341.23 (71062.56)
2036	1730.09 (73675.42)	1676.21 (69609.41)	3406.30 (71616.87)
2037	1737.66 (72973.91)	1682.59 (68836.03)	3420.25 (70877.90)

	2038	1771.29 (73008.56)	1710.27 (68613.34)	3481.56 (70781.25)
	2039	1806.70 (72807.02)	1762.80 (69091.07)	3569.50 (70923.23)
	2040	1839.09 (72392.12)	1783.19 (68216.06)	3622.27 (70274.29)
**60-64 years**				
	1990	682.19 (86812.24)	688.79 (83936.65)	1370.98 (85343.32)
	1991	702.08 (87198.15)	702.74 (84188.12)	1404.81 (85666.00)
	1992	716.91 (87570.90)	714.04 (84526.54)	1430.95 (86024.85)
	1993	728.31 (87879.93)	725.92 (84913.96)	1454.23 (86373.92)
	1994	737.09 (88041.96)	736.75 (85356.12)	1473.84 (86678.55)
	1995	745.50 (88095.23)	750.26 (85868.26)	1495.76 (86963.95)
	1996	753.54 (87845.00)	763.80 (86020.19)	1517.35 (86916.85)
	1997	760.93 (87236.78)	775.50 (85531.80)	1536.43 (86367.79)
	1998	767.38 (86513.13)	783.95 (84776.31)	1551.32 (85626.64)
	1999	773.74 (85915.41)	790.87 (84066.37)	1564.61 (84970.72)
	2000	780.90 (85514.71)	797.66 (83403.23)	1578.56 (84434.57)
	2001	786.66 (85174.26)	800.82 (82753.65)	1587.48 (83935.71)
	2002	789.83 (84837.74)	800.54 (82144.75)	1590.37 (83460.46)
	2003	792.42 (84420.08)	796.92 (81437.65)	1589.35 (82897.83)
	2004	796.74 (84061.52)	795.22 (80709.98)	1591.96 (82353.26)
	2005	803.93 (83918.80)	795.69 (80200.58)	1599.62 (82027.15)
	2006	818.06 (83643.56)	805.33 (79682.70)	1623.39 (81630.62)
	2007	844.09 (82570.60)	829.32 (78734.02)	1673.41 (80623.60)
	2008	871.69 (81607.85)	859.71 (77913.47)	1731.40 (79730.67)
	2009	906.88 (80483.81)	897.77 (77123.17)	1804.65 (78776.14)
	2010	936.27 (79881.45)	936.31 (76927.92)	1872.58 (78376.84)

2011	987.16 (80150.69)	996.64 (77614.09)	1983.80 (78855.93)
2012	1034.25 (81229.80)	1053.38 (78986.47)	2087.63 (80082.15)
2013	1086.19 (82357.88)	1114.31 (80430.15)	2200.49 (81370.29)
2014	1136.70 (83654.04)	1173.73 (81866.45)	2310.43 (82736.28)
2015	1195.80 (84448.97)	1235.45 (82676.83)	2431.25 (83539.06)
2016	1262.97 (87018.12)	1287.70 (84096.60)	2550.67 (85518.26)
2017	1337.63 (90254.17)	1344.15 (85992.01)	2681.78 (88066.38)
2018	1385.98 (92118.42)	1389.80 (87630.87)	2775.77 (89815.54)
2019	1430.43 (94011.29)	1438.86 (89715.46)	2869.29 (91806.84)
2020	1447.15 (93565.43)	1457.54 (89030.09)	2904.70 (91233.33)
2021	1423.83 (90918.55)	1439.70 (86782.12)	2863.53 (88790.74)
2022	1486.93 (93140.46)	1501.93 (88789.47)	2988.86 (90902.03)
2023	1528.28 (92983.46)	1537.94 (88327.54)	3066.22 (90588.39)
2024	1611.18 (94544.02)	1615.66 (89533.94)	3226.85 (91967.32)
2025	1649.06 (93140.59)	1646.18 (87863.13)	3295.24 (90427.24)
2026	1731.16 (94326.95)	1726.30 (88964.33)	3457.46 (91570.96)
2027	1754.47 (92765.42)	1751.23 (87673.93)	3505.70 (90150.19)
2028	1790.84 (92452.69)	1774.80 (86918.56)	3565.64 (89612.69)
2029	1824.50 (92317.93)	1806.97 (86963.18)	3631.47 (89573.50)
2030	1862.40 (92651.73)	1835.87 (87108.43)	3698.27 (89814.48)
2031	1886.29 (92498.04)	1848.55 (86655.13)	3734.85 (89510.80)
2032	1907.87 (92408.14)	1875.11 (86934.92)	3782.98 (89611.70)
2033	1918.97 (92091.78)	1891.32 (86893.53)	3810.29 (89436.02)
2034	1927.41 (91938.91)	1900.14 (86723.58)	3827.54 (89273.69)
2035	1928.67 (91569.78)	1906.56 (86530.95)	3835.23 (88993.60)

	2036	1946.70 (91963.37)	1919.76 (86609.61)	3866.47 (89224.87)
	2037	1948.66 (91415.30)	1922.79 (86075.86)	3871.46 (88683.10)
	2038	1959.44 (91177.08)	1937.90 (85988.17)	3897.34 (88520.97)
	2039	1985.78 (91652.91)	1968.66 (86566.59)	3954.44 (89048.18)
	2040	1983.75 (90716.56)	1963.31 (85454.77)	3947.07 (88020.70)
**65-69 years**				
	1990	626.00 (109331.07)	639.30 (96526.55)	1265.31 (102463.60)
	1991	647.41 (109291.10)	656.81 (96539.64)	1304.22 (102474.63)
	1992	670.55 (109341.35)	674.78 (96699.66)	1345.33 (102612.93)
	1993	694.95 (109376.31)	693.12 (97072.14)	1388.07 (102865.68)
	1994	720.08 (109619.41)	713.67 (97688.69)	1433.75 (103337.32)
	1995	743.90 (110063.55)	734.42 (98610.61)	1478.32 (104059.40)
	1996	764.67 (110425.92)	754.39 (99474.39)	1519.06 (104701.42)
	1997	780.38 (110781.39)	770.32 (100245.70)	1550.70 (105284.67)
	1998	792.48 (111000.97)	785.13 (100812.56)	1577.60 (105685.42)
	1999	802.98 (111090.43)	798.27 (101336.86)	1601.25 (106004.04)
	2000	813.54 (111034.28)	813.54 (101808.40)	1627.08 (106221.40)
	2001	825.73 (110820.11)	831.34 (102130.98)	1657.07 (106283.60)
	2002	839.57 (110439.70)	850.60 (102113.05)	1690.17 (106086.15)
	2003	853.96 (110116.63)	869.13 (102110.65)	1723.10 (105927.46)
	2004	868.61 (109995.62)	886.72 (102248.56)	1755.33 (105940.80)
	2005	884.10 (110088.73)	904.13 (102418.42)	1788.24 (106072.27)
	2006	898.48 (110208.47)	917.93 (102613.22)	1816.41 (106234.72)
	2007	910.69 (110354.58)	929.17 (102974.52)	1839.86 (106499.88)
	2008	923.76 (110517.44)	938.56 (103383.19)	1862.32 (106803.02)

2009	938.66 (110769.56)	950.58 (103771.57)	1889.24 (107134.40)
2010	956.70 (111349.28)	964.84 (104395.49)	1921.54 (107745.60)
2011	985.21 (112076.58)	992.73 (105255.69)	1977.95 (108546.14)
2012	1031.12 (111944.50)	1040.95 (105648.28)	2072.07 (108690.38)
2013	1080.67 (112046.97)	1097.36 (106121.96)	2178.03 (108981.34)
2014	1138.72 (111673.82)	1160.64 (106220.63)	2299.36 (108853.02)
2015	1182.97 (111395.53)	1215.07 (106263.36)	2398.03 (108734.62)
2016	1274.23 (114023.43)	1302.98 (107905.93)	2577.21 (110846.27)
2017	1362.87 (117842.86)	1383.21 (110235.54)	2746.08 (113884.20)
2018	1435.61 (119738.02)	1458.70 (111865.37)	2894.31 (115636.54)
2019	1502.76 (121565.97)	1537.60 (113924.53)	3040.36 (117577.54)
2020	1590.96 (124935.49)	1645.17 (117829.38)	3236.13 (121218.99)
2021	1617.38 (123555.79)	1674.75 (116518.93)	3292.13 (119873.00)
2022	1671.97 (124674.45)	1725.46 (117012.02)	3397.43 (120661.54)
2023	1721.01 (126094.74)	1781.44 (118517.96)	3502.44 (122123.74)
2024	1747.26 (126538.65)	1812.76 (119044.98)	3560.02 (122608.65)
2025	1770.46 (126882.30)	1832.38 (118968.70)	3602.85 (122730.24)
2026	1791.49 (126656.23)	1853.89 (118699.85)	3645.38 (122481.06)
2027	1822.60 (126250.75)	1882.37 (118116.94)	3704.97 (121982.98)
2028	1872.26 (125810.04)	1932.80 (117731.53)	3805.06 (121572.63)
2029	1940.42 (125599.13)	2009.66 (118018.52)	3950.08 (121624.54)
2030	2018.53 (125620.70)	2093.26 (118309.50)	4111.80 (121789.19)
2031	2092.63 (125524.84)	2162.59 (117943.04)	4255.22 (121553.65)
2032	2155.59 (125388.93)	2227.49 (117961.15)	4383.08 (121500.85)
2033	2205.84 (125203.70)	2277.18 (117918.86)	4483.02 (121394.26)

	2034	2246.60 (124892.27)	2310.62 (117532.16)	4557.22 (121048.86)
	2035	2292.79 (125222.96)	2342.94 (117448.23)	4635.73 (121169.06)
	2036	2322.99 (124968.22)	2368.05 (117235.55)	4691.04 (120941.36)
	2037	2354.99 (125061.75)	2394.09 (117187.58)	4749.09 (120964.32)
	2038	2373.93 (124843.57)	2408.13 (116776.78)	4782.06 (120646.71)
	2039	2397.90 (125272.14)	2450.74 (118026.15)	4848.64 (121501.81)
	2040	2395.92 (124508.96)	2433.25 (116493.15)	4829.17 (120336.81)
**70-74 years**				
	1990	503.10 (134083.58)	523.44 (111389.59)	1026.54 (121465.09)
	1991	520.29 (133736.15)	541.76 (111391.85)	1062.05 (121322.10)
	1992	538.66 (132866.10)	561.43 (110845.53)	1100.09 (120635.37)
	1993	558.00 (132000.95)	582.55 (110095.76)	1140.55 (119823.97)
	1994	578.10 (131275.11)	602.85 (109633.88)	1180.95 (119258.01)
	1995	599.11 (131026.03)	622.89 (109607.23)	1222.00 (119157.02)
	1996	621.63 (131105.53)	643.12 (109972.15)	1264.75 (119434.66)
	1997	645.93 (131242.57)	663.91 (110445.33)	1309.84 (119807.59)
	1998	671.63 (131326.21)	685.21 (111106.45)	1356.84 (120272.69)
	1999	698.33 (131658.19)	708.18 (111937.33)	1406.51 (120930.91)
	2000	723.79 (132203.94)	731.13 (113106.75)	1454.91 (121864.14)
	2001	746.47 (132682.05)	754.03 (114328.02)	1500.50 (122777.16)
	2002	765.72 (133381.97)	775.09 (115762.70)	1540.81 (123896.08)
	2003	782.91 (134082.75)	797.05 (117195.62)	1579.96 (124996.52)
	2004	800.07 (134729.84)	819.48 (118766.78)	1619.55 (126150.53)
	2005	817.42 (135131.51)	843.60 (120141.22)	1661.02 (127078.59)
	2006	836.04 (135266.46)	870.01 (121273.82)	1706.05 (127749.78)

2007	855.64 (135091.28)	898.44 (122022.37)	1754.08 (128065.82)
2008	875.13 (134900.48)	926.59 (122784.17)	1801.72 (128385.05)
2009	894.09 (134888.00)	953.02 (123619.70)	1847.11 (128829.10)
2010	913.73 (135117.48)	978.64 (124417.55)	1892.37 (129364.00)
2011	933.76 (135594.11)	1001.28 (125358.68)	1935.03 (130097.60)
2012	953.02 (136364.97)	1022.33 (126684.73)	1975.35 (131177.36)
2013	975.10 (137343.45)	1042.58 (128193.71)	2017.68 (132458.31)
2014	1000.20 (138475.24)	1065.27 (129562.12)	2065.48 (133730.39)
2015	1028.06 (139893.60)	1087.96 (130902.17)	2116.02 (135121.60)
2016	1083.73 (143684.24)	1131.19 (133135.98)	2214.92 (138096.40)
2017	1160.61 (146362.38)	1195.17 (134337.76)	2355.78 (140004.54)
2018	1223.11 (146987.95)	1262.34 (134947.66)	2485.45 (140615.93)
2019	1294.63 (146944.01)	1343.58 (135738.08)	2638.21 (141015.21)
2020	1382.94 (152537.63)	1458.32 (141086.31)	2841.26 (146437.15)
2021	1432.69 (150824.16)	1519.44 (139559.77)	2952.13 (144808.42)
2022	1492.77 (150820.87)	1591.99 (139740.71)	3084.76 (144891.82)
2023	1555.98 (151544.40)	1658.87 (139973.94)	3214.85 (145344.92)
2024	1615.74 (152012.52)	1732.29 (140935.79)	3348.02 (146072.47)
2025	1671.44 (152299.30)	1784.82 (140455.76)	3456.25 (145944.28)
2026	1721.10 (152351.91)	1839.81 (140512.27)	3560.91 (145996.02)
2027	1763.30 (152173.65)	1888.41 (140464.11)	3651.70 (145884.61)
2028	1789.90 (151594.33)	1918.80 (139910.98)	3708.71 (145316.10)
2029	1811.48 (151437.64)	1946.23 (139954.06)	3757.71 (145264.28)
2030	1834.76 (151554.80)	1972.30 (140082.36)	3807.06 (145386.32)
2031	1863.55 (151614.72)	1998.99 (139866.53)	3862.54 (145298.52)

	2032	1898.29 (151084.27)	2038.39 (139627.03)	3936.68 (144926.60)
	2033	1958.10 (150946.74)	2100.99 (139546.39)	4059.09 (144822.79)
	2034	2037.61 (151068.22)	2173.17 (138996.34)	4210.77 (144587.36)
	2035	2124.45 (151216.68)	2264.81 (139261.36)	4389.26 (144802.42)
	2036	2204.82 (151069.95)	2348.50 (139209.44)	4553.32 (144710.81)
	2037	2277.30 (151149.67)	2416.23 (138962.11)	4693.53 (144620.05)
	2038	2334.95 (151066.20)	2478.23 (139270.35)	4813.18 (144753.58)
	2039	2394.52 (151567.77)	2553.48 (140860.66)	4948.01 (145846.65)
	2040	2423.13 (150526.42)	2558.65 (139007.15)	4981.78 (144381.38)
**75-79 years**				
	1990	365.06 (145685.36)	411.56 (113530.61)	776.62 (126672.77)
	1991	370.46 (146746.74)	416.47 (114981.97)	786.93 (128028.21)
	1992	376.80 (148553.54)	421.81 (117014.11)	798.62 (130040.54)
	1993	384.37 (150788.34)	427.61 (119546.73)	811.98 (132546.59)
	1994	394.24 (152683.84)	437.43 (121566.38)	831.67 (134566.87)
	1995	408.28 (153484.17)	452.31 (122819.54)	860.60 (135679.89)
	1996	425.67 (153596.63)	471.81 (123221.91)	897.48 (135975.69)
	1997	444.10 (153084.83)	492.87 (123044.39)	936.96 (135662.39)
	1998	463.15 (152508.39)	515.18 (122601.66)	978.33 (135148.13)
	1999	483.02 (152012.73)	536.86 (122452.42)	1019.87 (134873.90)
	2000	503.36 (151887.22)	557.70 (122701.29)	1061.06 (135008.25)
	2001	524.84 (152102.81)	578.77 (123432.14)	1103.61 (135586.37)
	2002	548.48 (152543.26)	600.95 (124435.98)	1149.43 (136431.51)
	2003	574.10 (153088.97)	624.66 (125818.84)	1198.76 (137553.47)
	2004	601.08 (153976.73)	650.78 (127429.47)	1251.87 (138930.54)

2005	627.46 (155054.21)	677.13 (129347.16)	1304.59 (140555.07)
2006	651.76 (155836.45)	703.47 (131197.47)	1355.23 (141994.33)
2007	673.20 (156670.76)	729.01 (133349.39)	1402.21 (143612.79)
2008	692.47 (157343.29)	755.61 (135476.11)	1448.08 (145120.67)
2009	711.60 (157885.26)	783.28 (137778.78)	1494.88 (146670.10)
2010	730.62 (158083.10)	812.48 (139750.00)	1543.10 (147869.43)
2011	751.32 (158207.79)	844.13 (141465.79)	1595.46 (148885.24)
2012	773.72 (158292.73)	877.92 (142798.81)	1651.65 (149661.24)
2013	796.54 (158512.95)	910.98 (144084.61)	1707.52 (150473.95)
2014	818.88 (158947.32)	940.57 (145259.04)	1759.44 (151324.27)
2015	841.76 (159580.14)	967.73 (146156.21)	1809.49 (152108.53)
2016	879.56 (163083.93)	999.23 (148250.44)	1878.79 (154843.89)
2017	919.09 (167241.52)	1029.84 (150843.90)	1948.92 (158156.74)
2018	949.48 (169398.27)	1056.80 (153239.05)	2006.28 (160484.01)
2019	980.17 (171343.13)	1091.09 (156207.92)	2071.26 (163022.44)
2020	1035.81 (174944.23)	1140.21 (157992.74)	2176.02 (165632.34)
2021	1057.24 (173535.60)	1151.86 (155858.84)	2209.10 (163846.28)
2022	1095.90 (173758.39)	1194.24 (156394.44)	2290.14 (164248.88)
2023	1150.23 (174575.52)	1253.53 (156902.91)	2403.76 (164890.32)
2024	1219.21 (175919.87)	1330.16 (157682.92)	2549.37 (165908.17)
2025	1278.93 (175229.58)	1401.73 (157017.66)	2680.66 (165209.66)
2026	1344.45 (175439.21)	1482.74 (157425.16)	2827.18 (165506.62)
2027	1394.93 (174406.74)	1541.79 (156244.52)	2936.71 (164375.29)
2028	1444.99 (173863.55)	1603.01 (155974.73)	3048.00 (163972.98)
2029	1492.70 (173150.46)	1655.70 (155123.07)	3148.41 (163177.84)

	2030	1553.58 (174186.56)	1718.76 (155544.20)	3272.34 (163870.72)
	2031	1595.94 (173513.80)	1764.41 (154764.09)	3360.35 (163136.35)
	2032	1634.22 (172955.28)	1805.07 (154030.49)	3439.29 (162478.10)
	2033	1663.11 (172472.38)	1843.93 (154078.78)	3507.04 (162286.24)
	2034	1688.60 (172524.77)	1871.19 (154003.13)	3559.79 (162266.55)
	2035	1714.49 (172693.94)	1905.68 (154680.09)	3620.17 (162718.56)
	2036	1744.33 (172631.82)	1930.03 (154075.66)	3674.36 (162360.73)
	2037	1784.98 (172395.13)	1973.25 (153961.09)	3758.23 (162198.55)
	2038	1849.15 (172566.31)	2042.26 (154242.11)	3891.40 (162438.51)
	2039	1937.08 (173463.38)	2144.58 (155700.09)	4081.65 (163653.47)
	2040	2004.70 (172010.65)	2208.85 (153928.21)	4213.54 (162032.30)
**80+ years**				
	1990	323.89 (164808.48)	462.37 (128181.90)	786.26 (141099.09)
	1991	335.28 (164443.52)	481.38 (128599.53)	816.66 (141238.80)
	1992	346.94 (164301.01)	500.37 (129258.74)	847.32 (141627.10)
	1993	358.63 (164313.90)	519.74 (129932.46)	878.37 (142069.58)
	1994	370.24 (164682.93)	538.98 (130955.93)	909.22 (142870.64)
	1995	381.68 (165528.24)	557.33 (132160.46)	939.02 (143955.84)
	1996	393.11 (167022.82)	574.33 (133871.51)	967.43 (145615.67)
	1997	405.44 (169005.26)	592.01 (136026.29)	997.44 (147745.12)
	1998	418.55 (171078.47)	610.69 (138307.88)	1029.24 (149991.82)
	1999	433.24 (172868.44)	632.24 (140226.18)	1065.48 (151888.15)
	2000	450.80 (173785.50)	657.13 (141627.43)	1107.92 (153159.04)
	2001	470.38 (174323.92)	684.58 (142722.31)	1154.96 (154099.46)
	2002	491.02 (174706.58)	713.37 (143693.02)	1204.39 (154903.87)

2003	512.44 (175101.49)	743.88 (144577.23)	1256.32 (155644.35)
2004	535.61 (175328.20)	776.99 (145452.37)	1312.61 (156321.73)
2005	561.09 (175381.32)	812.15 (146186.00)	1373.25 (156854.79)
2006	588.61 (175460.18)	848.90 (146969.55)	1437.51 (157437.20)
2007	618.84 (175501.48)	887.73 (147782.82)	1506.57 (158035.44)
2008	650.67 (175560.69)	928.62 (148716.62)	1579.29 (158715.23)
2009	684.41 (175920.32)	972.98 (149825.67)	1657.39 (159601.75)
2010	720.10 (176589.32)	1020.30 (151245.53)	1740.40 (160793.69)
2011	756.51 (177362.79)	1069.77 (152831.64)	1826.27 (162120.03)
2012	793.94 (178413.60)	1120.45 (154712.23)	1914.39 (163732.90)
2013	830.19 (179241.47)	1172.80 (156507.54)	2002.99 (165191.62)
2014	865.52 (179849.38)	1225.81 (158249.74)	2091.32 (166526.80)
2015	901.18 (180185.40)	1278.57 (159631.54)	2179.75 (167532.47)
2016	947.34 (182052.22)	1335.90 (161073.74)	2283.24 (169161.65)
2017	997.86 (184419.02)	1396.71 (162643.80)	2394.57 (171060.62)
2018	1043.74 (185902.23)	1461.37 (164635.87)	2505.11 (172875.52)
2019	1090.14 (187614.55)	1529.49 (167287.69)	2619.63 (175186.21)
2020	1180.89 (192126.77)	1603.90 (170830.84)	2784.79 (179256.46)
2021	1220.58 (191653.92)	1643.27 (169989.03)	2863.85 (178593.44)
2022	1265.70 (192583.71)	1692.30 (170538.80)	2958.01 (179322.06)
2023	1314.79 (194026.26)	1743.10 (171387.24)	3057.89 (180439.63)
2024	1371.52 (195811.91)	1800.43 (172540.25)	3171.95 (181887.11)
2025	1411.82 (194637.66)	1838.16 (171389.05)	3249.98 (180768.81)
2026	1468.02 (195149.27)	1900.79 (172023.46)	3368.81 (181390.43)
2027	1511.44 (193524.58)	1947.32 (170563.45)	3458.76 (179890.33)

	2028	1574.63 (193254.40)	2022.73 (170477.76)	3597.36 (179750.87)
	2029	1642.53 (192169.99)	2108.06 (169983.36)	3750.59 (179035.67)
	2030	1733.94 (193097.68)	2208.84 (170016.58)	3942.79 (179449.66)
	2031	1813.12 (192463.88)	2308.56 (169685.25)	4121.68 (179004.82)
	2032	1888.32 (191731.96)	2410.20 (169597.80)	4298.52 (178658.22)
	2033	1966.89 (191077.03)	2510.03 (169100.44)	4476.92 (178099.89)
	2034	2060.28 (191149.88)	2623.49 (168939.34)	4683.77 (178039.11)
	2035	2160.49 (191617.16)	2746.95 (169203.37)	4907.43 (178389.82)
	2036	2253.93 (191674.79)	2857.50 (168804.65)	5111.43 (178179.38)
	2037	2343.12 (191914.18)	2967.43 (168797.61)	5310.55 (178272.08)
	2038	2423.92 (191951.39)	3075.13 (169066.82)	5499.05 (178444.27)
	2039	2510.92 (192556.78)	3198.01 (170256.50)	5708.93 (179394.22)
	2040	2577.62 (191513.02)	3272.33 (168844.40)	5849.94 (178134.97)
**all ages**				
	1990	22871.76 (84906.78)	23177.75 (87262.41)	46049.51 (86076.30)
	1991	23045.60 (84209.61)	23294.62 (86402.62)	46340.22 (85297.91)
	1992	23232.00 (83597.95)	23420.68 (85617.96)	46652.68 (84599.99)
	1993	23440.47 (83107.27)	23570.92 (84956.88)	47011.40 (84024.47)
	1994	23687.75 (82781.25)	23768.33 (84474.43)	47456.09 (83620.71)
	1995	23984.24 (82643.97)	24024.20 (84200.77)	48008.44 (83415.75)
	1996	24347.95 (82737.05)	24318.25 (84052.77)	48666.20 (83389.32)
	1997	24764.14 (83009.74)	24616.91 (83918.26)	49381.06 (83460.17)
	1998	25200.88 (83360.98)	24917.22 (83797.83)	50118.10 (83577.60)
	1999	25633.52 (83704.19)	25224.22 (83703.58)	50857.74 (83703.89)
	2000	26034.14 (83936.37)	25544.14 (83648.24)	51578.28 (83793.42)

2001	26356.09 (83905.35)	25817.68 (83434.75)	52173.77 (83671.82)
2002	26589.29 (83585.04)	26000.18 (82925.46)	52589.46 (83257.64)
2003	26756.94 (83064.76)	26121.13 (82228.80)	52878.07 (82649.69)
2004	26886.47 (82436.44)	26214.37 (81455.89)	53100.83 (81949.44)
2005	27006.00 (81790.43)	26314.24 (80717.58)	53320.24 (81257.43)
2006	27027.77 (80839.76)	26345.58 (79771.69)	53373.35 (80309.00)
2007	26915.38 (79477.05)	26271.55 (78507.21)	53186.93 (78995.02)
2008	26774.92 (78074.93)	26186.97 (77249.40)	52961.89 (77664.55)
2009	26725.50 (77001.65)	26196.53 (76314.43)	52922.03 (76659.93)
2010	26899.59 (76599.49)	26415.76 (76003.44)	53315.35 (76303.01)
2011	27310.75 (76863.43)	26874.28 (76374.49)	54185.02 (76620.15)
2012	27836.69 (77429.71)	27472.46 (77123.83)	55309.15 (77277.47)
2013	28428.21 (78165.60)	28148.07 (78071.12)	56576.28 (78118.56)
2014	29037.38 (78946.99)	28831.26 (79030.45)	57868.64 (78988.55)
2015	29618.11 (79633.11)	29452.15 (79802.73)	59070.27 (79717.59)
2016	30630.62 (81439.04)	30339.72 (81265.83)	60970.34 (81352.76)
2017	31726.81 (83438.91)	31283.25 (82856.16)	63010.06 (83148.57)
2018	32450.92 (84461.48)	31974.48 (83780.29)	64425.40 (84122.02)
2019	33150.58 (85418.19)	32666.25 (84704.62)	65816.83 (85062.53)
2020	33883.55 (85619.44)	33379.24 (84951.02)	67262.79 (85286.43)
2021	33149.50 (82929.92)	32618.60 (82175.67)	65768.10 (82554.12)
2022	34458.36 (85368.24)	33959.28 (84708.93)	68417.64 (85039.71)
2023	34061.18 (83588.07)	33578.48 (82952.59)	67639.66 (83271.38)
2024	35188.40 (85563.52)	34691.06 (84897.90)	69879.46 (85231.78)
2025	34976.64 (84294.45)	34358.87 (83319.61)	69335.51 (83808.54)

2026	36158.62 (86395.95)	35508.98 (85348.52)	71667.60 (85873.79)
2027	35599.74 (84355.74)	35006.06 (83419.58)	70605.80 (83888.98)
2028	35933.25 (84463.71)	35345.88 (83530.14)	71279.14 (83998.17)
2029	36136.79 (84284.38)	35584.91 (83418.49)	71721.70 (83852.53)
2030	37219.76 (86161.64)	36463.45 (84812.54)	73683.21 (85488.69)
2031	37259.12 (85631.08)	36431.83 (84100.84)	73690.94 (84867.65)
2032	37757.52 (86173.47)	36969.90 (84721.70)	74727.41 (85449.07)
2033	37791.11 (85672.56)	37067.91 (84349.33)	74859.02 (85012.19)
2034	38237.87 (86125.89)	37415.17 (84562.22)	75653.04 (85345.39)
2035	38469.89 (86109.55)	37653.49 (84544.40)	76123.39 (85328.19)
2036	38886.76 (86521.27)	38046.96 (84889.82)	76933.71 (85706.69)
2037	39111.79 (86520.42)	38282.91 (84898.72)	77394.70 (85710.58)
2038	39379.66 (86630.41)	38557.54 (85010.02)	77937.19 (85821.11)
2039	40006.30 (87540.91)	39378.38 (86335.07)	79384.67 (86938.58)
2040	39771.12 (86582.30)	38959.60 (84960.06)	78730.72 (85771.87)

Figure S3. SHAP summary plot of feature contributions for Chad, ranked by mean |SHAP| values, and SHAP dependence plots for each feature in the XGBoost model predicting diarrheal incidence rate

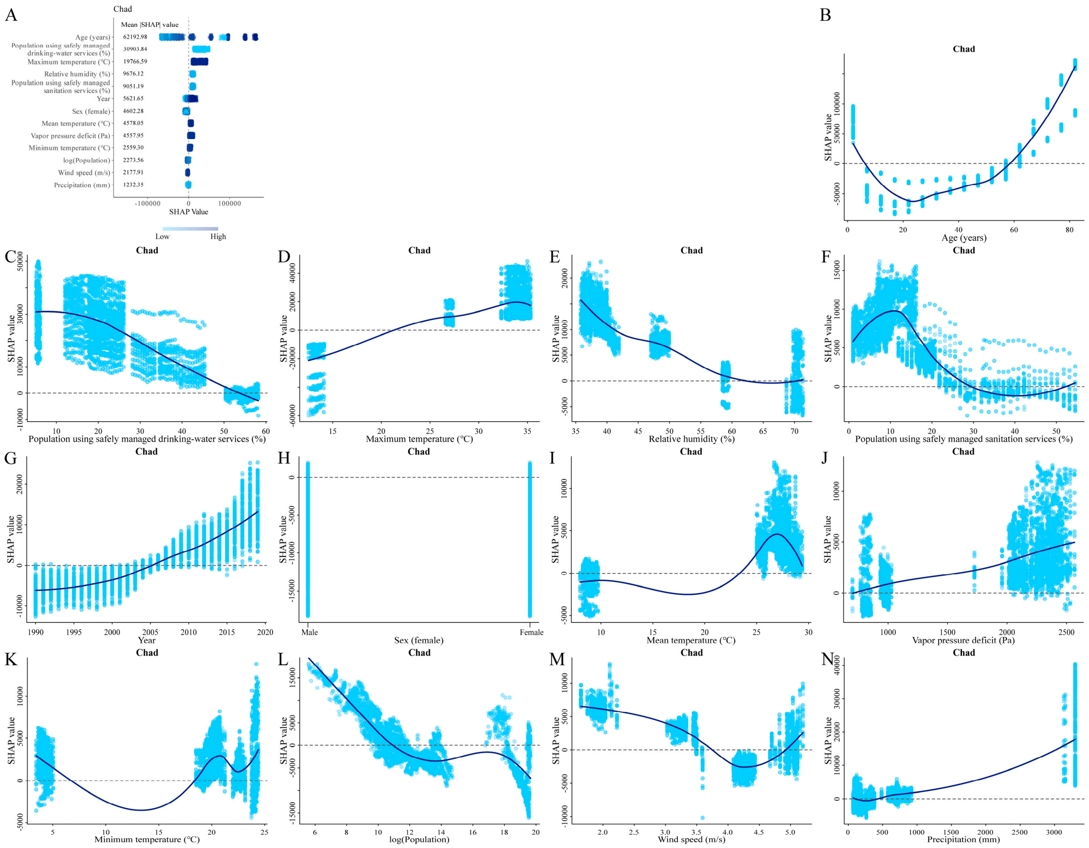

Abbreviations: SHAP, SHapley Additive exPlanations.

Figure S4. SHAP summary plot of feature contributions for Mauritania, ranked by mean |SHAP| values, and SHAP dependence plots for each feature in the XGBoost model predicting diarrheal incidence rate

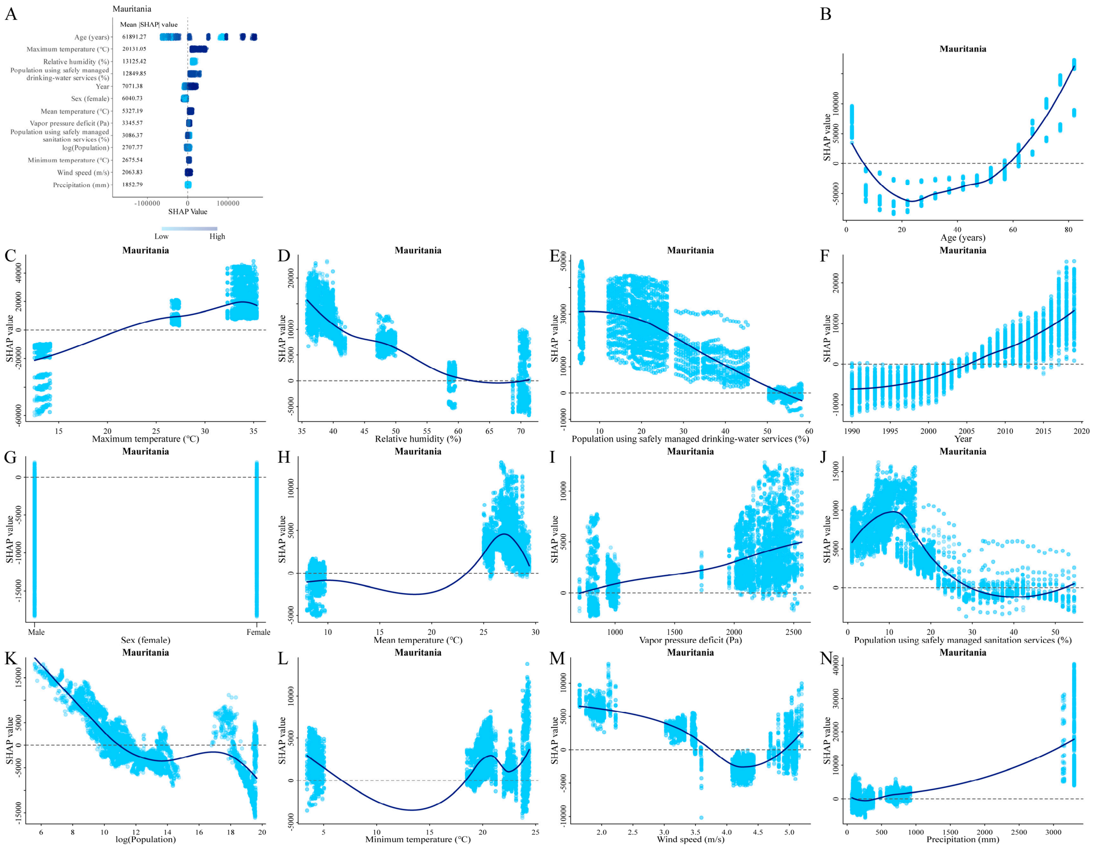

Abbreviations: SHAP, SHapley Additive exPlanations.

Figure S5. SHAP summary plot of feature contributions for Niger, ranked by mean |SHAP| values, and SHAP dependence plots for each feature in the XGBoost model predicting diarrheal incidence rate

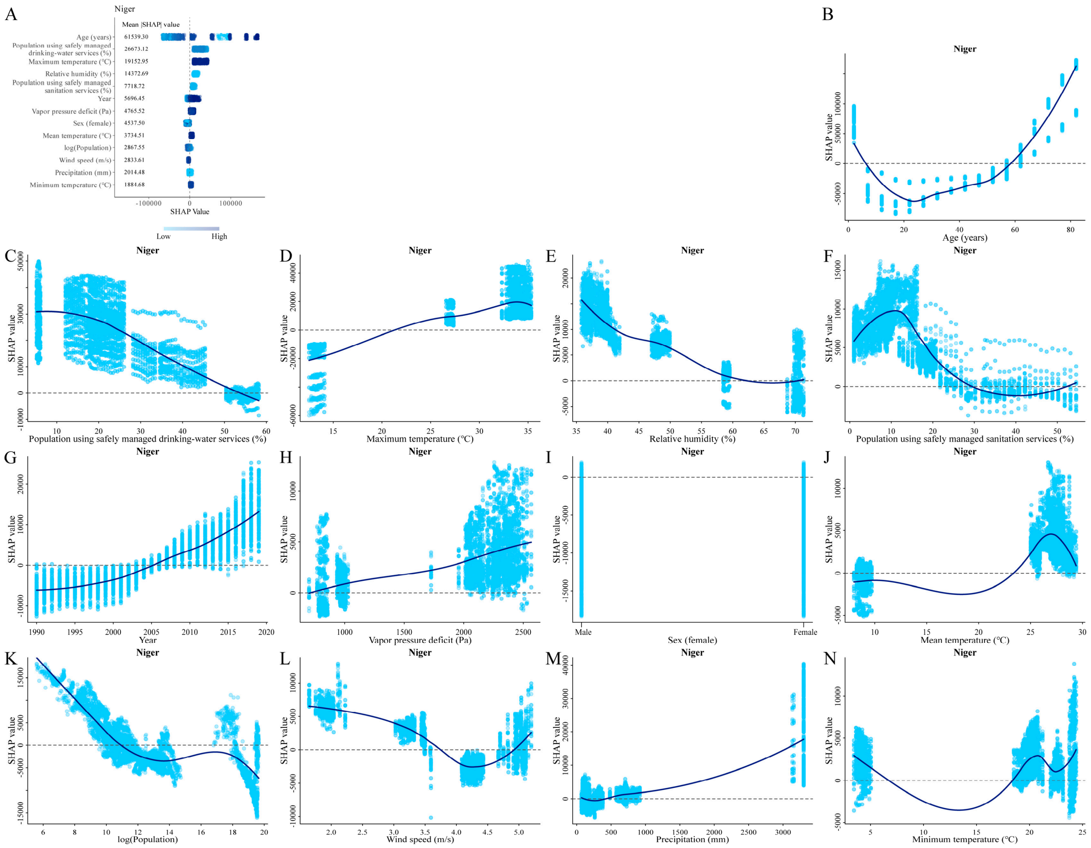

Abbreviations: SHAP, SHapley Additive exPlanations.

Figure S6. SHAP summary plot of feature contributions for Senegal, ranked by mean |SHAP| values, and SHAP dependence plots for each feature in the XGBoost model predicting diarrheal incidence rate

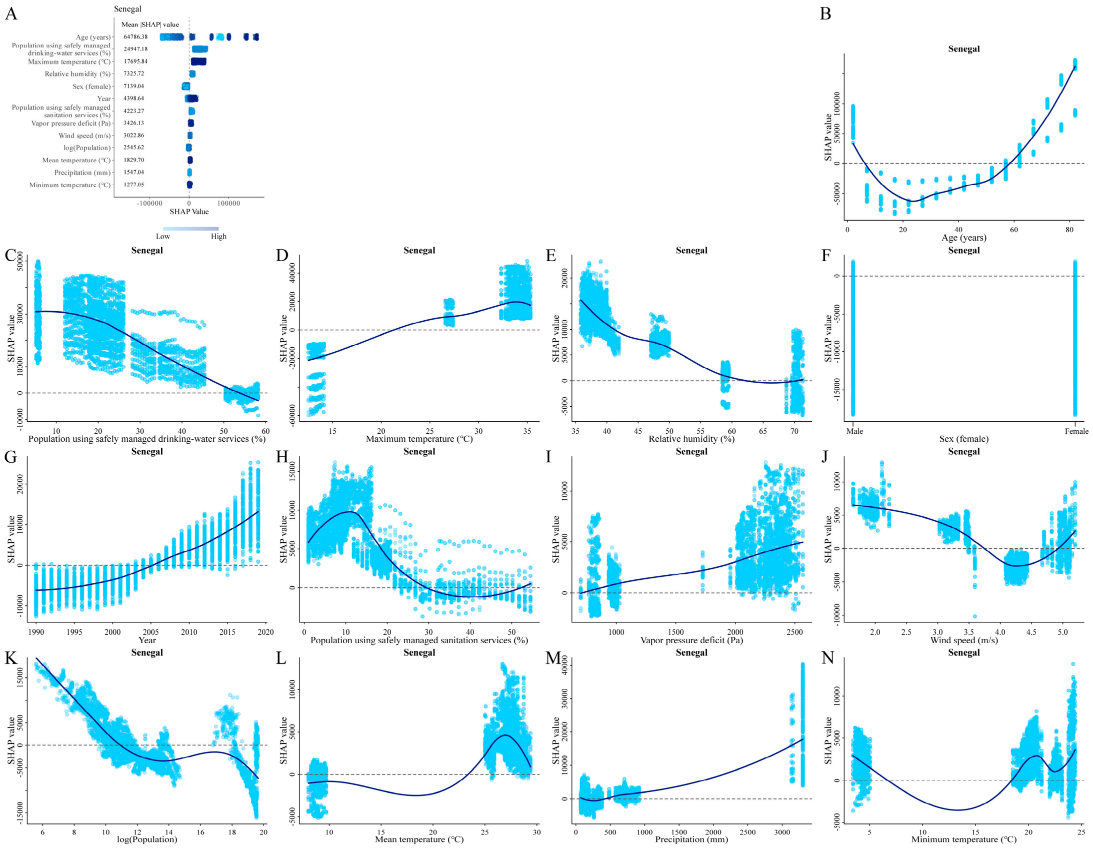

Abbreviations: SHAP, SHapley Additive exPlanations.

Figure S7. SHAP summary plot of feature contributions for Solomon Islands, ranked by mean |SHAP| values, and SHAP dependence plots for each feature in the XGBoost model predicting diarrheal incidence rate

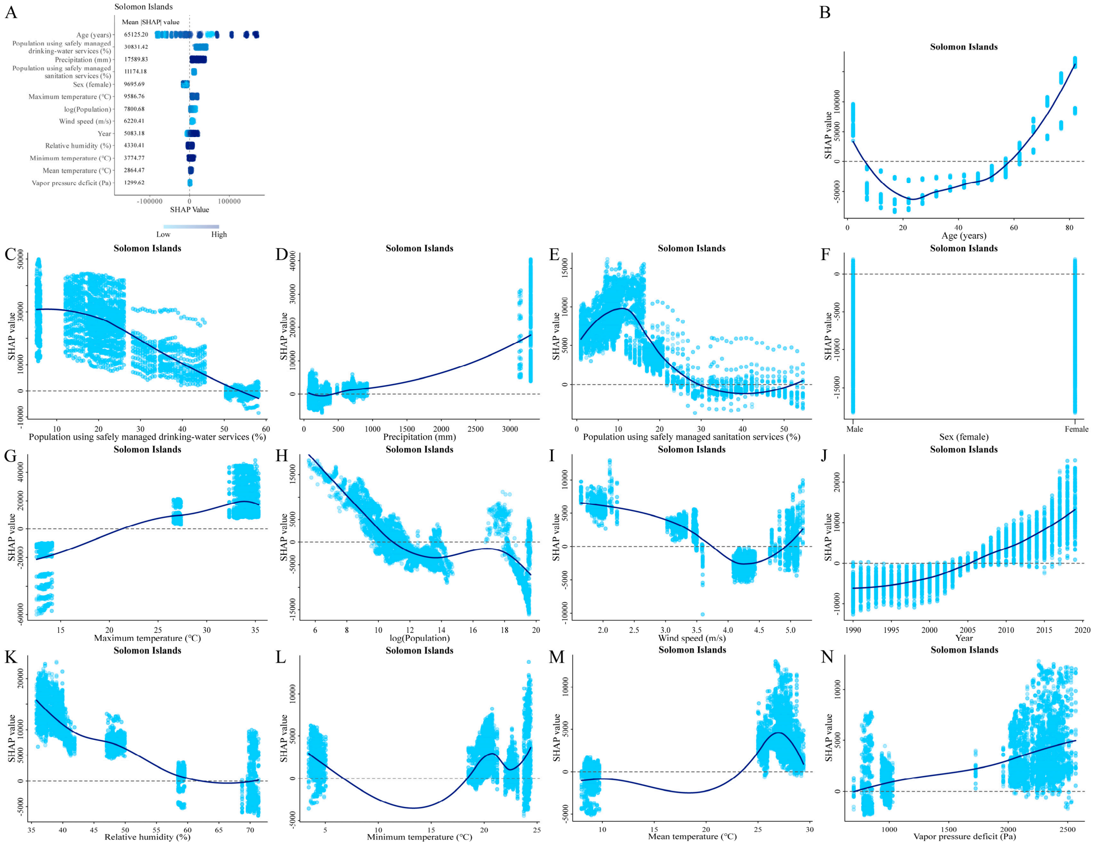

Abbreviations: SHAP, SHapley Additive exPlanations.

Figure S8. The global population by age group for different years

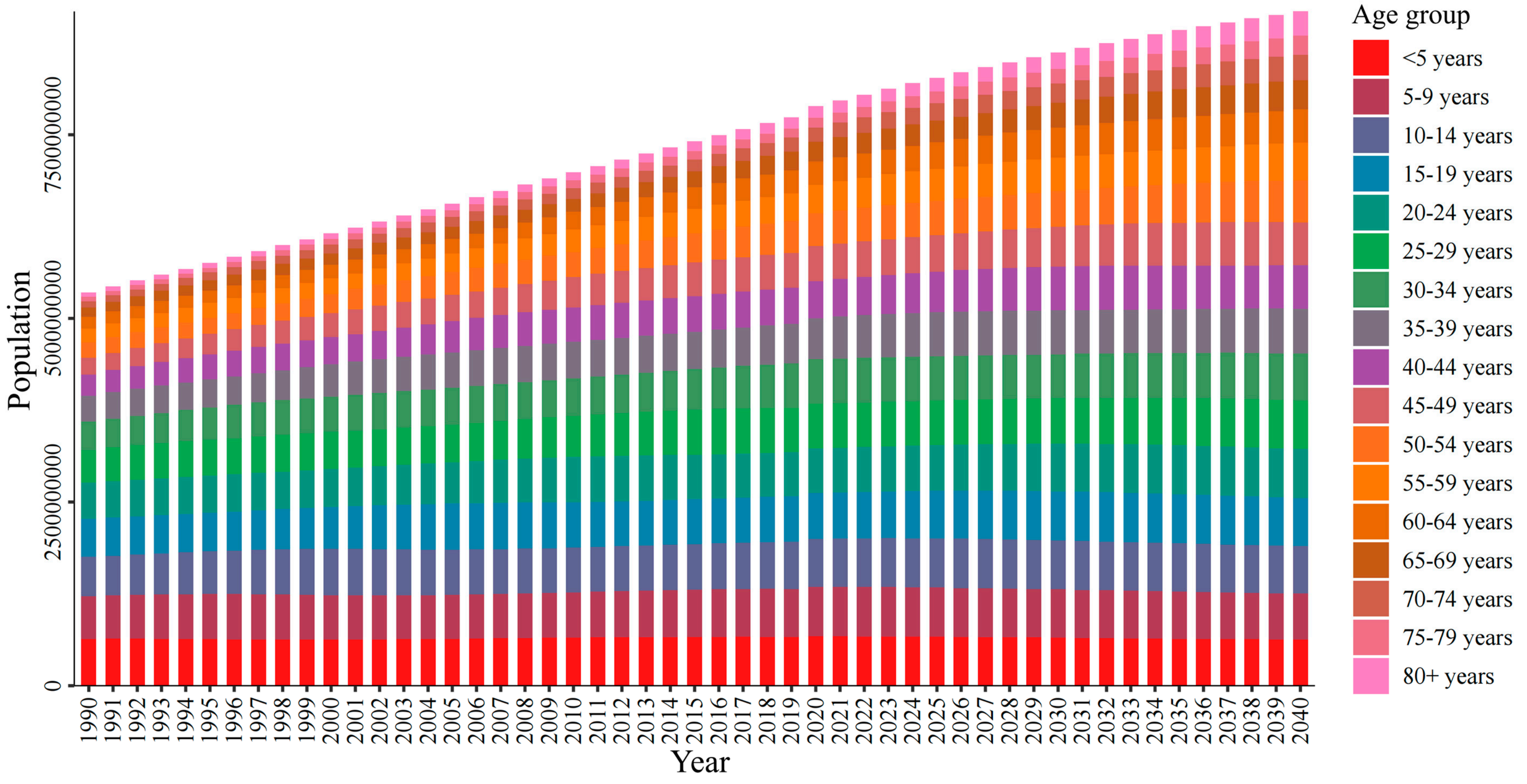

Table S7. The global population composition (%) by year from 1990–2040

Year	<5 years	5−9 years	10−14 years	15−19 years	20−24 years	25−29 years	30−34 years	35−39 years	40−44 years	45−49 years	50−54 years	55−59 years	60−64 years	65−69 years	70−74 years	75−79 years	80+ years
1990	11.82	10.94	10.03	9.71	9.21	8.28	7.21	6.59	5.35	4.34	3.97	3.47	3.00	2.31	1.58	1.15	1.04
1991	11.69	10.91	10.00	9.54	9.16	8.39	7.20	6.65	5.53	4.34	3.96	3.46	3.02	2.34	1.61	1.13	1.06
1992	11.53	10.89	10.00	9.39	9.09	8.47	7.25	6.68	5.63	4.43	3.92	3.47	3.02	2.38	1.65	1.11	1.08
1993	11.35	10.87	10.02	9.26	9.00	8.50	7.37	6.67	5.75	4.54	3.88	3.47	3.01	2.41	1.70	1.09	1.11
1994	11.15	10.83	10.05	9.18	8.90	8.49	7.52	6.64	5.84	4.69	3.84	3.47	3.00	2.44	1.74	1.09	1.12
1995	10.95	10.76	10.08	9.13	8.78	8.46	7.67	6.62	5.99	4.80	3.81	3.47	2.99	2.47	1.78	1.10	1.13
1996	10.76	10.65	10.13	9.13	8.66	8.43	7.78	6.63	6.04	4.96	3.81	3.46	2.99	2.49	1.81	1.13	1.14
1997	10.59	10.48	10.19	9.15	8.54	8.39	7.86	6.69	6.07	5.06	3.90	3.43	3.01	2.49	1.85	1.17	1.14
1998	10.43	10.29	10.23	9.20	8.45	8.33	7.88	6.81	6.06	5.17	4.00	3.40	3.02	2.49	1.88	1.21	1.14
1999	10.29	10.09	10.25	9.25	8.39	8.25	7.88	6.97	6.04	5.25	4.15	3.37	3.03	2.49	1.91	1.24	1.15
2000	10.15	9.89	10.23	9.30	8.36	8.17	7.86	7.12	6.02	5.39	4.26	3.34	3.04	2.49	1.94	1.28	1.18
2001	10.02	9.72	10.13	9.38	8.36	8.06	7.84	7.23	6.03	5.44	4.42	3.36	3.03	2.50	1.96	1.31	1.20
2002	9.91	9.58	9.97	9.46	8.39	7.96	7.82	7.31	6.10	5.47	4.51	3.45	3.02	2.52	1.97	1.33	1.23
2003	9.80	9.46	9.78	9.53	8.44	7.88	7.77	7.34	6.22	5.47	4.61	3.55	3.00	2.54	1.98	1.36	1.26
2004	9.71	9.35	9.58	9.57	8.50	7.83	7.71	7.34	6.36	5.46	4.70	3.70	2.98	2.56	1.98	1.39	1.30
2005	9.63	9.25	9.38	9.57	8.55	7.80	7.64	7.32	6.50	5.45	4.83	3.80	2.97	2.57	1.99	1.41	1.33
2006	9.56	9.16	9.21	9.49	8.63	7.80	7.55	7.31	6.61	5.46	4.88	3.95	2.99	2.57	2.01	1.44	1.37
2007	9.50	9.08	9.06	9.35	8.72	7.83	7.46	7.29	6.68	5.53	4.92	4.04	3.08	2.57	2.03	1.45	1.42
2008	9.44	9.01	8.93	9.17	8.80	7.87	7.39	7.25	6.71	5.64	4.92	4.14	3.18	2.56	2.06	1.46	1.46
2009	9.38	8.95	8.82	8.98	8.85	7.92	7.34	7.20	6.72	5.78	4.92	4.22	3.32	2.55	2.08	1.48	1.50
2010	9.32	8.90	8.72	8.80	8.85	7.97	7.31	7.13	6.71	5.92	4.91	4.34	3.42	2.55	2.09	1.49	1.55
2011	9.25	8.86	8.64	8.64	8.78	8.04	7.31	7.05	6.71	6.03	4.94	4.40	3.56	2.58	2.10	1.52	1.59

2012	9.17	8.82	8.58	8.50	8.66	8.13	7.34	6.98	6.70	6.10	5.01	4.44	3.64	2.66	2.10	1.54	1.63
2013	9.10	8.78	8.52	8.39	8.51	8.20	7.38	6.91	6.67	6.14	5.12	4.45	3.73	2.76	2.10	1.57	1.67
2014	9.01	8.74	8.47	8.29	8.34	8.25	7.43	6.87	6.63	6.16	5.26	4.45	3.81	2.88	2.11	1.59	1.71
2015	8.94	8.69	8.43	8.21	8.18	8.26	7.48	6.85	6.58	6.16	5.39	4.45	3.93	2.98	2.11	1.61	1.76
2016	8.87	8.63	8.39	8.14	8.04	8.21	7.56	6.86	6.52	6.16	5.50	4.48	3.98	3.10	2.14	1.62	1.80
2017	8.79	8.58	8.36	8.08	7.93	8.10	7.64	6.89	6.45	6.16	5.58	4.55	4.02	3.18	2.22	1.63	1.85
2018	8.68	8.52	8.33	8.04	7.83	7.97	7.72	6.94	6.40	6.15	5.62	4.66	4.04	3.27	2.31	1.63	1.89
2019	8.57	8.46	8.30	8.01	7.76	7.83	7.78	6.99	6.38	6.12	5.65	4.80	4.04	3.34	2.42	1.64	1.93
2020	8.57	8.51	8.25	7.94	7.71	7.69	7.69	7.09	6.41	6.08	5.68	4.87	4.04	3.39	2.46	1.67	1.97
2021	8.45	8.45	8.23	7.91	7.66	7.56	7.66	7.16	6.42	6.05	5.70	4.99	4.05	3.45	2.56	1.69	2.01
2022	8.33	8.40	8.20	7.88	7.62	7.47	7.60	7.22	6.44	6.03	5.71	5.08	4.09	3.50	2.65	1.73	2.05
2023	8.24	8.31	8.17	7.86	7.59	7.40	7.51	7.25	6.50	6.02	5.70	5.15	4.17	3.53	2.72	1.79	2.09
2024	8.14	8.21	8.13	7.85	7.56	7.35	7.40	7.26	6.58	6.01	5.68	5.20	4.28	3.54	2.80	1.87	2.13
2025	8.04	8.11	8.09	7.84	7.53	7.31	7.28	7.26	6.68	6.01	5.66	5.23	4.40	3.55	2.86	1.96	2.17
2026	7.94	8.01	8.05	7.83	7.52	7.27	7.17	7.25	6.76	6.03	5.64	5.25	4.52	3.57	2.92	2.05	2.23
2027	7.84	7.91	8.01	7.81	7.50	7.24	7.09	7.20	6.82	6.06	5.63	5.27	4.62	3.61	2.97	2.12	2.28
2028	7.73	7.83	7.93	7.80	7.49	7.22	7.04	7.13	6.86	6.12	5.63	5.27	4.69	3.69	3.01	2.19	2.36
2029	7.63	7.75	7.85	7.77	7.49	7.20	7.00	7.03	6.89	6.21	5.63	5.26	4.74	3.80	3.02	2.26	2.45
2030	7.54	7.67	7.77	7.75	7.49	7.19	6.97	6.93	6.90	6.31	5.64	5.25	4.78	3.92	3.04	2.32	2.55
2031	7.45	7.58	7.69	7.72	7.49	7.18	6.94	6.83	6.89	6.40	5.66	5.24	4.81	4.03	3.06	2.37	2.65
2032	7.36	7.50	7.60	7.69	7.49	7.18	6.93	6.77	6.86	6.47	5.70	5.24	4.83	4.13	3.11	2.42	2.75
2033	7.27	7.41	7.53	7.63	7.48	7.18	6.91	6.72	6.80	6.51	5.77	5.25	4.84	4.19	3.18	2.45	2.85
2034	7.19	7.33	7.47	7.56	7.47	7.19	6.91	6.70	6.72	6.55	5.86	5.25	4.84	4.25	3.29	2.47	2.97
2035	7.12	7.24	7.40	7.49	7.46	7.20	6.90	6.68	6.62	6.57	5.96	5.27	4.83	4.29	3.40	2.49	3.08
2036	7.04	7.17	7.32	7.42	7.43	7.21	6.90	6.66	6.54	6.57	6.06	5.30	4.83	4.32	3.51	2.52	3.20

2037	6.97	7.09	7.25	7.34	7.42	7.21	6.91	6.65	6.49	6.55	6.13	5.34	4.83	4.35	3.59	2.57	3.30
2038	6.91	7.02	7.17	7.29	7.37	7.22	6.92	6.65	6.45	6.50	6.18	5.42	4.85	4.36	3.66	2.64	3.39
2039	6.84	6.95	7.10	7.23	7.31	7.21	6.94	6.65	6.43	6.43	6.22	5.51	4.86	4.37	3.72	2.73	3.49
2040	6.78	6.88	7.03	7.17	7.25	7.21	6.95	6.66	6.42	6.35	6.25	5.62	4.89	4.37	3.76	2.83	3.58

Table S8. The global diarrheal incident cases and ASIR predicted by the XGBoost model, based on the adjustment of the proportions of population using safely managed drinking water services and safely managed sanitation services according to SDG 6

Year	Incidence cases (× 100,000)	ASIR (per 100,000)
2023	47014.53 (44647.12, 49625.49)	57687.41 (54755.31, 60886.84)
2024	52180.12 (49673.13, 54857.83)	63705.52 (60619.25, 66983.48)
2025	77421.22 (73215.00, 81956.46)	92851.64 (87722.29, 98338.23)
2026	107690.19 (101451.04, 114349.11)	129831.11 (122072.99, 137693.37)
2027	52415.47 (49745.08, 55655.42)	62301.12 (59150.83, 66319.35)
2028	49513.35 (47271.19, 52056.37)	58090.76 (55407.47, 61133.98)
2029	57476.70 (54496.60, 60556.04)	66807.58 (63418.20, 70436.32)
2030	122114.21 (114954.76, 129396.94)	140481.45 (131997.29, 148939.66)
2031	41302.01 (37361.46, 45469.54)	46742.23 (42130.23, 51613.17)
2032	41781.99 (37811.96, 45857.00)	46747.59 (41945.50, 51518.25)
2033	42256.75 (38230.82, 46438.14)	46750.46 (41993.19, 51565.76)
2034	42958.07 (38780.88, 47326.04)	47008.78 (42051.29, 52050.81)
2035	43677.36 (39337.25, 48323.75)	47265.45 (42441.88, 52777.07)
2036	44181.29 (39964.14, 49015.39)	47290.37 (42109.57, 52813.15)
2037	44697.51 (40564.02, 49504.61)	47377.78 (42364.31, 52897.58)
2038	45056.76 (40494.03, 49804.07)	47258.13 (42059.27, 52563.81)
2039	46508.69 (41504.23, 51785.05)	48394.58 (42784.59, 54192.18)
2040	45906.57 (41299.01, 50855.12)	47232.75 (42126.71, 52903.35)

SDG: Sustainable Development Goals

ASIR: age-standardized incidence rate
